# A Systems Genetics Approach Implicates *USF1*, *FADS3*, and Other Causal Candidate Genes for Familial Combined Hyperlipidemia

**DOI:** 10.1371/journal.pgen.1000642

**Published:** 2009-09-11

**Authors:** Christopher L. Plaisier, Steve Horvath, Adriana Huertas-Vazquez, Ivette Cruz-Bautista, Miguel F. Herrera, Teresa Tusie-Luna, Carlos Aguilar-Salinas, Päivi Pajukanta

**Affiliations:** 1 Department of Human Genetics, David Geffen School of Medicine at UCLA, Los Angeles, California, United States of America; 2 Department of Endocrinology and Metabolism, Instituto Nacional de Ciencias Médicas y Nutrición Salvador Zubirán, Mexico City, Mexico; 3 Surgery Division, Instituto Nacional de Ciencias Médicas y Nutrición Salvador Zubirán, Mexico City, Mexico; 4 Molecular Biology and Genomic Medicine Unit, Instituto de Investigaciones Biomédicas de la UNAM, Instituto Nacional de Ciencias Médicas y Nutrición Salvador Zubirán, Mexico City, Mexico; Princeton University, United States of America

## Abstract

We hypothesized that a common SNP in the 3' untranslated region of the upstream transcription factor 1 (*USF1*), rs3737787, may affect lipid traits by influencing gene expression levels, and we investigated this possibility utilizing the Mexican population, which has a high predisposition to dyslipidemia. We first associated rs3737787 genotypes in Mexican Familial Combined Hyperlipidemia (FCHL) case/control fat biopsies, with global expression patterns. To identify sets of co-expressed genes co-regulated by similar factors such as transcription factors, genetic variants, or environmental effects, we utilized weighted gene co-expression network analysis (WGCNA). Through WGCNA in the Mexican FCHL fat biopsies we identified two significant Triglyceride (TG)-associated co-expression modules. One of these modules was also associated with FCHL, the other FCHL component traits, and rs3737787 genotypes. This USF1-regulated FCHL-associated (URFA) module was enriched for genes involved in lipid metabolic processes. Using systems genetics procedures we identified 18 causal candidate genes in the URFA module. The FCHL causal candidate gene fatty acid desaturase 3 (*FADS3*) was associated with TGs in a recent Caucasian genome-wide significant association study and we replicated this association in Mexican FCHL families. Based on a *USF1*-regulated FCHL-associated co-expression module and SNP rs3737787, we identify a set of causal candidate genes for FCHL-related traits. We then provide evidence from two independent datasets supporting *FADS3* as a causal gene for FCHL and elevated TGs in Mexicans.

## Author Summary

By integrating a genetic polymorphism with genome-wide gene expression levels, we were able to attribute function to a genetic polymorphism in the *USF1* gene. The *USF1* gene has previously been associated with a common dyslipidemia, FCHL. FCHL is characterized by elevated levels of total cholesterol, triglycerides, or both. We demonstrate that this genetic polymorphism in *USF1* contributes to FCHL disease risk by modulating the expression of a group of genes functionally related to lipid metabolism, and that this modulation is mediated by *USF1*. One of the genes whose expression is modulated by *USF1* is *FADS3*, which was also implicated in a recent genome-wide association study for lipid traits. We demonstrated that a genetic polymorphism from the *FADS3* region, which was associated with triglycerides in a GWAS study of Caucasians, was also associated with triglycerides in Mexican FCHL families. Our analysis provides novel insight into the gene expression profile contributing to FCHL disease risk, and identifies *FADS3* as a new gene for FCHL in Mexicans.

## Introduction

Familial combined hyperlipidemia (FCHL) is a common atherogenic dyslipidemia conferring nearly two-fold greater risk for coronary heart disease [1]. FCHL is characterized by familial segregation of elevated fasting plasma triglycerides (TGs), total cholesterol (TC), or both [2],[3]. Another common characteristic of FCHL is elevated levels of fasting plasma apolipoprotein B (ApoB) [1]. In Mexico 12.6% of the general population have combined hyperlipidemia, suggesting that FCHL is a common dyslipidemia in the Mexican population [4].

We previously identified an association within the region of chromosome 1q21-q23 consistently linked to FCHL [5]–[9] with the associated linkage disequilibrium (LD) bin containing variants in upstream transcription factor 1 (*USF1*), and the adjacent gene, F11 receptor (*F11R*) [10]. The association with this LD bin containing *USF1* has been replicated for FCHL, and its component traits TC, TGs and low density lipoprotein cholesterol (LDL-C) in different populations [11]–[13], including Mexicans [14]. It has become evident that the SNP rs3737787 residing in the 3′ UTR of *USF1* captures the disease-associated signal, although its relationship to the causal polymorphism contributing to the etiology of FCHL is unknown. Previous studies involving direct sequencing, extensive genotyping and gene expression analyses of the *USF1* region have not identified any SNPs in the rs3737787 LD bin altering the coding sequence or the expression of *USF1* itself in fat [10] or lymphoblasts [11]. It has, however, been demonstrated that genes known to be regulated by *USF1* were differentially expressed between rs3737787 genotype groups in Finnish fat biopsies [15]. The direct targets of *USF1* were previously identified using chromatin immunoprecipitation and high-resolution promoter microarrays (ChIP-Chip) [16]. Indirect *USF1* target genes remain unknown.

We hypothesized that rs3737787 affects the function of *USF1*, and that this effect could be identified as the differential expression of genes directly or indirectly regulated by *USF1*. To test this hypothesis we first identified the direct and indirect targets of *USF1* by transiently over-expressing *USF1 in vitro* and assaying for differential expression using gene expression microarrays. Next we showed that there is overlap between the genes regulated by *USF1* and genes correlated with rs3737787 genotypes in 70 Mexican FCHL case/control fat biopsies. Adipose tissue is the major storage depot for TGs in the body, and has been suggested to play an important role in the hypertriglyceridemia component of FCHL [17]–[19]. It is also worth noting that TGs are the major focus of our study because the most associated FCHL-component trait with USF1 has been TGs [10],[14],[20]. Liver would also be a highly relevant tissue for gene expression studies of dyslipidemia due to its key role in the regulation of lipid metabolism. However, adipose tissue biopsies represent significantly less severe complications to the research subjects than liver biopsies. Adipose tissue has thus become the most studied tissue in FCHL because of its relevance to TG metabolism and the relatively minimal invasiveness of collecting subcutaneous fat biopsies [10], [15], [18], [21]–[26].

Recent systems genetic strategies that characterize interactions between genotype data and co-expression modules have successfully been applied to complex diseases [27]–[31]. Here we integrate weighted gene co-expression network analysis (WGCNA) with the SNP rs3737787 genotypes to identify an FCHL-related module and develop a systems genetic gene-screening strategy for causal candidate genes of FCHL related traits. We were able to identify two TG related co-expression modules, which are clusters of genes likely to be co-regulated by similar factors (i.e. transcription factors, genetic variants, or environmental effects). We tested the co-expression modules for association with FCHL, FCHL component traits, and rs3737787 genotypes. Several authors have suggested that genetic markers can be used as causal anchors for dissecting the causal relationships between traits. Since randomization is the most convincing method for establishing causal relationships between two correlated traits, it is natural to make use of genetically randomized genotypes (implied by Mendel's laws) to derive causality tests that are less susceptible to confounding by hidden variables [32]–[37]. Here we utilized the network edge orienting (NEO) software [37] coupled with the SNP rs3737787 genotypes to screen for causal candidate genes of FCHL and FCHL component traits. We followed-up a causal candidate gene for both FCHL and TGs, fatty acid desaturase 3 (*FADS3*), residing in the *FADS1-2-3* locus, which was recently implicated for TGs in a meta-analysis of genome-wide association studies for lipids in Caucasians [38]. In an independent Mexican FCHL dataset we replicate the association for TGs with the previously associated genome-wide SNP. To summarize, we identify two co-expression modules associated with TGs, the key component trait of FCHL. We then demonstrate that one of these co-expression modules is also associated with the SNP rs3737787. Our genetic marker based causal screening analysis implicates 18 FCHL causal candidate genes inside this module. Multiple lines of evidence validate the role of *FADS3* as a causal candidate gene for FCHL.

## Results

### Relating rs3737787 Correlated Gene Expression to *USF1*

We hypothesized that if the SNP rs3737787 tagged LD bin alters the function of the transcription factor *USF1*, the effect could be assayed on the downstream expression of *USF1* target genes, or the subsequent targets of those genes. We transiently over-expressed *USF1* in the HEK293T cell line and assayed for differential expression using gene expression microarrays to detect both direct and indirect targets of *USF1*. Over-expression of *USF1* was validated by western blot (Figure S1). In our over-expression analysis, we identified 2,897 genes that were directly or indirectly regulated by *USF1* over-expression *in vitro* (Table S1). We validated the differential expression of nine genes (*AGT*, *AK5*, *CNR1*, *GALR2*, *IGFBP7*, *KISSR1*, *PLXDC1*, *PLXNC1* and *PROK2*) using quantitative real-time PCR (Figure S2).

Next we utilized an independent gene expression dataset of 70 Mexican FCHL case/control fat biopsies whose clinical characteristics are given in Table 1. We observed 972 genes (gene expression profiles) significantly correlated with rs3737787 genotypes using an additive model (Table S2). The rs3737787 correlated genes had significant overlap both with i) the set of *USF1* regulated genes identified in our *USF1* over-expression experiment (n = 277; p-value = 3.0×10^−5^; fold-enrichment = 1.22) and ii) the previously published genes identified by ChIP-Chip [16] which are directly regulated by *USF1* (n = 117; p-value = 0.0051; fold-enrichment = 1.23). Furthermore, we also observed significant overlap between the rs3737787 correlated genes and the 2,189 genes differentially expressed between FCHL cases and normolipidemic controls (n = 245; p-value = 0.0030; fold-enrichment = 1.16) (Table S3), supporting a link from rs3737787 to FCHL etiology. Taken together, the overlap between rs3737787 correlated genes and genes regulated by *USF1* suggest that the effect of rs3737787 on FCHL is mediated through the transcription factor *USF1*.

**Table 1:** Clinical characteristics for Mexican FCHL fat biopsy cases/controls.

Clinical Trait	FCHL	Normolipidemic	P-value^A^
n (Male)	38 (21)	32 (15)	
Age	38±9.3	38±8.7	0.91
BMI	27±2.4	25±2.9	0.02
Total Cholesterol (mmol/L)	6.8±1.2	4.6±0.74	2.7×10^−13^
Triglycerides (mmol/L)	4.7±2.6	1.7±1.1	9.3×10^−8^
ApoB (g/L)	140±22	92±23	2.9×10^−12^

A Student's t-test p-value.

### Weighted Gene Co-Expression Network Analysis

**Figure 1:**
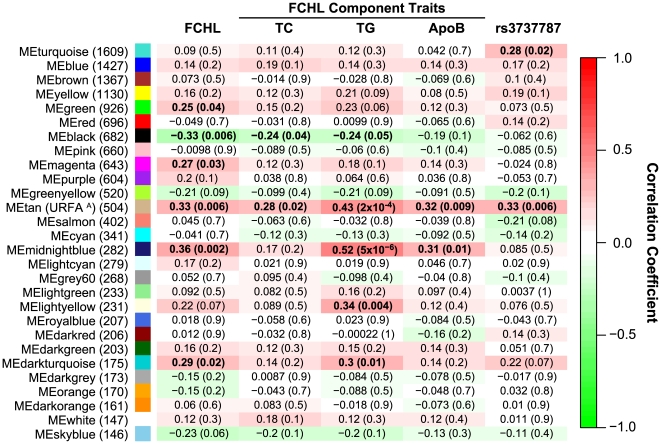
Correlation of module eigengenes (ME) with clinical traits and rs3737787 SNP genotypes. The rows are labeled by the ME color, and in parentheses is the number of genes in the module. The columns are labeled by SNP or clinical trait. The quantitative clinical traits, all except for FCHL which is qualitative, were corrected for significant covariates (age and/or sex) and standardized before use in analyses. The correlation coefficients are shown for each cell, and in parentheses is the p-value for the significance of the correlation. Cells are colorized based on the strength and sign of the correlation according to the scale on the right hand side of the figure. ^A^The tan module has been renamed to the *USF1*-regulated FCHL-associated (URFA) module.

The gene expression variation between individuals in the Mexican FCHL fat biopsies is due to a combination of genetic and environmental factors. Ascertainment for disease status enriches the cases with factors leading to hyperlipidemia, and contrasting the cases with the controls will allow these factors to be more easily identified. We utilized weighted gene co-expression networking analysis (WGCNA) to identify gene co-expression modules summarizing the main trends in variation for the Mexican FCHL case/control fat biopsies. The WGCNA method clustered the 14,942 gene expression probes on the Mexican FCHL case/control microarrays into 28 gene co-expression modules (Table S4). Each of the modules was labeled with a unique color as an identifier. We then characterized each module for enrichment of functionally-related genes (Table S5). To summarize the gene expression profiles of the highly correlated genes inside a given module, we use the first principal component, which is referred to as the module eigengene (ME). We tested the amount of trait variance explained by each ME (Table S6), which provides insight into how much influence each module has on a particular trait. We also tested each ME for correlation with the quantitative FCHL component traits: TC, TG and ApoB (Figure 1). Given the high level of correlation between the FCHL component traits (correlation TC & TG = 0.57; correlation TC & ApoB = 0.90; correlation TG & ApoB = 0.59), we approximate the total number of independent tests to be 2. Therefore, a Bonferroni correction would have to account for a total of 56 multiple comparisons (28 modules×2 independent tests). Thus using Bonferroni correction we would require a p-value less than or equal to 8.9×10^−4^ in order to maintain an experiment-wide type I error rate of 0.05. This highlights a major statistical advantage of our module based analyses over conventional differential expression analyses which have to account for tens of thousands of multiple comparisons.

### TG-Associated Module Eigengenes

We observed two modules (or more precisely MEs) significantly associated with TGs (tan ME correlation = 0.43, p-value = 2×10^−4^, Bonferroni-corrected p-value = 0.011; midnightblue ME correlation = 0.52, p-value = 5×10^−6^, Bonferroni-corrected p-value = 0.00028), the key component trait of FCHL. Interestingly, the associations of the tan and midnightblue ME with TGs were experiment-wide significant as they were less than the most conservative multiple comparison correction (Bonferroni-corrected p-value for 56 tests, p-value≤8.9×10^−4^). Using a multivariate regression model, we observed that the two TG associated MEs explained 30% of TG trait variance, which is relatively high considering this is a complex trait. Both the tan and midnightblue MEs were also marginally associated with qualitative FCHL disease status (tan ME correlation = 0.33, p-value = 0.006; midnightblue ME correlation = 0.36, p-value = 0.002), although these associations did not beat multiple testing correction (Figure 1). The association pattern of the tan ME with the FCHL component traits was characteristic of FCHL: elevated plasma levels of TC, TG and ApoB (TC correlation = 0.28, p-value = 0.02; TG correlation = 0.43, p-value = 2×10^−4^; ApoB correlation = 0.32, p-value = 0.009) (Figure 1). The midnightblue ME had a similar association pattern with the FCHL component traits (TG correlation = 0.52, p-value = 5×10^−6^; ApoB correlation = 0.31, p-value = 0.01), although it was not associated with TC (correlation = 0.17, p-value = 0.20) (Figure 1). We observed significant overlap (p-value≤2.7×10^−10^) between the FCHL differentially expressed genes and the tan and midnightblue module genes (tan module overlap p-value = 2.7×10^−10^; midnightblue module overlap p-value = 4.6×10^−31^) (Table 2). Given that FCHL is known to be a complex disease, it is not surprising that FCHL status is associated with multiple modules. Both of these modules are likely to be regulated by different genetic or environmental factors which contribute to the etiology of FCHL.

**Table 2:** Overlap of FCHL modules with FCHL differentially expressed gene list.

Module	FCHL Differentially Expressed Genes (n = 2,189)	
	Overlap (Enrichment^A^)	P-value
Tan (URFA^B^) (n = 504)	142 (1.60)	2.7×10^−10^
Midnightblue (n = 282)	130 (2.55)	4.6×10^−31^

A Enrichment is calculated as the fold-change in enrichment defined as the observed overlap divided by the expected overlap.

B The tan module has been renamed to the *USF1*-regulated FCHL-associated (URFA) module.

### rs3737787-Associated Module Eigengenes

Integrating the co-expression modules with genetic data provides insight into the function and regulation of each module. The turquoise and tan MEs were associated with the rs3737787 genotypes (turquoise ME correlation = 0.28, p-value = 0.02; tan ME correlation = 0.33, p-value = 0.006) (Figure 1). We observed a significant overlap between the rs3737787 correlated genes and both the turquoise and tan module genes (Table 3). The tan module was the only module to be associated with FCHL, the FCHL component trait signature, and rs3737787 genotypes. To emphasize the biological implications of the tan module, we renamed it as the *USF1*-regulated FCHL-associated (URFA) module. The turquoise module was not significantly associated with FCHL, suggesting that while the expression of turquoise module genes depends on rs3737787 genotypes, the gene functions of the turquoise module are less likely to be related to FCHL (Figure 1, Table S5). These results demonstrate that rs3737787 genotypes predict the expression of many genes, some of which predispose to FCHL.

**Table 3:** Overlap of rs3737787 modules with genes differentially expressed by the rs3737787 genotypes.

Module	rs3737787 Correlated Genes (n = 972)	
	Overlap (Enrichment^A^)	P-value
Turquoise (n = 1609)	345 (2.72)	1.6×10^−82^
Tan (URFA^B^) (n = 504)	188 (4.70)	1.4×10^−87^

A Enrichment is calculated as the fold-change in enrichment defined as the observed overlap divided by the expected overlap.

B The tan module has been renamed to the *USF1*-regulated FCHL-associated (URFA) module.

We should point out that our sample size of 70 individuals provides 80% power to detect a significant association (p-value≤0.05) with correlation coefficient = 0.33, and only limited power to rule out associations with a correlation coefficient<0.33. To have 80% power to detect a significant association (p-value≤0.05) when the true correlation coefficient = 0.2 requires 194 individuals, and when the true correlation coefficient = 0.1 requires 783 individuals. Therefore our sample size does not permit us to rule out associations of smaller magnitude (correlation coefficient<0.33). However, our sample size of 70 individuals provides enough power to detect significant moderate correlations (correlation coefficient≥0.33) between eigengenes, SNPs, and clinical traits.

### *USF1*-Regulated FCHL-Associated (URFA) Module Characterization

The URFA module contains 504 co-expressed genes that are enriched for the gene ontology (GO) Biological Process categories of Cellular Lipid Metabolic Processes and Lipid Metabolic Processes (p-values = 1.0×10^−5^ and 9.3×10^−6^; Benjamini-Hochberg multiple comparison corrected p-values = 0.022 and 0.039) (Table S5). The URFA ME accounts for 10% of the variation of FCHL, 6% of TC, 17% of TG, and 9% of ApoB (Table S6). The fact that the URFA ME was associated with rs3737787 reflects the fact that most module genes are at least partially regulated by this SNP. To evaluate whether the module causally affects FCHL component traits, we utilized the Network Edge Orienting (NEO) R software package [37] which takes SNP genotypes as input into structural equation models that compute causal edge orienting scores (referred to as LEO.NB scores). Since we are only considering a single SNP (rs3737787) we computed LEO.NB.*SingleMarker* scores for the causal orientation of a ME→trait. The LEO.NB.*SingleMarker* score is a relative model fitting index for the causal model (rs3737787→ME→trait) relative to alternative causal models, and the larger the value for the LEO.NB.*SingleMarker* score the stronger the evidence that this causal orientation is correct. We required that the our causal model fit at least two times better than the next best alternative model, which equates to a LEO.NB.*SingleMarker* score of 0.3 [28] (please see the Materials and Methods for a detailed description). We found sufficient evidence to infer a causal relationship between the URFA ME and fasting plasma TGs levels (LEO.NB.*SingleMarker* score = 0.31), the key component trait of FCHL. The LEO.NB.*SingleMarker* score for the URFA ME to FCHL was 0.25. We then used the NEO software to prioritize genes inside the URFA module by calculating the LEO.NB.*SingleMarker* scores evaluating the causal model (rs3737787→gene expression→trait). We identified 18 causal candidate genes for FCHL, and 171 causal candidate genes for fasting plasma TGs levels (Table S7). We observed 13 causal candidate genes for both FCHL and TG. None of the URFA module genes showed evidence for causally affecting TC or ApoB. Since our interest was in FCHL disease status, we characterized the 18 causal candidate genes for FCHL disease status as potential candidate genes for genetic association studies in Mexican FCHL families (Table 4).

**Table 4:** Genes causally linked to FCHL (rs3737787→Gene Expression→FCHL) with LEO.NB.*SingleMarker* score≥0.30 from the URFA module.

Affymetrix ProbeSet	Gene Name	Associations	Network Connectivity	Kathiresan GWAS Minimum Regional P-values^B^	
				LDL	TG
214033_at	*ABCC6*	CAD, HDL, PSE, TG	34.1	7.5×10^−3^ (rs212077)	1.2×10^−4^ (rs9924674)
203925_at	*GCLM*	AvgIMT, CHD, MI, T2D, VCI	33.1	8.4×10^−4^ (rs2281525)	4.1×10^−3^ (rs12070273)
209740_s_at	*PNPLA4*	NA	28.3	NA^C^	NA^C^
204257_at	*FADS3*	HDL, PUFA, TG	27	4.2×10^−4^ (rs174549)	3.3×10^−7^ (rs102275)
227117_at	*XPOT*	NA	20.3	1.2×10^−3^ (rs10878151)	1.1×10^−3^ (rs11504159)
212799_at	*STX6*	NA^A^	17.2	1.5×10^−3^ (rs17299701)	9.1×10^−3^ (rs6658713)
204057_at	*IRF8*	HEPC	15.3	2.6×10^−4^ (rs903194)	5.7×10^−4^ (rs4843966)
214152_at	*CPR8*	NA	12.9	7.9×10^−3^ (rs12902248)	1.6×10^−2^ (rs4774780)
214696_at	*C17orf91*	NA	12.1	7.9×10^−5^ (rs17761734)	6.6×10^−4^ (rs2955626)
205404_at	*HSD11B1*	BC, HT, T2D	11.1	1.6×10^−4^ (rs6659502)	2.1×10^−3^ (rs7536585)
236664_at	*AKT2*	MetS, T2D, TC/HDL	9.8	7.0×10^−3^ (rs10412191)	4.6×10^−4^ (rs4803342)
203625_x_at	*SKP2*	NA	8.9	9.7×10^−3^ (rs1610218)	4.8×10^−4^ (rs6895261)
50374_at	*C17orf90*	NA	8.5	2.5×10^−3^ (rs11150780)	1.8×10^−2^ (rs7210742)
229144_at	*KIAA1026*	NA	5.3	9.3×10^−4^ (rs9442193)	3.5×10^−3^ (rs12141589)
205452_at	*PIGB*	NA	3.6	7.9×10^−3^ (rs12902248)	1.6×10^−2^ (rs4774780)
209268_at	*VPS45A*	NA^A^	2.7	5.1×10^−3^ (rs7537292)	1.3×10^−2^ (rs6587552)
214252_s_at	*CLN5*	NCL	2.2	3.5×10^−3^ (rs851251)	9.5×10^−3^ (rs1537063)
228641_at	*CARD8*	AZ, RA	0.8	4.9×10^−5^ (rs3760802)	1.6×10^−4^ (rs11669775)

AvgIMT indicates average intima-media thickness; AZ, Alzheimer's disease; BC, body composition; CAD, coronary artery disease; CHD, coronary heart disease; HDL, low levels of high-density lipoprotein cholesterol; HEPC, hepatitis-C; HT, hypertension; MI, myocardial infarction; NCL, neuronal ceroid-lipofuscinosis; PSE, pseudoxanthoma elasticum; PUFA, polyunsaturated fatty acids; T2D, type II diabetes; TC/HDL, total cholesterol/high-density lipoprotein cholesterol ratio; TG, hypertriglyceridemia; and VCI, vascular cognitive impairment.

A *STX6* and *VSP45A* proteins are known to physically interact.

B Minimum P-value from a region spanning 1 Mbp on either side of the gene from Kathiresan et al., 2009 [38].

C *PNPLA4* resides on the X chromosome, and there weren't any SNPs tested within the specified region in the Kathiresan et al., 2009 [38].

### Replication of Association to the *FADS1-2-3* Gene Locus

Fatty acid desaturase 3 (*FADS3*) was 1 of the 18 genes causally linked to FCHL. Interestingly, variation from the *FADS1-2-3* genomic region was previously associated with TGs in a recent meta-analysis of GWAS in Caucasians [38]. We chose to follow-up the SNP rs174547 residing in the *FADS1-2-3* locus which was significantly associated with TGs at the genome-wide level in this previous meta-analysis [38]. The same study demonstrated that the SNP rs102275, in complete LD with rs174547 in Caucasians, predicted the expression of *FADS1* and to a lesser extent *FADS3* in human liver [38]. We hypothesized that because *FADS3* expression was associated with FCHL, any variation affecting the expression of *FADS3* could be associated with FCHL or an FCHL component trait, especially TGs. Therefore we genotyped both rs174547 and rs102275 in the Mexican FCHL case/control fat biopsies (n = 70). We observed a large allele frequency discrepancy between the Mexican and Caucasian populations for both rs174547 and rs102275, which was also present in the HapMap Phase 3 data (rs174547: Caucasian T allele = 0.66, Mexican T allele = 0.34; rs102275: Caucasian T allele = 0.65, Mexican T allele = 0.30). The LD between rs174547 and rs102275 was reduced in the Mexican samples compared to the Caucasian population (Mexican r^2^ = 0.90; Caucasian r^2^ = 1.0), indicating that the SNPs are no longer completely redundant in the Mexican population. Therefore, we report the results for both SNPs (Table 5). In the 70 Mexican individuals undergoing fat biopsies, the major alleles of both SNPs were associated with hypertriglyceridemia as defined by TGs dichotomized by the 90^th^ age-sex specific population percentiles (rs174547 p-value = 0.024, rs102275 p-value = 0.012). Despite the switch in allele frequencies, the association was observed with the same allele (C) as in Caucasians and thus, the direction of the association is identical to the previous genome-wide association study [38]. We then proceeded to test these two SNPs for replication in the Mexican families with FCHL and its component traits (Table 6). The major alleles of both SNPs were associated with hypertriglyceridemia as defined by TGs dichotomized by the 90^th^ age-sex specific population percentiles (rs174547 p-value = 0.0071, rs102275 p-value = 0.0034), and again the direction of the association was consistent with the previous genome-wide association study [38]. The association in the Mexican FCHL families with rs102275 was Bonferroni corrected significant (rs102275 corrected p-value = 0.034). These results replicate the findings for the *FADS1-2-3* locus in the Mexican population.

**Table 5:** Replication of association evidence for *FADS1-2-3* locus in 70 Mexican FCHL case/control fat biopsies with qualitative lipid traits.

SNP	Minor Allele	MAF	FCHL		FCHL Component Traits					
					TC		TG		ApoB	
			Beta	P-value	Beta	P-value	Beta	P-value	Beta	P-value
rs174547	T	0.33	−0.59±0.35	0.09	−0.42±0.35	0.23	−0.94±0.42	0.024	−0.75±0.37	0.044
rs102275	T	0.29	−0.62±0.35	0.072	−0.5±0.35	0.16	−1.12±0.45	0.012	−0.74±0.37	0.044

**Table 6:** Replication of association evidence for *FADS1-2-3* locus in Mexican FCHL families with qualitative lipid traits.

SNP	Minor Allele	MAF	FCHL		FCHL-Related Traits					
					TC		TG		ApoB	
			Z	P-value	Z	P-value	Z	P-value	Z	P-value
rs174547	T	0.28	−0.55	0.58	−0.37	0.7	−2.69	0.0071	−0.29	0.77
rs102275	T	0.23	−0.65	0.52	−1.08	0.28	−2.93	0.0034^A^	−0.91	0.36

A Multiple testing corrected significant (Bonferroni correction≤0.0063 = 0.05/[2 SNPs×4 traits]).

### Predicting the Expression of *FADS1* and *FADS3* in Adipose Tissue

Of the three fatty acid desaturase genes in the *FADS1-2-3* locus, we observed that only the expression of *FADS3* was associated with FCHL disease status in adipose tissue (204257_at correlation = 0.31, p-value = 0.0084; 216080_s_at correlation = 0.31, p-value = 0.0092). This result indicates that increasing expression of *FADS3* is associated with increased risk for FCHL. We then tested whether the two SNPs rs174547 and rs102275 predicted the expression of *FADS1* and/or *FADS3* in the Mexican FCHL case/control fat biopsies. We observed a marginally significant prediction of *FADS1* expression (rs174547 correlation = 0.26, p-value = 0.027; rs107225 correlation = 0.20, p-value = 0.089), and little to no evidence for prediction of *FADS3* expression (rs174547 correlation = −0.15, p-value = 0.22; rs102275 correlation = −0.14, p-value = 0.25). These results were consistent with the results from Caucasian human liver samples where rs102275 predicted the expression of *FADS1* and to a lesser extent *FADS3* expression.

## Discussion

By integrating both genetic and transcriptome profile data we were able to attribute a function to the FCHL associated, rs3737787-tagged LD bin. We first provide evidence that the SNP rs3737787 has an effect on global expression profiles, and further evidence suggesting that this effect is mediated by *USF1*. We then identified two TG-associated co-expression modules one of which (the URFA module) was also associated with the FCHL, the other FCHL component traits, and rs3737787 genotypes. The URFA module was enriched for the GO Biological Process categories Lipid Metabolic Process and Cellular Lipid Metabolic process. The URFA module also contained eighteen genes causally linked to FCHL. One of the causal genes for FCHL, *FADS3*, coincides with a previous genome-wide significant association signal for TGs [38]. We demonstrate that the genome-wide associated SNP also has an effect on TGs in the Mexican FCHL families. In summary, we demonstrate the function of the rs3737787 LD bin, construct and characterize a co-expression network for Mexican FCHL case/control fat biopsies, and demonstrate that the loci identified as causal can harbor variants associated with FCHL or FCHL component traits.

There are three SNPs from the rs3737787 tagged LD bin residing within the USF1 gene. Although our current study does not allow us to pinpoint which of these three USF1 SNPs in LD is the actual causative SNP, we were able to demonstrate that an allele-dependent effect of the rs3737787 tagged LD bin on downstream genes is mediated by USF1 and not the adjacent gene F11R. To identify the causal SNP in USF1, additional functional studies are warranted that investigate allele-dependent effects of each of the three SNPs on USF1 isoform and protein levels.

Gene co-expression networking methods have been successfully applied in a variety of different settings [39]–[46]. We utilized weighted gene co-expression network analysis (WGCNA) for the following reasons. First, it is assumed that co-expression is a biological process, and as such a co-expression module is a set of genes that are likely to be co-regulated by similar factors (e.g. shared transcription factors, genetic variants or environmental effects). Second, modules (and corresponding module eigengenes) represent a biologically motivated data reduction method which greatly alleviates the multiple comparison problem inherent in genomic data analysis. Third, we provide annotation tables for module membership (Table S4) and URFA module causal candidate genes measures (Table S7), which provides a resource for other investigators. Our unbiased module detection analysis identified a module that was associated with rs3737787 genotypes, fasting plasma TG levels, FCHL disease status, and contained genes that are causal drivers of TG levels. It is difficult to ascertain if the URFA module can be identified in other tissues. Nevertheless, we consider that the identification of the URFA module in fat does further implicate a biological role of fat tissue in FCHL, although we cannot establish whether these FCHL modules are tissue-specific.

Our approach provides insight to how the SNP rs3737787 confers increased risk for FCHL, by demonstrating that it regulates the URFA module eigengene which in turn contributes to increased TG levels, a key component trait of FCHL. The URFA module can be considered rs3737787-genotype dependent, while the other TG associated midnightblue module was not dependent on rs3737787 genotypes. The variation regulating other modules remains to be discovered.

We conducted a literature search for links between the 18 FCHL causal candidate genes from the URFA module to lipid related phenotypes and/or atherogenic processes. Three of the FCHL causal candidate genes were directly related to lipid phenotypes (*ABCC6*, *AKT2*, *HSD11B1*), and two others were likely to be related to lipid phenotypes (*FADS3*, *PNPLA4*) via homology. We also identified genes which were related to atherogenic processes such as inflammation (*CARD8*, *ICSBP1*, *STX6*) and reactive oxygen species (*GCLM*). Among the 18 genes there are also putative genes and genes with little known function. The function of these novel candidate genes can be studied *in vivo* or *in vitro*, and will be useful in understanding how the URFA module contributes to the etiology of FCHL. Importantly, some of the genes causally linked to FCHL (*ABCC6*, *AKT2*, *FADS3*, *GCLM*, *HSD11B1*) have already been associated with FCHL related traits in humans (Table 3 and Table S8). Studies in mice have also demonstrated that genetic manipulation of *Abcc6*, *Akt2* and *Hsd11b1* affect FCHL component traits or related phenotypes [47]–[49]. Together these data support a causal association between the 18 causal candidate genes from the URFA module and FCHL. Although we present strong statistical and biological evidence in support of our findings, we want to emphasize that additional biological validation studies are warranted.

Identifying the associated gene(s) from the genetic association evidence for the *FADS1-2-3* locus in humans is complicated by the fact that multiple LD blocks have been associated with several different but related lipid traits [38], [50]–[57], as well as coronary artery disease [55] and nonfatal myocardial infarction [57]. It has been established that both *FADS1* and *FADS2* function as a fatty acid desaturase [58],[59] in the pathway generating polyunsaturated fatty acids from the essential Omega-3 and Omega-6 fatty acids. The function of *FADS3* is unknown, although it shares 52% sequence identity with *FADS1* and 62% with *FADS2* [60], suggesting that *FADS3* may also function as a fatty acid desaturase. Our findings demonstrate that *FADS3* expression is regulated by rs3737787 genotypes in adipose tissue, and that this differential expression causes increased risk for FCHL. Neither *FADS1* nor *FADS2* were regulated by rs3737787 genotypes, nor were the expression of these two genes associated with FCHL. Future studies to identify the function of *FADS3* will be required to elucidate the mechanisms by which differential regulation of *FADS3* in adipose tissue predisposes to FCHL.

Our systems genetic analysis integrated SNP genotypes with gene expression levels to unravel the complex disorder that is familial combined hyperlipidemia. With this study we have identified new intriguing causal candidate genes for genetic studies with FCHL, and we provide causal candidate genes for FCHL related component traits (Table S7). These data may form the starting point for future studies that further elucidate the etiology of FCHL. The fact that we were able to account for 30% of fasting plasma TG trait variance is very promising, and that it required the expression of hundreds of genes to observe this demonstrates that FCHL is a complex and heterogeneous disorder. The URFA module is regulated by the SNP rs3737787 in *USF1*, suggesting that variation in the *USF1* gene acts as a master regulator of genes contributing to the FCHL phenotype. Our analysis of gene expression and SNP genotype data from few but carefully selected individuals demonstrates that a well designed systems genetic study can lead to insights on complex disease traits.

## Materials and Methods

### *USF1* Over-Expression

Transient transfection and over-expression of *USF1* were conducted in HEK293T cells, which express the *USF1* protein (Figure S1). The *USF1* coding region was PCR amplified from cDNA (forward-primer = ATGAAGGGGCAGCAGAAAACAG, reverse primer = TTAGTTGCTGTCATTCTTGATG) and cloned into pcDNA4/HisMax TOPO TA mammalian expression vector (Invitrogen, Cat. No. K864-20). Plasmid for transient transfection was prepared using the Endo Free Plasmid Kit Mega (Qiagen, Cat. No. 12363). Over-expression of *USF1* was validated by western blot using Anti-HisG-HRP (Invitrogen, Cat No. R941-25) and Rabbit Anti-*USF1* (Santa-Cruz, Cat. No. sc-8983) (Figure S1). Total RNA was collected using the RNeasy Plus Mini Kit (Cat No. 74134) 48 hours after transient transfection of HEK293T cells. Microarray hybridization has been described previously [22]. After applying the quality control pipeline, a total of 16,570 probesets passed and were utilized in the analyses. Differential expression between *USF1* and empty vector control transfected cells was analyzed using a Student's T-test (Table S1). The differential expression of nine genes (*AGT*, *AK5*, *CNR1*, *GALR2*, *IGFBP7*, *KISSR1*, *PLXDC1*, *PLXNC1* and *PROK2*) was successfully validated using quantitative real-time PCR (Figure S2 and Table S9). The transient transfection and over-expression of *USF1* microarray data can be accessed in MIAME compliant format from NCBI GEO database (GSE17300).

### Mexican FCHL Case/Control Gene Expression Microarrays

We collected 70 Mexican FCHL case/control fat biopsies from umbilical subcutaneous adipose tissue under local anesthesia. Each participant provided written informed consent. The study design was approved by the ethics committees of the INCMNSZ and UCLA. Clinical characteristics are summarized in Table 1. Differential expression for a subset of these microarrays has been validated by qRT-PCR previously [22],[25]. We have previously described in detail the procedures for RNA extraction [10],[15],[25] and microarray hybridization [22]. After applying the microarray quality control pipeline, a total of 14,942 probesets passed and were utilized in analyses. The microarray data can be accessed in MIAME compliant format from the NCBI Gene Expression Omnibus (GEO) database (GSE17170).

### Microarray Quality Control Pipeline

A rigorous quality control pipeline was developed to prepare the gene expression microarray data for analysis. First, the CEL files were imported into R 2.8.0 using the justRMA function of the Affymetrix library from Bioconductor, which applies background subtraction and quantile normalization. An alternate CDF file (U133Plus2msk.cdf.gz) was used to exclude mis-targeted and nonspecific probes from the microarrays [61], and probesets with less than 7 remaining probes were also excluded. Probesets with more than 50% absent calls were excluded, as calculated using the panp package from Bioconductor. Finally, the ComBat library in R was used to correct for batch effect [62].

### Mexican Microarray Analyses

Only residuals of the clinical traits corrected for significant covariates, such as age and sex, were used in analyses. There was minimal relatedness between individuals, but to avoid confounding due to relatedness all analyses with the Mexican FCHL cases/controls were corrected for kinship. The lmekin function from the kinship package in R was utilized for the analyses of differential gene expression. Differential expression of genes based on the FCHL status was assayed by regressing FCHL status with the covariates age, sex and kinship on the expression of each gene. Differential expression of genes based on the additive model for rs3737787 genotypes was assayed by regressing the rare allele counts with the covariates age, sex, FCHL status and kinship on the expression of each gene. Significance of the overlap of independent gene lists was calculated using Fisher's exact test. We report two-tailed p-values and fold change of enrichment to demonstrate whether there are more or less overlapping genes than expected. Functional enrichment analysis was conducted using the DAVID software package [63]. The Benjamini-Hochberg (B–H) multiple testing corrected p-values are reported. Power calculations were conducted utilizing pwr.r.test function from the pwr package in R.

### Weighted Gene Co-Expression Network Analysis (WGCNA)

For the 70 Mexican FCHL case/control fat biopsies, a weighted gene co-expression network was constructed using the blockwiseModules function from the WGCNA package in R [64]. We utilized residuals of gene expression corrected for age, sex and kinship in the WGCNA. The blockwiseModules function allows the entire dataset of 14,942 genes to be utilized in the construction of the weighted gene co-expression network, by splitting the genes into sets of less than 2,000 genes [64]. For each set of genes the pair-wise correlation matrix is computed, and using the power function the correlations are weighted to a power of β [65]. An advantage of WGCNA is the fact that the results are highly robust with respect to the choice of the parameter β [64]. WGCNA is designed to identify modules of densely interconnected genes by searching for genes with similar patterns of connectivity with other genes, which can be summarized as the topological overlap between genes [65]. Topological overlap is then calculated from the weighted co-expression matrix, and turned into a dissimilarity measure (1 - topological overlap) for average linkage hierarchical clustering. The dynamic tree-cutting algorithm [66] is then used to identify modules of co-expressed genes. After all blocks have been processed, a gene is reassigned to another module if it is found to have higher connectivity to this other module, and modules whose eigengenes are highly correlated are merged [64]. Genes that are not assigned to a module are assigned to the grey module. The maximum block size was set at 2,000 genes; a power (β) was set to 6 as this was found to be optimal (and also happens to be the default setting); minimum module size was set at 100; the minimum height for merging modules was set at 0.2; and the maximum height at which the tree could be cut was set to 1. All the other parameters were left at default settings.

### Causality Testing

To infer causal flow between genetic markers, gene expression traits and clinical traits, we utilized the single.marker.analysis function from the Network Edge Orienting (NEO) package in R [37]. We also utilized residuals of gene expression corrected for age, sex and kinship in causality testing. There are five different models for causal flow that are tested in the single.marker.analysis function. The model of interest for these studies infers a causal flow anchored by the SNP rs3737787 which affects the expression of a gene or gene co-expression module that in turn alters a clinical trait (M→A→B). The fit of this model was assessed using the local structural equation model (SEM) based, edge orienting, next best single marker (LEO.NB.*SingleMarker*) score, which is the log_10_ probability of this model divided by the log_10_ probability of the next best fitting alternative model [37]. We chose to use the same threshold as utilized by Presson et al., where the LEO.NB.*SingleMarker* score was required to be positive and at least twice as probable as the next best alternative model, a LEO.NB.SingleMarker score of 0.3≈log_10_(2) [31]. Thus any LEO.NB.*SingleMarker* score greater than or equal to 0.3 was considered sufficient to infer causal flow.

### Mexican FCHL Families

A total of 872 individuals from 74 Mexican FCHL families were collected at Instituto Nacional de Ciencias Medicas y Nutricion, Salvador Zubiran, as described previously [22],[25]. The 90^th^ age-sex specific Mexican population percentiles for TGs and TC were used to determine the affection status, as described previously [22],[25]. Each participant provided written informed consent. The study design was approved by the ethics committees of the INCMNSZ and UCLA.

### Genotyping

The rs3737787, rs174547 and rs102275 SNPs were genotyped using the pyrosequencing technique (Biotage). Primers for PCR were designed using the Primer3 software [67], and labeled detection primers using SNP Primer Design software (Biotage). Sequencing was performed using an ABI 377XL automated DNA sequencer (Applied Biosystems). All SNPs had at least 90% genotype call rate in each study sample, and when necessary individuals were sequenced to achieve at least a 90% genotype call rate for each SNP.

### Genetic Association Testing

All genetic association testing was conducted using qualitative phenotypes, where the continuous FCHL related phenotypes are dichotomized by the 90^th^ age-sex specific percentiles of TGs and TC. Association for the Mexican FCHL fat biopsy case/control study sample was calculated by regressing genotypes in an additive model to the dichotomized trait of interest using logistic regression with the glm function in R. We utilized the Family Based Association Testing (FBAT) software package to test for association in the Mexican FCHL families [68]. Because the *FADS1-2-3* locus resides in a linked region, the -e option of FBAT was used. This option utilizes a null model of no association in the presence of linkage. FBAT implements a generalization of the transmission disequilibrium test (TDT) [69] in the extended pedigrees. Thus, FBAT is not sensitive to population stratification or admixture [68]–[70]. The FBAT –e option also corrects for cryptic relatedness. Due to these features FBAT -e provides a robust test for association in these extended Mexican families.

## Supporting Information

Figure S1 Western blot demonstrating HEK293T endogenous *USF1* expression and *USF1* over-expression at the protein level. Protein collected from HEK293T cells transfected with *USF1* is labeled *USF1* overexpression, protein collected from HEK293T cells transfected with an empty vector is labeled Empty Vector, and protein collected from HEK293T cells which were not tranfected is labeled HEK293T. Protein amounts were quantitated using BCA assay and 10 µg of protein was loaded for each lane. (A) Probed with Anti-HisG-HRP antibody which should detect only the over expressed *USF1* protein with a polyhistidine tag. (B) Probed with Anti-*USF1* antibody, which will detect over expressed as well as endogenous *USF1* and demonstrates the relative amount of over-expression of *USF1*. This also demonstrates that the HEK293T cell line does express *USF1* protein at a detectable level by western blot.
(1.00 MB EPS)

Figure S2 qRT-PCR validation of genes identified as differentially expressed due to *USF1* over-expression on microarrays. The validation genes were: *AGT* = angeotensin, *AK5* = adenylate kinase 5, *CNR1* = canaboid receptor 1, *GALR2* = galanin receptor 2, *IGFBP7* = insulin-like growth factor binding protein 7, *KISS1R* = KISS1 receptor, *PLXDC1* = plexin domain containing 1, *PLXNC1* = plexin C1, and *PROK2* = prokineticin 2. The endogenous housekeeping controls were: *B2M* = beta-2-microglobulin, and *HPRT* = hypoxanthine phosphoribosyltransferase. Student's t-test p-values were calculated between *USF1* over-expression and Empty Vector control, and a single asterisk (*) indicates a p-value≤0.05, a double asterisk (**) indicates a p-value≤0.005, and a triple asterisk (***) indicates a p-value≤0.0005.
(0.99 MB EPS)

Table S1 Genes differentially expressed by *USF1* over-expression in HEK293T cells (p-value≤0.05).
(1.80 MB PDF)

Table S2 Genes correlated to FCHL-associated rs3737787 genotypes (additive model) in Mexican FCHL case/control fat biopsies (p-value≤0.05).
(0.64 MB PDF)

Table S3 Genes differentially expressed between FCHL cases and normolipidemic controls in Mexican FCHL case/control fat biopsies (p-value≤0.05).
(1.42 MB PDF)

Table S4 WGCNA module membership for all probes from the Mexican FCHL case/control fat biopsies.
(11.40 MB ZIP)

Table S5 Functionally enriched annotation terms for the set of genes comprising each co-expression module (Benjamini-Hochberg corrected p-value≤0.05).
(0.11 MB PDF)

Table S6 Trait variance explained by WGCNA co-expression module eigengenes (Adjusted R^2^).
(0.02 MB PDF)

Table S7 Causality testing results for URFA module genes terminating in FCHL, TC, TG, and ApoB.
(0.38 MB PDF)

Table S8 Previous association evidence with lipid or atherogenic traits for genes causally linked to FCHL from the URFA module.
(0.07 MB PDF)

Table S9 qRT-PCR primers for validation of genes identified as differentially expressed due to *USF1* over-expression on microarrays.
(0.01 MB PDF)
