# Supplementary material for: A Systems Genetics Approach Implicates USF1, FADS3, and Other Causal Candidate Genes for Familial Combined Hyperlipidemia
Source: PLoS Genet. 2009 Sep 11;5(9):e1000642. doi: 10.1371/journal.pgen.1000642 (PMC2730565; doi:10.1371/journal.pgen.1000642)
Supplement: Table S1 — Genes differentially expressed by USF1 over-expression in HEK293T cells (p-value≤0.05). (1.80 MB PDF) [file pgen.1000642.s003.pdf]

Table S1. Genes differentially expressed by USF1 over-expression in HEK293T cells (p-value ≤ 0.05).

Probe ID	Entrez ID	Fold-Change	T Statistic	Degrees of Freedom	P-value
218488_at	8891	0.93	-49.70482	3.68	2.41E-06
212209_at	23389	0.97	-24.13402	3.83	2.49E-05
221816_s_at	51131	1.25	22.18223	3.99	2.52E-05
219257_s_at	8877	1.36	20.91317	3.97	3.25E-05
203701_s_at	55621	1.09	18.72748	3.97	5.07E-05
223441_at	26503	1.07	17.94752	3.95	6.17E-05
233329_s_at	51315	0.88	-22.44875	3.48	6.79E-05
205548_s_at	10950	0.97	-16.79517	3.97	7.74E-05
205935_at	2294	1.11	42.67046	2.63	8.29E-05
211774_s_at	25974	0.93	-16.15706	4.00	8.58E-05
219078_at	55105	0.88	-15.5137	4.00	0.0001008
205205_at	5971	1.10	15.49357	4.00	0.0001016
218628_at	51019	0.86	-19.42782	3.51	0.000107
218683_at	58155	0.91	-14.87036	4.00	0.0001191
213529_at	146542	1.18	14.65078	4.00	0.0001268
227862_at	388610	1.46	16.52613	3.68	0.000137
201963_at	2180	1.11	22.8196	3.11	0.0001442
234040_at	3070	0.79	-18.51189	3.38	0.0001613
225784_s_at	55906	0.92	-13.9659	3.91	0.0001765
209723_at	5272	1.15	17.32755	3.42	0.0001866
227870_at	57722	1.21	13.26111	3.96	0.0001981
219538_at	54554	0.89	-13.49756	3.91	0.000199
220631_at	64172	0.93	-18.40452	3.26	0.000209
238461_at	317649	1.13	15.02736	3.59	0.0002284
228095_at	9678	0.90	-13.4298	3.82	0.0002342
219742_at	80758	1.16	12.92342	3.92	0.0002348
221520_s_at	55143	1.05	13.46542	3.81	0.000237
225932_s_at	3181	0.96	-12.42602	4.00	0.0002421
223886_s_at	81847	0.96	-13.4085	3.75	0.0002649
219863_at	51191	1.15	13.83051	3.67	0.000267
230483_at	NA	0.91	-16.73685	3.24	0.0002931
239165_at	NA	0.86	-12.77298	3.78	0.0003024
223157_at	84273	1.06	19.79688	2.96	0.0003054
213117_at	55958	0.95	-16.23037	3.27	0.0003086
218632_at	79654	1.26	11.70181	3.99	0.0003107
212296_at	10213	0.95	-16.07083	3.26	0.0003246
223608_at	84288	1.57	15.04705	3.38	0.0003271
225835_at	6558	1.09	11.6389	3.96	0.0003285
202364_at	4601	1.07	13.92675	3.52	0.0003311
203569_s_at	8481	0.92	-15.6734	3.28	0.0003377
220009_at	79836	1.16	18.30801	2.98	0.0003675
37986_at	2057	1.13	12.20813	3.75	0.0003737
209577_at	5833	1.12	11.23117	3.96	0.0003791
226756_at	168455	0.94	-11.3298	3.90	0.0003954
227375_at	81573	0.95	-11.38885	3.88	0.0004005
203715_at	6905	0.93	-16.19493	3.12	0.0004076
205085_at	4998	0.93	-12.6923	3.59	0.0004129

56197_at	254863	1.15	18.37777	2.92	0.0004136
204404_at	6558	1.10	11.20255	3.89	0.0004196
224443_at	84791	0.86	-10.58289	4.00	0.0004541
230563_at	221002	1.09	10.82064	3.93	0.0004571
202809_s_at	65123	0.94	-10.56082	3.99	0.0004621
209155_s_at	22978	0.93	-10.82889	3.90	0.0004731
219494_at	25788	0.89	-12.9258	3.46	0.0004732
225327_at	56204	1.14	16.46103	3.00	0.0004916
227554_at	402560	0.93	-14.88371	3.16	0.0004938
210757_x_at	1601	1.14	10.6644	3.91	0.000494
223174_at	84280	0.93	-10.4603	3.96	0.0004951
221934_s_at	55152	1.08	11.90619	3.60	0.0005085
226782_at	253512	1.07	10.58468	3.89	0.0005234
230449_x_at	NA	0.86	-12.06736	3.55	0.0005256
202713_s_at	9692	0.89	-10.19662	3.98	0.0005379
219288_at	57415	0.92	-13.95963	3.22	0.0005495
206833_s_at	98	1.60	16.75787	2.90	0.000557
226458_at	NA	0.86	-12.62	3.40	0.0005605
51192_at	54961	1.09	12.40044	3.44	0.0005669
228736_at	113510	0.89	-11.71145	3.56	0.0005692
201414_s_at	4676	1.05	10.12576	3.94	0.0005788
223294_at	51260	0.97	-9.781676	3.98	0.0006294
243507_s_at	149840	0.86	-12.36544	3.36	0.0006434
207495_at	9364	0.96	-10.97548	3.63	0.0006478
210058_at	5603	1.11	10.49276	3.74	0.000657
207124_s_at	10681	1.08	9.545339	3.99	0.0006786
38671_at	23129	1.11	10.08819	3.81	0.0006921
204224_s_at	2643	1.19	24.78885	2.34	0.0006986
239790_s_at	23404	0.93	-11.42585	3.48	0.0007047
200868_s_at	55905	0.92	-9.633111	3.93	0.0007063
219063_at	79169	1.04	10.24157	3.74	0.0007133
227970_at	80045	1.20	9.485589	3.95	0.0007344
202794_at	3628	1.13	9.624823	3.90	0.0007357
226170_at	2140	0.92	-9.4691	3.95	0.0007368
221646_s_at	79844	1.12	9.556093	3.92	0.0007377
226055_at	27106	1.19	9.742038	3.84	0.0007583
224616_at	1783	1.05	10.1245	3.73	0.0007615
222817_at	80270	1.16	12.2062	3.28	0.0007626
220366_at	64100	1.45	13.09322	3.13	0.0007803
210410_s_at	4439	0.77	-11.02811	3.48	0.0007909
226021_at	157506	1.04	9.605688	3.84	0.0008037
226073_at	219854	0.93	-9.151168	3.98	0.0008087
209859_at	114088	1.19	9.34447	3.91	0.0008161
219517_at	80237	1.22	11.56456	3.35	0.0008172
208882_s_at	51366	0.92	-10.25169	3.64	0.0008182
236080_at	NA	0.83	-9.785912	3.76	0.0008269
213497_at	25841	1.13	9.045853	4.00	0.0008313
228904_at	3213	0.95	-9.615811	3.80	0.0008417
210142_x_at	10211	1.07	9.189491	3.93	0.0008458
230185_at	79725	0.84	-9.834882	3.72	0.0008494
232398_at	284992	0.81	-10.84747	3.47	0.0008496
222683_at	56254	0.96	-10.1116	3.64	0.0008516

207628_s_at	114049	1.07	11.48434	3.32	0.0008713
223000_s_at	50848	1.11	13.24283	3.03	0.0008784
211383_s_at	22884	1.12	11.59075	3.29	0.0008805
220974_x_at	81855	1.17	15.10119	2.81	0.0008856
205090_s_at	51172	1.12	11.72813	3.26	0.000895
36552_at	26005	0.89	-9.088082	3.91	0.0009062
205189_s_at	2176	0.95	-12.82014	3.07	0.0009098
209331_s_at	4149	1.07	8.997552	3.94	0.0009108
203047_at	6793	1.06	8.903663	3.95	0.0009267
212614_at	84159	0.94	-8.989185	3.92	0.0009333
203450_at	25776	0.92	-8.953685	3.93	0.0009342
214106_s_at	2762	1.07	8.877479	3.96	0.0009348
242070_at	NA	1.26	13.01982	3.03	0.0009372
229521_at	168455	0.91	-9.278228	3.81	0.0009415
224741_x_at	60674	0.97	-8.979266	3.91	0.0009418
226398_s_at	118924	0.90	-8.768132	3.98	0.0009539
226926_at	93099	0.97	-9.180084	3.82	0.0009651
213019_at	26953	0.92	-8.798764	3.96	0.0009673
213847_at	5630	1.15	8.644236	4.00	0.0009872
225777_at	89958	1.08	9.38884	3.73	0.0009924
202996_at	57804	0.92	-8.584168	3.99	0.0010275
223424_s_at	7589	0.91	-8.888352	3.87	0.0010333
230973_at	400745	1.27	8.649665	3.95	0.0010401
212894_at	6832	1.05	10.01992	3.51	0.0010522
216266_s_at	10565	0.92	-8.885288	3.85	0.0010535
220262_s_at	65989	1.10	9.671694	3.60	0.0010542
220892_s_at	29968	0.96	-11.53904	3.18	0.0010637
226800_at	84455	0.89	-8.468221	4.00	0.0010658
220358_at	55509	1.24	8.948245	3.81	0.0010746
235346_at	139341	0.97	-10.33905	3.41	0.0010848
218303_x_at	51315	0.90	-9.959021	3.49	0.001096
201705_at	5713	0.96	-8.433759	3.99	0.0010965
226333_at	3570	1.30	16.01244	2.62	0.0010965
39966_at	10675	1.14	10.75489	3.31	0.0010966
201375_s_at	5515	1.02	19.69967	2.37	0.0010999
211255_x_at	9191	0.97	-11.93792	3.09	0.0011003
221524_s_at	58528	1.06	10.38198	3.39	0.0011018
221838_at	84861	0.90	-8.790937	3.84	0.0011048
224841_x_at	60674	0.97	-8.49279	3.94	0.0011214
208819_at	4218	0.96	-8.517461	3.93	0.0011285
219446_at	55188	0.87	-8.399619	3.97	0.00113
209056_s_at	988	0.96	-10.46777	3.33	0.001163
203410_at	10947	0.92	-9.238905	3.64	0.0011782
204333_s_at	175	0.89	-8.61253	3.84	0.001202
208928_at	5447	1.16	8.196864	4.00	0.0012071
236918_s_at	151827	1.18	8.556312	3.85	0.0012104
200954_at	527	1.08	12.05674	3.01	0.0012167
203499_at	1969	1.22	21.2569	2.25	0.0012265
235484_at	375743	0.90	-8.715195	3.77	0.0012372
225868_at	91107	1.32	10.98939	3.17	0.0012464
201920_at	6574	1.03	8.189646	3.97	0.0012515
228948_at	2043	0.97	-10.51955	3.25	0.001271

204925_at	1497	1.10	8.954107	3.67	0.0012747
203409_at	1643	0.78	-20.84291	2.24	0.0012956
228121_at	7042	1.25	8.229928	3.90	0.001323
203659_s_at	10206	0.93	-8.29836	3.87	0.0013233
227846_at	11245	1.15	10.88309	3.15	0.0013296
222474_s_at	56993	1.04	8.572626	3.76	0.0013353
230748_at	9120	1.20	8.45726	3.80	0.0013425
219566_at	79156	1.14	8.012053	3.98	0.0013513
229344_x_at	57494	0.88	-8.283441	3.86	0.0013524
203821_at	1839	1.36	16.18347	2.50	0.0013531
229933_at	148304	0.83	-9.953536	3.32	0.0013858
231975_s_at	166968	0.92	-8.798574	3.64	0.0013921
36711_at	23764	1.31	14.37402	2.64	0.0013934
200006_at	11315	0.99	-7.899147	3.99	0.0014093
220121_at	55180	0.85	-11.04774	3.08	0.0014117
226741_at	51234	1.10	17.78351	2.37	0.0014118
200696_s_at	2934	1.17	8.348809	3.79	0.0014199
223231_at	83940	0.93	-10.82211	3.12	0.0014215
215411_s_at	10758	1.07	8.486232	3.74	0.0014219
38037_at	1839	1.39	14.35581	2.63	0.0014357
228977_at	53342	1.17	8.09608	3.88	0.0014414
219639_x_at	56965	0.94	-9.60515	3.37	0.0014475
201696_at	6429	0.96	-18.9676	2.28	0.0014662
203462_x_at	8662	1.05	7.810645	3.99	0.0014693
204170_s_at	1164	1.02	7.997393	3.90	0.0014723
213392_at	124152	0.88	-8.126322	3.84	0.001477
40829_at	23038	1.09	8.168903	3.82	0.0014792
219066_at	60490	0.87	-8.201542	3.81	0.0014815
210010_s_at	6576	1.07	8.607375	3.65	0.0014893
223316_at	83643	1.10	7.763528	3.99	0.0014924
227758_at	85004	0.90	-11.12253	3.01	0.0015295
230815_at	389765	0.89	-8.267185	3.75	0.0015299
211115_x_at	8487	0.93	-9.343787	3.40	0.001532
228661_s_at	NA	0.80	-9.624576	3.33	0.0015328
213094_at	57211	1.25	9.069335	3.48	0.0015334
213008_at	55215	0.95	-7.763136	3.97	0.0015364
225044_at	115024	1.02	22.14616	2.11	0.0015381
202399_s_at	10239	0.94	-7.939562	3.88	0.0015459
204347_at	387851	1.13	9.601272	3.32	0.001559
233141_s_at	54879	0.79	-23.03811	2.07	0.0015707
222995_s_at	57414	0.93	-13.33771	2.68	0.001576
34225_at	7469	1.04	13.06767	2.71	0.0015776
220770_s_at	63920	0.84	-9.879735	3.24	0.0015814
213836_s_at	55062	1.10	7.883568	3.88	0.0015835
232581_x_at	59269	1.09	7.704229	3.96	0.0015878
219556_at	80178	0.95	-7.754627	3.93	0.0015967
235785_at	463	0.92	-8.023037	3.81	0.0016036
217977_at	51734	1.12	12.9599	2.70	0.0016393
203855_at	22911	1.08	8.042188	3.78	0.0016423
208249_s_at	23483	0.96	-8.558862	3.58	0.0016429
228916_at	143884	0.95	-7.513556	4.00	0.0016806
225308_s_at	85461	0.97	-7.517117	3.99	0.0016955

217414_x_at	3040	1.38	8.41518	3.61	0.0016964
218126_at	55177	1.09	22.16419	2.07	0.0017002
200620_at	9528	0.97	-7.830407	3.84	0.0017035
214608_s_at	2138	0.91	-8.198009	3.68	0.0017043
207749_s_at	5523	0.89	-8.443787	3.59	0.0017047
201250_s_at	6513	1.10	7.785955	3.85	0.0017073
235142_at	127557	1.07	9.796358	3.20	0.0017095
208103_s_at	81611	1.07	7.482064	3.99	0.0017148
218584_at	79600	0.89	-12.40518	2.74	0.0017271
207558_s_at	5308	0.93	-7.511247	3.97	0.0017325
203053_at	10286	0.97	-7.452139	3.99	0.0017418
219406_at	79078	0.90	-7.519824	3.95	0.0017511
223187_s_at	94101	0.98	-7.470066	3.98	0.0017514
222839_s_at	64895	0.96	-9.52291	3.25	0.0017521
222701_s_at	79145	1.04	7.592573	3.92	0.0017561
203468_at	8558	0.87	-9.214331	3.33	0.0017569
219217_at	79731	0.95	-14.39859	2.51	0.0017643
232201_at	85409	1.09	7.497789	3.96	0.0017643
226543_at	63915	0.90	-8.999212	3.38	0.0017695
204416_x_at	341	1.19	7.720049	3.84	0.0017819
226324_s_at	26160	0.86	-8.087043	3.68	0.0017879
239853_at	147700	1.16	7.402595	3.98	0.0018153
203658_at	788	0.86	-10.01785	3.11	0.0018253
200863_s_at	8766	0.98	-9.247322	3.29	0.0018262
230418_s_at	57452	1.17	7.630131	3.85	0.0018375
233177_s_at	25953	1.17	7.392645	3.96	0.0018565
218270_at	79590	0.96	-7.317258	4.00	0.0018593
213951_s_at	29893	0.90	-7.486577	3.91	0.0018633
235199_at	NA	1.14	8.72752	3.42	0.0018636
201131_s_at	999	1.36	15.12506	2.42	0.0018656
213625_at	387032	0.85	-8.947328	3.35	0.0018702
211699_x_at	3040	1.33	10.51932	2.98	0.0018842
204504_s_at	8479	0.95	-15.37881	2.39	0.0018888
205333_s_at	9986	1.02	7.339409	3.96	0.0019142
203097_s_at	9693	0.93	-7.262684	4.00	0.0019155
212828_at	8871	1.09	7.299218	3.98	0.001917
202738_s_at	5257	0.94	-7.528774	3.86	0.0019179
201599_at	4942	1.03	7.408582	3.92	0.0019196
200971_s_at	27230	1.03	7.514296	3.86	0.0019303
209239_at	4790	0.95	-9.033607	3.30	0.0019354
203556_at	22882	1.12	8.884305	3.35	0.0019367
206173_x_at	2553	1.07	7.686126	3.77	0.0019599
231164_at	440331	1.19	7.302242	3.95	0.0019663
202136_at	10771	1.11	9.869129	3.08	0.0019674
235339_at	83852	0.76	-8.380264	3.49	0.0019699
226565_at	147184	0.95	-13.33607	2.56	0.0019711
225117_at	284058	0.98	-8.515693	3.44	0.001973
211114_x_at	8487	0.91	-7.260934	3.97	0.0019737
223173_at	83985	1.13	12.15412	2.69	0.0019852
226745_at	285440	0.91	-11.87563	2.72	0.0020062
228429_x_at	64147	1.15	7.631379	3.76	0.0020168
213701_at	91298	0.98	-7.530378	3.81	0.0020179

222256_s_at	8681	0.90	-7.171413	3.99	0.0020281
242931_at	NA	1.19	14.73068	2.41	0.0020337
209721_s_at	25900	1.07	7.138668	4.00	0.0020369
204828_at	5883	1.02	7.627952	3.75	0.0020508
239435_x_at	134549	1.04	8.679002	3.36	0.0020528
202670_at	5604	1.06	8.943485	3.28	0.0020697
226337_at	92344	0.87	-11.72744	2.72	0.0020705
203966_s_at	5494	1.05	8.745194	3.31	0.0021256
203570_at	4016	1.25	8.139111	3.51	0.00213
228019_s_at	51023	0.95	-7.335944	3.85	0.0021337
227213_at	134637	0.92	-11.11884	2.80	0.0021344
219313_at	54762	0.81	-8.848505	3.28	0.0021372
201769_at	9685	0.95	-7.613488	3.72	0.0021384
209112_at	1027	0.97	-8.84476	3.28	0.0021398
202429_s_at	5530	1.04	8.482117	3.39	0.0021423
224441_s_at	85015	0.85	-7.0694	3.98	0.0021477
226947_at	375513	0.95	-13.88332	2.45	0.0021592
201957_at	4660	0.91	-7.072565	3.97	0.0021603
219581_at	80746	0.94	-8.433085	3.38	0.0021914
200777_s_at	9689	1.03	9.517966	3.08	0.0022167
214016_s_at	6421	0.93	-11.84016	2.67	0.0022193
204193_at	1120	0.88	-6.972705	3.99	0.0022378
207035_at	7781	1.09	7.29448	3.81	0.0022583
201174_s_at	54386	1.02	7.062473	3.93	0.0022605
210347_s_at	53335	0.94	-7.609731	3.66	0.0022625
209586_s_at	58497	0.95	-7.739859	3.61	0.0022627
217839_at	10342	0.99	-8.905822	3.21	0.0022647
226285_at	4076	0.96	-7.772852	3.59	0.0022683
205379_at	874	1.23	7.43605	3.73	0.0022803
221335_x_at	56006	0.92	-7.143278	3.88	0.0022892
204233_s_at	1119	1.11	14.73105	2.35	0.0022897
223576_at	51250	0.90	-10.14497	2.92	0.0022956
222872_x_at	64859	0.93	-7.417638	3.73	0.0023013
201424_s_at	8451	1.11	9.580912	3.03	0.0023113
229810_at	NA	0.87	-7.53128	3.67	0.0023286
215023_s_at	5189	0.84	-7.137966	3.86	0.0023396
210764_s_at	3491	1.07	6.881275	4.00	0.0023433
232164_s_at	83481	1.08	6.87748	4.00	0.0023459
225455_at	117143	0.97	-7.54199	3.66	0.0023538
224759_s_at	90488	0.97	-6.866227	4.00	0.0023634
220206_at	79830	0.89	-6.973808	3.93	0.0023692
239142_at	317671	0.91	-7.287508	3.76	0.0023773
201714_at	7283	0.97	-6.866704	3.98	0.0023917
212694_s_at	5096	1.09	7.127928	3.82	0.0024394
226760_at	203411	0.96	-7.704171	3.55	0.0024398
212378_at	2618	0.96	-6.920532	3.93	0.0024489
212408_at	26092	1.08	11.63654	2.63	0.0024623
225992_at	8028	0.95	-7.054107	3.85	0.0024668
36888_at	23354	0.94	-6.963429	3.89	0.0024675
204804_at	6737	0.89	-6.971222	3.88	0.0024819
226135_at	54887	0.92	-6.888043	3.92	0.0024961
218340_s_at	55236	0.95	-18.91471	2.05	0.0024989

208688_x_at	8662	1.04	9.061447	3.10	0.0025014
218905_at	55656	0.97	-13.78053	2.38	0.0025041
208786_s_at	81631	1.04	6.896136	3.92	0.0025076
218803_at	55743	1.04	6.942833	3.89	0.0025099
222103_at	466	1.05	7.778896	3.49	0.0025284
202024_at	439	1.06	8.031641	3.40	0.0025293
236664_at	NA	0.85	-8.808531	3.15	0.0025429
226780_s_at	154791	0.94	-7.119195	3.77	0.002555
218109_s_at	64747	1.12	6.718548	4.00	0.0025556
209824_s_at	406	0.88	-6.778589	3.96	0.0025727
211036_x_at	51433	0.97	-12.20229	2.53	0.0025923
230467_at	339456	1.15	6.859573	3.90	0.0026022
226975_at	55599	0.83	-6.738141	3.97	0.0026068
209803_s_at	7262	1.37	15.20328	2.24	0.0026355
203767_s_at	412	1.32	8.177389	3.31	0.0026426
204465_s_at	9118	0.94	-8.253708	3.28	0.002643
200889_s_at	6745	0.99	-6.758848	3.93	0.0026524
204141_at	7280	1.05	6.922361	3.84	0.0026593
226258_at	196394	0.82	-7.256237	3.67	0.00266
202382_s_at	10007	1.05	6.669235	3.98	0.0026621
233208_x_at	53981	0.96	-14.59588	2.28	0.0026679
235320_at	84100	0.89	-7.084443	3.75	0.0026754
244455_at	343450	0.85	-6.747655	3.93	0.0026758
218723_s_at	28984	1.53	11.94261	2.54	0.0026792
209717_at	7813	1.05	7.74014	3.44	0.0027069
202336_s_at	5066	1.05	6.666398	3.96	0.002733
225851_at	2342	0.95	-6.631777	3.97	0.0027442
213677_s_at	5378	0.90	-8.52726	3.17	0.0027544
210346_s_at	57396	0.93	-6.893325	3.82	0.0027548
212365_at	4430	1.05	6.824143	3.85	0.0027564
223551_at	5570	1.29	9.321996	2.96	0.0027587
223339_at	93974	0.93	-8.558008	3.15	0.002767
201301_s_at	307	1.18	7.156388	3.67	0.0027734
209710_at	2624	1.09	6.577861	3.99	0.0027928
203140_at	604	0.92	-6.746855	3.88	0.0027934
235039_x_at	286826	1.15	6.919833	3.79	0.0027965
218399_s_at	55038	0.97	-9.387913	2.93	0.0028233
224461_s_at	84883	1.24	6.804579	3.84	0.002829
229667_s_at	3218	1.29	6.537536	4.00	0.0028295
236302_at	22843	0.92	-7.345395	3.56	0.0028419
210779_x_at	8487	0.90	-7.483286	3.50	0.0028436
212409_s_at	26092	1.13	7.460758	3.51	0.002846
202380_s_at	4820	0.91	-8.346592	3.19	0.0028469
204780_s_at	355	1.30	7.98987	3.31	0.0028493
209745_at	10229	0.89	-7.185369	3.63	0.0028599
204454_at	23641	0.96	-7.140513	3.65	0.0028657
202491_s_at	8518	0.96	-6.869717	3.78	0.0028867
218617_at	54802	0.91	-6.793689	3.82	0.0028898
232289_at	92080	1.19	10.21717	2.75	0.0028903
223560_s_at	55471	1.05	7.793199	3.36	0.0029003
213376_at	22890	1.06	6.992829	3.70	0.0029142
211745_x_at	3040	1.41	9.626139	2.85	0.0029211

226043_at	26086	1.07	7.232241	3.58	0.0029346
213233_s_at	55958	0.96	-6.517538	3.97	0.0029439
219126_at	55274	1.05	12.58814	2.41	0.0029636
40225_at	2580	1.05	6.468404	3.99	0.0029682
213572_s_at	1992	1.09	10.28106	2.71	0.0029875
209575_at	3588	0.94	-6.730241	3.82	0.0029932
219847_at	79885	1.09	7.400112	3.48	0.00301
201617_x_at	800	1.11	6.507667	3.95	0.003014
228037_at	5914	0.91	-11.89807	2.48	0.0030156
203820_s_at	10643	1.07	7.096158	3.62	0.0030184
218686_s_at	64285	1.13	6.423657	4.00	0.0030252
211678_s_at	55905	0.94	-6.667856	3.84	0.0030369
219155_at	26207	1.09	8.197522	3.18	0.0030426
36994_at	527	1.07	12.53288	2.40	0.0030589
220721_at	80110	0.91	-6.407365	3.99	0.0030734
201749_at	1889	1.06	7.674285	3.35	0.0030997
205004_at	55922	0.98	-6.431422	3.95	0.0031324
202949_s_at	2274	1.06	7.933194	3.24	0.003135
206721_at	57821	0.87	-6.563126	3.86	0.0031494
204950_at	22900	0.89	-7.26572	3.50	0.0031537
206140_at	9355	1.11	6.352256	3.99	0.003161
227214_at	57120	0.98	-6.359379	3.99	0.0031624
213644_at	201134	0.92	-8.118619	3.17	0.0031869
204157_s_at	23387	0.93	-7.15789	3.53	0.0032
230240_at	8444	0.93	-6.319643	4.00	0.0032079
219469_at	79659	0.92	-6.346494	3.98	0.0032115
229928_at	NA	1.20	6.511816	3.87	0.0032131
219372_at	28981	0.87	-9.995118	2.72	0.0032182
219019_at	55367	0.90	-7.435674	3.40	0.00323
227361_at	9953	1.25	12.32071	2.39	0.0032359
218883_s_at	79682	0.98	-6.297651	4.00	0.0032574
218205_s_at	2872	1.07	7.181551	3.50	0.0032578
219596_at	56906	0.92	-6.689303	3.75	0.0032594
210114_at	27130	0.89	-6.288339	4.00	0.0032688
201145_at	10456	0.98	-6.39717	3.92	0.0032815
217979_at	27075	1.03	6.987468	3.58	0.0032855
212603_at	10240	0.95	-17.09475	2.02	0.0032855
218336_at	5202	0.96	-6.728281	3.71	0.0033129
223684_s_at	23583	0.94	-6.885934	3.63	0.0033178
226334_s_at	130872	0.89	-9.864475	2.71	0.0033347
207338_s_at	7752	0.94	-6.255039	4.00	0.0033412
201393_s_at	3482	1.07	6.543061	3.81	0.0033413
200052_s_at	3608	1.03	6.25188	4.00	0.0033466
234863_x_at	26271	0.97	-7.301518	3.42	0.0033619
218080_x_at	11124	0.94	-6.308381	3.95	0.0033664
211212_s_at	5001	0.91	-6.723691	3.69	0.0033736
228044_at	387923	0.81	-8.412715	3.03	0.0033799
217845_x_at	25994	1.03	7.207377	3.45	0.0033977
221927_s_at	83451	0.97	-6.73765	3.67	0.0034331
219531_at	55722	0.93	-6.265724	3.95	0.0034491
202593_s_at	51573	0.96	-7.145741	3.46	0.0034514
219627_at	79970	0.81	-10.32339	2.61	0.0034729

200878_at	2034	1.07	11.97154	2.38	0.0034752
201348_at	2878	1.15	16.33519	2.03	0.0034832
224994_at	817	1.11	8.04353	3.11	0.0034958
234995_at	152185	0.91	-6.16978	4.00	0.0035054
231824_at	55132	1.18	9.015885	2.85	0.0035066
209332_s_at	4149	1.04	6.186246	3.98	0.0035138
228990_at	85028	1.17	8.292966	3.03	0.0035221
220998_s_at	81622	1.11	6.231183	3.95	0.0035375
218924_s_at	1486	0.88	-6.2174	3.95	0.0035456
201421_s_at	79084	0.96	-8.487914	2.97	0.0035477
202963_at	5993	0.96	-7.795456	3.18	0.0035504
227603_at	284459	0.89	-6.694642	3.65	0.0035772
227160_s_at	79133	0.92	-7.531963	3.27	0.0035832
217920_at	10905	1.06	8.798355	2.89	0.0035852
203089_s_at	27429	1.08	9.010172	2.84	0.003594
223665_at	84517	1.16	6.163205	3.97	0.0035987
203643_at	2077	0.94	-7.56729	3.25	0.0036114
218268_at	64786	0.95	-8.697607	2.90	0.0036243
214074_s_at	2017	1.09	13.64801	2.20	0.0036351
201302_at	307	1.20	6.621524	3.67	0.0036392
226738_at	124997	1.10	7.773583	3.17	0.003641
203909_at	10479	0.96	-7.599789	3.23	0.0036456
201420_s_at	79084	0.96	-6.11018	3.99	0.0036535
212636_at	9444	0.93	-6.870667	3.53	0.0036609
202126_at	8899	0.96	-7.198031	3.38	0.003676
224747_at	92912	1.05	12.44	2.30	0.0036986
218616_at	57117	0.95	-6.952063	3.48	0.0036992
225736_at	26263	0.93	-8.630046	2.90	0.0037167
225602_at	152007	1.14	6.331481	3.81	0.0037399
226239_at	129303	1.12	6.063348	4.00	0.0037418
204346_s_at	11186	0.93	-9.545411	2.69	0.0037719
204957_at	5001	0.90	-6.67904	3.60	0.003773
240382_at	1832	1.34	15.66572	2.03	0.0037878
203428_s_at	25842	0.95	-6.920014	3.47	0.0037939
221258_s_at	81930	0.90	-7.980044	3.06	0.003801
202481_at	9249	1.16	6.1069	3.94	0.0038103
203062_s_at	9656	0.94	-10.65996	2.49	0.0038503
227481_at	154043	1.07	6.127798	3.91	0.0038787
200720_s_at	10121	0.94	-9.905508	2.60	0.0039075
204274_at	9166	0.95	-6.437987	3.70	0.0039089
220060_s_at	55010	0.90	-6.967815	3.42	0.0039126
202462_s_at	9879	0.94	-6.452306	3.68	0.0039252
226796_at	116236	1.16	8.815198	2.81	0.0039296
200880_at	3301	1.03	6.779758	3.50	0.0039371
201040_at	2771	1.09	6.249842	3.80	0.0039621
227455_at	221545	0.90	-6.358797	3.73	0.0039641
212406_s_at	4661	0.96	-6.116115	3.88	0.0039786
200718_s_at	6500	1.04	13.93774	2.12	0.0040184
225321_s_at	29990	0.92	-6.089641	3.89	0.0040261
212271_at	5594	1.05	7.347884	3.23	0.0040287
230032_at	64172	0.92	-5.982923	3.96	0.0040398
210947_s_at	4437	0.94	-6.223654	3.79	0.0040402

229338_at	23350	0.75	-6.343189	3.72	0.0040453
202890_at	9053	1.11	6.039536	3.92	0.0040515
204369_at	5290	0.89	-6.609162	3.56	0.0040522
57516_at	92595	0.89	-7.581781	3.14	0.0040538
212813_at	83700	0.98	-6.794018	3.46	0.0040599
226547_at	7994	0.97	-6.570074	3.58	0.0040718
56256_at	51092	0.90	-6.990237	3.37	0.0040827
213480_at	8674	0.93	-6.42243	3.65	0.0041148
203050_at	7158	0.92	-8.545384	2.84	0.0041205
207153_s_at	11146	0.95	-7.574223	3.12	0.0041388
226151_x_at	9946	0.95	-6.684731	3.50	0.0041451
213624_at	10924	1.08	5.959827	3.95	0.0041498
232147_at	84464	0.87	-6.023792	3.90	0.004162
226080_at	85464	0.96	-7.875043	3.02	0.0041769
200867_at	55905	0.93	-7.937892	3.00	0.0041859
219358_s_at	55803	1.07	8.698012	2.79	0.0041974
208055_s_at	26091	0.94	-5.872767	4.00	0.0042031
205486_at	10420	1.15	8.00425	2.97	0.0042233
209539_at	9459	1.06	7.58587	3.10	0.0042504
207559_s_at	9203	0.93	-5.87171	3.98	0.0042621
209481_at	54861	0.95	-6.146478	3.78	0.0042623
224830_at	11051	1.01	5.869428	3.98	0.0042634
226271_at	54332	0.88	-5.847336	4.00	0.0042673
207627_s_at	7024	1.03	6.071729	3.83	0.0042709
209106_at	8648	0.94	-7.045891	3.29	0.0043059
213398_s_at	56948	0.96	-5.832284	4.00	0.0043066
213059_at	90993	1.09	7.602508	3.08	0.0043158
213959_s_at	23322	0.85	-6.176958	3.75	0.0043178
220311_at	29104	0.92	-5.833283	3.99	0.0043255
227964_at	83786	1.04	6.093855	3.80	0.0043314
209361_s_at	57060	1.07	5.829942	3.99	0.0043337
238794_at	119392	0.90	-6.934854	3.33	0.0043404
210201_x_at	274	1.11	5.818919	4.00	0.0043523
226004_at	81928	1.18	7.386649	3.15	0.0043524
203564_at	2189	0.94	-7.918389	2.97	0.0043528
224785_at	283991	1.05	5.831279	3.98	0.0043639
204045_at	9338	1.04	9.995315	2.51	0.0043697
213785_at	55705	0.92	-5.870011	3.95	0.0043735
228829_at	11016	0.96	-8.30257	2.85	0.0043973
225554_s_at	51434	0.91	-6.029077	3.81	0.0044412
228374_at	27291	0.88	-5.922244	3.89	0.0044422
229380_at	NA	1.15	5.842021	3.95	0.004444
219241_x_at	54961	1.12	11.13151	2.34	0.0044696
218932_at	54680	0.97	-8.963978	2.68	0.0044801
227688_at	57631	0.94	-5.775508	3.99	0.0044802
226070_at	286257	0.96	-6.430956	3.55	0.004482
209252_at	23438	0.91	-6.329857	3.61	0.0044919
204751_x_at	1824	1.10	6.671383	3.42	0.0044929
222369_at	79829	0.94	-7.226833	3.18	0.0044956
218371_s_at	55269	0.83	-13.6779	2.08	0.0044982
218046_s_at	51021	0.95	-5.90113	3.89	0.0044992
219459_at	55703	0.93	-6.38116	3.57	0.0045095

227882_at	79147	0.93	-6.712283	3.40	0.0045139
235274_at	NA	0.82	-7.913023	2.94	0.0045196
235889_at	NA	0.89	-5.934941	3.86	0.0045241
226919_at	57226	0.94	-5.853735	3.91	0.0045534
205944_s_at	8218	1.10	7.623626	3.02	0.004556
228354_at	118812	0.88	-6.902694	3.30	0.0045573
65718_at	25960	0.91	-6.303941	3.60	0.0045603
218819_at	26512	0.94	-5.791971	3.96	0.0045628
203017_s_at	117178	0.96	-10.23253	2.45	0.0045654
202631_s_at	10513	0.89	-8.779407	2.71	0.0045665
207417_s_at	7730	0.84	-6.141646	3.70	0.0045721
222262_s_at	55500	0.93	-5.775018	3.97	0.0045815
203605_at	6729	1.06	6.443838	3.52	0.004585
212788_x_at	2512	1.02	6.728497	3.37	0.0045859
203282_at	2632	1.11	9.464436	2.57	0.0045949
238054_at	113622	1.21	8.78164	2.70	0.004605
222251_s_at	26205	0.91	-5.727245	4.00	0.0046168
204102_s_at	1938	1.02	6.781242	3.34	0.0046296
203572_s_at	6878	0.95	-5.77327	3.95	0.0046374
218146_at	55830	0.98	-7.311628	3.12	0.004644
220755_s_at	50854	0.97	-5.749961	3.97	0.0046449
202371_at	79921	1.05	11.42785	2.28	0.0046465
201839_s_at	4072	1.23	5.998856	3.78	0.0046538
34846_at	816	1.07	9.680019	2.52	0.004656
213527_s_at	146542	1.15	7.103319	3.19	0.0046781
200804_at	7009	1.03	7.088515	3.19	0.0046887
206507_at	9753	0.88	-8.535534	2.74	0.0047251
206102_at	9837	0.96	-5.91064	3.82	0.0047253
232242_at	5793	0.92	-5.687279	4.00	0.0047267
212541_at	80308	0.95	-5.707283	3.98	0.0047339
228536_at	90826	0.92	-12.09296	2.20	0.0047404
211043_s_at	1212	1.04	5.6914	3.99	0.0047608
207956_x_at	23047	0.96	-8.097624	2.85	0.0047612
217597_x_at	10966	1.06	12.03988	2.20	0.0047674
204835_at	5422	0.94	-6.101634	3.68	0.0047712
227980_at	493826	0.96	-8.260357	2.80	0.0047821
219351_at	6399	0.95	-7.01614	3.20	0.0047858
218138_at	8195	0.86	-8.437548	2.75	0.0047984
200027_at	4677	0.98	-5.664049	4.00	0.0048027
222578_s_at	79876	1.07	7.526072	3.01	0.0048056
201263_at	6897	0.96	-6.160771	3.63	0.0048101
217883_at	27249	0.98	-6.384345	3.50	0.0048167
227366_at	83547	1.16	7.509591	3.01	0.0048206
214086_s_at	10038	0.94	-6.077754	3.68	0.0048267
205406_s_at	53340	0.92	-5.793431	3.88	0.004838
45297_at	30846	0.90	-6.762456	3.30	0.0048485
209652_s_at	5228	1.15	8.685787	2.69	0.0048494
228023_x_at	55599	0.92	-5.641853	3.99	0.0048854
235275_at	656	1.23	5.678786	3.96	0.0048874
227267_at	134359	0.89	-8.020111	2.85	0.0048944
228122_at	285331	0.89	-12.26357	2.16	0.0049087
207304_at	7596	0.86	-7.425561	3.02	0.0049384

222585_x_at	51315	0.87	-5.832422	3.82	0.0049509
200693_at	57534	1.02	5.618522	3.99	0.0049513
224895_at	10413	0.96	-7.185908	3.10	0.0049652
205344_at	10675	1.14	10.51646	2.35	0.0050118
206491_s_at	8775	1.11	5.986788	3.70	0.005015
212835_at	23172	0.94	-5.673655	3.93	0.0050305
211999_at	3020	1.02	5.592973	3.99	0.0050484
204576_s_at	23059	0.93	-6.288046	3.50	0.0050569
229622_at	151176	1.08	5.859501	3.78	0.0050573
220588_at	55653	1.09	7.928208	2.84	0.0050587
212880_at	23335	0.88	-5.802265	3.81	0.0050835
224894_at	10413	0.97	-5.618224	3.96	0.0050864
202266_at	51567	0.98	-5.727633	3.87	0.0050917
221713_s_at	79929	1.12	9.337588	2.52	0.0051031
212190_at	5270	1.09	12.58134	2.11	0.005115
230404_at	401207	0.78	-5.794608	3.81	0.005134
206653_at	10622	1.23	7.052454	3.12	0.005158
213304_at	23116	1.08	6.30973	3.46	0.0051996
223274_at	6941	0.90	-6.549512	3.32	0.0052504
233873_x_at	55149	0.93	-6.276183	3.46	0.0052547
226902_at	8975	0.95	-5.625099	3.91	0.0052624
208861_s_at	546	0.96	-7.024985	3.11	0.0052653
219510_at	10721	0.92	-6.800947	3.20	0.005277
219968_at	51385	0.91	-11.58512	2.19	0.0052776
209950_s_at	50853	1.23	6.128456	3.54	0.0052911
241234_at	389025	0.83	-5.638729	3.89	0.0052963
212184_s_at	23118	0.97	-6.033286	3.60	0.0052984
201361_at	79073	0.96	-5.7647	3.79	0.0053016
225359_at	131118	0.94	-5.616193	3.90	0.0053136
223004_s_at	51300	0.94	-5.498386	4.00	0.0053353
214091_s_at	2878	1.15	9.155619	2.52	0.0053398
222601_at	55236	0.94	-5.497599	3.99	0.0053598
230105_at	10481	0.87	-6.952471	3.12	0.005376
219336_s_at	51008	0.91	-5.482624	3.99	0.0054138
223391_at	81537	1.05	5.494539	3.98	0.0054221
218433_at	79646	1.07	7.443103	2.93	0.005425
210652_s_at	22996	1.25	5.483385	3.99	0.0054306
203330_s_at	6811	1.05	8.273723	2.69	0.0054567
217834_s_at	10492	1.03	8.052619	2.75	0.0054636
219598_s_at	51389	0.97	-8.288082	2.69	0.00549
222155_s_at	79581	1.04	5.492473	3.96	0.0055034
218487_at	210	0.92	-5.701929	3.79	0.0055114
204302_s_at	9811	1.10	6.80773	3.15	0.005539
226730_s_at	57695	0.92	-6.616037	3.23	0.0055459
205006_s_at	9397	0.93	-6.142941	3.48	0.0055612
209681_at	10560	1.03	6.567946	3.25	0.005583
200974_at	59	0.75	-7.845346	2.79	0.0055898
212309_at	23122	0.96	-5.422729	4.00	0.0056108
226330_s_at	55578	0.89	-5.754478	3.72	0.0056191
225220_at	NA	0.90	-5.608675	3.83	0.0056288
201351_s_at	10730	1.01	5.414563	4.00	0.0056392
219926_at	64208	1.06	5.917806	3.60	0.0056418

224060_s_at	51611	0.96	-5.409162	4.00	0.0056652
219438_at	79570	1.38	8.516144	2.61	0.0056916
210455_at	27291	0.87	-5.427686	3.97	0.0057101
223095_at	83742	1.16	6.50144	3.26	0.0057151
204978_at	11129	0.94	-12.43171	2.06	0.0057176
58780_s_at	55701	0.90	-5.577252	3.84	0.0057219
225058_at	56927	0.95	-5.817551	3.65	0.0057236
224523_s_at	84319	0.96	-5.535158	3.87	0.0057251
202693_s_at	9263	1.08	7.126987	2.99	0.0057428
223035_s_at	10056	0.93	-5.495827	3.90	0.0057595
218263_s_at	58486	0.97	-5.535248	3.86	0.005763
218040_at	55119	0.96	-5.790336	3.67	0.0057646
204919_at	11272	0.85	-5.465521	3.92	0.0057795
218065_s_at	56674	1.01	5.393404	3.98	0.0057797
202541_at	9255	0.96	-5.57545	3.83	0.0057806
210868_s_at	79071	0.93	-5.886962	3.59	0.0057831
203166_at	10428	0.94	-5.376313	4.00	0.0057832
223068_at	27436	0.97	-5.396184	3.97	0.0058303
209818_s_at	22927	1.09	10.2527	2.29	0.0058321
210138_at	8601	1.11	5.976153	3.53	0.0058359
206650_at	55721	0.87	-5.478388	3.89	0.0058385
201735_s_at	1182	0.94	-7.439148	2.87	0.0058465
218511_s_at	55163	1.03	5.846144	3.61	0.0058658
201338_x_at	2971	0.97	-5.367651	3.99	0.0058782
225564_at	221178	1.09	6.398441	3.28	0.0058826
233064_at	388494	1.24	11.01746	2.19	0.005892
218710_at	55622	0.93	-6.308254	3.32	0.0058934
203093_s_at	10469	0.94	-5.551159	3.82	0.0058992
236487_at	132320	0.94	-5.362985	3.98	0.0059065
65630_at	283232	1.18	5.97712	3.51	0.0059083
201664_at	10051	0.98	-11.84459	2.09	0.0059366
223311_s_at	57504	0.90	-5.37733	3.96	0.0059549
209427_at	6525	0.91	-5.362559	3.97	0.005964
227341_at	222389	0.94	-5.322091	4.00	0.0059973
215091_s_at	2971	0.97	-5.814564	3.60	0.0060094
201739_at	6446	1.06	5.992936	3.48	0.0060171
244353_s_at	9519	0.88	-5.795866	3.61	0.0060197
227181_at	348801	0.90	-5.330503	3.99	0.0060259
202947_s_at	2995	1.14	6.270123	3.32	0.0060276
210658_s_at	23062	1.05	8.332121	2.60	0.0060429
221916_at	4747	1.05	8.795044	2.50	0.006052
212507_at	23505	1.06	5.74427	3.64	0.0060545
213012_at	4734	0.89	-5.549941	3.78	0.0060685
214414_x_at	3040	1.57	10.17271	2.27	0.0060795
209517_s_at	9070	0.97	-5.392997	3.91	0.006081
210323_at	27285	1.13	5.350694	3.95	0.0060894
202478_at	28951	0.87	-5.509018	3.81	0.0060997
236247_at	387338	0.93	-5.305542	3.99	0.0060999
222906_at	28982	1.06	5.421677	3.88	0.0061006
224860_at	90871	0.85	-5.624107	3.72	0.0061025
209433_s_at	5471	0.95	-5.372729	3.93	0.0061027
228360_at	130576	1.19	5.307239	3.99	0.0061032

224217_s_at	11124	0.95	-12.57692	2.01	0.0061269
209969_s_at	6772	0.97	-5.395103	3.90	0.0061275
200065_s_at	375	1.03	6.127464	3.38	0.0061371
227738_s_at	79798	1.10	5.901116	3.51	0.0061385
227875_at	90293	0.95	-5.290605	3.99	0.0061487
218957_s_at	80227	0.92	-5.554355	3.76	0.0061596
207467_x_at	831	1.04	6.598325	3.14	0.0061688
212010_s_at	55573	1.01	5.282132	4.00	0.006169
228495_at	253635	0.95	-5.572495	3.74	0.0061917
212037_at	5411	0.96	-6.987436	2.97	0.0062252
225737_s_at	26263	0.93	-6.21338	3.31	0.0062485
234979_at	144233	0.95	-6.192436	3.32	0.0062487
235463_s_at	253782	1.11	7.228929	2.88	0.0062659
218949_s_at	55278	0.91	-8.130096	2.62	0.0062825
225945_at	79027	0.96	-6.133217	3.35	0.0062928
225881_at	84912	0.94	-5.25663	3.99	0.0063028
208899_x_at	51382	1.05	7.622987	2.75	0.0063042
204332_s_at	175	0.89	-7.357061	2.83	0.006306
217716_s_at	29927	1.04	5.352264	3.90	0.0063307
225813_at	54542	0.96	-5.292871	3.95	0.0063413
203490_at	2000	1.05	5.916233	3.47	0.0063455
208453_s_at	7511	1.03	5.232448	4.00	0.006373
205077_s_at	5281	0.94	-5.757267	3.56	0.0063859
209806_at	85236	1.06	6.200268	3.29	0.0063903
217766_s_at	23585	1.02	5.972477	3.42	0.0064004
218998_at	54942	0.93	-5.584151	3.69	0.0064036
223792_at	7549	0.90	-9.349783	2.36	0.0064061
203683_s_at	7423	1.10	5.645193	3.64	0.0064072
235063_at	149840	0.86	-5.267631	3.95	0.0064222
207030_s_at	1466	0.99	-5.227598	3.99	0.0064239
226907_at	81706	1.05	5.375485	3.85	0.0064422
204421_s_at	2247	0.86	-7.002878	2.93	0.0064486
208971_at	7389	1.07	7.100175	2.90	0.0064519
219374_s_at	79796	0.93	-5.389246	3.84	0.0064528
201834_at	5564	0.92	-7.463977	2.78	0.0064551
204739_at	1060	0.93	-9.044648	2.41	0.0064622
206888_s_at	398	1.06	5.325932	3.89	0.0064641
212192_at	115207	1.07	6.158298	3.30	0.0064693
221951_at	283232	1.20	6.491643	3.14	0.0064735
229903_x_at	55599	0.87	-9.350826	2.35	0.006491
202468_s_at	8727	1.05	5.487162	3.74	0.0065078
229313_at	203859	1.06	6.161187	3.29	0.0065086
206756_at	56548	1.10	5.408004	3.81	0.0065169
205140_at	8790	0.95	-5.214374	3.98	0.0065299
202135_s_at	10120	0.93	-5.378838	3.83	0.0065492
207275_s_at	2180	1.11	8.257113	2.56	0.0065493
229194_at	84333	1.06	5.193216	4.00	0.0065504
209478_at	201254	0.97	-8.856543	2.43	0.006561
201095_at	1611	0.96	-5.195035	3.99	0.006563
213283_s_at	6297	0.92	-8.442649	2.51	0.0065899
211963_s_at	10092	0.98	-8.116031	2.58	0.00661
231844_at	157247	0.92	-7.020474	2.90	0.0066116

226422_at	51290	0.99	-7.205125	2.84	0.0066217
224444_s_at	84791	0.88	-5.318001	3.86	0.0066473
201850_at	822	1.16	5.695729	3.56	0.0066525
226459_at	118788	1.38	8.851338	2.42	0.0066652
217786_at	10419	0.98	-6.067677	3.32	0.0066713
222052_at	284325	0.93	-5.179901	3.99	0.0066739
204601_at	9683	0.91	-6.694443	3.02	0.0066772
218288_s_at	60492	0.94	-9.880852	2.26	0.0066789
201651_s_at	11252	1.03	5.588257	3.63	0.0066882
226196_s_at	112752	0.93	-7.593405	2.71	0.0066904
226116_at	NA	0.96	-5.854261	3.44	0.0066921
225531_at	91768	1.09	6.44675	3.12	0.0067018
213547_at	23066	1.08	5.180775	3.98	0.0067051
222065_s_at	2314	1.06	5.252974	3.91	0.0067054
209263_x_at	7106	1.13	6.792994	2.97	0.0067082
223220_s_at	83666	1.21	7.431899	2.75	0.0067129
219168_s_at	55615	1.10	5.364128	3.80	0.0067284
226876_at	359845	1.11	5.153999	4.00	0.0067341
208749_x_at	10211	1.06	6.626764	3.04	0.0067344
213126_at	112950	0.92	-5.801231	3.47	0.0067395
223130_s_at	29116	1.08	5.288864	3.86	0.0067705
202137_s_at	10771	1.10	7.28774	2.79	0.0067723
230186_at	219902	0.95	-8.321914	2.52	0.0067723
201835_s_at	5564	0.92	-6.008536	3.34	0.0067788
223272_s_at	84284	0.94	-6.150834	3.25	0.0068031
211566_x_at	9577	0.94	-5.217596	3.92	0.0068212
202811_at	10617	0.95	-5.285315	3.85	0.0068264
205074_at	6584	1.06	5.156303	3.97	0.0068332
202623_at	55837	0.96	-5.169914	3.96	0.0068529
210622_x_at	8558	0.92	-5.524401	3.64	0.006856
232054_at	64881	0.94	-6.43421	3.10	0.0068601
34726_at	784	1.05	5.146903	3.98	0.0068612
223398_at	84270	0.92	-5.733411	3.49	0.0068715
219317_at	11201	0.87	-6.079128	3.28	0.0068864
213312_at	57150	0.91	-7.289024	2.78	0.0068967
204618_s_at	2553	1.08	5.75049	3.48	0.0068974
238508_at	80174	0.89	-5.215777	3.90	0.0069155
220746_s_at	51720	0.95	-9.144758	2.35	0.0069243
229400_at	3236	1.08	5.110132	4.00	0.0069341
214330_at	91647	1.15	11.41332	2.05	0.0069347
239151_at	255326	0.89	-5.983523	3.32	0.0069378
224734_at	3146	1.09	6.018463	3.30	0.0069604
36566_at	1497	1.14	10.20827	2.18	0.006969
226998_at	80155	0.94	-6.248013	3.18	0.0069775
202745_at	9101	0.92	-5.370329	3.75	0.0069811
218385_at	55168	0.95	-6.371729	3.11	0.0069894
207076_s_at	445	1.13	5.120553	3.98	0.0069914
201656_at	3655	1.18	6.889075	2.90	0.006992
221505_at	81611	1.06	5.092874	4.00	0.0070211
219688_at	55212	0.88	-5.93673	3.34	0.0070254
228660_x_at	10505	0.90	-5.173552	3.91	0.0070374
221214_s_at	26012	1.05	5.094432	3.99	0.0070408

208492_at	5994	0.89	-5.731587	3.46	0.0070434
218007_s_at	51065	0.94	-5.67305	3.50	0.0070483
219952_s_at	57192	1.10	6.61393	3.00	0.0070663
221575_at	51540	0.92	-10.48645	2.14	0.0070665
235673_at	9014	0.96	-5.447349	3.67	0.0070684
227157_at	201973	0.89	-6.406609	3.08	0.0070768
204949_at	3385	1.45	9.931387	2.21	0.0070975
214435_x_at	5898	1.05	5.940554	3.32	0.0071128
225524_at	118429	1.09	5.091379	3.98	0.0071137
216064_s_at	175	0.86	-5.207721	3.86	0.0071442
218828_at	254863	1.15	6.043002	3.26	0.0071604
226781_at	154791	0.96	-6.408273	3.07	0.0071703
227628_at	493869	0.95	-5.131367	3.93	0.0071748
218018_at	8566	1.06	5.227379	3.83	0.0071768
225132_at	26224	0.96	-5.833329	3.37	0.0071943
224478_s_at	84310	0.92	-6.194637	3.17	0.0072162
234311_s_at	85865	0.90	-9.02996	2.33	0.0072942
32209_at	23625	1.04	7.803031	2.58	0.0073044
223602_at	84749	0.93	-6.064327	3.22	0.0073054
219641_at	55070	0.93	-5.146052	3.89	0.0073104
65521_at	51619	1.05	7.60367	2.63	0.0073141
219338_s_at	54839	0.89	-5.162176	3.87	0.0073202
216250_s_at	9404	0.71	-9.310827	2.28	0.0073263
226483_at	137695	0.96	-5.249551	3.78	0.0073337
225888_at	80018	0.93	-5.032533	4.00	0.0073393
214240_at	51083	1.13	8.425959	2.43	0.007382
203762_s_at	51626	0.92	-5.058657	3.95	0.0074476
203946_s_at	384	1.09	7.911007	2.54	0.0074497
203801_at	63931	0.87	-9.229017	2.28	0.0074666
201551_s_at	3916	1.08	5.859559	3.31	0.0074789
204585_s_at	3897	1.11	5.042288	3.95	0.0074963
217785_s_at	10652	1.08	6.026438	3.21	0.0075202
65493_at	63897	0.92	-5.036628	3.95	0.007521
218663_at	64151	0.95	-7.336889	2.69	0.0075322
219356_s_at	51510	1.05	11.24132	2.02	0.0075913
212048_s_at	8565	0.96	-5.307807	3.69	0.0075936
222774_s_at	81831	1.07	5.349605	3.65	0.0076053
216305_s_at	6936	0.94	-5.753197	3.36	0.0076088
227656_at	55780	0.92	-6.27897	3.07	0.0076509
211500_at	5600	1.11	6.386584	3.02	0.0076528
221580_s_at	79101	0.94	-6.372794	3.02	0.0076538
200870_at	11171	0.98	-5.326845	3.66	0.0076634
217099_s_at	50628	0.98	-4.965171	4.00	0.0076935
225604_s_at	152007	1.25	5.260119	3.70	0.0077151
226253_at	201255	1.11	6.203264	3.09	0.0077195
220840_s_at	55732	0.85	-6.712886	2.87	0.0077483
54632_at	63892	0.91	-10.44025	2.09	0.0077643
226582_at	400043	0.94	-5.014371	3.93	0.007765
222589_at	51701	0.95	-5.051654	3.89	0.0077664
238590_x_at	84314	0.91	-5.971604	3.20	0.0077838
223057_s_at	57510	0.95	-5.474467	3.52	0.0077838
239760_at	1353	0.90	-6.549178	2.93	0.0077881

235737_at	85480	0.82	-6.53359	2.93	0.0077974
203718_at	10908	1.15	5.672702	3.38	0.007803
238452_at	127943	1.09	5.718002	3.35	0.0078059
202823_at	6921	1.08	6.00687	3.18	0.0078128
201501_s_at	2926	1.02	5.413353	3.56	0.0078385
218681_s_at	23753	1.07	5.231876	3.70	0.0079075
226981_at	143941	0.94	-4.943756	3.98	0.0079125
236402_at	673	0.88	-5.866963	3.24	0.0079298
229491_at	133308	1.13	6.585665	2.90	0.0079299
205240_at	29899	0.95	-6.599292	2.89	0.0079363
219862_s_at	26502	1.09	5.268674	3.66	0.0079427
64440_at	84818	1.05	4.973781	3.93	0.0079716
223219_s_at	25904	0.94	-5.520137	3.46	0.0079826
219838_at	64927	0.89	-5.308312	3.62	0.0079941
231319_x_at	64147	1.15	8.898654	2.29	0.008016
33322_i_at	2810	1.04	5.05034	3.84	0.0080344
218581_at	63874	1.02	4.916324	3.98	0.0080489
227334_at	159195	0.92	-4.902022	4.00	0.0080535
203306_s_at	10559	0.93	-6.976008	2.74	0.0080565
227284_at	90321	0.88	-5.654228	3.35	0.0080698
201616_s_at	800	1.13	4.92975	3.96	0.0080925
212607_at	10000	0.94	-8.080573	2.44	0.0080944
203690_at	10426	0.96	-4.920713	3.96	0.0081202
239824_s_at	84314	0.88	-6.574399	2.88	0.0081313
238711_s_at	7707	0.89	-5.041841	3.83	0.0081495
228253_at	84695	1.10	5.889406	3.20	0.0081576
231896_s_at	8562	0.97	-5.478139	3.45	0.0081909
226684_at	55102	0.96	-4.932276	3.94	0.0081947
205571_at	51601	0.92	-5.775464	3.26	0.0081974
214672_at	23093	0.92	-5.259931	3.62	0.0082052
201067_at	5701	0.96	-6.031268	3.11	0.0082086
227442_at	285521	0.91	-5.20584	3.66	0.0082242
208970_s_at	7389	1.06	7.418852	2.59	0.0082354
208190_s_at	51599	1.09	7.235231	2.64	0.0082543
218959_at	3226	1.07	5.586529	3.37	0.0082627
216996_s_at	22868	0.96	-5.703795	3.29	0.0082692
31845_at	2000	1.04	5.023427	3.83	0.0082781
222646_s_at	30001	1.03	5.751083	3.26	0.0082801
225976_at	91408	0.95	-5.357134	3.53	0.0082827
218673_s_at	10533	0.94	-5.012348	3.83	0.0082891
202539_s_at	3156	1.03	4.9001	3.95	0.0082961
224347_x_at	118424	0.95	-5.831573	3.21	0.0083086
210378_s_at	8636	1.03	6.777204	2.78	0.0083217
203845_at	8850	1.05	6.926726	2.73	0.0083299
212958_x_at	5066	1.06	10.52269	2.04	0.0083347
201162_at	3490	1.21	6.412177	2.92	0.0083483
235498_at	127255	0.81	-4.939695	3.90	0.0083505
219426_at	192669	0.95	-5.133961	3.71	0.0083533
224492_s_at	199692	0.91	-4.911706	3.93	0.0083558
205550_s_at	9577	0.94	-6.801165	2.77	0.0083605
202934_at	3099	1.06	5.174874	3.67	0.0083648
219635_at	80095	0.85	-4.981569	3.85	0.0083653

222742_s_at	64792	0.96	-7.678678	2.51	0.0083691
226886_at	2673	0.94	-7.520678	2.55	0.0083772
204034_at	23474	1.09	5.961845	3.12	0.0084053
201189_s_at	3710	1.09	4.844999	3.99	0.0084073
204282_s_at	10667	0.90	-5.676496	3.29	0.0084116
226707_at	93100	1.11	5.472534	3.41	0.0084801
204018_x_at	3040	1.32	6.885767	2.73	0.0084863
227668_at	146705	1.06	6.713001	2.78	0.0085025
222939_s_at	117247	1.09	8.898412	2.25	0.0085114
212833_at	91137	0.95	-5.693199	3.26	0.0085145
213685_at	6924	0.90	-5.01795	3.79	0.0085235
213913_s_at	23329	1.29	9.395046	2.17	0.0085241
218231_at	55577	1.07	5.258314	3.57	0.0085272
218440_at	56922	1.06	4.894524	3.92	0.0085281
219767_s_at	9946	0.92	-4.820344	4.00	0.0085297
226191_at	2932	0.94	-8.389932	2.34	0.0085369
203376_at	51362	0.97	-4.942744	3.86	0.0085478
204729_s_at	6804	1.09	5.400972	3.45	0.0085655
228326_at	253769	0.88	-6.524417	2.85	0.008574
217880_at	996	0.96	-6.371721	2.91	0.0085925
223114_at	84274	0.97	-6.010959	3.07	0.0086147
212721_at	140890	0.91	-7.496501	2.53	0.0086193
202255_s_at	26037	0.91	-4.814229	3.98	0.0086466
202277_at	10558	1.02	5.038356	3.75	0.0086496
205768_s_at	11001	1.23	6.137946	3.01	0.0086526
202824_s_at	6921	1.03	4.816915	3.98	0.0086721
209449_at	57819	0.96	-4.921114	3.86	0.0086768
227611_at	123283	1.09	4.865694	3.92	0.0086991
243299_at	7444	0.90	-6.003933	3.06	0.0087168
225525_at	85379	1.11	4.814515	3.97	0.008725
220027_s_at	54922	1.09	5.967566	3.08	0.0087429
216272_x_at	85360	0.96	-4.843643	3.93	0.008754
225958_at	1911	0.91	-6.217829	2.95	0.0087779
219553_at	29922	0.94	-4.96335	3.79	0.0088451
229710_at	NA	0.95	-4.767622	4.00	0.0088555
223618_at	56776	1.08	6.538471	2.81	0.0088556
221743_at	10658	0.98	-5.543599	3.31	0.0088576
218066_at	10723	1.07	4.852611	3.90	0.0088751
227545_at	580	0.89	-5.251505	3.52	0.0088792
209264_s_at	7106	1.21	10.10188	2.05	0.0089231
218987_at	55729	0.95	-6.121565	2.98	0.0089328
228239_at	54065	0.90	-4.909362	3.83	0.0089479
226758_at	51631	0.96	-9.109978	2.18	0.008968
231233_at	NA	0.93	-4.756974	3.99	0.0089744
225698_at	114915	0.98	-5.159739	3.58	0.008996
204812_at	9183	0.94	-5.678512	3.21	0.009004
227141_at	127253	0.97	-5.00187	3.72	0.0090086
202365_at	84747	0.95	-4.833844	3.89	0.0090481
218008_at	55069	1.03	4.912116	3.80	0.0090656
336_at	6915	0.93	-4.811485	3.91	0.0090784
226468_at	27246	0.95	-4.75383	3.97	0.0090866
47069_at	55615	1.08	4.980942	3.73	0.0090895

225475_at	57708	0.96	-5.147572	3.58	0.0090951
200661_at	5476	1.08	4.730193	4.00	0.009103
219073_s_at	114884	1.17	8.719049	2.23	0.0091195
214141_x_at	6432	0.98	-4.724978	4.00	0.0091394
203565_s_at	4331	0.95	-5.82673	3.11	0.0091431
218886_at	55003	0.91	-4.960937	3.74	0.0091466
213239_at	10464	0.90	-10.36509	2.00	0.0091546
205202_at	5110	1.03	4.95942	3.74	0.0091602
201175_at	51075	0.97	-5.395162	3.37	0.0091701
224945_at	55727	0.96	-4.748974	3.96	0.0091856
227240_at	25791	1.10	9.996092	2.04	0.0092116
206837_at	8092	0.95	-5.247499	3.48	0.0092174
208985_s_at	8669	1.04	6.004065	3.00	0.0092386
235282_at	NA	0.91	-4.769263	3.93	0.0092652
226868_at	283464	0.95	-5.206912	3.50	0.0092657
220408_x_at	55578	0.90	-5.712642	3.15	0.0092787
211033_s_at	5191	0.92	-4.983953	3.70	0.0092865
229433_at	64062	0.93	-5.300506	3.43	0.0092926
200896_x_at	3068	0.97	-4.973489	3.70	0.0092992
219405_at	55128	0.91	-4.756219	3.93	0.009309
217993_s_at	27430	0.99	-5.219307	3.49	0.0093116
209549_s_at	1716	0.96	-5.177515	3.52	0.0093348
223606_x_at	55425	1.04	5.700773	3.15	0.009339
225313_at	63939	0.95	-5.406661	3.34	0.0093448
201427_s_at	6414	0.92	-9.455616	2.10	0.0093562
203211_s_at	8898	0.97	-5.495834	3.27	0.0093809
210243_s_at	8703	1.03	5.702067	3.15	0.0093862
222610_s_at	64766	0.96	-6.136305	2.92	0.0094288
229794_at	199704	0.85	-4.884418	3.77	0.0094386
209902_at	545	0.84	-5.60938	3.19	0.0094724
217080_s_at	9455	1.09	5.372169	3.35	0.0094863
219419_at	79863	0.95	-6.214253	2.88	0.009488
209833_at	8738	0.92	-5.034359	3.61	0.0095101
244834_at	79363	0.87	-4.868741	3.78	0.0095105
227348_at	25973	0.92	-4.68483	3.98	0.0095107
210249_s_at	8648	0.93	-4.680919	3.99	0.0095121
225481_at	122786	1.05	5.639226	3.17	0.0095175
212281_s_at	27346	1.03	5.875378	3.04	0.0095212
205622_at	6610	1.07	5.283722	3.41	0.0095222
203668_at	4123	0.91	-7.664164	2.41	0.0095293
204510_at	8317	0.95	-4.66752	4.00	0.009541
222523_at	59343	0.95	-5.092956	3.56	0.0095502
227667_at	404093	1.13	8.852636	2.18	0.009562
227686_at	92106	0.94	-4.719028	3.93	0.0095667
228398_at	123228	0.81	-4.722184	3.93	0.009569
226194_at	283489	0.95	-5.165559	3.49	0.0096048
222918_at	51209	1.15	6.273121	2.84	0.0096177
229090_at	220930	0.79	-4.660557	3.99	0.0096236
216100_s_at	26092	1.14	6.080246	2.92	0.0096451
225534_at	114926	0.93	-5.591869	3.18	0.0096782
212615_at	80205	0.94	-4.649531	3.99	0.0096995
64432_at	51275	0.91	-6.65193	2.69	0.0097004

202579_x_at	10473	0.97	-7.358862	2.47	0.0097239
238497_at	219902	0.93	-8.314255	2.26	0.0097317
201136_at	5355	1.13	4.65455	3.97	0.0097749
214264_s_at	90141	0.92	-6.749867	2.64	0.0097782
203578_s_at	9057	1.08	5.41065	3.28	0.009785
203847_s_at	10270	1.05	5.079482	3.53	0.00979
226274_at	158563	0.94	-5.261591	3.39	0.009791
224987_at	221477	0.91	-5.368982	3.31	0.0098006
226913_s_at	30812	1.14	5.753301	3.07	0.0098063
242338_at	169200	1.05	4.804682	3.79	0.0098635
222192_s_at	60526	0.92	-5.628621	3.13	0.0098696
218888_s_at	81831	1.06	5.026249	3.57	0.0098924
214274_s_at	30	1.10	4.661341	3.95	0.009893
209343_at	80303	1.05	8.504326	2.21	0.0099073
226086_at	57586	1.29	5.556625	3.17	0.0099252
225432_s_at	57325	0.88	-5.029013	3.55	0.0099798
226163_at	221504	0.89	-4.606644	4.00	0.0099809
219911_s_at	28231	1.25	8.229049	2.26	0.0099993
217984_at	8635	1.07	8.343035	2.23	0.0100051
201725_at	8872	0.96	-5.695819	3.08	0.0100082
210124_x_at	10505	0.92	-4.723251	3.85	0.0100138
220605_s_at	22933	1.05	4.679347	3.90	0.0100252
229269_x_at	170463	1.05	4.969292	3.60	0.0100483
205521_at	9941	0.91	-5.869556	2.98	0.0100586
221270_s_at	81890	1.09	5.896774	2.96	0.0100661
38691_s_at	6440	1.10	6.506166	2.70	0.0100882
235134_at	NA	0.82	-7.430632	2.42	0.0100983
212083_at	113419	0.96	-4.67458	3.89	0.0101138
240037_at	150771	1.08	4.595867	3.99	0.0101505
226093_at	196513	0.93	-9.649206	2.02	0.0101713
227940_at	339803	0.89	-7.012912	2.53	0.0101745
205087_at	25950	0.95	-6.708266	2.62	0.0101811
203150_at	10244	0.96	-6.243138	2.79	0.0101972
218024_at	51660	1.05	5.898173	2.95	0.0102074
218344_s_at	55758	0.93	-4.577887	4.00	0.0102112
212682_s_at	91289	1.04	4.8036	3.74	0.0102135
202167_s_at	64210	0.93	-4.607044	3.96	0.0102456
206397_x_at	10715	1.13	5.71331	3.04	0.0102459
218035_s_at	54502	1.12	9.176668	2.08	0.0102741
224518_s_at	84527	0.93	-5.473451	3.18	0.0102826
224048_at	84101	0.90	-5.084568	3.46	0.0103062
213069_at	57493	1.08	7.405388	2.41	0.0103081
58900_at	222070	1.02	5.177492	3.38	0.0103117
218418_s_at	25959	0.96	-8.287496	2.22	0.0103191
204348_s_at	387851	1.09	5.861567	2.96	0.0103227
221652_s_at	55726	0.96	-5.711266	3.03	0.010342
217403_s_at	7770	0.87	-5.079222	3.46	0.0103505
227784_s_at	9382	0.94	-5.111065	3.43	0.0103578
226657_at	256306	1.15	4.564367	3.99	0.0103593
225257_at	90324	0.92	-5.038982	3.49	0.0103646
225394_s_at	85437	0.95	-4.586267	3.96	0.0103727
221847_at	NA	0.96	-5.700569	3.03	0.0103728

200935_at	811	1.12	4.661825	3.87	0.0103758
230345_at	NA	1.12	4.769926	3.75	0.0103845
208638_at	10130	1.02	6.216944	2.79	0.010396
221801_x_at	4747	1.07	5.794041	2.98	0.0104197
202330_s_at	7374	1.03	5.124464	3.41	0.010432
207877_s_at	4931	0.96	-4.884299	3.62	0.0104554
213427_at	10799	0.97	-4.64813	3.87	0.0104605
206302_s_at	440672	1.06	5.53597	3.12	0.0104622
219518_s_at	80237	1.28	8.745332	2.13	0.0104831
220553_s_at	55015	0.93	-4.835448	3.66	0.010489
216356_x_at	8938	1.12	4.599103	3.93	0.0104912
210788_s_at	51635	0.97	-4.594653	3.93	0.01051
213478_at	23254	1.07	4.919074	3.58	0.0105119
218512_at	55759	0.98	-4.538024	4.00	0.0105129
201532_at	5684	0.98	-4.55835	3.97	0.0105148
219922_s_at	4054	1.11	7.426496	2.39	0.0105208
218861_at	64320	0.93	-5.021667	3.48	0.0105228
203811_s_at	11080	1.05	6.50778	2.66	0.0105367
227626_at	85315	1.08	4.534496	4.00	0.010546
204364_s_at	65055	1.14	5.196457	3.34	0.010558
217678_at	23657	0.92	-4.649447	3.85	0.0105813
228051_at	202451	1.18	5.608134	3.06	0.010603
219833_s_at	114327	0.87	-5.576685	3.08	0.010608
227607_at	57559	0.88	-5.260961	3.28	0.0106133
214934_at	374868	0.86	-5.022356	3.47	0.0106258
225834_at	389835	0.97	-5.943185	2.88	0.0106339
212720_at	10914	1.05	4.530213	3.99	0.0106448
238854_at	NA	0.81	-4.931547	3.55	0.0106449
221547_at	8559	1.07	6.861839	2.53	0.0106475
204227_s_at	7084	0.88	-4.842815	3.63	0.0106645
224887_at	84572	1.13	4.829331	3.64	0.010667
219265_at	79817	1.12	6.577509	2.62	0.0106728
35626_at	6448	0.95	-5.135626	3.37	0.0106916
209598_at	10687	0.93	-4.525225	3.99	0.0106992
229568_at	79817	1.10	5.072203	3.42	0.0107054
226588_at	57703	0.95	-6.206559	2.76	0.0107172
224713_at	84365	0.97	-5.513675	3.10	0.0107284
222609_s_at	51013	0.97	-4.667965	3.81	0.0107405
225043_at	121260	0.96	-5.363981	3.20	0.0107449
232296_s_at	85476	0.96	-5.011381	3.46	0.0107466
203135_at	6908	0.95	-6.292439	2.72	0.0107691
209903_s_at	545	0.89	-8.547815	2.15	0.0107724
217742_s_at	51322	0.96	-4.970737	3.49	0.0107904
228348_at	55180	0.84	-8.439853	2.16	0.0107953
236321_at	285550	0.85	-4.521569	3.98	0.0107956
213558_at	27445	0.89	-4.662905	3.80	0.0108004
235113_at	122769	0.96	-5.522722	3.09	0.0108029
218656_s_at	10186	0.94	-4.553378	3.93	0.0108052
221045_s_at	8863	1.26	5.518707	3.09	0.0108205
225796_at	54899	1.06	6.660344	2.58	0.0108229
219242_at	80254	0.95	-5.497751	3.10	0.0108233
225570_at	254428	1.05	4.683785	3.77	0.0108288

211208_s_at	8573	0.97	-4.575924	3.90	0.010832
215111_s_at	8848	0.92	-5.298082	3.23	0.0108398
218003_s_at	2287	0.97	-5.238181	3.27	0.0108445
217759_at	54765	0.96	-4.610209	3.86	0.0108491
212473_s_at	9645	1.18	8.29062	2.18	0.0108759
208869_s_at	23710	1.08	5.359182	3.18	0.0108927
228355_s_at	91942	0.94	-4.500279	3.99	0.0109018
227769_at	2850	1.03	4.522822	3.96	0.0109138
234295_at	51163	0.96	-4.569257	3.90	0.0109249
230146_s_at	23413	1.07	4.692302	3.75	0.0109251
219492_at	26511	1.02	4.674219	3.77	0.0109283
222451_s_at	51114	1.08	4.932942	3.51	0.0109399
209029_at	50813	0.96	-5.146218	3.33	0.0109481
242138_at	1745	1.11	7.750681	2.29	0.0109547
217967_s_at	116496	0.86	-7.182654	2.42	0.0109791
203860_at	5095	0.94	-5.986835	2.83	0.0109839
235964_x_at	140711	0.97	-4.960206	3.48	0.0109844
51774_s_at	222070	1.02	4.912429	3.52	0.0109865
229444_at	64771	1.08	5.059678	3.39	0.0109942
204752_x_at	10038	0.95	-6.120312	2.77	0.011
234710_s_at	56965	0.94	-4.827585	3.60	0.0110035
218693_at	23555	1.05	5.502575	3.08	0.0110065
219204_s_at	63826	0.91	-4.490279	3.98	0.0110125
222574_s_at	79665	0.98	-5.511121	3.07	0.0110329
211094_s_at	4763	0.91	-5.475247	3.09	0.0110369
201697_s_at	1786	0.96	-8.781046	2.09	0.011037
226114_at	80818	0.92	-4.475431	4.00	0.0110453
221277_s_at	83480	0.94	-4.588156	3.85	0.0110541
203665_at	3162	1.09	4.49435	3.97	0.0110556
229878_at	85459	0.92	-5.747901	2.94	0.0110592
222388_s_at	55737	1.03	4.901879	3.52	0.0110671
216484_x_at	3068	0.96	-4.684895	3.74	0.0110832
208645_s_at	6208	1.01	8.345811	2.16	0.0110925
220212_s_at	63892	0.90	-5.580985	3.03	0.011096
238912_x_at	138241	0.96	-5.343775	3.17	0.011104
205775_at	26240	0.95	-6.291049	2.69	0.0111161
227325_at	255783	1.06	4.621689	3.80	0.0111225
244532_x_at	401131	0.97	-6.586975	2.58	0.011152
203562_at	9638	1.13	8.192104	2.18	0.0112508
32137_at	3714	1.11	4.824409	3.57	0.0112514
213058_at	23331	0.95	-4.449253	4.00	0.0112694
212506_at	8301	0.97	-4.58721	3.82	0.0112735
230421_at	345462	0.85	-6.312686	2.67	0.0113064
223741_s_at	94015	1.07	7.589109	2.29	0.0113381
213031_s_at	84942	0.96	-6.474603	2.61	0.0113399
225940_at	317649	1.07	4.743483	3.64	0.0113605
202423_at	7994	0.97	-4.489327	3.93	0.0113788
204086_at	23532	0.96	-4.467364	3.96	0.0113792
203232_s_at	6310	0.95	-6.380423	2.64	0.0113871
204266_s_at	1119	1.07	5.302353	3.17	0.0113883
212186_at	31	0.93	-5.239541	3.21	0.0113974
226356_at	84895	0.90	-4.945213	3.44	0.0113988

218714_at	78994	0.95	-5.308643	3.16	0.0114274
203098_at	9425	0.94	-4.437067	3.99	0.0114284
229202_at	80003	0.94	-5.902112	2.83	0.011463
238059_at	NA	0.87	-4.491496	3.91	0.0114741
219124_at	80185	0.88	-4.425832	4.00	0.011481
210059_s_at	5603	1.13	5.853469	2.84	0.0115157
225019_at	817	1.04	7.80321	2.23	0.0115864
226033_at	57478	0.96	-4.489507	3.90	0.011594
203894_at	27175	0.90	-8.570301	2.09	0.0115966
203152_at	64976	0.95	-4.428964	3.97	0.0115988
234304_s_at	51194	1.05	4.805702	3.54	0.0116028
222154_s_at	26010	1.04	5.403108	3.08	0.0116074
205231_s_at	7957	1.24	7.615043	2.27	0.0116102
222530_s_at	8195	0.94	-4.440137	3.96	0.0116253
231869_at	57691	0.95	-5.035872	3.34	0.0116277
203550_s_at	10712	0.96	-4.409844	4.00	0.0116334
200945_s_at	22872	0.98	-4.498745	3.88	0.011639
217854_s_at	5434	1.03	6.420618	2.60	0.01164
227963_at	388341	1.12	4.417599	3.98	0.0116554
226452_at	5163	1.04	7.753384	2.24	0.0116821
225088_at	123811	0.93	-7.346633	2.33	0.0116956
203436_at	10556	0.95	-4.448395	3.93	0.0117019
205047_s_at	440	0.96	-5.211445	3.20	0.0117174
212441_at	9778	0.94	-4.427717	3.96	0.0117256
213222_at	23236	0.95	-4.938504	3.41	0.0117262
208737_at	9550	1.02	4.581331	3.76	0.0117457
221484_at	9334	1.06	4.697073	3.63	0.0117502
210202_s_at	274	1.12	4.404871	3.98	0.0117558
227624_at	57667	0.92	-6.221054	2.66	0.0117618
1555393_s_at	84537	0.87	-7.500901	2.29	0.0117856
219368_at	4674	0.91	-5.327412	3.11	0.0117909
206092_x_at	51750	1.07	4.473808	3.88	0.0118071
211061_s_at	4247	1.04	4.493922	3.85	0.011835
213243_at	157680	0.93	-6.597015	2.52	0.0118595
218622_at	79023	0.97	-4.41778	3.95	0.01186
201423_s_at	8451	1.07	4.720927	3.59	0.0118626
203984_s_at	842	0.93	-4.612504	3.71	0.0118662
226792_s_at	90990	1.09	4.507206	3.83	0.011895
204867_at	2644	1.11	4.997659	3.34	0.0119082
224303_x_at	51199	1.08	5.075789	3.27	0.0119173
207022_s_at	3948	0.86	-4.406102	3.96	0.0119266
212446_s_at	253782	1.07	4.72534	3.58	0.0119482
234660_s_at	22894	0.97	-4.728091	3.58	0.0119489
224952_at	26115	0.91	-5.263126	3.13	0.0119581
229097_at	81624	0.95	-5.437813	3.02	0.0119588
225974_at	169200	1.08	4.576152	3.74	0.0119686
226892_at	26148	0.94	-7.066164	2.38	0.0119745
201962_s_at	10193	0.91	-4.404512	3.95	0.0119977
212987_at	26268	0.97	-4.363322	4.00	0.0120308
224671_at	124995	0.97	-4.360602	4.00	0.0120605
208864_s_at	7295	1.02	5.443773	3.01	0.012067
229986_at	377064	0.91	-4.38357	3.96	0.0121095

227508_at	NA	0.87	-4.362581	3.99	0.0121198
38290_at	10636	1.09	4.480256	3.83	0.0121462
209568_s_at	23179	1.07	4.37471	3.96	0.0121661
227359_at	127700	0.90	-4.478433	3.82	0.0121975
227990_at	10569	1.06	5.529893	2.95	0.0122043
213340_s_at	57212	0.97	-5.111661	3.21	0.0122199
205770_at	2936	1.10	4.341977	4.00	0.0122509
218244_at	55035	0.95	-5.797644	2.81	0.0122527
204767_s_at	2237	0.98	-4.341998	4.00	0.0122534
226946_at	133686	1.06	7.48322	2.26	0.0122566
233764_s_at	55132	1.15	4.542264	3.74	0.0122615
206670_s_at	2571	0.89	-5.287807	3.09	0.0122732
203092_at	10469	0.92	-5.122973	3.20	0.0122939
225946_at	11228	0.92	-5.102901	3.21	0.0123089
218041_x_at	54407	0.99	-4.896596	3.37	0.0123142
202931_x_at	274	1.10	4.924305	3.34	0.0123749
226327_at	22847	0.96	-4.446263	3.83	0.0124103
225342_at	387851	1.11	7.040676	2.36	0.0124239
212115_at	90861	0.97	-5.068038	3.22	0.0124456
218122_s_at	59343	0.97	-4.365033	3.93	0.0124679
208716_s_at	54499	1.02	4.333067	3.97	0.0125047
218243_at	80230	0.96	-4.326876	3.98	0.0125114
235513_at	NA	0.90	-4.419612	3.85	0.0125278
225614_at	113174	0.97	-4.956026	3.30	0.0125291
225941_at	317649	1.08	4.673921	3.56	0.0125495
224641_at	84248	1.02	4.666335	3.56	0.0125694
238496_at	54904	0.96	-5.27949	3.06	0.0125816
214439_x_at	274	1.14	6.910252	2.38	0.0125855
211922_s_at	847	0.94	-4.374962	3.90	0.0126034
205938_at	22843	0.84	-6.887294	2.38	0.0126069
225526_at	4289	0.95	-4.839217	3.39	0.0126284
218593_at	55131	0.96	-4.350405	3.93	0.0126431
214818_at	284001	0.93	-4.321538	3.97	0.0126566
210062_s_at	51385	0.84	-6.308053	2.56	0.0126858
224097_s_at	50848	1.10	6.010391	2.67	0.0127029
215499_at	5606	1.04	4.310488	3.97	0.0127181
223451_s_at	51192	0.93	-7.487977	2.23	0.0127353
218970_s_at	51076	0.93	-4.529603	3.69	0.0127519
223542_at	84250	0.91	-5.015736	3.23	0.0127625
223322_at	83593	0.94	-4.345896	3.92	0.0127627
207843_x_at	1528	0.98	-5.345433	3.00	0.0127735
208979_at	23054	0.95	-5.553927	2.88	0.0127749
212333_at	25940	0.96	-5.373485	2.99	0.0127801
218168_s_at	56997	1.04	4.296928	3.98	0.0127808
218085_at	51510	1.06	6.451132	2.51	0.012787
205260_s_at	97	0.94	-5.321954	3.02	0.0127898
223277_at	54859	0.96	-5.144776	3.13	0.0127903
213708_s_at	6945	0.97	-4.323194	3.94	0.0127988
205345_at	580	0.91	-5.618974	2.85	0.0128178
223062_s_at	29968	0.98	-5.55502	2.88	0.0128186
213772_s_at	23062	1.04	4.941374	3.28	0.0128538
213361_at	23424	0.91	-7.155672	2.30	0.0128739

231772_x_at	64946	0.94	-4.548991	3.65	0.0128758
226118_at	79172	0.92	-6.008494	2.66	0.0129013
212069_s_at	84726	0.96	-4.509014	3.69	0.0129037
211959_at	3488	0.94	-4.293002	3.97	0.0129209
206289_at	3201	0.94	-5.400261	2.95	0.0129433
204703_at	8100	0.88	-6.203585	2.58	0.0129473
228010_at	5522	1.17	4.275263	3.99	0.0129592
224614_at	1783	1.09	5.153882	3.11	0.0129902
239406_at	7746	0.85	-5.42216	2.94	0.0130153
203048_s_at	9652	0.93	-4.481397	3.71	0.013021
219161_s_at	51192	0.93	-5.409534	2.94	0.013024
227920_at	57673	1.10	4.32262	3.91	0.013029
225014_at	389203	0.91	-4.267953	3.99	0.0130313
209572_s_at	8726	0.96	-6.263784	2.55	0.0130546
226267_at	122953	0.91	-6.294124	2.54	0.0130648
226449_at	153241	0.97	-5.199669	3.06	0.0131162
215148_s_at	9546	0.87	-5.917534	2.68	0.0131162
227015_at	57168	0.89	-4.751966	3.41	0.0131207
225060_at	84918	1.07	6.481563	2.47	0.0131212
226855_at	NA	0.91	-4.295387	3.94	0.0131258
204858_s_at	1890	1.17	4.266007	3.98	0.0131291
203513_at	80208	0.96	-5.226945	3.04	0.0131453
213804_at	3633	0.89	-8.527294	2.02	0.013168
211084_x_at	23683	1.04	4.567207	3.59	0.0131755
229423_at	1111	0.79	-5.251696	3.02	0.0131778
212237_at	171023	0.96	-5.412843	2.93	0.0131849
226580_at	84312	0.96	-6.665101	2.41	0.0132156
223334_at	84233	0.94	-5.199119	3.05	0.0132217
223295_s_at	55692	0.94	-4.355602	3.84	0.0132273
220323_at	79935	1.08	4.501614	3.66	0.013249
212025_s_at	2314	1.06	5.168039	3.07	0.0132526
217364_x_at	8669	1.08	6.039666	2.62	0.0132607
227416_s_at	85437	0.94	-5.87522	2.69	0.0132804
206669_at	2571	0.90	-4.653915	3.49	0.0133002
203511_s_at	27095	0.97	-4.24956	3.98	0.0133018
225447_at	2820	1.08	4.401136	3.77	0.0133103
228859_at	91431	0.91	-4.265176	3.95	0.0133564
219203_at	51016	0.92	-4.945293	3.22	0.0133633
203442_x_at	256364	1.07	4.351788	3.82	0.0134055
202584_at	4799	0.93	-4.275139	3.92	0.0134333
203962_s_at	10529	1.11	4.739775	3.39	0.0134464
225509_at	56757	0.87	-5.133812	3.08	0.0134506
219403_s_at	10855	1.08	4.966651	3.20	0.013463
223546_x_at	55692	0.92	-5.74855	2.73	0.0134633
202182_at	2648	0.95	-5.798476	2.71	0.0134666
218973_at	79631	0.95	-5.552602	2.83	0.0134823
210132_at	1944	1.10	5.711673	2.75	0.0134847
222496_s_at	54502	1.16	4.331499	3.83	0.0135224
226546_at	NA	0.93	-4.516742	3.61	0.013531
201575_at	22938	0.98	-5.274462	2.98	0.0135411
227037_at	201164	1.05	4.728944	3.39	0.0135709
230078_at	96459	0.95	-4.437156	3.69	0.0135727

228200_at	286101	0.85	-4.664826	3.45	0.0135738
220865_s_at	23590	0.98	-4.42174	3.71	0.0135791
218542_at	55165	1.05	4.809895	3.31	0.0135845
218157_x_at	56882	0.96	-4.254171	3.93	0.0135885
230454_at	130026	1.08	8.278894	2.03	0.0136149
217975_at	51186	0.98	-5.036099	3.13	0.013622
223493_at	26272	0.91	-4.384436	3.75	0.01365
220370_s_at	57602	1.16	4.369275	3.77	0.0136585
221697_at	440738	1.13	4.550397	3.55	0.0136622
212334_at	2799	1.05	4.847478	3.27	0.013663
206247_at	4277	1.05	4.312003	3.84	0.0136661
201433_s_at	9791	1.02	4.7671	3.34	0.013672
227983_at	196383	1.02	4.213239	3.98	0.0136836
213807_x_at	4233	1.07	4.270855	3.89	0.0137222
205700_at	8630	1.12	4.196223	4.00	0.0137382
215905_s_at	9410	0.97	-5.583398	2.79	0.0137511
206552_s_at	6863	1.15	4.954091	3.18	0.0137548
225028_at	550643	0.94	-4.210542	3.98	0.0137592
210681_s_at	9958	0.97	-4.326627	3.81	0.0137747
206070_s_at	2042	0.91	-7.188561	2.24	0.0137964
212616_at	80205	0.95	-4.405267	3.71	0.0137976
225492_at	NA	0.96	-5.154962	3.03	0.0137976
215706_x_at	7791	0.97	-4.196574	3.99	0.0138099
224634_at	54865	0.95	-4.985559	3.15	0.0138227
232067_at	84553	1.12	4.782048	3.31	0.0138246
210951_x_at	5873	0.92	-6.279673	2.49	0.0138326
207735_at	54941	1.15	7.02316	2.27	0.0138336
229351_at	29964	0.92	-4.580701	3.50	0.0138484
206451_at	55171	0.94	-4.246374	3.91	0.0138561
213645_at	55556	0.97	-5.305147	2.93	0.0138604
208358_s_at	7368	1.09	6.878446	2.31	0.0138678
218441_s_at	26015	0.87	-4.36467	3.75	0.0138715
204280_at	10636	1.07	4.420311	3.68	0.0138754
225970_at	80821	1.12	5.03843	3.10	0.0138975
219491_at	78999	1.14	6.117653	2.55	0.0139067
228191_at	NA	1.04	4.510035	3.56	0.0139768
214877_at	54901	0.95	-7.928904	2.07	0.0140186
228146_at	339263	0.94	-4.205926	3.94	0.0140398
48106_at	55652	0.94	-4.502338	3.56	0.0140486
209199_s_at	4208	1.12	6.858838	2.30	0.0140652
204207_s_at	8732	0.97	-5.396658	2.86	0.014079
238880_at	2971	0.87	-4.357769	3.73	0.0140811
221865_at	203197	1.12	5.240845	2.95	0.0140838
242560_at	2177	0.98	-7.148931	2.23	0.0140867
206082_at	10866	0.89	-4.198076	3.95	0.014094
235919_at	NA	0.95	-4.165135	4.00	0.0141212
230055_at	80759	0.85	-4.869021	3.21	0.0141231
204905_s_at	9521	0.96	-4.284342	3.82	0.014135
202514_at	1739	0.96	-5.475002	2.81	0.0141462
216228_s_at	11169	0.89	-4.899636	3.18	0.0141487
218916_at	79724	0.97	-4.428851	3.63	0.0141765
203520_s_at	24149	0.95	-4.27375	3.83	0.0141832

208619_at	1642	1.03	4.41619	3.65	0.0141858
214686_at	10781	0.94	-5.370744	2.87	0.014193
228827_at	862	0.93	-4.231512	3.89	0.0142046
218781_at	79677	0.94	-4.194487	3.94	0.0142078
215489_x_at	9454	0.94	-4.659544	3.38	0.0142517
32062_at	9684	0.96	-4.216627	3.90	0.0142618
212323_s_at	55187	0.95	-4.163902	3.97	0.0143185
230763_at	128153	0.74	-6.242837	2.47	0.0143629
214749_s_at	54470	0.94	-4.990998	3.09	0.014367
224609_at	57153	0.90	-4.145386	3.99	0.0143706
228521_s_at	53916	0.97	-4.795427	3.25	0.014387
225855_at	57669	0.93	-5.13629	2.99	0.0144056
229446_at	NA	0.83	-4.163747	3.95	0.0144453
217726_at	22818	0.96	-4.234783	3.85	0.0144564
225684_at	348235	0.97	-5.376614	2.84	0.0144577
210130_s_at	7108	1.17	4.222445	3.87	0.0144584
202500_at	3300	0.90	-4.1392	3.99	0.0144599
201366_at	310	0.94	-4.183572	3.92	0.014468
221425_s_at	81689	0.96	-5.18589	2.95	0.0144979
203465_at	9801	0.98	-4.14385	3.98	0.0145023
212799_at	10228	1.07	4.629712	3.38	0.014528
219112_at	51735	0.90	-4.245808	3.82	0.0145398
226878_at	3111	0.85	-4.798955	3.22	0.0145952
223168_at	58480	0.93	-5.762927	2.64	0.0146041
223381_at	83540	0.98	-4.469748	3.53	0.0146297
219511_s_at	9627	0.89	-5.312138	2.86	0.0146804
213036_x_at	489	1.08	5.680595	2.67	0.0147114
218536_at	57380	0.96	-4.227311	3.83	0.0147134
218722_s_at	79714	0.94	-4.266537	3.77	0.014715
201555_at	4172	0.97	-5.786584	2.62	0.0147211
213056_at	23150	0.89	-4.161596	3.92	0.0147359
218718_at	56034	0.93	-4.120126	3.98	0.0147418
200075_s_at	2987	1.03	5.786136	2.62	0.0147995
216180_s_at	8871	1.22	5.917362	2.56	0.0148001
203714_s_at	6905	0.95	-7.05221	2.21	0.0148048
226157_at	7029	0.96	-4.352028	3.65	0.0148065
205774_at	2161	1.12	6.955437	2.23	0.0148346
215416_s_at	30968	0.97	-4.979059	3.06	0.0148421
225658_at	339745	0.94	-4.138365	3.94	0.0148489
232589_at	NA	0.89	-4.311401	3.70	0.0148529
215438_x_at	2935	1.02	5.890048	2.57	0.014855
37278_at	6901	0.96	-4.128272	3.95	0.0148689
212857_x_at	10923	1.03	4.162187	3.90	0.0148693
201801_s_at	2030	1.10	4.120422	3.96	0.0148806
205283_at	2218	0.94	-4.300461	3.70	0.0149384
228868_x_at	81620	0.98	-6.771547	2.28	0.0149487
41329_at	57147	0.90	-6.444092	2.37	0.0149541
36030_at	25900	1.07	4.262717	3.75	0.0149724
218315_s_at	51654	0.95	-4.895155	3.11	0.0149863
203307_at	2794	1.05	5.133666	2.94	0.0150083
218725_at	79751	1.05	4.089834	4.00	0.0150097
228341_at	131870	0.88	-4.319604	3.67	0.0150188

219648_at	55686	0.95	-7.598356	2.08	0.015023
44146_at	26205	0.96	-4.632666	3.33	0.0150407
209965_s_at	5892	0.92	-5.646864	2.66	0.0150411
225866_at	84154	0.96	-6.007405	2.51	0.0150431
44111_at	26276	0.96	-5.55537	2.70	0.0150607
225898_at	84058	0.93	-4.392725	3.57	0.0150624
209005_at	26234	0.94	-5.823201	2.58	0.0150631
202718_at	3485	1.09	4.412292	3.55	0.0150647
225459_at	154810	0.93	-4.596023	3.36	0.0150946
223384_s_at	89122	0.96	-6.132745	2.46	0.0151025
203487_s_at	25852	0.95	-4.891934	3.10	0.0151096
221987_s_at	55720	1.04	7.498158	2.10	0.0151156
225414_at	284996	1.07	8.026365	2.00	0.0151163
204917_s_at	4300	1.06	4.218282	3.79	0.0151423
226527_at	23248	0.97	-4.093266	3.97	0.0151512
213450_s_at	23308	1.05	5.133157	2.93	0.0151616
207839_s_at	51754	0.92	-4.803996	3.16	0.0151762
212837_at	23172	0.93	-7.270068	2.14	0.0151855
212282_at	27346	1.03	6.589634	2.31	0.0151935
209778_at	9321	0.89	-4.272077	3.71	0.0152031
225167_at	55691	1.09	4.249822	3.74	0.0152128
201111_at	1434	0.98	-5.180285	2.90	0.015242
219583_s_at	55812	0.86	-4.791512	3.17	0.0152547
205282_at	7804	1.07	4.192621	3.81	0.0152652
207357_s_at	55568	0.92	-4.068186	4.00	0.0152822
209086_x_at	4162	0.97	-5.082023	2.95	0.0152847
242938_s_at	3607	1.05	4.118411	3.91	0.0153078
204065_at	9486	0.96	-4.260395	3.71	0.0153176
227042_at	150223	0.96	-5.294061	2.82	0.0153284
227351_at	146174	0.93	-5.316598	2.81	0.0153366
226020_s_at	1600	0.95	-4.101321	3.94	0.0153381
229287_at	NA	0.92	-4.442974	3.49	0.0153412
219362_at	60560	0.97	-4.348878	3.59	0.0153595
225826_at	326625	0.94	-4.068548	3.98	0.0153789
227426_at	6654	0.94	-4.286056	3.67	0.0154056
201349_at	9368	1.08	6.054657	2.47	0.0154062
220334_at	26575	0.87	-5.916641	2.52	0.0154382
226750_at	55132	1.08	6.354392	2.37	0.0154942
219200_at	79072	0.95	-4.070735	3.96	0.0155087
235992_s_at	200008	0.90	-4.049563	3.99	0.0155483
212335_at	2799	1.05	4.361158	3.56	0.0155512
228075_x_at	51106	0.95	-4.04759	3.99	0.0155575
201760_s_at	55884	1.12	5.684829	2.61	0.0155638
228144_at	91975	0.92	-4.05227	3.98	0.0155708
215773_x_at	10038	0.95	-6.080632	2.45	0.0155836
210776_x_at	6929	0.95	-5.026118	2.96	0.0156229
222464_s_at	79892	0.97	-4.155887	3.82	0.0156345
203800_s_at	63931	0.96	-4.04955	3.98	0.0156378
227689_at	7770	0.94	-5.000932	2.98	0.0156765
215471_s_at	9053	1.07	4.034204	4.00	0.0156813
219654_at	9200	1.14	6.233816	2.39	0.0156819
226473_at	84733	0.94	-4.179114	3.78	0.015694

205175_s_at	3795	1.12	4.234263	3.70	0.0157138
213761_at	56890	0.88	-4.496779	3.39	0.0157264
218734_at	79829	0.93	-4.101312	3.88	0.0157695
229119_s_at	54902	0.94	-4.40266	3.49	0.0157712
215223_s_at	6648	1.07	7.733767	2.02	0.0157754
205993_s_at	6909	1.12	4.159702	3.79	0.0157766
225610_at	115426	0.96	-4.656692	3.23	0.0157981
205219_s_at	2585	0.88	-4.857481	3.07	0.0158165
205543_at	22824	0.88	-5.365547	2.75	0.0158312
205161_s_at	8800	0.90	-5.08482	2.91	0.0158433
200059_s_at	387	0.99	-5.194635	2.84	0.0158463
202733_at	8974	1.06	4.484225	3.39	0.0158568
228027_at	114928	0.93	-4.824092	3.09	0.0158631
203627_at	3480	1.04	6.246237	2.38	0.0158643
218499_at	51765	1.04	6.920339	2.19	0.0158842
227003_at	9364	0.96	-4.342503	3.54	0.0158931
219458_s_at	63899	0.93	-4.170907	3.76	0.0159066
224215_s_at	28514	1.07	5.767944	2.55	0.015944
201936_s_at	8672	0.97	-4.027853	3.97	0.0159601
203329_at	5797	1.07	4.015671	3.99	0.0160061
209230_s_at	26471	1.08	5.611196	2.61	0.0160096
219247_s_at	79683	1.09	4.411426	3.45	0.016024
238844_s_at	4867	0.84	-4.012101	3.99	0.0160305
227999_at	170394	1.07	4.270854	3.62	0.0160343
204693_at	11135	0.92	-4.588107	3.27	0.0160345
211450_s_at	2956	0.98	-5.094345	2.89	0.0160382
232860_x_at	55285	0.87	-5.624968	2.61	0.0160387
210768_x_at	54499	1.03	4.039294	3.94	0.0160718
214963_at	23279	0.93	-4.003892	4.00	0.0160821
231827_at	84515	0.93	-4.09634	3.85	0.0161121
202781_s_at	51763	1.05	4.134738	3.79	0.0161539
209695_at	11156	1.17	3.996597	4.00	0.0161865
90265_at	11033	1.02	4.036135	3.93	0.0162023
217988_at	57820	0.97	-4.186885	3.71	0.0162176
222867_s_at	51003	0.97	-4.066193	3.88	0.0162325
219327_s_at	55890	1.03	4.260113	3.61	0.0162525
209943_at	26235	0.91	-4.1608	3.74	0.0162616
200692_s_at	3313	0.97	-5.973126	2.45	0.0162704
226007_at	122961	0.98	-4.171671	3.72	0.0162716
201700_at	896	1.04	4.00138	3.98	0.0162737
203660_s_at	5116	1.04	4.397666	3.44	0.0162759
212954_at	8798	0.93	-4.572109	3.27	0.016299
209406_at	9532	0.96	-4.606437	3.23	0.0163111
209412_at	7109	0.93	-4.001739	3.97	0.0163126
224909_s_at	57580	1.12	4.087226	3.84	0.0163244
239169_at	201299	0.87	-5.554927	2.62	0.0163292
238963_at	92400	0.82	-4.941517	2.97	0.0163323
224416_s_at	80306	1.06	4.03709	3.91	0.0163366
238458_at	286097	0.90	-4.283397	3.57	0.0163392
233540_s_at	55755	0.94	-6.902653	2.17	0.0163581
214194_at	22894	0.95	-4.366521	3.47	0.0163797
201481_s_at	5834	1.04	7.033229	2.14	0.0163844

211177_s_at	10587	0.89	-6.039637	2.42	0.0163904
209894_at	3953	1.07	6.873359	2.18	0.0163924
212457_at	7030	0.93	-4.005684	3.95	0.0164268
227055_at	196410	1.11	4.888994	2.99	0.0164518
213634_s_at	55687	0.96	-4.022571	3.92	0.0164652
205250_s_at	80184	0.90	-4.292934	3.54	0.0164811
1552275_s_at	54899	1.05	4.137716	3.74	0.0165112
223226_x_at	170463	1.06	4.042885	3.88	0.0165277
225573_at	84129	0.92	-4.227218	3.61	0.0165813
209882_at	6016	0.96	-5.600613	2.58	0.0165958
219354_at	55295	0.90	-4.236245	3.60	0.0166093
203058_s_at	9060	1.08	4.011265	3.92	0.0166177
226188_at	29094	0.96	-4.031849	3.88	0.0166498
209204_at	8543	0.93	-4.784958	3.05	0.0166978
227856_at	132720	1.04	4.111728	3.76	0.0166994
211501_s_at	8662	1.06	4.282245	3.53	0.0167034
210570_x_at	5601	0.97	-4.009297	3.91	0.0167068
203383_s_at	2800	0.92	-6.356752	2.30	0.0167146
205130_at	5891	0.94	-6.310498	2.31	0.0167191
201475_x_at	4141	0.96	-4.741101	3.08	0.0167199
235609_at	83990	0.93	-4.737846	3.09	0.0167297
205637_s_at	6457	1.09	6.377286	2.29	0.0167418
220143_x_at	55692	0.95	-4.245401	3.57	0.0167437
213698_at	9204	0.89	-4.08951	3.79	0.0167476
1560477_a_at	148398	1.19	4.675817	3.13	0.016757
225482_at	547	1.11	5.444074	2.64	0.0167637
237040_at	143884	0.96	-4.139565	3.71	0.0167648
219663_s_at	80757	1.12	6.286319	2.32	0.0167654
227998_at	140576	1.14	3.972964	3.96	0.0167943
218890_x_at	51318	0.96	-6.702125	2.20	0.0168121
225804_at	124936	0.92	-4.042117	3.85	0.016831
224596_at	23446	0.96	-4.237237	3.57	0.0168655
226905_at	359845	1.11	4.712086	3.09	0.0168752
221492_s_at	64422	0.95	-3.98899	3.93	0.0168842
209530_at	784	1.04	5.881021	2.45	0.016888
223649_s_at	51629	1.04	4.109086	3.74	0.016893
217448_s_at	9878	1.07	4.788604	3.03	0.0169026
229793_at	29974	0.89	-4.545153	3.24	0.0169173
240280_at	402682	0.90	-3.942666	4.00	0.0169222
207709_at	5563	0.89	-7.193409	2.08	0.0169231
226434_at	221908	0.94	-4.412212	3.37	0.0169234
202358_s_at	399979	0.91	-5.16438	2.78	0.0169446
232219_x_at	27005	0.93	-5.122456	2.80	0.0169653
224859_at	80381	1.02	5.583333	2.57	0.0169778
1552347_at	9946	0.95	-4.074114	3.78	0.0169932
226566_at	81559	1.04	4.047839	3.81	0.0170401
225806_at	84962	0.88	-5.448684	2.62	0.017046
208116_s_at	4121	1.09	4.756504	3.05	0.0170514
207165_at	3161	0.97	-4.086013	3.76	0.0170618
209736_at	9580	1.06	4.118652	3.70	0.0171088
230281_at	123775	1.10	5.260866	2.72	0.0171121
208107_s_at	81691	0.91	-4.547335	3.22	0.0171246

224415_s_at	84681	0.94	-4.918806	2.92	0.0171277
226065_at	144165	0.96	-3.930948	3.99	0.0171377
215716_s_at	490	1.04	3.934662	3.99	0.0171396
220954_s_at	29990	0.90	-4.588707	3.18	0.0171601
219123_at	7775	0.91	-5.35903	2.66	0.017163
231969_at	56977	1.20	6.43924	2.25	0.0171809
224587_at	10923	1.06	3.981878	3.90	0.0171991
204676_at	25880	0.94	-3.937844	3.97	0.0172101
228652_at	284309	0.94	-4.537533	3.21	0.0172515
225955_at	284207	1.09	4.89239	2.93	0.017255
205284_at	9816	0.94	-4.369655	3.38	0.0173021
227836_at	84294	0.92	-4.210673	3.56	0.0173167
202020_s_at	10314	0.97	-4.725554	3.05	0.0173363
212453_at	26128	0.96	-4.267872	3.49	0.0173398
218726_at	55355	1.02	3.915534	4.00	0.0173411
233167_at	83642	1.12	4.685679	3.08	0.0173623
225512_at	253461	1.04	4.458352	3.28	0.0173658
209815_at	5727	1.03	4.648677	3.11	0.0173715
202246_s_at	1019	0.99	-4.085987	3.72	0.0173729
218676_s_at	58488	0.95	-4.010333	3.84	0.0173738
235911_at	440995	1.05	4.668583	3.09	0.0173779
210428_s_at	9146	1.08	6.230392	2.30	0.0173803
204603_at	9156	0.93	-6.463381	2.23	0.0174059
216841_s_at	6648	1.08	3.912183	3.99	0.0174135
205667_at	7486	0.96	-4.727971	3.04	0.0174288
200770_s_at	3915	0.99	-4.398447	3.33	0.0174323
218196_at	28962	0.96	-5.400999	2.62	0.0174495
222843_at	63979	0.95	-5.522744	2.56	0.0174549
228745_at	80006	1.12	5.835846	2.43	0.0174631
226387_at	222194	0.98	-3.946908	3.93	0.0174674
230356_at	NA	1.14	5.398976	2.62	0.0174707
224598_at	11282	1.02	3.929131	3.96	0.0174709
221888_at	54862	0.96	-3.961359	3.90	0.0174752
220669_at	54726	0.88	-4.016203	3.81	0.0174837
225900_at	23233	0.90	-4.206544	3.55	0.0174885
208927_at	8405	0.97	-4.342919	3.39	0.0174903
222785_x_at	64776	0.91	-4.019403	3.81	0.0174974
205005_s_at	9397	0.93	-4.906447	2.90	0.0174991
210463_x_at	55621	1.07	3.992161	3.84	0.0175491
212357_at	23201	0.95	-4.699084	3.05	0.0175568
236641_at	9928	0.95	-5.322488	2.65	0.0175672
225935_at	NA	0.94	-4.131891	3.64	0.0175761
202527_s_at	4089	0.97	-4.75853	3.00	0.017583
201270_x_at	23386	0.95	-4.370252	3.35	0.0175972
218662_s_at	64151	0.96	-4.171609	3.58	0.0176107
37512_at	8630	1.16	4.317382	3.41	0.0176141
227865_at	414328	0.89	-6.282516	2.27	0.0176335
205794_s_at	4857	0.87	-4.086465	3.69	0.0176482
227139_s_at	84343	0.94	-4.75787	3.00	0.0176715
223313_s_at	81557	0.92	-5.615758	2.51	0.0176857
243118_at	285753	0.88	-3.908459	3.97	0.0176869
242655_at	580	0.92	-3.924815	3.94	0.0177208

203910_at	9411	1.06	3.997154	3.81	0.0177441
225363_at	5728	0.97	-4.457438	3.25	0.0177449
226120_at	123016	0.93	-4.770922	2.98	0.0177549
210638_s_at	26268	0.97	-4.5436	3.17	0.0177618
223947_s_at	9439	0.95	-4.761774	2.99	0.0177652
204023_at	5984	0.96	-5.205943	2.70	0.0177865
58367_s_at	79744	0.97	-4.015285	3.78	0.0178018
203350_at	164	0.97	-3.887363	3.99	0.0178417
219919_s_at	54961	1.11	3.961171	3.86	0.0178458
226889_at	57539	0.92	-3.98116	3.83	0.0178659
209273_s_at	81689	0.95	-4.001218	3.79	0.0178668
212166_at	23039	0.94	-4.222797	3.49	0.017874
203133_at	10952	1.02	3.923404	3.92	0.0178911
233898_s_at	26127	0.94	-4.39476	3.30	0.0178917
227672_at	619348	1.19	7.190845	2.04	0.0179154
219202_at	79651	1.15	5.332876	2.63	0.0179394
212749_s_at	25898	0.94	-4.19919	3.51	0.0179762
208680_at	5052	1.02	3.94117	3.88	0.0179768
211681_s_at	10611	1.14	4.014522	3.76	0.0179776
231932_at	80342	0.83	-4.296714	3.39	0.0179867
221744_at	10238	1.10	3.86954	4.00	0.0180008
213730_x_at	6929	0.95	-4.658241	3.05	0.0180259
214276_at	11278	0.92	-3.916604	3.91	0.0180321
222617_s_at	63877	0.95	-6.736238	2.13	0.0180606
227584_at	89796	1.08	3.86503	4.00	0.0180717
204599_s_at	10573	1.03	4.431206	3.24	0.0181349
212904_at	57470	1.02	3.873236	3.98	0.0181376
218909_at	26750	0.94	-4.278654	3.40	0.0181385
46256_at	90864	1.06	6.108613	2.30	0.0181418
225077_at	283680	0.94	-4.409969	3.26	0.0181447
218715_at	55813	0.98	-3.954225	3.83	0.0181811
234735_s_at	27005	0.92	-5.136561	2.71	0.0181893
223055_s_at	57510	0.97	-7.018998	2.06	0.0181987
220744_s_at	55764	0.91	-3.928956	3.87	0.0182018
223026_s_at	51699	0.95	-5.660443	2.46	0.018204
207618_s_at	617	0.95	-5.698149	2.45	0.0182179
234465_at	146956	0.92	-5.968453	2.35	0.0182381
220924_s_at	54407	0.99	-4.056225	3.67	0.0182715
206235_at	3981	0.84	-4.120636	3.58	0.0182934
203534_at	27257	0.97	-3.905372	3.90	0.0182992
231940_at	57711	0.94	-4.267598	3.40	0.0183226
210802_s_at	27292	0.97	-3.851058	3.99	0.0183458
221622_s_at	55863	0.94	-4.522225	3.13	0.0183953
227658_s_at	65977	0.93	-5.042597	2.76	0.0184026
222801_s_at	64940	0.92	-3.84414	4.00	0.0184167
225209_s_at	118424	0.97	-6.031497	2.31	0.0184574
212485_at	23131	0.93	-3.980981	3.76	0.018492
209591_s_at	655	1.10	4.320171	3.32	0.018494
226032_at	835	0.96	-4.73658	2.95	0.0185268
212439_at	9807	0.92	-4.299912	3.34	0.0185638
203256_at	1001	1.15	3.832142	4.00	0.0185854
223066_at	23557	0.96	-6.135468	2.27	0.0186041

219882_at	79739	0.95	-3.831401	4.00	0.0186055
211941_s_at	5037	1.03	4.232882	3.41	0.0186551
209165_at	26574	0.97	-4.689898	2.98	0.0186716
220547_s_at	54537	1.04	6.283869	2.22	0.0186738
215273_s_at	10474	0.92	-5.564505	2.48	0.0186858
218354_at	51693	1.05	4.138147	3.51	0.0187442
219539_at	79833	0.96	-3.821933	4.00	0.0187648
201412_at	26020	1.11	5.53662	2.48	0.0188022
228041_at	132949	0.91	-4.82653	2.87	0.0188026
227098_at	150290	0.91	-4.253658	3.37	0.0188103
221028_s_at	81577	0.93	-3.849273	3.94	0.0188114
206935_at	5100	1.15	4.226941	3.40	0.018818
203467_at	5372	0.94	-4.087385	3.57	0.0188351
203981_s_at	5826	0.95	-4.451569	3.16	0.0188488
210386_s_at	4580	0.97	-5.018005	2.74	0.0188566
205562_at	10557	0.88	-4.161975	3.47	0.0188628
209860_s_at	310	0.95	-4.17953	3.45	0.0188679
205891_at	136	1.08	4.646858	2.99	0.0189421
217983_s_at	8635	1.07	6.070649	2.28	0.018952
204832_s_at	657	0.96	-3.956652	3.74	0.0190061
222894_x_at	79133	0.92	-4.341645	3.26	0.0190325
241710_at	130271	0.87	-3.899729	3.83	0.0190546
211343_s_at	1305	1.23	4.068956	3.58	0.0190649
218947_s_at	55149	0.91	-5.137163	2.66	0.0190679
209458_x_at	3040	1.33	7.102797	2.01	0.0190714
235961_at	23432	1.12	3.887436	3.85	0.0190747
212731_at	157567	1.08	5.11077	2.67	0.0190979
218824_at	55228	0.97	-7.005422	2.03	0.0191009
205251_at	8864	1.12	5.123066	2.67	0.0191038
218538_s_at	57380	0.95	-3.987401	3.69	0.019112
226558_at	150483	0.96	-4.680784	2.95	0.0191174
202898_at	9672	1.11	4.42022	3.17	0.0191213
227337_at	353322	1.20	4.03131	3.62	0.0191409
213334_x_at	11219	0.96	-4.768508	2.88	0.0191505
206915_at	4821	1.10	3.877119	3.86	0.01916
242515_x_at	56672	0.98	-3.887394	3.84	0.0191744
212317_at	23534	0.95	-5.344367	2.55	0.0191751
201046_s_at	5886	0.96	-4.147869	3.46	0.0191983
203035_s_at	10401	0.91	-4.578607	3.03	0.0191984
219618_at	51135	0.87	-4.302668	3.28	0.0192004
226867_at	55667	0.95	-5.994812	2.29	0.0192022
201586_s_at	6421	1.02	4.014669	3.64	0.019215
204435_at	9818	0.83	-3.81793	3.95	0.019226
35179_at	26229	0.96	-3.816237	3.95	0.0192472
228378_at	91298	0.93	-3.788978	4.00	0.0192863
202032_s_at	4122	1.07	4.539696	3.05	0.0192939
210008_s_at	6183	1.07	4.178843	3.41	0.019306
228249_at	119710	0.93	-4.261939	3.32	0.0193104
201443_s_at	10159	1.03	4.517956	3.07	0.0193109
228830_s_at	11016	0.93	-4.574008	3.02	0.0193154
204408_at	27301	0.95	-5.522763	2.46	0.0193612
238813_at	212	0.92	-4.634277	2.97	0.0193689

213811_x_at	6929	0.96	-6.999077	2.02	0.0193992
214045_at	11019	0.89	-4.166304	3.42	0.0194034
244032_at	79840	0.90	-4.495782	3.08	0.0194186
228595_at	3292	0.90	-4.842792	2.81	0.0194282
221808_at	9367	0.96	-5.433832	2.49	0.0194316
209963_s_at	2057	1.15	6.679839	2.08	0.0194694
221762_s_at	63935	0.97	-4.12203	3.47	0.0194926
227898_s_at	286128	0.88	-4.315962	3.25	0.0194968
244359_s_at	401131	0.96	-3.81186	3.93	0.019509
227064_at	91369	0.95	-5.910892	2.30	0.0195181
235064_s_at	149840	0.90	-3.931593	3.73	0.0195281
204283_at	10667	0.93	-5.312728	2.54	0.0195354
204717_s_at	3177	0.87	-4.201268	3.37	0.0195367
228314_at	84230	0.95	-3.861553	3.84	0.0196078
201334_s_at	23365	0.97	-4.926203	2.75	0.0196181
223421_at	50626	0.96	-3.773698	3.99	0.0196242
200090_at	2339	0.98	-3.813538	3.91	0.0196594
209533_s_at	9373	0.96	-3.778437	3.98	0.0196598
243252_at	NA	0.88	-5.731718	2.36	0.0196637
204093_at	902	1.03	6.323279	2.17	0.0197184
226650_at	90637	1.06	3.766334	3.99	0.0197274
211034_s_at	196515	0.95	-4.245462	3.30	0.0197417
219879_s_at	78995	0.95	-4.064303	3.52	0.0197462
AFFX-r2-P1-cre-5_at	2777477	0.99	-3.922542	3.72	0.0197534
227720_at	124930	1.12	5.071239	2.66	0.0197555
213134_x_at	10950	0.99	-4.621932	2.95	0.0197582
209592_s_at	10238	1.09	6.469333	2.13	0.0197611
202729_s_at	4052	1.10	3.760896	4.00	0.0197637
219407_s_at	10319	1.14	4.185266	3.36	0.0198235
220326_s_at	55701	0.91	-3.851757	3.83	0.0198292
224500_s_at	84315	0.95	-3.778323	3.96	0.0198385
223773_s_at	85028	1.07	5.777344	2.33	0.0198591
209838_at	9318	0.96	-4.796168	2.82	0.0199104
218139_s_at	55745	1.02	3.920486	3.71	0.0199205
239376_at	NA	0.94	-4.219535	3.31	0.0199453
226316_at	64062	0.94	-4.967139	2.70	0.0199567
225891_at	286262	1.06	3.811618	3.88	0.0200035
227804_at	116238	1.07	3.934564	3.68	0.0200063
220617_s_at	55205	0.94	-3.823371	3.85	0.0200631
218374_s_at	57102	0.93	-5.100117	2.62	0.0200848
203999_at	6857	0.95	-3.753576	3.97	0.0201134
235301_at	222223	1.06	3.863143	3.78	0.0201236
222990_at	29979	1.03	3.969423	3.61	0.0201329
225017_at	64770	0.92	-5.166755	2.58	0.0201614
213186_at	9666	0.93	-4.29136	3.22	0.0201622
203739_at	7764	0.89	-4.324424	3.18	0.0201629
223407_at	84080	0.93	-4.09846	3.44	0.0201765
224162_s_at	79791	1.08	3.945663	3.64	0.0201774
219307_at	57107	0.91	-4.459687	3.06	0.0201884
213463_s_at	317662	0.94	-3.987589	3.58	0.0201986
212088_at	23203	1.07	3.736597	4.00	0.0202009
228516_at	146059	0.94	-4.273127	3.23	0.0202472

228949_at	57708	0.89	-6.537328	2.09	0.0202667
218278_at	54663	1.03	4.745885	2.83	0.0202718
203072_at	4643	0.96	-3.784756	3.90	0.0202775
221229_s_at	55006	0.94	-3.842872	3.80	0.0202852
225197_at	29035	0.91	-3.731235	4.00	0.0202986
231727_s_at	57409	0.93	-4.664239	2.88	0.020341
231530_s_at	64776	0.93	-4.35332	3.14	0.0203731
205437_at	10520	0.84	-6.176769	2.18	0.0204008
221769_at	90864	1.08	4.256566	3.23	0.0204148
209434_s_at	5471	0.96	-3.726531	3.99	0.020454
219961_s_at	55857	0.96	-3.752843	3.94	0.0204775
49485_at	11108	0.92	-4.45771	3.04	0.0204791
220201_at	54542	0.94	-5.211208	2.54	0.0204863
202188_at	9688	0.93	-5.978777	2.24	0.0204964
213199_at	26005	0.90	-4.126381	3.37	0.0205061
228145_s_at	57541	0.93	-4.700566	2.84	0.0205254
235545_at	55635	0.96	-5.140969	2.57	0.0205378
207845_s_at	10393	0.96	-4.030157	3.49	0.020545
208789_at	284119	1.09	6.225335	2.16	0.0205472
235509_at	137682	0.94	-3.713827	4.00	0.0205896
225633_at	147991	0.91	-4.960914	2.67	0.0206714
202889_x_at	9053	1.09	4.562775	2.94	0.0206781
224962_at	90120	0.95	-3.800585	3.83	0.0206861
203916_at	8509	0.97	-4.300835	3.17	0.0207135
223307_at	83461	0.99	-3.750529	3.91	0.0207253
216602_s_at	2193	0.95	-3.810598	3.80	0.0207584
210061_at	51385	0.88	-4.321859	3.14	0.0207586
201989_s_at	1389	0.96	-4.558899	2.93	0.0208057
229892_at	347918	0.93	-4.809727	2.75	0.0208357
228379_at	10204	0.97	-6.021168	2.21	0.0208453
225975_at	54510	0.94	-3.727767	3.94	0.0208872
228323_at	57082	0.95	-5.114298	2.57	0.0208886
219277_s_at	55753	0.91	-4.830804	2.73	0.0208891
211543_s_at	2870	0.96	-3.697016	4.00	0.0208945
219487_at	79738	0.90	-4.2539	3.20	0.0209683
209707_at	10026	0.96	-4.078691	3.39	0.0209715
209471_s_at	2339	0.98	-4.959248	2.65	0.0209756
218979_at	80010	0.94	-4.262274	3.19	0.0209797
222466_s_at	28977	0.98	-3.776122	3.84	0.0209885
204831_at	1024	1.07	3.796691	3.80	0.0209996
54037_at	89781	0.89	-3.916353	3.61	0.0210163
208884_s_at	51366	0.93	-4.532896	2.94	0.0210291
230165_at	151246	0.94	-4.472191	2.99	0.021034
226470_at	2686	0.93	-4.321248	3.12	0.0210511
239302_s_at	NA	0.87	-3.791678	3.81	0.0210548
202956_at	10565	0.95	-3.773892	3.84	0.0210551
228131_at	10849	0.87	-4.165605	3.28	0.0210553
217944_at	541468	1.05	6.234425	2.14	0.0210587
45687_at	78994	0.97	-4.638895	2.85	0.0210625
220305_at	78993	1.11	3.858296	3.69	0.0210809
202978_s_at	58487	0.97	-4.325427	3.11	0.0211175
223290_at	57026	1.05	3.945126	3.56	0.0211318

219361_s_at	64782	0.92	-5.514998	2.37	0.0211401
208913_at	23062	1.06	3.942959	3.56	0.0211533
201573_s_at	2107	1.03	4.44943	3.00	0.0211676
208722_s_at	51433	0.95	-5.505574	2.37	0.0211969
225869_s_at	81622	1.16	4.391217	3.05	0.021205
218526_s_at	29098	0.97	-3.98213	3.50	0.0212086
225587_at	92305	0.90	-3.752041	3.86	0.0212235
221685_s_at	54908	0.96	-3.760033	3.84	0.0212617
229468_at	1018	1.07	4.046925	3.41	0.0212803
209790_s_at	839	0.95	-4.524946	2.93	0.0212875
218108_at	55148	0.97	-5.676421	2.30	0.0212895
226844_at	79817	1.09	3.674676	4.00	0.0213111
221774_x_at	55578	0.88	-3.737698	3.88	0.0213156
205769_at	11001	1.13	5.828577	2.25	0.0213397
203061_s_at	9656	0.91	-4.024609	3.43	0.0213484
230031_at	3309	1.06	3.676716	3.99	0.0213604
217921_at	10905	1.10	4.132626	3.30	0.0213705
223021_x_at	51534	0.94	-6.095775	2.16	0.0213788
201365_at	4947	0.95	-4.080657	3.36	0.0213913
219172_at	80019	1.13	4.69668	2.79	0.0214169
203249_at	2145	0.95	-4.018271	3.43	0.0214724
215084_s_at	115353	0.93	-3.741591	3.85	0.021497
220939_s_at	54878	0.95	-4.719365	2.77	0.0215054
219258_at	54962	0.95	-5.203677	2.49	0.0215236
224647_at	219771	0.95	-4.739693	2.75	0.0215303
209849_s_at	5889	0.97	-3.662859	4.00	0.0215347
204067_at	6821	0.95	-4.276856	3.13	0.0215476
224782_at	153527	1.04	3.750936	3.83	0.0215802
203124_s_at	4891	0.93	-5.302984	2.44	0.0215964
208718_at	10521	0.98	-3.692568	3.93	0.0215982
201980_s_at	6251	1.05	4.700499	2.78	0.0216149
229512_at	NA	0.90	-3.854107	3.65	0.0216674
200098_s_at	51433	0.97	-5.087806	2.54	0.021675
231921_at	80067	0.89	-3.675225	3.96	0.0217044
203538_at	819	1.04	6.34025	2.08	0.0217056
200094_s_at	1938	1.03	4.777374	2.72	0.0217553
227431_at	222194	1.04	5.219349	2.47	0.0217616
215280_s_at	8541	1.11	4.398114	3.00	0.021762
218449_at	55325	0.94	-3.840321	3.66	0.0217666
203594_at	8634	0.98	-5.040119	2.56	0.0217671
228968_at	203523	0.93	-3.768479	3.78	0.0217728
218945_at	79091	0.91	-4.432629	2.97	0.0217838
232683_s_at	56965	0.93	-4.976785	2.60	0.0217939
211595_s_at	64963	0.94	-4.080443	3.32	0.0218105
230326_s_at	51501	0.93	-5.863714	2.22	0.0218119
213106_at	10396	1.12	5.630286	2.30	0.0218421
209654_at	23379	0.95	-3.924293	3.53	0.0218456
218125_s_at	55246	0.96	-4.058673	3.35	0.0218746
226600_at	160418	0.96	-3.834774	3.66	0.0218986
213666_at	23157	0.94	-4.755939	2.72	0.0219102
223222_at	60386	0.95	-4.067502	3.33	0.0219171
204341_at	147166	0.92	-4.218677	3.16	0.0219263

236075_s_at	7775	0.90	-3.643135	4.00	0.0219495
210145_at	5321	0.89	-3.771347	3.75	0.0219805
224750_at	91445	0.94	-4.144861	3.23	0.0220156
220341_s_at	51149	0.96	-4.089977	3.30	0.0220248
204423_at	4289	0.94	-4.753536	2.72	0.0220281
218866_s_at	51728	0.95	-3.725373	3.83	0.0220356
217780_at	51398	0.98	-5.083547	2.53	0.0220436
41512_at	8315	0.92	-3.777377	3.73	0.022068
223847_s_at	57222	0.96	-3.903049	3.54	0.022072
212089_at	4000	1.05	6.067314	2.14	0.0220827
213918_s_at	25836	0.96	-3.63204	4.00	0.0221199
209475_at	9958	0.91	-4.077709	3.30	0.022139
225601_at	3149	1.05	3.761838	3.75	0.0221401
214356_s_at	23392	1.05	3.694172	3.87	0.0221474
242521_at	NA	1.13	5.401436	2.37	0.0221539
204016_at	23395	0.95	-4.626499	2.80	0.0221753
220941_s_at	54149	0.92	-3.726665	3.81	0.0221824
209100_at	7866	0.96	-6.235575	2.09	0.0221926
224988_at	221477	0.93	-3.633071	3.99	0.022197
35156_at	203069	0.98	-3.64679	3.96	0.0222236
218605_at	64216	1.04	4.924745	2.60	0.0222303
209300_s_at	25977	0.96	-4.796825	2.68	0.0222432
230486_at	54039	1.07	3.746221	3.77	0.0222518
222444_at	51566	0.96	-4.009905	3.37	0.0222912
212188_at	115207	1.06	3.929936	3.48	0.0222966
44702_at	85360	0.96	-3.692856	3.86	0.0223145
238035_at	6670	0.95	-5.110518	2.50	0.022316
204862_s_at	4832	1.04	3.974946	3.41	0.0223433
238607_at	162979	1.12	3.637633	3.96	0.0223482
219248_at	80745	0.91	-6.055502	2.14	0.0223504
219038_at	79710	1.03	3.618973	4.00	0.0223779
214220_s_at	7840	0.93	-3.77368	3.71	0.022378
208788_at	60481	1.05	4.98359	2.56	0.0223803
212630_at	11336	1.02	3.618927	4.00	0.0223911
203816_at	1716	0.94	-3.643513	3.95	0.0224224
233564_s_at	81602	0.88	-6.208662	2.09	0.0224386
236958_at	79074	0.92	-5.658215	2.26	0.022473
204427_s_at	10959	1.05	3.620509	3.99	0.0224762
225680_at	222229	1.05	3.654037	3.92	0.0224816
225629_s_at	57659	1.10	4.229625	3.11	0.022506
219343_at	55664	0.88	-3.620047	3.98	0.022524
212666_at	57154	0.91	-4.256175	3.08	0.0225273
229014_at	441094	0.92	-4.115245	3.23	0.0225305
201990_s_at	1389	0.95	-6.426381	2.03	0.0225651
229493_at	401021	0.93	-3.991623	3.37	0.0225675
228482_at	284040	0.89	-3.639676	3.94	0.0225721
204234_s_at	7748	0.90	-5.6758	2.25	0.0225787
241991_at	159091	0.84	-5.174068	2.45	0.0225801
203381_s_at	348	1.20	3.609503	4.00	0.0225902
39705_at	23309	0.92	-3.988317	3.37	0.0226233
205052_at	549	0.97	-3.659656	3.89	0.0226661
213659_at	7626	0.93	-4.089922	3.25	0.0226957

203695_s_at	1687	1.06	3.775372	3.68	0.0227059
212798_s_at	57037	1.05	5.575447	2.28	0.0227286
218518_at	51306	0.94	-4.672039	2.73	0.0227496
204662_at	9738	0.95	-5.145553	2.46	0.0227497
226027_at	375757	0.92	-3.618272	3.96	0.0227504
228275_at	NA	0.94	-3.604039	3.99	0.0227545
218649_x_at	9147	0.95	-3.712452	3.78	0.0227695
200855_at	9611	0.94	-3.629051	3.94	0.0227991
214761_at	23090	1.17	4.240384	3.08	0.0228204
219990_at	79733	0.97	-4.661025	2.73	0.0228221
227572_at	84749	0.95	-3.876202	3.51	0.0228307
226383_at	120534	0.95	-4.646337	2.74	0.0228345
222427_s_at	51520	0.97	-3.59607	4.00	0.0228772
212940_at	1291	1.14	3.722392	3.75	0.022896
207515_s_at	9533	0.98	-4.616986	2.76	0.0228971
242283_at	200095	0.92	-4.276358	3.04	0.0229276
200969_at	27230	1.04	3.854868	3.53	0.0229377
219020_at	64342	0.94	-4.276953	3.03	0.0229671
201995_at	2131	0.96	-3.618449	3.94	0.022971
213505_s_at	10147	0.98	-3.757927	3.68	0.0229901
234915_s_at	8562	0.96	-5.309239	2.37	0.0229935
205742_at	7137	0.84	-4.04494	3.28	0.0230038
225808_at	124512	0.96	-4.252432	3.05	0.0230289
204354_at	25913	0.96	-4.435404	2.89	0.023035
221786_at	387263	0.98	-4.834873	2.61	0.0230648
229744_at	6744	0.88	-3.898456	3.46	0.0231154
228555_at	817	1.11	4.863503	2.59	0.0231474
210592_s_at	6303	1.02	4.592036	2.76	0.0231697
218940_at	79609	0.94	-3.61663	3.92	0.0231787
223060_at	55017	0.96	-3.579078	4.00	0.0232081
225194_at	5356	0.96	-5.082132	2.47	0.0232246
202569_s_at	4140	0.96	-4.656473	2.71	0.0232303
226202_at	57541	0.93	-4.280345	3.01	0.0232425
43511_s_at	NA	1.17	5.439102	2.31	0.0232496
224897_at	80232	1.03	4.959155	2.53	0.023251
226528_at	345778	0.92	-3.575977	4.00	0.0232622
229208_at	55142	0.92	-6.433239	2.00	0.0232634
201938_at	8099	0.99	-6.30774	2.03	0.0233029
218027_at	29088	0.97	-4.182259	3.10	0.0233067
226087_at	84328	0.95	-3.704451	3.74	0.0233232
225971_at	80821	1.04	3.697958	3.75	0.0233643
224511_s_at	84817	1.03	3.925599	3.40	0.0233732
218780_at	29911	0.92	-5.366174	2.33	0.0233926
212557_at	26036	0.96	-4.263945	3.02	0.0233987
209075_s_at	23479	0.98	-3.603851	3.93	0.0234079
229228_at	9586	0.92	-3.598658	3.94	0.0234164
212214_at	4976	0.94	-5.202762	2.40	0.0234597
221664_s_at	50848	1.21	5.138876	2.43	0.0234608
229674_at	56256	0.92	-3.591512	3.94	0.0234819
221962_s_at	7328	1.06	6.375424	2.01	0.0235252
214683_s_at	1195	0.96	-4.147318	3.12	0.0235512
227620_at	NA	0.92	-4.207972	3.06	0.023553

209702_at	79068	0.97	-6.308056	2.02	0.0235534
218729_at	56925	1.07	3.577585	3.97	0.0235543
204459_at	1478	0.98	-3.78018	3.60	0.0235607
230062_at	440804	1.14	4.618104	2.72	0.0235768
229377_at	79774	0.90	-3.631657	3.85	0.0236093
204394_at	8501	1.07	3.564018	3.99	0.0236184
219512_at	79980	0.93	-5.796276	2.17	0.0236591
222977_at	6836	1.04	5.527195	2.26	0.023671
212539_at	9557	0.97	-4.775148	2.62	0.0236724
214713_at	56252	0.94	-4.988174	2.50	0.0236779
236798_at	NA	0.94	-4.492394	2.81	0.0236875
229984_at	56986	0.90	-3.588973	3.93	0.0236964
225272_at	112483	0.96	-4.620866	2.71	0.0237221
205286_at	7022	0.91	-5.283923	2.35	0.023725
214765_s_at	27163	1.07	3.569409	3.97	0.023732
51146_at	55650	0.93	-3.553291	4.00	0.0237446
203274_at	474383	0.96	-4.726684	2.64	0.0237598
225429_at	5537	1.02	3.586037	3.93	0.0237647
227394_at	4684	0.93	-3.669551	3.76	0.0237893
211602_s_at	7220	0.81	-5.21618	2.38	0.0238027
218802_at	55013	1.07	5.31913	2.33	0.0238158
227604_at	151146	0.92	-3.92382	3.36	0.0238743
230434_at	493911	0.89	-3.571983	3.94	0.0238953
227193_at	283651	0.95	-3.659408	3.77	0.0238968
202846_s_at	5279	0.96	-3.929453	3.35	0.0239144
204824_at	2021	1.07	3.985744	3.28	0.0239238
213187_x_at	2512	1.03	6.092362	2.07	0.0239249
227249_at	4629	0.96	-4.052685	3.20	0.0239292
238662_at	89978	0.88	-5.422992	2.29	0.0239463
208821_at	6628	1.02	3.827927	3.49	0.0239489
212041_at	9114	1.06	3.658302	3.77	0.0239636
201715_s_at	22985	0.93	-3.623951	3.83	0.0239823
201029_s_at	4267	0.97	-3.70569	3.68	0.0240097
202852_s_at	79719	0.95	-5.826601	2.14	0.0240126
202702_at	7726	0.93	-3.543031	3.99	0.0240508
236478_at	3454	0.88	-4.664019	2.67	0.0240605
206038_s_at	7182	0.86	-3.761752	3.58	0.0240862
208787_at	11222	0.98	-3.690466	3.70	0.0241178
225838_at	26122	0.96	-3.702692	3.67	0.0241281
235812_at	255919	0.96	-3.562022	3.94	0.0241433
1552287_s_at	172	0.89	-4.625539	2.69	0.02415
224437_s_at	51534	0.96	-3.564985	3.93	0.0241696
238561_s_at	84294	0.87	-4.825767	2.56	0.0242025
234985_at	143458	1.05	3.564435	3.93	0.0242111
244321_at	80055	0.89	-4.847125	2.55	0.0242354
212535_at	4205	0.95	-3.55968	3.93	0.0242521
241535_at	NA	0.87	-3.772494	3.55	0.0242736
203977_at	6901	0.95	-4.208766	3.01	0.0242974
220607_x_at	51497	0.98	-3.794492	3.51	0.0243041
226037_s_at	51616	0.96	-5.312545	2.32	0.0243177
201386_s_at	1665	0.98	-4.072082	3.15	0.024331
226854_at	26164	0.93	-4.829963	2.55	0.0243493

211936_at	3309	1.03	4.368278	2.87	0.0243539
225024_at	58490	0.96	-4.067371	3.15	0.024361
218298_s_at	80017	1.10	4.174032	3.04	0.0244
235411_at	84547	0.89	-4.681671	2.64	0.0244096
212815_at	10973	0.94	-3.783288	3.52	0.0244207
235343_at	79805	1.19	5.822914	2.13	0.0244209
212740_at	30849	0.94	-3.96771	3.27	0.0244269
203612_at	705	0.96	-3.526131	3.99	0.024431
206431_x_at	23061	0.95	-3.520815	4.00	0.0244368
226515_at	133957	0.97	-4.100862	3.11	0.0244565
224984_at	10725	0.95	-4.086452	3.13	0.0244625
206023_at	10874	1.04	5.010102	2.45	0.0244637
242093_at	94122	1.08	5.861019	2.11	0.0244974
218137_s_at	60682	1.03	4.445772	2.80	0.0245197
227639_at	10026	0.94	-3.74962	3.56	0.0245277
226371_at	5927	0.94	-3.523914	3.98	0.0245308
219960_s_at	51377	0.96	-3.61863	3.79	0.0245533
203466_at	4358	0.92	-4.666377	2.63	0.0246447
223228_at	84247	0.92	-3.513858	3.99	0.0246464
224836_at	58476	1.19	5.714313	2.15	0.0246476
207508_at	518	1.02	3.574649	3.87	0.0246483
212540_at	997	1.02	3.586096	3.84	0.0247162
202055_at	3836	0.98	-3.569078	3.87	0.0247278
204462_s_at	6567	1.07	3.594915	3.81	0.0247857
1729_at	8717	0.95	-3.563611	3.87	0.0247907
225521_at	51434	0.92	-5.258315	2.32	0.0247988
202654_x_at	64844	0.96	-3.865178	3.37	0.0248076
229120_s_at	56882	0.97	-3.992166	3.21	0.0248417
225311_at	3712	0.95	-6.055973	2.04	0.0248452
204517_at	5480	1.04	4.026201	3.17	0.0248479
234936_s_at	57545	0.90	-4.231871	2.96	0.0248497
204353_s_at	25913	0.94	-3.916833	3.30	0.0248794
210235_s_at	8500	0.95	-3.99672	3.20	0.0249419
202673_at	8813	1.02	3.500859	3.99	0.0249768
220753_s_at	51084	1.17	3.799015	3.45	0.0249784
217923_at	553115	0.96	-4.793904	2.54	0.025043
226779_at	92255	0.96	-4.177546	3.00	0.0250575
219467_at	54826	0.93	-3.799928	3.44	0.0250617
212777_at	6654	0.96	-3.520469	3.94	0.0250807
218579_s_at	60625	0.90	-3.611082	3.75	0.025092
209124_at	4615	1.04	5.653585	2.16	0.0250981
227349_at	3070	0.89	-3.639889	3.70	0.0250995
229384_at	NA	0.93	-4.770708	2.55	0.0251371
218553_s_at	79047	1.03	3.83238	3.39	0.0251432
205555_s_at	4488	0.92	-3.683934	3.62	0.0251465
221058_s_at	51192	0.92	-5.364246	2.26	0.0251516
226996_at	253558	0.98	-4.251527	2.92	0.0251677
201118_at	5226	1.04	3.558066	3.85	0.0251926
221478_at	665	1.04	3.841084	3.38	0.0251975
218167_at	51321	0.97	-4.766092	2.54	0.0252241
203846_at	22954	0.93	-3.611891	3.74	0.0252405
222414_at	58508	0.91	-3.592269	3.78	0.0252449

223300_s_at	79780	0.92	-5.496863	2.21	0.025254
203353_s_at	4152	0.96	-3.517029	3.93	0.0252623
203054_s_at	6988	0.90	-3.595504	3.77	0.0252821
202910_s_at	976	1.09	4.171612	2.99	0.0252891
1552310_at	123207	0.92	-3.912434	3.27	0.0252896
203427_at	25842	0.97	-4.030969	3.13	0.025304
233647_s_at	81602	0.92	-4.687939	2.59	0.0253104
226502_at	255520	0.97	-6.058441	2.03	0.025332
203997_at	5774	0.93	-3.578444	3.79	0.025352
209487_at	11030	0.94	-5.619173	2.16	0.0253758
226951_at	79074	0.97	-4.996022	2.41	0.0254048
214733_s_at	54432	0.94	-5.723267	2.12	0.0254092
212077_at	800	1.07	3.88151	3.30	0.0254421
202804_at	4363	1.02	4.316192	2.85	0.0254445
215785_s_at	26999	0.96	-3.557027	3.83	0.0254584
225928_at	10490	0.92	-3.551256	3.84	0.0254734
32069_at	9683	0.94	-3.881503	3.30	0.0254741
210284_s_at	23118	0.95	-3.917153	3.25	0.0254847
218721_s_at	54953	0.93	-3.538095	3.86	0.0254851
212235_at	23129	1.13	3.702222	3.56	0.0254993
227754_at	159195	0.91	-3.516451	3.91	0.0255121
218549_s_at	51115	0.98	-3.961053	3.20	0.0255131
209656_s_at	83604	0.96	-3.504204	3.93	0.0255214
203678_at	22909	0.88	-5.308182	2.27	0.0255221
203712_at	9933	0.92	-4.758615	2.53	0.0255324
218064_s_at	26993	0.91	-5.365927	2.25	0.0255395
218820_at	56967	1.22	4.518236	2.69	0.0255517
225260_s_at	64983	0.98	-3.483396	3.97	0.025562
229983_at	166815	0.93	-3.898135	3.27	0.0255631
212054_x_at	23061	0.95	-4.335566	2.82	0.025583
225545_at	29904	1.09	3.995093	3.15	0.0255846
218898_at	79850	1.06	4.432357	2.75	0.0255934
238737_at	127428	0.95	-3.562472	3.80	0.025606
213099_at	23357	0.96	-3.667356	3.61	0.0256077
40560_at	6909	1.16	5.339939	2.25	0.0256543
230320_at	84897	0.90	-3.499676	3.93	0.0256643
204992_s_at	5217	0.97	-3.517609	3.89	0.0256804
224509_s_at	84816	0.92	-3.54043	3.84	0.0256858
211971_s_at	10128	1.02	4.792484	2.51	0.0256869
235099_at	152189	0.92	-5.712636	2.12	0.0256923
219295_s_at	26577	1.16	5.767357	2.10	0.0256924
218626_at	56478	1.05	4.457793	2.72	0.0257332
208898_at	51382	1.05	4.84292	2.48	0.0257345
223556_at	3070	0.96	-3.462847	4.00	0.025772
210849_s_at	27072	0.94	-3.483542	3.95	0.0257998
212450_at	9728	0.97	-3.657981	3.61	0.0258056
222698_s_at	55364	0.95	-5.112622	2.34	0.0258108
223056_s_at	57510	0.96	-4.049596	3.08	0.02583
224165_s_at	64799	0.67	-5.320524	2.25	0.0258414
208639_x_at	10130	1.02	4.043445	3.08	0.0258496
228899_at	NA	1.05	3.525215	3.86	0.0258539
219677_at	80176	1.09	3.477938	3.96	0.0258602

219278_at	9064	1.13	3.585855	3.73	0.0258817
203382_s_at	348	1.13	3.499865	3.90	0.0258999
230547_at	NA	0.80	-3.467219	3.98	0.0259004
200677_at	754	1.01	3.482964	3.94	0.0259058
202109_at	23647	0.96	-4.009832	3.12	0.0259284
225910_at	284019	0.95	-3.58177	3.73	0.0259622
215113_s_at	26168	1.05	3.776659	3.41	0.0259778
201880_at	25820	0.98	-3.580344	3.73	0.0259979
225965_at	80821	1.10	4.587029	2.62	0.0260146
227969_at	400960	0.92	-5.0604	2.36	0.0260179
39248_at	360	1.18	5.101979	2.34	0.0260293
236305_at	317671	0.93	-3.520414	3.85	0.026032
225936_at	163126	0.96	-3.787201	3.39	0.0260347
223117_s_at	55031	0.94	-3.61746	3.66	0.0260397
203234_at	7378	1.11	4.31863	2.81	0.0260404
217752_s_at	55748	0.93	-3.587066	3.71	0.0261102
217852_s_at	55207	0.98	-4.218019	2.89	0.0261351
212907_at	7779	1.11	5.415374	2.20	0.0261388
217957_at	29105	0.98	-3.445708	4.00	0.0261576
223450_s_at	83548	0.95	-3.695141	3.52	0.0261661
223284_at	57106	0.92	-5.008353	2.37	0.0261785
225766_s_at	3842	1.03	4.159773	2.95	0.0261789
225096_at	55352	1.01	3.790612	3.37	0.0261862
241498_at	283254	0.95	-4.057707	3.05	0.0262064
205780_at	638	1.25	5.292106	2.25	0.026221
217968_at	7260	0.90	-5.154089	2.31	0.0262371
208735_s_at	10106	0.95	-4.83881	2.46	0.0262385
219928_s_at	26256	0.92	-3.581492	3.71	0.0262406
228266_s_at	50810	0.96	-3.933733	3.18	0.0262437
211152_s_at	27429	1.05	3.656391	3.58	0.0262449
202511_s_at	9474	0.97	-4.465989	2.69	0.0262499
218508_at	55802	0.94	-5.340077	2.23	0.026254
212436_at	51592	0.96	-3.462881	3.95	0.0262545
32502_at	81544	1.07	3.838927	3.30	0.0262624
1552417_a_at	121441	0.87	-4.045164	3.05	0.0262899
221187_s_at	80199	0.94	-3.439824	4.00	0.0263002
225748_at	84946	0.97	-4.554771	2.62	0.0263125
203947_at	1479	0.96	-3.654951	3.57	0.0263161
211509_s_at	57142	1.02	3.766437	3.40	0.0263292
204873_at	5189	0.91	-3.956203	3.15	0.0263299
209164_s_at	1534	1.08	4.464496	2.69	0.0263338
239058_at	2303	0.90	-3.688159	3.52	0.0263528
206003_at	9662	0.95	-4.601463	2.59	0.0263601
203269_at	8439	1.03	3.447146	3.97	0.02639
212971_at	833	0.97	-5.445967	2.18	0.0264039
218370_s_at	64766	0.95	-3.442835	3.98	0.0264201
214869_x_at	26130	0.96	-3.88011	3.24	0.0264215
214173_x_at	8725	0.96	-3.507424	3.84	0.0264331
227437_at	NA	0.87	-5.150571	2.30	0.0264367
212399_s_at	9686	1.05	4.825395	2.46	0.0264596
204587_at	9016	0.96	-3.440304	3.98	0.026496
223188_at	56006	0.93	-3.532315	3.78	0.0265172

200843_s_at	2058	0.96	-4.873281	2.43	0.0265219
204744_s_at	3376	0.98	-3.471871	3.91	0.0265235
228391_at	285440	0.93	-3.726663	3.44	0.0265325
223320_s_at	23456	1.06	3.72171	3.45	0.0265416
223383_at	84937	0.96	-3.8431	3.28	0.0265487
209787_s_at	10473	0.97	-3.473503	3.90	0.0265776
214449_s_at	23433	1.04	3.490564	3.86	0.0266045
227436_at	56957	0.92	-3.752112	3.40	0.0266114
212704_at	23318	0.96	-4.165102	2.91	0.0266323
201289_at	3491	1.06	3.502559	3.83	0.0266715
201611_s_at	23463	0.95	-3.589942	3.66	0.0266845
207144_s_at	4435	1.09	5.904478	2.03	0.0266953
214440_at	9	0.94	-3.549972	3.73	0.0267033
242019_at	253782	1.16	3.508091	3.81	0.0267145
220734_s_at	80772	0.96	-5.25771	2.24	0.0267773
218609_s_at	318	0.93	-3.638268	3.56	0.0268027
219205_at	63826	0.93	-3.819818	3.29	0.0268523
238585_at	79712	1.07	3.940475	3.14	0.0268585
220223_at	79915	0.90	-4.819529	2.44	0.0268588
224626_at	113829	0.97	-3.41648	4.00	0.0268833
52169_at	92335	0.97	-3.421442	3.99	0.026893
203640_at	10150	0.93	-4.642173	2.54	0.0269036
219120_at	80304	0.93	-3.655489	3.53	0.0269096
219079_at	51167	0.94	-5.038928	2.33	0.0269335
241825_at	129450	0.84	-5.65512	2.09	0.0269442
203512_at	27095	0.96	-4.153228	2.91	0.0269545
204728_s_at	11169	0.91	-3.456701	3.90	0.0269571
226446_at	55502	1.06	4.295351	2.78	0.0269598
203016_s_at	117178	0.97	-3.637804	3.55	0.0269768
209271_at	2186	0.98	-4.147073	2.91	0.0269857
219333_s_at	11132	0.92	-4.71562	2.49	0.0269949
221480_at	3184	1.03	3.725779	3.41	0.0270117
226349_at	121053	0.93	-5.360362	2.19	0.0270127
222682_s_at	10827	0.94	-3.451017	3.91	0.0270207
201553_s_at	3916	1.05	4.321173	2.76	0.0270391
242584_at	84140	0.95	-3.415997	3.98	0.0270755
232341_x_at	22927	1.07	3.565594	3.67	0.0271149
225656_at	114327	0.93	-4.700901	2.50	0.0271186
218972_at	55761	0.95	-3.408198	3.99	0.0271579
201954_at	10095	1.11	3.626415	3.55	0.0272209
221712_s_at	54663	1.06	5.758373	2.05	0.0272528
205271_s_at	23552	0.93	-3.408606	3.98	0.0272572
200957_s_at	6749	0.96	-4.208015	2.84	0.0272605
209737_at	9863	0.89	-4.287109	2.77	0.0272625
203740_at	10200	0.98	-3.401978	4.00	0.0272725
220731_s_at	55707	0.95	-3.754887	3.35	0.0272797
218009_s_at	9055	0.98	-3.485234	3.81	0.0272809
222754_at	51095	0.97	-3.415189	3.96	0.027288
213127_s_at	112950	0.91	-4.273178	2.78	0.0273172
238599_at	134728	0.89	-3.721212	3.39	0.0273787
216226_at	6875	1.06	3.548199	3.67	0.0274349
206752_s_at	1677	1.07	3.394786	4.00	0.0274353

202762_at	9475	0.95	-3.530783	3.71	0.027456
206364_at	9928	0.96	-3.399782	3.98	0.0274773
226866_at	114799	0.95	-5.036278	2.31	0.0274972
225259_at	51560	1.07	5.542934	2.11	0.0275105
208744_x_at	10808	1.03	3.629786	3.52	0.0275141
229198_at	57558	0.90	-4.351803	2.71	0.0275194
210605_s_at	4240	1.06	3.433012	3.90	0.0275226
201964_at	23064	0.96	-3.527737	3.71	0.0275241
214039_s_at	55353	1.02	3.575754	3.61	0.0275621
229273_at	6299	0.94	-3.432178	3.90	0.0275649
212100_s_at	84271	0.99	-3.896197	3.14	0.0275676
221250_s_at	83463	1.05	3.492803	3.77	0.0275737
209393_s_at	9470	0.98	-4.984955	2.33	0.0275815
223154_at	65008	0.96	-4.340074	2.72	0.0275868
219675_s_at	80146	0.95	-4.246668	2.79	0.0275952
209340_at	6675	1.04	4.781785	2.43	0.0276209
220587_s_at	64223	1.02	4.104373	2.92	0.0276236
212148_at	5087	0.93	-4.16428	2.86	0.0276431
224756_s_at	7920	0.92	-4.308932	2.74	0.0276588
1552381_at	135295	0.89	-3.885434	3.15	0.0276927
212050_at	147179	0.95	-3.415374	3.92	0.0277216
238422_at	151534	0.92	-3.411322	3.93	0.0277263
204979_s_at	6450	0.91	-3.411624	3.93	0.0277369
201192_s_at	5306	1.05	5.417339	2.14	0.0277755
222728_s_at	79101	0.94	-3.381224	4.00	0.0277881
229849_at	NA	1.14	4.849295	2.39	0.0277908
234464_s_at	146956	0.93	-4.615761	2.52	0.0278125
206613_s_at	9015	0.91	-4.398988	2.66	0.0278151
227020_at	388403	1.08	3.808943	3.24	0.027817
206044_s_at	673	0.93	-4.227753	2.79	0.0278798
209600_s_at	51	1.06	4.399551	2.66	0.0278894
228542_at	57380	0.92	-3.419667	3.90	0.0278968
203837_at	4217	1.05	3.615581	3.52	0.0279379
226945_at	84236	0.95	-3.886687	3.13	0.0279466
204883_s_at	3364	0.94	-3.554946	3.62	0.0279727
206342_x_at	3423	0.95	-4.588571	2.53	0.0279765
238542_at	80328	1.04	4.089357	2.91	0.0279813
209209_s_at	10979	0.96	-3.403898	3.92	0.0280064
204278_s_at	9166	0.93	-4.40175	2.65	0.0280118
203278_s_at	51317	0.94	-3.372696	4.00	0.0280126
230672_at	54882	0.91	-3.395615	3.94	0.0280148
212800_at	10228	1.12	3.528126	3.66	0.0280311
212552_at	3241	1.04	3.476717	3.76	0.0280366
202728_s_at	4052	1.17	4.589023	2.52	0.0280392
213216_at	23252	0.93	-3.370054	4.00	0.0280443
213368_x_at	8541	1.07	3.902687	3.11	0.0280462
230259_at	282969	1.08	5.164345	2.23	0.0280596
222201_s_at	9994	0.94	-4.1342	2.86	0.0280673
202174_s_at	5108	0.97	-3.377883	3.98	0.0280736
224815_at	149951	0.96	-3.4196	3.88	0.0280984
234929_s_at	55812	0.86	-3.470024	3.77	0.0281293
205761_s_at	11062	0.87	-3.7184	3.34	0.0281462

39835_at	6305	1.05	4.489023	2.58	0.0281472
232488_at	85007	0.94	-3.669258	3.41	0.0281594
203241_at	7405	0.90	-4.96226	2.32	0.0281632
235532_at	93183	0.95	-3.602235	3.52	0.028167
224578_at	55920	1.03	3.369052	3.99	0.0281838
225341_at	80298	0.95	-3.36554	4.00	0.028212
228855_at	440388	0.83	-4.139886	2.85	0.0282237
225290_at	55500	0.97	-3.889965	3.11	0.0282282
208923_at	23191	1.03	3.49577	3.71	0.0282522
212945_s_at	23269	0.93	-3.375831	3.97	0.0282677
241703_at	154661	1.05	3.870553	3.13	0.0282717
227162_at	57684	0.91	-4.295697	2.72	0.0282741
239671_at	NA	0.91	-3.419273	3.86	0.0282928
200060_s_at	10921	1.02	5.340716	2.15	0.0282945
212160_at	11260	0.97	-3.513236	3.67	0.0283022
227585_at	84896	0.93	-3.374994	3.96	0.0283243
226112_at	6443	0.96	-4.245864	2.75	0.0283362
209604_s_at	2625	0.92	-3.90963	3.08	0.0283374
243707_at	NA	0.91	-4.803966	2.39	0.0283491
235145_at	51043	1.08	3.541084	3.61	0.028353
201805_at	5571	0.96	-4.522661	2.55	0.0283844
202294_at	10274	0.98	-4.026718	2.95	0.0284134
212008_at	23190	0.97	-3.80294	3.21	0.028427
205433_at	590	0.91	-3.411291	3.87	0.0284349
208643_s_at	7520	0.99	-3.896582	3.09	0.0284423
201987_at	9969	0.97	-3.665113	3.40	0.028462
223437_at	5465	0.94	-3.791356	3.22	0.028498
204085_s_at	1203	0.93	-3.520376	3.64	0.0285098
202200_s_at	6732	1.01	3.371549	3.95	0.028517
222988_s_at	252839	0.97	-4.365826	2.65	0.028523
212024_x_at	2314	1.04	3.78648	3.22	0.0285285
202457_s_at	5530	1.03	5.156828	2.22	0.0285355
218266_s_at	23413	1.08	3.350702	4.00	0.0285518
202616_s_at	4204	0.93	-3.507501	3.66	0.0285568
226270_at	55770	0.92	-3.469615	3.73	0.0285904
240616_at	NA	0.91	-3.527368	3.62	0.0285904
205055_at	3682	0.98	-3.517006	3.64	0.028615
227109_at	120227	0.91	-4.049602	2.91	0.0286377
226736_at	91612	0.91	-3.867342	3.11	0.0286448
212286_at	23253	1.07	4.301289	2.69	0.028658
208370_s_at	1827	1.04	3.693569	3.34	0.0286685
200840_at	3735	0.99	-3.713455	3.31	0.0286957
223236_at	84081	0.96	-3.36923	3.94	0.0287018
226195_at	112752	0.91	-3.409582	3.85	0.028758
212730_at	23336	0.89	-3.703287	3.32	0.028763
227134_at	285521	1.14	5.164738	2.21	0.028769
219515_at	56980	1.10	3.384433	3.90	0.0287852
213988_s_at	6303	1.05	4.006477	2.95	0.0288248
229766_at	353274	0.95	-3.975775	2.98	0.0288261
212423_at	219654	1.06	3.733886	3.27	0.0288306
225775_at	340348	1.07	3.549781	3.56	0.0288457
218761_at	54778	0.95	-4.079945	2.87	0.0288648

203657_s_at	8722	1.12	4.576231	2.49	0.0288906
225352_at	7095	0.97	-4.504595	2.54	0.0289096
231046_at	NA	0.78	-5.267764	2.16	0.0289347
203694_s_at	8449	0.96	-5.323341	2.14	0.0289608
213934_s_at	7571	0.91	-3.364801	3.93	0.0289643
213671_s_at	4141	0.96	-3.499586	3.64	0.0289774
219365_s_at	79012	1.11	4.636748	2.46	0.02898
219703_at	55329	0.89	-4.093029	2.85	0.0289827
229091_s_at	54619	1.10	3.705238	3.30	0.0289847
225409_at	493753	0.93	-4.272373	2.70	0.0290284
209161_at	9128	0.97	-3.335114	3.99	0.0290774
204008_at	10126	0.92	-4.90364	2.31	0.0291688
204531_s_at	672	0.97	-3.482221	3.66	0.029169
226629_at	124935	1.09	3.925625	3.01	0.0291977
202066_at	8500	1.02	3.670897	3.34	0.0292354
218497_s_at	246243	0.96	-4.192318	2.75	0.0292504
203432_at	7112	0.97	-4.026085	2.90	0.0292509
222500_at	51645	0.95	-4.998832	2.26	0.0292571
224714_at	84365	0.97	-3.583051	3.47	0.0292682
224926_at	60412	0.94	-3.406059	3.81	0.0292876
216883_x_at	5147	0.97	-4.947925	2.28	0.0293352
224957_at	6139	1.03	4.212594	2.73	0.0294124
222510_s_at	23609	0.95	-3.443891	3.72	0.0294181
223262_s_at	26127	0.95	-4.440956	2.56	0.0294377
226245_at	284252	0.90	-4.397654	2.59	0.0294378
205171_at	5775	0.96	-4.500451	2.52	0.0294468
229025_s_at	196294	0.98	-3.329058	3.97	0.0294586
218558_s_at	54148	0.98	-3.505063	3.59	0.0294962
222512_at	51667	0.97	-3.33672	3.95	0.0294973
225264_at	57038	0.98	-3.31546	4.00	0.0295336
209210_s_at	10979	0.98	-4.776549	2.36	0.0295537
225456_at	5469	0.95	-3.316223	3.99	0.0295865
223393_s_at	57616	0.93	-4.318328	2.64	0.0295882
203221_at	7088	0.97	-3.759416	3.19	0.0296149
218367_x_at	27005	0.94	-4.771519	2.36	0.0296305
237449_at	221833	0.89	-4.653786	2.42	0.0296674
226817_at	1824	1.05	4.102502	2.81	0.029678
231853_at	51174	0.96	-3.368462	3.86	0.0296806
206308_at	1787	0.92	-3.969424	2.94	0.0297093
202912_at	133	1.05	3.397302	3.79	0.0297163
209462_at	333	0.97	-3.414046	3.75	0.0297922
226815_at	132001	0.86	-3.822908	3.09	0.0298371
225153_at	85476	0.97	-3.475361	3.62	0.029842
205304_s_at	3764	0.90	-3.628701	3.36	0.0298732
218688_at	26007	0.93	-3.309456	3.98	0.0298892
212480_at	23384	0.91	-3.521942	3.53	0.0299044
204319_s_at	6001	1.10	3.378474	3.82	0.0299074
203115_at	2235	1.10	3.763448	3.16	0.0299078
200815_s_at	5048	0.97	-3.864537	3.04	0.0299116
200975_at	5538	0.98	-4.603248	2.44	0.0299211
227424_x_at	84996	0.94	-4.392884	2.57	0.0299254
203082_at	9790	0.96	-3.615648	3.37	0.0299348

203339_at	8604	0.91	-4.006918	2.89	0.0299421
213188_s_at	84864	0.95	-4.909977	2.28	0.0299643
235014_at	147727	0.87	-4.343376	2.60	0.0299736
209285_s_at	23272	0.96	-3.298771	4.00	0.0299805
227176_at	114134	1.09	3.422852	3.72	0.0299813
219129_s_at	79685	0.92	-3.45558	3.65	0.029982
219661_at	64901	0.93	-4.280273	2.65	0.0299945
205880_at	5587	0.90	-5.038263	2.22	0.0299986
205156_s_at	41	1.07	3.300231	3.99	0.0300059
208785_s_at	81631	1.05	3.314893	3.96	0.0300088
212376_s_at	57634	0.92	-3.892632	3.00	0.0300256
214604_at	3237	1.05	5.090181	2.19	0.0300301
228361_at	1870	0.89	-3.402815	3.75	0.0300673
224331_s_at	64979	0.98	-4.460174	2.52	0.0300716
236160_at	9321	0.92	-4.487263	2.50	0.0300731
229615_at	NA	0.88	-4.109834	2.78	0.0300799
223180_s_at	29090	0.94	-3.590108	3.40	0.0300936
218178_s_at	57132	1.03	3.389095	3.78	0.0301168
218709_s_at	51098	0.96	-3.453059	3.64	0.0301543
226909_at	85460	0.92	-3.906037	2.98	0.0301638
204566_at	8493	0.95	-4.475861	2.50	0.0302013
209724_s_at	7541	0.92	-3.31873	3.93	0.0302092
238693_at	80012	0.86	-4.09801	2.79	0.0302333
229160_at	139221	0.93	-3.445339	3.65	0.0302393
214427_at	4839	0.96	-3.389906	3.76	0.0302426
218763_at	53407	1.03	3.581487	3.41	0.0302598
242706_s_at	9439	0.95	-3.376384	3.79	0.03026
229886_at	375444	0.90	-5.080059	2.19	0.0302673
225677_at	55973	0.98	-3.333151	3.89	0.0302935
209018_s_at	65018	1.04	4.078093	2.80	0.0302939
223457_at	26958	0.94	-4.480788	2.49	0.0303118
202982_s_at	10965	0.95	-5.240786	2.13	0.030312
210821_x_at	1058	0.96	-4.178459	2.71	0.0303228
218075_at	8086	0.96	-3.33737	3.87	0.030362
204937_s_at	10782	0.95	-3.334996	3.87	0.0304096
205357_s_at	185	1.23	5.201013	2.14	0.0304304
227224_at	55103	0.95	-3.622063	3.33	0.030455
210036_s_at	3757	1.18	4.053428	2.82	0.0304707
225742_at	4194	0.88	-3.359244	3.81	0.0304741
203186_s_at	6275	1.10	3.585422	3.38	0.030523
201507_at	5201	0.98	-3.896703	2.97	0.0305696
227703_s_at	94121	0.86	-4.336671	2.58	0.03058
217349_s_at	4007	1.10	3.282641	3.99	0.0305929
219128_at	54980	0.95	-5.03534	2.20	0.0306022
217771_at	51280	1.05	3.333794	3.86	0.0306268
206565_x_at	349035	0.85	-4.894595	2.26	0.0306332
212412_at	10611	1.04	3.331624	3.86	0.0306888
209610_s_at	6509	0.89	-3.503259	3.51	0.0306934
226986_at	26100	1.03	3.361309	3.79	0.0307222
218377_s_at	10069	0.91	-3.287384	3.96	0.0307604
217978_s_at	55585	0.97	-5.398828	2.05	0.0307817
227417_at	54996	1.08	3.844223	3.01	0.0308113

205172_x_at	1212	1.04	4.982368	2.21	0.0308187
236577_at	NA	0.89	-3.556928	3.41	0.0308589
218475_at	27037	1.05	3.527154	3.45	0.0308691
218437_s_at	54585	0.98	-3.83885	3.01	0.0309024
239047_at	159091	0.88	-3.441044	3.61	0.0309533
228832_at	90024	1.08	4.929427	2.23	0.0309761
219953_s_at	56672	0.97	-3.807677	3.05	0.0309994
208115_x_at	26098	0.89	-3.263574	4.00	0.0310062
209905_at	3205	0.97	-3.35206	3.78	0.031032
218517_at	79960	0.95	-3.815884	3.03	0.0310518
219344_at	55315	1.11	3.915277	2.92	0.0311241
212752_at	23332	0.97	-3.27562	3.96	0.0311261
218257_s_at	56886	0.95	-3.292609	3.91	0.0311297
218351_at	54951	0.95	-4.697689	2.34	0.0311602
226508_at	80012	0.95	-3.409172	3.65	0.0311692
221760_at	4121	1.06	4.725392	2.32	0.0311824
203866_at	54475	0.90	-4.824398	2.27	0.0311839
203832_at	6636	0.97	-4.59996	2.39	0.0312056
208649_s_at	7415	1.03	3.283513	3.93	0.0312345
226771_at	57198	0.94	-3.274706	3.95	0.0312345
226187_at	1040	0.93	-3.261947	3.98	0.0312587
226861_at	140461	0.95	-3.648981	3.24	0.0312806
209655_s_at	83604	0.96	-3.460442	3.54	0.0312846
218569_s_at	55709	0.93	-3.274884	3.94	0.0312899
212969_x_at	256364	1.08	4.259076	2.61	0.0312944
203867_s_at	54475	0.97	-3.409245	3.64	0.0312951
202391_at	10409	0.93	-4.633883	2.37	0.0312994
218991_at	63897	0.93	-4.120508	2.72	0.0313115
226152_at	145567	0.91	-5.289709	2.08	0.0313327
204887_s_at	10733	0.96	-3.738228	3.11	0.0313749
223209_s_at	55829	0.97	-3.644405	3.24	0.031402
204610_s_at	11007	1.11	4.8652	2.25	0.0314057
226541_at	84085	0.96	-3.261997	3.97	0.0314082
224391_s_at	54414	0.92	-3.2644	3.96	0.0314149
226185_at	1040	0.92	-4.08616	2.74	0.0314202
212606_at	23001	0.95	-3.248142	4.00	0.0314321
203638_s_at	2263	0.96	-3.38072	3.69	0.0314338
234299_s_at	51199	1.07	3.480749	3.50	0.0314466
207812_s_at	26003	0.98	-3.260135	3.97	0.0314601
203015_s_at	117178	0.95	-5.431405	2.02	0.0314633
227107_at	24145	0.95	-3.28258	3.91	0.0314638
202951_at	11329	0.94	-4.107592	2.72	0.0314706
200690_at	3313	0.95	-3.249617	3.99	0.0315542
218639_s_at	79364	0.92	-3.434915	3.57	0.0316029
222607_s_at	22894	0.98	-3.316807	3.82	0.0316178
221203_s_at	55689	0.93	-3.50284	3.44	0.0316303
227429_at	283229	1.08	3.389213	3.66	0.0316325
201953_at	10519	1.03	3.250522	3.98	0.0316367
203607_at	22876	0.95	-3.242306	4.00	0.0316445
224845_s_at	57606	0.97	-3.251374	3.97	0.031649
223022_s_at	51534	0.94	-4.785637	2.27	0.0316847
223098_s_at	282809	0.99	-3.241873	3.99	0.0316921

201912_s_at	2935	1.03	4.520399	2.42	0.0316997
218948_at	55278	0.90	-4.341466	2.53	0.0317442
225139_at	80004	0.95	-3.701843	3.14	0.0317548
225246_at	57620	0.92	-4.383103	2.50	0.0317563
235117_at	494143	0.96	-3.354606	3.72	0.0317903
215236_s_at	8301	0.97	-3.513474	3.41	0.0318037
222857_s_at	27345	1.06	4.6667	2.33	0.0318085
232269_x_at	79006	1.05	4.139152	2.67	0.0318729
227046_at	201266	0.91	-3.232713	4.00	0.0318943
220739_s_at	26505	1.04	3.262448	3.92	0.0318945
203292_s_at	55823	1.06	4.758315	2.28	0.0318999
225296_at	57693	0.94	-4.304969	2.55	0.031924
238441_at	5563	0.96	-3.263577	3.91	0.031969
224946_s_at	84317	1.04	4.218684	2.61	0.0319813
228458_at	441150	0.90	-3.425473	3.56	0.032002
209122_at	123	1.05	5.220118	2.08	0.032013
203445_s_at	10106	0.94	-3.741082	3.07	0.0320385
225925_s_at	84196	0.98	-3.2518	3.94	0.0320661
203291_at	4850	0.94	-3.470861	3.47	0.0320819
223446_s_at	84062	1.05	3.277999	3.87	0.0320829
219714_s_at	55799	1.09	3.938381	2.85	0.0320976
211960_s_at	7879	1.03	3.254515	3.92	0.0321163
204161_s_at	22875	0.92	-3.362379	3.68	0.0321272
211474_s_at	5269	1.08	3.324603	3.76	0.03215
212643_at	93487	0.97	-3.25839	3.91	0.032169
223677_at	83734	0.83	-5.249616	2.06	0.0321756
212928_at	23270	0.93	-3.601665	3.25	0.0321869
204281_at	7004	1.04	3.492909	3.42	0.0322088
206213_at	7480	0.90	-3.241903	3.95	0.0322139
227318_at	NA	1.11	3.223748	3.99	0.0322376
224695_at	55571	0.92	-3.514053	3.38	0.0322467
225228_at	128338	0.96	-3.224435	3.99	0.032247
218594_at	55127	0.97	-3.224138	3.99	0.032272
204720_s_at	9829	1.07	3.535327	3.35	0.0322749
207974_s_at	6500	1.03	3.448202	3.49	0.0323169
232293_at	254251	0.92	-3.591237	3.26	0.0323589
202190_at	1477	0.95	-3.857625	2.92	0.0323888
225154_at	94056	1.02	3.219852	3.99	0.0323896
201188_s_at	3710	1.10	3.991718	2.78	0.0324453
212805_at	23273	1.07	3.256435	3.89	0.0324596
227482_at	57143	0.88	-5.165128	2.09	0.0324597
218312_s_at	65982	0.90	-3.227342	3.96	0.0324725
223570_at	55388	0.95	-3.726235	3.06	0.0324776
201392_s_at	3482	1.14	3.649513	3.16	0.0324998
227117_at	11260	0.95	-3.37479	3.62	0.032504
218277_s_at	79665	0.98	-3.502155	3.39	0.0325236
224768_at	55677	0.96	-3.239003	3.93	0.0325531
209649_at	10254	0.94	-3.260747	3.87	0.0325681
212885_at	10199	0.96	-4.642167	2.32	0.0325779
229018_at	84190	0.93	-3.249125	3.90	0.0326293
225227_at	6498	0.94	-3.219323	3.97	0.032638
218483_s_at	56912	1.02	3.210417	3.99	0.0326715

221761_at	159	1.08	3.713848	3.07	0.0326857
213189_at	84864	0.96	-3.207072	4.00	0.0327256
228380_at	NA	0.92	-4.285632	2.53	0.032727
214752_x_at	2316	1.06	3.596112	3.22	0.0328132
223273_at	84520	0.96	-4.64796	2.30	0.0328265
226158_at	54800	1.06	3.325859	3.70	0.0328265
220937_s_at	27090	1.06	3.216331	3.96	0.0328527
201999_s_at	6993	0.98	-3.713551	3.06	0.0328718
224298_s_at	337867	1.03	3.311458	3.73	0.0328747
209578_s_at	23275	0.93	-3.608801	3.20	0.0329093
212216_at	9581	1.01	3.284626	3.79	0.0329127
201723_s_at	2589	1.02	3.325495	3.69	0.0329446
227395_at	219899	0.95	-3.718177	3.05	0.0329697
221253_s_at	81567	0.98	-4.653143	2.30	0.0329852
228306_at	29097	0.98	-3.659971	3.12	0.0330052
227492_at	4950	0.98	-3.263747	3.83	0.0330075
217884_at	55226	0.96	-3.220607	3.94	0.0330215
202842_s_at	4189	1.06	4.367757	2.46	0.0330434
201891_s_at	567	1.02	3.757528	2.99	0.0330442
218227_at	10101	1.03	3.661318	3.12	0.0330454
201099_at	8239	0.98	-3.203904	3.98	0.0330587
219484_at	29915	0.88	-5.289322	2.02	0.0330671
203022_at	10535	0.97	-4.949291	2.15	0.0330793
222760_at	80139	0.97	-3.490474	3.37	0.0330977
203264_s_at	23229	0.94	-4.915807	2.17	0.0331028
212820_at	23312	0.94	-3.581131	3.22	0.0331334
226481_at	9730	0.95	-3.194273	4.00	0.0331372
224800_at	57590	1.02	3.22215	3.92	0.0331521
212743_at	25898	0.90	-3.859518	2.87	0.0331866
232915_at	54555	1.07	3.441364	3.45	0.0332188
218964_at	10620	0.95	-3.387538	3.54	0.0332457
212542_s_at	55023	0.93	-3.655997	3.11	0.0333274
220171_x_at	55425	1.06	3.242938	3.85	0.0333669
222605_at	55758	0.96	-3.204577	3.95	0.0333845
201266_at	7296	1.05	5.202789	2.05	0.0333939
221530_s_at	79365	1.15	3.678514	3.07	0.0334035
235688_s_at	9618	0.95	-3.245432	3.84	0.0334101
229963_at	340542	0.92	-4.099006	2.64	0.0334317
223480_s_at	57129	0.97	-4.817122	2.20	0.0334756
238565_at	NA	0.92	-3.18213	4.00	0.0334761
204634_at	6787	0.95	-3.741208	2.99	0.0335349
209922_at	8315	0.94	-3.768041	2.96	0.0335409
225147_at	9265	1.04	3.179478	4.00	0.0335526
223368_s_at	28989	0.98	-3.187594	3.98	0.0335643
203385_at	1606	1.07	3.195972	3.95	0.0335708
201768_s_at	9685	0.96	-3.876152	2.84	0.0335743
209014_at	9500	0.97	-3.2362	3.85	0.0336241
237968_at	64225	1.10	3.184414	3.98	0.0336328
217640_x_at	220134	0.91	-3.87706	2.83	0.033633
214061_at	93594	0.96	-3.397118	3.50	0.0336453
213501_at	51	1.04	3.781405	2.94	0.0336654
223293_at	84219	0.87	-3.727623	3.00	0.0336681

224471_s_at	8945	0.96	-3.543021	3.25	0.0336742
203060_s_at	9060	1.05	3.185523	3.97	0.0337379
213911_s_at	3015	1.01	3.403798	3.48	0.033751
49111_at	NA	1.19	5.170083	2.05	0.0337605
212255_s_at	27032	1.03	3.311904	3.66	0.0337737
221591_s_at	54478	1.13	4.262191	2.50	0.0337794
236777_at	221584	0.91	-3.827812	2.88	0.0337839
213407_at	23035	0.94	-4.411946	2.40	0.033794
221481_x_at	3184	0.99	-4.470062	2.37	0.0338091
202923_s_at	2729	1.05	4.898783	2.15	0.0338321
221735_at	57599	0.97	-3.183485	3.96	0.0338483
225297_at	115106	0.98	-3.189718	3.95	0.0338652
211047_x_at	1175	0.98	-4.226176	2.52	0.0338779
204546_at	9764	0.93	-3.503661	3.30	0.0338837
233841_s_at	64426	0.95	-3.170618	4.00	0.0338884
225091_at	85364	0.95	-4.035211	2.67	0.0339064
204216_s_at	79882	0.96	-3.472435	3.35	0.0339082
235112_at	158405	0.94	-3.704296	3.01	0.0339428
218502_s_at	7227	0.90	-3.171444	3.99	0.0339684
226648_at	55662	0.93	-3.330922	3.61	0.0339697
229846_s_at	79109	0.94	-4.393306	2.41	0.0340112
238539_at	84343	0.94	-3.19598	3.91	0.0340555
226331_at	56987	0.95	-3.948635	2.74	0.0340761
224318_s_at	55683	0.95	-3.208596	3.88	0.0340851
201695_s_at	4860	1.03	3.275602	3.72	0.0340874
204920_at	1373	0.93	-3.851021	2.84	0.0340947
202306_at	5436	0.99	-3.212238	3.87	0.0341103
210573_s_at	10623	0.94	-4.607207	2.28	0.0341105
213552_at	26035	0.94	-3.885665	2.80	0.0341223
202022_at	230	1.10	3.169389	3.98	0.0341386
219502_at	55247	0.97	-4.276005	2.47	0.0342725
219179_at	51339	1.16	3.382479	3.48	0.0342735
221227_x_at	51805	0.97	-4.90757	2.13	0.0343051
202249_s_at	50717	0.94	-3.183327	3.93	0.034312
227261_at	11278	0.88	-3.413962	3.42	0.0343189
226568_at	284611	0.96	-3.175285	3.95	0.0343381
209375_at	7508	0.95	-4.206225	2.52	0.0343444
213760_s_at	27309	0.95	-3.314467	3.61	0.0343578
228028_at	150946	1.03	3.582842	3.15	0.0343906
222654_at	54928	1.04	3.306644	3.63	0.0343969
205039_s_at	10320	1.06	3.181864	3.92	0.0344095
226024_at	150684	1.05	4.155428	2.55	0.0344451
204293_at	6448	0.92	-4.746169	2.20	0.0344473
221812_at	54455	0.91	-4.743679	2.20	0.0344503
209798_at	4863	0.98	-3.407191	3.43	0.0344661
230766_at	NA	0.93	-3.694357	3.00	0.0344714
55093_at	54480	0.97	-3.584475	3.14	0.0344795
215001_s_at	2752	1.03	3.221593	3.82	0.034488
226396_at	51	1.09	3.272613	3.69	0.0345153
223169_s_at	58480	0.89	-3.698206	2.99	0.0345175
219303_at	79596	0.95	-3.50396	3.26	0.0345329
47773_at	54455	0.94	-3.850666	2.82	0.0345971

202058_s_at	3836	0.95	-3.147846	4.00	0.0345985
201448_at	7072	0.95	-4.991842	2.09	0.0346098
203941_at	55756	0.95	-3.266664	3.70	0.0346119
225282_at	64744	1.06	3.199166	3.86	0.0346197
219622_at	55647	1.06	4.570514	2.28	0.0346331
222428_s_at	51520	0.98	-3.712717	2.96	0.0346873
225705_at	90799	0.94	-4.587027	2.27	0.0347004
236282_at	NA	0.83	-5.160768	2.02	0.0347009
220935_s_at	55755	0.94	-4.165288	2.54	0.0347123
214017_s_at	9704	0.95	-5.110634	2.04	0.0347154
226166_x_at	27148	0.89	-3.184235	3.89	0.0347206
225399_at	116461	0.96	-3.493936	3.26	0.0347409
213196_at	23361	0.95	-3.50711	3.24	0.0347875
212718_at	10914	1.02	4.525516	2.30	0.0347948
235991_at	200008	0.85	-4.34019	2.41	0.0348378
227814_at	348793	0.90	-3.167297	3.92	0.034875
219037_at	51018	0.97	-3.75385	2.90	0.0349101
212802_s_at	26130	0.96	-3.196147	3.84	0.0349658
206500_s_at	55320	0.94	-3.648077	3.03	0.03497
211475_s_at	573	1.02	3.15183	3.96	0.0349955
205160_at	8800	0.93	-3.397464	3.41	0.0349995
225320_at	90550	0.97	-3.173367	3.90	0.0350071
225085_at	55230	0.93	-3.871287	2.77	0.0350191
209445_x_at	55744	0.97	-3.391856	3.42	0.035023
211755_s_at	515	0.99	-3.295458	3.61	0.035033
225740_x_at	4194	0.95	-3.53899	3.18	0.0350348
208094_s_at	81576	0.91	-4.812464	2.15	0.0350363
32723_at	1477	0.95	-3.328128	3.54	0.0350941
222499_at	51021	0.95	-3.224948	3.76	0.0351019
239225_at	51550	0.97	-4.315019	2.42	0.0351214
235089_at	84961	0.95	-3.159525	3.92	0.0351245
234107_s_at	92675	0.98	-3.290377	3.61	0.0351307
210002_at	2627	1.08	3.397154	3.40	0.0351337
213454_at	1325	0.98	-4.229793	2.47	0.035138
200691_s_at	3313	0.98	-3.489694	3.24	0.0351516
206746_at	631	1.17	3.744624	2.90	0.0351721
201678_s_at	56941	1.01	3.129171	4.00	0.0352138
214830_at	145389	0.93	-3.181184	3.86	0.0352159
204556_s_at	22873	0.93	-3.984374	2.66	0.035222
202939_at	10269	0.98	-3.857068	2.78	0.0352241
221306_at	2850	1.04	3.815679	2.82	0.0352447
201712_s_at	5903	0.97	-3.133959	3.98	0.0352476
203530_s_at	6810	1.05	3.96573	2.67	0.0352766
219547_at	1355	0.97	-3.410011	3.37	0.0352852
223321_s_at	53834	1.07	3.835853	2.80	0.0352895
213887_s_at	5434	1.05	3.814273	2.82	0.0352929
201446_s_at	7072	0.94	-4.462982	2.32	0.0352974
203754_s_at	2972	1.05	3.662509	2.99	0.0353845
230311_s_at	93166	0.94	-3.803693	2.83	0.0354332
209153_s_at	6929	0.94	-4.224739	2.46	0.0354461
228917_at	22869	0.93	-3.38587	3.40	0.0354533
213474_at	154881	0.93	-4.340524	2.39	0.0354639

224173_s_at	51263	0.94	-3.139283	3.95	0.0354738
235644_at	165055	0.93	-4.556282	2.26	0.0354777
209470_s_at	2823	1.24	4.635346	2.22	0.0355101
233919_s_at	22927	1.05	3.17844	3.84	0.0355119
41386_i_at	23135	1.04	4.732181	2.17	0.0355124
203635_at	10311	0.94	-4.933713	2.09	0.0355148
238865_at	132430	0.96	-4.997769	2.06	0.0355196
236515_at	55291	0.95	-5.074116	2.03	0.0355346
218260_at	79016	1.05	4.315148	2.40	0.0355443
209306_s_at	23075	0.96	-4.421736	2.33	0.0355736
218603_at	51696	1.01	3.328168	3.50	0.0355796
231045_x_at	280636	0.97	-3.182745	3.83	0.0355829
202542_s_at	9255	0.95	-4.837268	2.12	0.0355908
232780_s_at	51058	0.91	-3.191889	3.80	0.0355957
203279_at	9695	0.95	-3.474834	3.24	0.0356177
213190_at	91949	0.92	-3.282786	3.59	0.0356322
203117_s_at	9924	0.95	-3.152535	3.90	0.0356325
204768_s_at	2237	0.97	-4.587341	2.24	0.0356512
227630_at	5529	0.93	-3.360817	3.43	0.035677
209674_at	1407	1.05	5.078266	2.03	0.0356777
222460_s_at	80011	0.97	-3.15995	3.88	0.0356879
218134_s_at	55696	0.98	-3.181366	3.82	0.0357169
201471_s_at	8878	0.97	-3.232172	3.70	0.0357268
205956_x_at	29893	0.94	-3.554092	3.12	0.0357274
225605_at	90313	1.04	3.196613	3.78	0.0357764
212396_s_at	23065	1.02	3.124123	3.97	0.0357961
201059_at	2017	1.03	3.132065	3.94	0.035807
215691_x_at	51668	0.97	-3.812868	2.80	0.0358199
212091_s_at	1291	1.09	3.113787	3.99	0.0358359
228812_at	NA	0.96	-3.471029	3.23	0.035843
219208_at	80204	0.91	-3.425286	3.31	0.0358613
209844_at	10481	0.90	-3.236744	3.68	0.0358626
226126_at	93627	0.92	-3.22552	3.70	0.0358649
44669_at	NA	0.95	-3.329206	3.48	0.035876
227172_at	89894	1.10	4.840286	2.12	0.0358816
213329_at	23380	1.06	3.149743	3.89	0.0358866
201112_s_at	1434	0.97	-5.074815	2.02	0.03589
219551_at	55840	1.12	3.454949	3.26	0.0359005
205489_at	1428	1.14	4.845967	2.11	0.0359231
218637_at	55364	0.94	-3.598009	3.05	0.0359295
223548_at	54823	0.94	-3.987861	2.63	0.0359596
218434_s_at	65985	1.05	3.423049	3.31	0.0359732
225484_at	95681	0.90	-4.439682	2.31	0.0360443
204276_at	7084	0.92	-4.205818	2.46	0.036051
202739_s_at	5257	0.95	-3.204	3.74	0.0360593
219154_at	144404	1.04	3.445299	3.26	0.0360944
228497_at	55356	0.89	-3.896159	2.70	0.0360944
220967_s_at	79943	0.92	-3.215677	3.71	0.0360959
218359_at	80023	0.94	-3.189952	3.77	0.036115
212795_at	23325	0.95	-4.433119	2.31	0.0361155
225046_at	389831	1.04	3.446725	3.26	0.0361295
212949_at	23397	0.97	-3.172205	3.81	0.0361569

220484_at	55283	1.02	3.101956	4.00	0.0361852
201084_s_at	9774	0.98	-4.727378	2.16	0.0362008
205078_at	5281	0.96	-3.205342	3.72	0.036217
203324_s_at	858	1.05	3.749003	2.85	0.0362616
214838_at	375035	1.08	3.113827	3.96	0.0362678
219259_at	64218	0.94	-4.159798	2.48	0.036284
218332_at	55859	0.97	-3.105506	3.98	0.0362908
218596_at	54662	0.94	-3.491301	3.18	0.0363008
205327_s_at	92	0.97	-3.442367	3.25	0.0363087
221896_s_at	25994	1.02	4.22249	2.43	0.0363435
209174_s_at	54870	1.02	3.654832	2.95	0.0363519
206492_at	2272	0.88	-3.260213	3.59	0.0363605
222151_s_at	80254	0.90	-4.199889	2.45	0.0364021
215954_s_at	58509	1.06	3.387372	3.34	0.0364113
52159_at	51409	0.95	-3.8052	2.78	0.0364183
217919_s_at	28977	0.98	-3.320193	3.46	0.0364627
209291_at	3400	0.98	-3.094617	3.99	0.0364989
222533_at	51185	0.96	-3.986662	2.61	0.0365288
227274_at	55333	0.97	-3.173412	3.78	0.0365292
220738_s_at	27330	0.94	-3.433575	3.25	0.0365968
227653_at	57570	0.93	-3.887558	2.69	0.036601
228889_at	84837	0.88	-3.833853	2.74	0.0366148
218816_at	55227	1.05	4.565287	2.22	0.0366813
204764_at	2342	0.94	-3.437693	3.24	0.0366827
204779_s_at	3217	0.95	-3.156861	3.81	0.0366949
212393_at	6305	1.06	3.209931	3.68	0.0367007
226700_at	199746	0.90	-4.10077	2.51	0.0367092
204690_at	9482	0.92	-4.811362	2.10	0.0367134
226729_at	57695	0.93	-3.212322	3.67	0.0367249
226310_at	253260	0.92	-3.159711	3.80	0.0367286
201089_at	526	0.96	-3.223258	3.65	0.036756
200853_at	3015	1.02	3.342157	3.40	0.0367571
216267_s_at	11070	1.10	4.85506	2.08	0.0367889
218156_s_at	55720	1.03	3.086543	3.99	0.0368151
228645_at	NA	1.11	3.109209	3.92	0.036857
208632_at	9921	0.96	-3.083037	4.00	0.0368694
201871_s_at	51035	1.06	3.132853	3.86	0.0368811
213473_at	8315	0.91	-4.0025	2.58	0.0368834
210933_s_at	6624	1.09	3.828281	2.73	0.0369008
219105_x_at	23594	0.96	-3.759322	2.81	0.0369012
206066_s_at	5889	0.94	-4.81657	2.10	0.0369017
203038_at	5796	0.97	-3.345613	3.38	0.0369192
225603_s_at	NA	0.93	-4.791763	2.11	0.036935
202763_at	836	1.04	3.55218	3.05	0.0369393
218773_s_at	22921	0.93	-3.511979	3.11	0.0369432
62212_at	79078	0.96	-3.520834	3.10	0.0369905
225625_at	121642	0.98	-3.53311	3.08	0.0369981
222763_s_at	55339	0.97	-4.04122	2.54	0.0370032
209228_x_at	7991	0.96	-3.24655	3.58	0.0370076
218333_at	51009	0.98	-3.947535	2.62	0.0370351
219110_at	54433	0.99	-3.08718	3.97	0.0370479
202433_at	10237	0.97	-3.370209	3.33	0.0370489

213203_at	10302	0.93	-4.204006	2.42	0.0370561
212791_at	127703	0.89	-4.471554	2.26	0.0370808
230493_at	387914	1.17	4.710492	2.14	0.0370988
209946_at	7424	0.91	-3.543189	3.06	0.0371129
33736_at	9399	0.89	-4.322923	2.34	0.0371142
225305_at	123096	1.11	3.907424	2.65	0.0371212
217893_s_at	79647	1.03	3.082447	3.98	0.0371385
222028_at	7596	0.90	-4.016162	2.56	0.0371408
226127_at	221120	0.92	-3.547454	3.05	0.0371546
213074_at	134728	0.96	-3.469331	3.16	0.0371671
213413_at	286749	0.89	-3.259964	3.54	0.0372046
212359_s_at	23053	1.05	3.956298	2.60	0.03723
234307_s_at	26153	1.05	3.366214	3.33	0.0372343
225039_at	6120	0.95	-3.68525	2.87	0.0372356
221504_s_at	51606	1.05	4.948316	2.03	0.0373007
242488_at	NA	0.94	-3.574005	3.01	0.0373367
218795_at	51205	0.91	-3.212281	3.63	0.0373421
212435_at	51592	0.98	-3.084852	3.95	0.0373755
217760_at	54765	0.96	-3.873372	2.67	0.0373959
224872_at	57609	1.04	3.092574	3.93	0.0374093
219932_at	28965	1.36	4.632805	2.16	0.037418
201410_at	55041	0.98	-3.067149	3.99	0.0375033
211250_s_at	6452	1.08	4.807515	2.08	0.0375179
211028_s_at	3795	1.08	4.55196	2.20	0.0375215
206533_at	1138	1.05	3.103884	3.89	0.0375216
203102_s_at	4247	1.03	3.164369	3.73	0.0375224
225213_at	160760	1.02	4.045136	2.52	0.0375689
203100_s_at	9425	0.92	-3.11143	3.86	0.0375983
217789_at	58533	0.97	-3.061808	4.00	0.0376121
228177_at	1387	0.95	-3.194249	3.65	0.0376214
209413_at	8704	0.95	-3.066553	3.98	0.037638
218585_s_at	51514	0.97	-3.182391	3.68	0.0376481
203156_at	11215	0.95	-4.196685	2.40	0.0376874
225444_at	NA	0.91	-4.651123	2.15	0.0376941
209726_at	770	1.08	3.114952	3.84	0.0377129
204966_at	576	1.11	3.166371	3.71	0.0377576
205123_s_at	8577	1.04	3.074224	3.95	0.0377683
214058_at	4610	1.09	3.059601	3.99	0.0378005
211240_x_at	1500	0.97	-3.057991	3.99	0.0378038
227968_at	347862	0.96	-3.067545	3.97	0.037805
218462_at	80135	0.97	-3.511322	3.07	0.0378371
227209_at	1272	0.93	-3.118403	3.82	0.03784
225149_at	55795	0.95	-3.413742	3.21	0.0378639
223197_s_at	56916	0.99	-3.054548	4.00	0.0379187
201227_s_at	4714	0.97	-3.878069	2.64	0.0379542
201707_at	5824	0.97	-3.89864	2.62	0.0379775
226366_at	257218	0.96	-3.050946	4.00	0.0380158
208949_s_at	81625	1.09	3.400942	3.22	0.0380265
211070_x_at	1622	1.03	3.226804	3.55	0.0380797
216088_s_at	5688	1.05	3.465029	3.12	0.0380968
212655_at	23174	0.92	-3.310529	3.38	0.0381288
203018_s_at	117178	0.96	-3.058889	3.96	0.0381469

202250_s_at	50717	0.89	-3.487191	3.08	0.0381703
210964_s_at	8908	0.91	-3.21107	3.57	0.0381992
212022_s_at	4288	0.95	-3.125286	3.78	0.038205
226343_at	54878	0.95	-4.295102	2.32	0.0382088
213526_s_at	55851	0.96	-4.394626	2.26	0.0383003
221983_at	79137	1.06	3.972064	2.55	0.038303
218920_at	54540	0.96	-3.838863	2.67	0.0383251
225344_at	135112	0.97	-3.113214	3.80	0.0383344
205657_at	23498	1.05	4.440641	2.23	0.0383355
212936_at	83989	0.94	-3.207105	3.57	0.0383508
238829_at	80208	1.13	4.370783	2.27	0.0383593
203033_x_at	2271	0.96	-3.125921	3.77	0.0383598
230330_at	8493	0.93	-3.040504	4.00	0.0384129
221562_s_at	23410	0.92	-3.053138	3.96	0.0384143
222606_at	55055	0.96	-3.804059	2.69	0.038422
226517_at	586	0.98	-3.056687	3.95	0.0384348
212901_s_at	23283	0.94	-3.33357	3.31	0.0384838
201930_at	4175	0.98	-3.845373	2.65	0.038484
224760_at	6667	0.97	-3.280218	3.41	0.0385599
225004_at	84336	0.96	-3.670097	2.83	0.0385774
219998_at	29094	0.95	-3.485538	3.06	0.038611
204295_at	6834	0.96	-3.140048	3.71	0.0386338
234728_s_at	60625	0.90	-3.040055	3.98	0.0386374
204303_s_at	9811	1.08	3.29148	3.38	0.0386994
205024_s_at	5888	0.95	-3.593333	2.91	0.0387814
229815_at	NA	0.88	-4.369772	2.26	0.0387875
223982_s_at	50640	1.02	4.03976	2.48	0.0388365
223461_at	51256	0.94	-4.093866	2.43	0.0388685
223593_at	51166	0.98	-3.819471	2.66	0.0389022
212162_at	57498	0.95	-3.640014	2.85	0.0389491
203026_at	9925	0.95	-4.046674	2.47	0.0389564
221570_s_at	29081	0.97	-3.155654	3.65	0.0389938
205895_s_at	9221	0.97	-4.056423	2.46	0.0390041
228666_at	348110	0.98	-3.024834	4.00	0.0390225
218941_at	26190	0.97	-3.139119	3.69	0.0390225
215000_s_at	9637	0.95	-3.203009	3.54	0.0390231
211546_x_at	6622	1.06	3.023676	4.00	0.0390283
207494_s_at	7629	0.93	-3.028252	3.99	0.0390327
242900_at	NA	0.92	-3.285398	3.37	0.0390468
200790_at	4953	0.99	-3.059756	3.89	0.0390616
225886_at	1655	1.03	3.045371	3.93	0.0390834
238996_x_at	226	1.11	3.253456	3.43	0.0391128
223396_at	85025	0.96	-3.135315	3.69	0.0391485
201236_s_at	7832	0.96	-3.266772	3.40	0.0391692
222199_s_at	55909	0.95	-4.508746	2.17	0.0392185
226363_at	10057	1.07	3.126998	3.70	0.0392214
238653_at	9860	0.95	-4.523613	2.16	0.0392801
223312_at	84279	1.10	4.731187	2.07	0.0392813
203387_s_at	9882	1.06	4.12025	2.40	0.0393012
212943_at	9847	0.98	-4.282615	2.29	0.039309
212645_x_at	9577	0.94	-4.389142	2.23	0.0393323
223263_s_at	26127	0.93	-3.933674	2.54	0.0393418

209984_at	23081	1.05	3.050066	3.90	0.0393447
209037_s_at	10938	1.05	3.127893	3.69	0.0393475
211935_at	23204	1.02	4.539907	2.15	0.0393678
222558_at	55197	0.98	-3.120726	3.71	0.0393736
212934_at	137886	0.96	-3.086513	3.79	0.039388
207891_s_at	11219	0.96	-4.399445	2.22	0.0394271
213326_at	6843	1.07	4.527188	2.16	0.0394959
203250_at	22828	0.97	-3.040149	3.91	0.0395171
222216_s_at	63875	0.95	-3.344427	3.24	0.0395373
223403_s_at	84172	0.97	-3.18357	3.55	0.0395794
227818_at	64793	0.97	-4.566021	2.13	0.0395934
227021_at	221656	0.95	-3.691054	2.76	0.0395997
226095_s_at	146517	0.95	-3.296892	3.32	0.0396087
219694_at	54491	0.97	-3.013427	3.98	0.0396208
226633_at	51762	0.95	-3.183431	3.54	0.0396263
223352_s_at	55028	1.05	3.568329	2.90	0.0396277
224200_s_at	56852	0.92	-3.945562	2.52	0.0396331
203972_s_at	8504	1.03	4.207305	2.33	0.0396509
203667_at	6902	1.02	3.151992	3.61	0.0396595
229925_at	388662	1.12	3.541086	2.93	0.0397019
221788_at	5238	0.95	-3.382444	3.16	0.0397263
228049_x_at	NA	0.94	-3.183461	3.54	0.03973
218176_at	64110	0.96	-4.036908	2.44	0.0398012
225112_at	10152	0.97	-3.017687	3.96	0.0398157
218219_s_at	55915	1.09	4.738711	2.05	0.039835
226221_at	57589	0.92	-3.627345	2.82	0.039843
225361_x_at	159090	0.94	-3.00225	4.00	0.0398545
219526_at	79697	0.93	-3.043038	3.88	0.0398898
201628_s_at	10670	0.98	-3.33511	3.23	0.0398913
224446_at	84298	0.97	-3.420681	3.09	0.0399344
39249_at	360	1.07	3.333784	3.23	0.0399412
225332_at	11339	0.94	-3.867403	2.58	0.0399457
232184_at	57679	0.95	-2.999296	4.00	0.0399774
205063_at	8487	0.94	-4.111311	2.38	0.0399985
217761_at	55256	1.03	3.21859	3.44	0.0400331
239538_at	84083	0.96	-3.362941	3.18	0.0400502
229887_at	259173	1.04	3.060424	3.81	0.0400892
216231_s_at	567	1.02	4.586666	2.11	0.040099
215884_s_at	29978	0.97	-3.226444	3.42	0.0401377
226609_at	285761	1.05	3.265044	3.34	0.0401476
223497_at	57579	0.96	-3.675333	2.75	0.0402044
217437_s_at	6867	1.09	4.457251	2.17	0.0402156
218338_at	1911	0.91	-3.050203	3.83	0.0402188
219216_at	54465	0.91	-3.883391	2.55	0.0402471
204918_s_at	4300	1.14	3.580215	2.86	0.0402796
205427_at	6940	0.95	-3.067786	3.77	0.0403653
221135_s_at	28990	0.85	-4.409174	2.19	0.0404013
225994_at	53981	0.96	-3.353129	3.17	0.0404095
224458_at	84302	1.08	3.185094	3.49	0.0404098
212509_s_at	439921	1.03	3.692178	2.73	0.0404129
204365_s_at	65055	1.07	4.806526	2.01	0.0404148
53076_at	11285	0.96	-3.132608	3.61	0.0404154

236347_at	166785	0.91	-3.462388	3.01	0.0404492
218352_at	55213	0.95	-3.445332	3.03	0.0405183
226744_at	79066	0.96	-3.005824	3.94	0.0405379
48580_at	30827	1.03	3.552713	2.88	0.0405775
216095_x_at	8776	1.02	3.007728	3.93	0.0405834
235256_s_at	130589	0.96	-3.068039	3.76	0.0405839
201478_s_at	1736	0.97	-3.373876	3.13	0.0405897
221050_s_at	54676	0.95	-3.53254	2.90	0.040617
203992_s_at	7403	0.96	-3.445316	3.02	0.0406242
201663_s_at	10051	0.97	-3.071623	3.74	0.0406739
200803_s_at	7009	1.05	4.688032	2.05	0.0406838
225485_at	95681	0.89	-3.673067	2.74	0.0406919
203599_s_at	11193	0.96	-3.208207	3.42	0.0407233
204269_at	11040	1.10	3.960444	2.47	0.0408023
229592_at	6345	0.90	-4.005654	2.43	0.0408692
209318_x_at	5325	1.06	3.02983	3.84	0.0408898
213992_at	1288	0.94	-4.409943	2.18	0.0408903
223027_at	51429	0.97	-3.087852	3.68	0.0409322
232549_at	54033	0.87	-4.463833	2.15	0.0409587
223594_at	84216	1.11	4.507383	2.12	0.0409669
218383_at	54930	0.94	-3.479449	2.96	0.0409963
203885_at	23011	1.02	3.195317	3.43	0.0410142
244052_at	84869	0.94	-3.37793	3.10	0.0410406
204190_at	10208	0.93	-4.324785	2.22	0.0410517
231299_at	116988	1.18	3.821556	2.58	0.0410656
227791_at	285195	0.95	-3.092916	3.66	0.0411289
222457_s_at	51474	1.07	3.01938	3.85	0.0411372
223735_at	84100	0.91	-4.558094	2.09	0.0411548
200030_s_at	5250	1.01	2.970402	4.00	0.0411749
203175_at	391	0.95	-3.122493	3.58	0.0411825
200759_x_at	4779	0.94	-3.030266	3.82	0.0411938
228769_at	342945	0.95	-3.194467	3.42	0.0412246
218827_s_at	55125	0.96	-3.492025	2.93	0.0412324
218235_s_at	51118	0.97	-4.040809	2.39	0.0412408
228260_at	1993	0.93	-3.256273	3.30	0.0412412
223008_s_at	23731	0.98	-3.144311	3.53	0.0412771
219740_at	79805	1.15	3.823592	2.57	0.0412929
219109_at	79582	0.95	-2.975601	3.97	0.0413171
226354_at	114294	1.05	3.248624	3.31	0.041356
226426_at	23394	0.93	-4.113309	2.34	0.041389
223302_s_at	79027	0.91	-3.37504	3.09	0.0414202
205061_s_at	5393	0.95	-3.590021	2.80	0.0414266
238494_at	26146	0.98	-3.038753	3.78	0.0414334
218713_at	79664	0.96	-3.307142	3.20	0.0414594
200766_at	1509	1.07	4.525844	2.10	0.0414886
208165_s_at	10279	0.92	-3.202989	3.39	0.0414972
218471_s_at	582	0.94	-3.008428	3.86	0.0415005
229175_at	114826	0.95	-2.977395	3.95	0.0415021
226131_s_at	6217	1.00	4.308516	2.21	0.0415033
209303_at	4724	0.94	-3.023683	3.81	0.0415055
205631_at	9786	0.95	-3.784531	2.59	0.0415193
226283_at	282809	1.06	3.879028	2.51	0.0415409

55692_at	63916	0.98	-3.493897	2.91	0.0415505
201264_at	11316	1.04	3.050863	3.73	0.0415785
231976_at	55180	0.77	-4.557104	2.08	0.0415991
203323_at	858	1.05	3.099943	3.61	0.041631
224364_s_at	53938	1.03	3.542825	2.85	0.0416377
225196_s_at	64949	1.02	2.987878	3.91	0.0416652
226124_at	146198	0.91	-4.113134	2.33	0.0416725
201677_at	56941	1.06	3.070327	3.68	0.0416857
222408_s_at	51646	1.03	3.184796	3.41	0.0416993
201988_s_at	1389	0.93	-3.206545	3.37	0.0417002
202427_s_at	25874	0.98	-3.898725	2.48	0.0417467
200829_x_at	7756	0.99	-3.238218	3.30	0.0417626
230243_at	93587	0.93	-3.075141	3.66	0.041784
203019_x_at	117178	0.94	-3.112046	3.57	0.0417897
224797_at	57561	0.87	-3.433081	2.98	0.0418124
222426_at	79109	0.94	-3.027069	3.78	0.0418236
227933_at	84894	1.08	4.705834	2.01	0.0418318
225848_at	155061	0.92	-3.567241	2.81	0.0418384
213902_at	427	0.97	-2.968741	3.95	0.0418457
238644_at	114803	0.87	-2.995717	3.87	0.0418465
219345_at	51027	0.94	-3.067824	3.67	0.0418757
204030_s_at	29970	1.03	2.956587	3.99	0.0418785
228503_at	NA	0.91	-3.152197	3.47	0.0418955
220278_at	55693	0.80	-4.078406	2.34	0.0419071
213385_at	1124	0.93	-3.504697	2.88	0.0419893
226320_at	10189	1.03	3.127419	3.52	0.0420056
209647_s_at	9655	0.95	-2.97265	3.93	0.0420074
203431_s_at	9743	0.92	-3.186231	3.39	0.0420114
240677_at	84872	0.85	-3.013716	3.81	0.0420127
213989_x_at	54093	0.96	-3.613285	2.75	0.042044
218378_s_at	79706	0.97	-2.948312	4.00	0.0420577
223774_at	85028	1.07	3.430677	2.97	0.0420782
235054_at	131870	0.87	-3.148032	3.47	0.0420976
209215_at	10227	1.06	3.562866	2.80	0.0420994
209620_s_at	22	0.96	-3.065362	3.66	0.0421093
213738_s_at	498	0.98	-3.786431	2.57	0.0421169
213367_at	155060	1.04	2.948555	3.99	0.0421263
228494_at	55607	1.09	2.956402	3.97	0.042134
221268_s_at	81537	1.06	3.555723	2.81	0.0421433
201207_at	7126	0.96	-4.382217	2.15	0.0421588
242064_at	54549	0.95	-3.891356	2.48	0.042173
219979_s_at	51501	0.91	-4.029169	2.37	0.0421918
218001_at	51116	1.03	3.263325	3.23	0.0422503
225606_at	10018	1.02	2.998758	3.83	0.0422618
218316_at	26520	0.96	-3.926628	2.44	0.0422697
241803_s_at	401522	0.86	-3.270079	3.22	0.0422946
228332_s_at	280636	0.98	-3.063992	3.65	0.0422968
232001_at	439949	0.94	-3.137974	3.48	0.0422988
204788_s_at	5498	0.95	-2.942984	3.99	0.0423324
227307_at	90139	0.89	-2.995244	3.83	0.0423865
212811_x_at	6509	0.91	-2.98201	3.87	0.042392
219617_at	79823	0.96	-2.942298	3.99	0.0424548

231855_at	57650	0.95	-3.14811	3.45	0.0424671
218520_at	29110	0.99	-3.324372	3.12	0.0424792
201943_s_at	1362	1.03	3.497559	2.87	0.0424978
238010_at	339448	0.92	-4.149875	2.28	0.0425406
222276_at	55798	0.88	-3.257035	3.23	0.042553
209990_s_at	9568	1.36	4.652006	2.02	0.0425538
203763_at	51626	0.94	-3.602585	2.74	0.0425591
206583_at	55634	0.92	-3.157731	3.42	0.0425687
201607_at	11137	0.95	-3.402213	2.99	0.0425955
209867_s_at	23284	1.09	3.725776	2.61	0.0426107
212021_s_at	4288	0.94	-3.134881	3.46	0.0426345
222400_s_at	55256	1.03	3.60757	2.73	0.0426723
222759_at	51111	0.88	-3.520891	2.83	0.0426946
209085_x_at	5981	0.97	-3.060405	3.64	0.0426962
209962_at	2057	1.15	3.7142	2.62	0.0427104
205598_at	10293	0.92	-2.93328	4.00	0.0427157
223526_at	83608	0.96	-2.998013	3.80	0.0427378
202130_at	8780	0.95	-3.349637	3.06	0.0427608
209158_s_at	9266	0.98	-2.96554	3.89	0.042776
218976_at	56521	1.04	2.936287	3.98	0.0427887
200915_x_at	3895	0.98	-3.218731	3.28	0.0427949
224600_at	8545	0.99	-3.059922	3.63	0.0428096
225029_at	550643	0.98	-2.93166	3.99	0.0428165
240572_s_at	374443	0.88	-2.929258	4.00	0.0428652
205443_at	6617	0.97	-3.148107	3.42	0.0428936
228274_at	113675	0.92	-3.541421	2.79	0.0429326
219097_x_at	79086	0.97	-3.974195	2.39	0.0429379
230508_at	27122	1.18	3.003144	3.77	0.0429394
203187_at	1793	0.89	-3.043709	3.66	0.0429424
222415_at	58508	0.95	-3.729659	2.59	0.0429699
241447_at	84289	0.92	-3.4251	2.94	0.0430267
201057_s_at	2804	0.97	-3.007449	3.75	0.0430275
229954_at	55349	1.14	4.245231	2.20	0.0430868
244362_at	NA	0.80	-4.515209	2.06	0.0431069
222387_s_at	55737	1.07	4.438803	2.10	0.0431162
205803_s_at	7220	0.89	-4.573668	2.04	0.043131
239319_at	NA	0.91	-3.546992	2.78	0.0431356
202494_at	10450	0.97	-3.198699	3.30	0.0431541
204921_at	2622	0.96	-4.475511	2.08	0.0431567
229605_at	200008	0.84	-4.288456	2.18	0.0431967
217882_at	55831	1.01	3.708557	2.60	0.0432009
204009_s_at	3265	0.96	-2.934919	3.95	0.0432091
203517_at	10651	0.96	-3.896764	2.44	0.0432155
222890_at	29070	0.82	-3.404822	2.96	0.0432165
212858_at	124222	0.95	-3.107404	3.49	0.0432218
232014_at	90075	0.87	-3.020295	3.70	0.0433196
221736_at	57148	0.93	-2.920776	3.99	0.0433213
205120_s_at	6443	0.96	-3.027138	3.68	0.0433238
215867_x_at	771	1.03	3.326321	3.07	0.0433305
227179_at	27067	0.94	-4.602639	2.02	0.0433756
226312_at	253260	0.97	-2.927936	3.96	0.0433873
228089_x_at	374395	0.97	-2.934282	3.94	0.043475

224586_x_at	10923	1.04	4.619164	2.01	0.0435021
208724_s_at	5861	1.02	3.783421	2.52	0.0435056
206261_at	8187	0.91	-2.918121	3.98	0.0435341
224320_s_at	84515	0.92	-3.569301	2.74	0.0435409
212717_at	9842	1.03	2.916533	3.99	0.0435511
225073_at	51535	1.02	3.553618	2.75	0.0435969
215731_s_at	10198	0.95	-3.100252	3.48	0.0435969
205575_at	10882	1.08	3.679366	2.62	0.0436086
217778_at	27173	0.95	-2.941068	3.90	0.0436487
212662_at	5817	0.93	-3.011956	3.70	0.0436859
230533_at	23613	1.08	4.571409	2.02	0.0436964
225063_at	84993	0.96	-3.214521	3.24	0.0437136
221940_at	27079	0.91	-4.154835	2.24	0.0437322
203955_at	9858	0.95	-3.20061	3.27	0.0437962
204160_s_at	22875	0.94	-3.720043	2.57	0.0438263
209709_s_at	3161	0.97	-3.186723	3.29	0.0438472
219152_at	50512	1.14	3.157828	3.34	0.043901
201097_s_at	378	1.01	2.907053	3.99	0.043908
229370_at	NA	1.12	3.735315	2.55	0.043921
218025_s_at	10455	0.98	-3.075916	3.52	0.0439312
225261_x_at	51497	0.97	-2.903212	4.00	0.043985
202428_x_at	1622	1.04	3.60451	2.68	0.0440035
228002_at	91734	0.94	-3.294413	3.09	0.0440269
218478_s_at	55596	0.95	-3.040342	3.60	0.0440442
221031_s_at	81575	0.91	-3.017489	3.66	0.0440545
205303_at	3764	0.93	-4.239239	2.18	0.044115
222524_s_at	140459	1.09	3.477372	2.82	0.0441207
225865_x_at	51497	0.97	-3.147405	3.35	0.0441214
209250_at	8560	1.03	4.520087	2.04	0.0441403
209773_s_at	6241	1.04	3.297555	3.08	0.0441828
204761_at	9712	0.91	-4.235786	2.18	0.0441952
47530_at	51531	1.05	3.835867	2.45	0.0442043
234924_s_at	57592	0.94	-2.980782	3.75	0.0442124
218796_at	55612	1.13	3.708317	2.57	0.0442169
206745_at	3227	0.96	-3.246555	3.16	0.0442307
203210_s_at	5985	0.98	-3.068488	3.52	0.0442358
203243_s_at	10611	1.07	3.294904	3.08	0.0442584
213822_s_at	89910	0.96	-2.909862	3.96	0.0442687
223656_s_at	56063	0.95	-3.599127	2.67	0.0442755
210131_x_at	6391	1.02	2.960424	3.79	0.044361
224721_at	84128	0.95	-4.042619	2.29	0.0443879
235334_at	256435	0.90	-2.899172	3.98	0.0443937
234192_s_at	80318	0.97	-2.971375	3.76	0.0443942
228751_at	57396	0.92	-3.476293	2.81	0.0444443
200648_s_at	2752	1.05	3.020094	3.62	0.0444714
202758_s_at	8625	0.95	-3.921162	2.37	0.0445661
236502_at	NA	0.80	-3.7402	2.52	0.0445785
220235_s_at	55791	0.95	-2.922241	3.89	0.0446014
213400_s_at	6907	0.97	-2.990989	3.69	0.0446207
226787_at	7566	0.93	-3.079226	3.47	0.0446475
218181_s_at	9448	0.95	-3.011869	3.63	0.0446481
225258_at	54751	0.96	-3.127118	3.36	0.0446745

209820_s_at	10607	1.04	2.888417	4.00	0.0446889
204256_at	79071	0.96	-4.542977	2.01	0.0446967
209358_at	6882	1.03	2.893376	3.98	0.0447236
209781_s_at	10656	1.04	3.577383	2.68	0.0447377
222182_s_at	4848	1.02	2.904586	3.94	0.0447588
226319_s_at	10189	1.05	3.346072	2.97	0.0447671
226269_at	54332	0.89	-3.210961	3.19	0.0447931
227790_at	90025	0.95	-2.885426	4.00	0.0447968
223557_s_at	23671	1.12	4.17334	2.20	0.0448005
205512_s_at	9131	0.97	-2.885089	4.00	0.0448096
230329_s_at	11162	0.95	-3.832381	2.44	0.0448275
203598_s_at	11193	0.95	-3.145649	3.32	0.0448356
215684_s_at	84164	0.92	-3.87461	2.40	0.0448358
205125_at	5333	1.06	3.043153	3.54	0.0448642
216897_s_at	199870	0.95	-2.887265	3.99	0.0448744
226519_s_at	85007	0.96	-3.483747	2.78	0.0448759
221479_s_at	665	1.07	3.193414	3.22	0.0449005
206037_at	883	1.08	3.632086	2.62	0.0449125
218830_at	51121	0.97	-3.42836	2.85	0.0449402
1552486_s_at	114294	1.09	3.212755	3.18	0.0449752
228255_at	65062	0.95	-3.189764	3.22	0.0450037
214168_s_at	7082	0.94	-3.006677	3.63	0.0450047
226038_at	91694	1.08	4.100412	2.24	0.0450273
215215_s_at	81691	0.92	-3.366131	2.93	0.0450488
222396_at	51155	1.03	2.880786	3.99	0.0450492
225230_at	128338	0.97	-4.081633	2.25	0.0450582
225732_at	57542	1.04	4.192372	2.18	0.0450717
220140_s_at	29916	0.91	-3.568942	2.68	0.0450892
223256_at	55632	0.93	-2.879073	4.00	0.0451027
207015_s_at	8854	1.10	2.945277	3.78	0.0451739
218823_s_at	54793	1.04	2.911057	3.89	0.0451899
209440_at	5634	0.98	-2.879005	3.99	0.0452024
200711_s_at	6500	1.02	3.127849	3.33	0.0452369
218023_s_at	51307	0.95	-2.884961	3.97	0.0452499
221909_at	84900	0.92	-3.850808	2.41	0.0452711
225890_at	92667	0.97	-3.373134	2.91	0.0453123
223694_at	81786	1.14	4.5148	2.01	0.0453321
222869_s_at	55520	0.91	-3.101463	3.38	0.0453493
207232_s_at	9666	0.89	-3.139526	3.30	0.0453526
201609_x_at	23463	0.97	-3.248783	3.10	0.0453745
221792_at	51560	1.03	3.795527	2.45	0.0453753
212201_at	23141	0.96	-3.206569	3.16	0.0455612
209804_at	9937	0.94	-3.15574	3.26	0.0455743
218884_s_at	60558	0.92	-3.773973	2.46	0.0456102
200020_at	23435	1.02	2.878847	3.95	0.0456811
205618_at	5638	0.95	-2.885621	3.93	0.045745
205851_at	10201	0.97	-2.889318	3.92	0.045756
204264_at	1376	0.91	-2.944436	3.75	0.0457579
227158_at	112487	0.96	-3.822769	2.41	0.0458336
212051_at	147179	0.95	-4.195918	2.16	0.0458586
222794_x_at	55149	0.94	-3.270626	3.04	0.0459192
212424_at	22984	0.95	-3.368444	2.89	0.0459484

220685_at	54954	0.90	-3.904265	2.34	0.0459584
212501_at	1051	0.95	-3.586998	2.62	0.0459628
209645_s_at	219	0.98	-3.317431	2.96	0.0460216
201773_at	23394	0.97	-3.197189	3.16	0.0460633
227986_at	79175	0.90	-3.077054	3.39	0.0460722
203179_at	2592	1.07	3.42905	2.80	0.0460909
243829_at	673	0.94	-3.445085	2.78	0.0460939
209379_s_at	54462	0.95	-2.906967	3.84	0.0461004
221577_x_at	9518	0.91	-4.283561	2.10	0.0461125
222566_at	51111	0.93	-3.184933	3.18	0.0461279
228944_at	257	0.94	-2.9101	3.82	0.0461593
211855_s_at	9016	0.97	-2.994911	3.58	0.0461694
219826_at	79744	0.89	-2.974658	3.64	0.0461788
202955_s_at	10565	0.94	-3.020137	3.52	0.0461842
200045_at	23	0.96	-3.422665	2.81	0.0461993
203633_at	1374	1.09	3.184913	3.17	0.0462188
45633_at	64785	0.95	-4.075478	2.22	0.0462358
204709_s_at	9493	0.97	-3.050929	3.44	0.0462689
223527_s_at	81602	0.90	-4.16184	2.17	0.0462733
211987_at	7155	0.98	-3.114983	3.30	0.0462754
221882_s_at	58986	1.02	3.258098	3.04	0.0463152
212442_s_at	253782	1.04	3.620695	2.58	0.0463201
238803_at	143279	0.90	-3.359744	2.88	0.0463997
236236_at	NA	0.92	-2.978549	3.61	0.0464017
235968_at	116987	0.94	-3.273889	3.01	0.046413
203882_at	10379	0.90	-3.393303	2.84	0.0464355
211754_s_at	10478	1.04	4.302216	2.08	0.0464619
227298_at	401264	0.87	-4.452538	2.01	0.0464625
204558_at	8438	0.96	-3.970505	2.28	0.0464747
203928_x_at	4137	1.08	3.00295	3.54	0.046485
202218_s_at	9415	1.07	3.036843	3.46	0.0464928
227456_s_at	221545	0.93	-3.643484	2.55	0.046501
201219_at	1488	0.97	-2.847479	4.00	0.0465503
203853_s_at	9846	1.11	3.988545	2.27	0.0465556
210994_x_at	373	0.90	-4.237994	2.12	0.0465592
209430_at	9044	0.96	-3.284243	2.99	0.046561
218494_s_at	56731	0.95	-2.968243	3.63	0.0465679
219490_s_at	64858	0.94	-3.133867	3.25	0.0465785
226594_at	51004	0.92	-2.856408	3.96	0.0466011
202398_at	10239	0.90	-2.921057	3.76	0.0466321
242844_at	5229	0.90	-3.029679	3.47	0.0466891
227063_at	254863	0.97	-2.844706	4.00	0.0466962
241360_at	NA	0.89	-2.880246	3.87	0.0467759
225310_at	27316	0.94	-3.748941	2.44	0.0467766
217007_s_at	8751	1.09	3.190165	3.13	0.0468028
220956_s_at	112398	0.93	-3.149234	3.20	0.0468728
209090_s_at	51100	0.96	-2.84489	3.98	0.0468793
236275_at	84626	0.99	-2.899314	3.80	0.0469112
228622_s_at	3338	1.04	2.878868	3.87	0.0469266
209091_s_at	51100	0.98	-2.946677	3.66	0.0469435
226713_at	152137	0.95	-3.045505	3.42	0.0469673
224015_s_at	64432	0.92	-3.624254	2.55	0.047011

225236_at	92400	0.91	-4.24388	2.10	0.0470258
212456_at	23277	1.04	2.890634	3.82	0.0470277
209455_at	23291	0.98	-2.845367	3.97	0.0470395
215695_s_at	8908	0.91	-3.01496	3.48	0.0470465
214288_s_at	5689	0.98	-3.137209	3.22	0.0470635
226793_at	283267	0.97	-2.842255	3.98	0.0470795
237400_at	27109	1.11	3.499941	2.68	0.0470983
202061_s_at	6400	0.98	-3.665392	2.51	0.0471522
226019_at	115209	0.94	-3.094613	3.30	0.04716
227008_at	374659	0.94	-3.814718	2.38	0.0471875
227924_at	NA	0.96	-3.201846	3.09	0.0472246
225152_at	90441	1.03	3.64837	2.52	0.047233
201364_s_at	4947	0.96	-3.265396	2.98	0.0473057
225176_at	NA	1.09	3.138979	3.20	0.0473341
209084_s_at	9364	0.94	-3.66331	2.50	0.0473674
223534_s_at	83694	0.91	-3.089997	3.30	0.0474033
227151_at	257364	1.08	3.597704	2.56	0.0474294
200892_s_at	6434	0.98	-2.858008	3.90	0.0474835
225161_at	85476	0.97	-2.991853	3.51	0.0475152
242305_at	NA	0.93	-3.35038	2.85	0.0475216
221878_at	388969	0.91	-2.855458	3.90	0.0475259
212447_at	25948	0.95	-3.628664	2.53	0.0476169
218755_at	10112	0.98	-2.824877	4.00	0.0476374
206416_at	7755	0.95	-2.831508	3.97	0.0476561
241434_at	3652	0.92	-2.997857	3.49	0.0476786
222600_s_at	55236	0.95	-2.854513	3.89	0.0477016
203386_at	9882	1.06	3.380739	2.80	0.0477045
218124_at	54884	1.06	3.834136	2.35	0.0477196
50374_at	339229	0.95	-3.460671	2.70	0.0477571
204254_s_at	7421	1.12	2.823402	3.99	0.04776
209615_s_at	5058	0.94	-2.970837	3.55	0.0477679
222390_at	51322	0.98	-2.942017	3.63	0.0477787
227421_at	54059	0.93	-3.536866	2.61	0.0477956
219621_at	63967	0.89	-4.056572	2.19	0.0478123
225651_at	7325	0.98	-3.426998	2.74	0.0478325
1552426_a_at	80213	1.04	4.02608	2.21	0.0478514
222808_at	55849	1.04	2.858161	3.87	0.04786
212068_s_at	84726	0.96	-3.455974	2.70	0.0478622
201346_at	79602	0.98	-3.744847	2.41	0.0478641
217908_s_at	55827	0.98	-3.759661	2.40	0.0478826
212803_at	4665	0.95	-2.851833	3.88	0.0479406
203391_at	2286	1.05	3.047813	3.36	0.0479476
226557_at	91875	0.95	-3.096593	3.25	0.0479557
200018_at	6207	0.99	-2.852112	3.88	0.047982
225536_at	113452	1.09	2.824549	3.97	0.0479837
226770_at	260425	0.95	-2.836897	3.93	0.047991
212390_at	9659	1.13	2.852404	3.88	0.0479968
204669_s_at	11237	1.07	2.952461	3.58	0.0480347
223266_at	55437	0.94	-2.825071	3.97	0.0480568
201422_at	10437	1.04	3.229961	3.01	0.0480582
214202_at	5229	0.92	-3.571682	2.57	0.0480648
201630_s_at	52	0.98	-3.300649	2.90	0.0480662

209858_x_at	65258	0.97	-3.144161	3.15	0.0481109
225876_at	57185	0.94	-2.814922	4.00	0.0481151
221999_at	51231	0.96	-3.014185	3.42	0.0481311
202983_at	6596	0.98	-2.911936	3.68	0.0481835
202779_s_at	27338	1.05	4.240531	2.07	0.0481956
222525_s_at	55246	0.94	-3.320003	2.86	0.0482338
230174_at	127018	0.94	-3.302179	2.89	0.0482421
226625_at	7049	1.05	2.995836	3.46	0.0482887
224742_at	26090	1.03	2.944547	3.59	0.0482954
235085_at	157285	1.04	3.611062	2.52	0.048299
226456_at	116028	0.96	-2.810971	3.99	0.0483485
232038_at	221322	0.88	-2.830899	3.93	0.0483489
203363_s_at	9776	1.04	2.814887	3.98	0.0483656
212379_at	2618	0.96	-3.487969	2.65	0.0483723
218453_s_at	55836	0.90	-4.331581	2.02	0.0483755
226391_at	4708	0.91	-4.106528	2.14	0.0483846
227122_at	NA	0.86	-3.884002	2.29	0.0483867
230079_at	54879	0.92	-3.278866	2.92	0.0483876
211956_s_at	10209	0.99	-2.816506	3.97	0.0484031
230611_at	284612	0.90	-2.827641	3.93	0.048405
44617_at	79676	0.92	-2.975469	3.50	0.0484107
204208_at	8732	0.95	-3.889816	2.28	0.048463
201577_at	4830	0.97	-3.806557	2.34	0.0485154
218439_s_at	51397	0.96	-3.656577	2.47	0.0485248
205633_s_at	211	0.96	-3.852859	2.31	0.0485335
229863_s_at	54859	0.96	-4.302382	2.03	0.0485485
224879_at	90871	0.95	-3.519688	2.60	0.0485538
227409_at	57594	0.94	-3.496031	2.63	0.0485801
204857_at	8379	1.06	3.745197	2.39	0.0486085
223076_s_at	54888	1.03	3.46096	2.67	0.0486107
230277_at	NA	0.86	-3.416424	2.72	0.0486587
234726_s_at	64418	0.93	-2.86607	3.79	0.0486756
211976_at	11224	0.94	-2.802408	4.00	0.0487165
227132_at	51123	1.08	3.467916	2.66	0.0487228
209845_at	23608	0.97	-2.858938	3.81	0.0487434
217630_at	90806	0.92	-2.984032	3.46	0.0487485
222431_at	10927	0.98	-2.826387	3.91	0.0487623
223978_s_at	54675	0.98	-2.810761	3.97	0.0487634
244038_at	112840	0.95	-4.31097	2.02	0.0488051
224900_at	51479	0.97	-3.196139	3.03	0.0488258
210547_x_at	3382	1.10	3.305708	2.86	0.0488377
227859_at	51277	0.87	-4.000973	2.20	0.04884
218415_at	26276	0.95	-3.58082	2.53	0.0488614
215497_s_at	23038	1.05	2.889868	3.70	0.0489132
223433_at	57002	0.95	-3.917732	2.25	0.0489158
212895_s_at	29	1.04	2.978418	3.46	0.0489328
219101_x_at	79575	1.10	3.215281	2.99	0.0489589
200970_s_at	27230	1.03	2.957647	3.51	0.0489729
224827_at	92181	0.99	-2.899141	3.67	0.0489748
212866_at	203069	0.97	-2.833262	3.87	0.0489901
223711_s_at	29087	0.94	-3.040268	3.32	0.0490033
217783_s_at	51646	1.04	3.74225	2.38	0.0490193

221649_s_at	56342	0.97	-3.576842	2.53	0.0490332
205995_x_at	9657	0.95	-2.812098	3.94	0.0490578
228544_s_at	57325	0.94	-2.803147	3.97	0.0490742
228176_at	1903	0.86	-3.605817	2.50	0.0490916
232352_at	64843	1.04	2.817161	3.92	0.0491104
224248_x_at	80011	0.97	-2.794987	4.00	0.0491271
229145_at	119504	0.95	-3.448009	2.66	0.0491311
228950_s_at	57708	0.96	-2.803137	3.96	0.0491464
218646_at	54969	0.96	-2.877329	3.72	0.0491596
218212_s_at	4338	0.95	-2.807631	3.95	0.0492001
209507_at	6119	0.95	-4.0528	2.15	0.0492319
204807_at	10329	0.94	-2.800981	3.97	0.0492425
219649_at	29929	0.96	-2.839983	3.83	0.0492495
223804_s_at	25917	0.97	-2.868123	3.74	0.0492705
211071_s_at	10962	1.02	3.641849	2.46	0.0492729
208922_s_at	10482	1.03	4.0525	2.15	0.0492992
225225_at	11339	0.97	-3.659881	2.44	0.0493402
202516_s_at	1739	0.95	-3.036342	3.31	0.0494019
201724_s_at	2589	1.02	4.122446	2.11	0.0494104
219231_at	96764	0.97	-3.037552	3.30	0.0494309
240537_s_at	440356	0.93	-2.842511	3.81	0.0494531
222449_at	56937	0.93	-2.798328	3.96	0.0494684
202710_at	10282	0.96	-3.035914	3.30	0.0494867
225543_at	9329	0.95	-3.151932	3.08	0.0494907
218575_at	64682	0.95	-4.026223	2.16	0.049499
226003_at	55605	1.06	3.170782	3.04	0.0495322
216125_s_at	10048	0.95	-3.151455	3.07	0.0495855
223318_s_at	84266	1.03	2.854665	3.76	0.0495912
203242_s_at	10611	1.12	4.258212	2.03	0.0496496
206387_at	1045	1.07	2.855553	3.75	0.0497166
223528_s_at	64745	0.97	-4.222163	2.05	0.0497256
228031_at	79183	0.96	-3.089153	3.18	0.0497266
227787_s_at	90390	0.94	-3.174633	3.02	0.0497507
218423_x_at	51542	0.96	-3.479365	2.61	0.049752
206864_s_at	8739	0.98	-3.438243	2.65	0.0497875
205986_at	9625	1.05	3.371293	2.73	0.0498059
208066_s_at	2959	1.01	2.785522	3.98	0.0498147
217028_at	7852	1.07	3.741907	2.36	0.0498224
225269_s_at	192137	0.97	-3.9388	2.21	0.0498344
217886_at	2060	0.92	-3.200497	2.98	0.0498451
238597_at	81573	0.92	-2.912019	3.58	0.0498533
218365_s_at	55157	0.94	-3.840467	2.28	0.0499173
239277_at	23597	0.94	-2.819312	3.85	0.0499904
