# Supplementary material for: A Systems Genetics Approach Implicates USF1, FADS3, and Other Causal Candidate Genes for Familial Combined Hyperlipidemia
Source: PLoS Genet. 2009 Sep 11;5(9):e1000642. doi: 10.1371/journal.pgen.1000642 (PMC2730565; doi:10.1371/journal.pgen.1000642)
Supplement: Table S2 — Genes correlated to FCHL-associated rs3737787 genotypes (additive model) in Mexican FCHL case/control fat biopsies (p-value≤0.05). (0.64 MB PDF) [file pgen.1000642.s004.pdf]

Table S2. Genes correlated to FCHL associated rs3737787 genotypes (additive model) in Mexican FCHL case/control fat biopsies (p-value ≤ 0.05).

Probe ID	Entrez ID	Effect Estimate	Std. Error	T-statistic	P-value
225091_at	85364	-0.120841	0.029313	-4.12239	9.91E-05
229211_at	285193	0.17443	0.042934	4.062779	0.000122
223515_s_at	51805	0.235321	0.058177	4.04492	0.00013
227009_at	NA	0.171814	0.043789	3.923698	0.000197
224728_at	64756	0.168484	0.043883	3.839411	0.000263
236122_at	387921	0.237422	0.0634	3.744834	0.000361
220949_s_at	78996	0.169526	0.045428	3.731745	0.000377
203545_at	79053	0.191475	0.052135	3.672675	0.000458
200690_at	3313	0.214029	0.058404	3.66464	0.00047
213282_at	340562	0.178646	0.048821	3.659189	0.000479
218669_at	57826	0.162974	0.044853	3.6335	0.000521
227776_at	55331	0.203662	0.056449	3.607925	0.000566
212799_at	10228	0.182995	0.050945	3.592009	0.000596
230266_at	338382	0.230118	0.064366	3.575141	0.00063
236321_at	285550	0.30235	0.084675	3.57072	0.000639
228011_at	137392	0.15592	0.044116	3.534336	0.000719
213895_at	2012	0.284328	0.080778	3.519872	0.000753
222824_at	55176	0.202137	0.057435	3.519408	0.000754
214047_s_at	8930	0.142977	0.040832	3.50164	0.000798
208934_s_at	3964	0.280079	0.080132	3.495215	0.000815
210681_s_at	9958	0.165123	0.047708	3.461143	0.000908
205077_s_at	5281	0.154838	0.044778	3.457873	0.000918
212175_s_at	204	0.146784	0.042738	3.434541	0.000988
208982_at	5175	-0.244748	0.071883	-3.404814	0.001086
215983_s_at	7993	0.184619	0.054498	3.387609	0.001146
223113_at	51524	0.204003	0.061233	3.331613	0.001365
213686_at	NA	0.15223	0.046082	3.303442	0.00149
238022_at	388279	0.276379	0.083694	3.302272	0.001495
222729_at	55294	0.166456	0.050723	3.281691	0.001593
226594_at	51004	0.15314	0.046677	3.28088	0.001597
235623_at	55250	-0.156681	0.047891	-3.271613	0.001643
203801_at	63931	0.218678	0.066871	3.27013	0.001651
208936_x_at	3964	0.222039	0.067945	3.267912	0.001662
242657_at	3487	-0.225248	0.069458	-3.242921	0.001794
222432_s_at	57003	0.150471	0.04642	3.241552	0.001802
206441_s_at	54939	0.118699	0.036635	3.24007	0.00181
220615_s_at	55711	0.265445	0.082627	3.212572	0.001968
203947_at	1479	0.111868	0.034847	3.210275	0.001982
226734_at	9470	0.142298	0.044364	3.20754	0.001999
206412_at	2241	0.155456	0.048953	3.175626	0.002201
213500_at	9276	0.093989	0.02961	3.174258	0.002211
223223_at	64801	0.140082	0.044173	3.171211	0.002231
210830_s_at	5445	0.207033	0.06539	3.166105	0.002266
218447_at	56942	0.206705	0.065793	3.141775	0.002438
AFFX-HUMISGF3A/M97935_MB_at	6772	0.344271	0.109624	3.140477	0.002447
223623_at	84417	-0.349309	0.111486	-3.133225	0.002501
235198_at	28962	0.292758	0.093823	3.120316	0.002599

238554_at	283852	0.186452	0.059824	3.116688	0.002627
222156_x_at	9236	0.266269	0.085469	3.115411	0.002637
221788_at	5238	0.157762	0.050658	3.114233	0.002647
208783_s_at	4179	0.10329	0.03321	3.110183	0.002679
221622_s_at	55863	0.167392	0.053923	3.104293	0.002726
222366_at	23394	-0.187697	0.060526	-3.101119	0.002752
233873_x_at	55149	-0.098612	0.031804	-3.100627	0.002756
223468_s_at	56963	-0.186111	0.060663	-3.06796	0.003036
209902_at	545	0.222256	0.072496	3.065748	0.003056
203655_at	7515	-0.11175	0.036476	-3.063677	0.003075
AFFX-HUMISGF3A/M97935_MA_at	6772	0.242311	0.079197	3.059599	0.003112
202272_s_at	23219	0.199134	0.065096	3.059072	0.003117
206477_s_at	4858	-0.128296	0.04237	-3.027993	0.003415
214696_at	84981	0.215337	0.071283	3.02087	0.003487
221892_at	9563	-0.267379	0.088632	-3.016735	0.00353
225409_at	493753	0.120449	0.03993	3.01647	0.003533
205395_s_at	4361	0.234889	0.077977	3.012277	0.003576
222151_s_at	80254	0.215244	0.071604	3.00602	0.003642
228970_at	339487	0.181798	0.060505	3.00467	0.003656
209580_s_at	8930	0.151726	0.050563	3.000704	0.003699
217922_at	10905	0.143037	0.04777	2.994275	0.003769
222578_s_at	79876	0.099069	0.0331	2.993043	0.003782
229126_at	55266	0.148658	0.049694	2.991492	0.003799
232144_at	5087	-0.237698	0.079597	-2.986255	0.003858
227566_at	50863	0.291884	0.097837	2.983359	0.00389
215283_at	400642	0.219088	0.073443	2.983125	0.003893
229419_at	55294	0.194869	0.065373	2.980873	0.003918
217808_s_at	79109	0.178381	0.059842	2.980861	0.003919
218059_at	51123	0.109468	0.036725	2.980764	0.00392
213017_at	171586	0.166764	0.055992	2.978328	0.003948
203662_s_at	7111	-0.19575	0.065739	-2.977695	0.003955
224964_s_at	54331	0.249795	0.083931	2.976182	0.003972
229666_s_at	1479	0.181327	0.061008	2.972175	0.004019
202089_s_at	25800	0.23066	0.077698	2.968672	0.00406
203661_s_at	7111	-0.192684	0.065031	-2.962974	0.004127
209132_s_at	54939	0.135776	0.045902	2.957968	0.004187
221541_at	83716	-0.238108	0.080627	-2.953206	0.004245
207749_s_at	5523	0.1535	0.052087	2.946986	0.004322
221895_at	158747	0.230381	0.078204	2.945896	0.004335
219307_at	57107	0.168091	0.057101	2.94373	0.004363
210570_x_at	5601	0.117661	0.040027	2.939547	0.004415
228368_at	57569	0.296568	0.100943	2.937973	0.004435
205055_at	3682	0.181625	0.061913	2.933554	0.004492
218488_at	8891	0.157978	0.05394	2.92878	0.004554
228817_at	NA	0.183591	0.06276	2.925296	0.0046
218890_x_at	51318	0.209449	0.071688	2.921697	0.004648
242760_x_at	9488	0.144231	0.049417	2.918668	0.004688
213970_at	285282	0.145903	0.049996	2.918285	0.004693
201992_s_at	3799	0.27035	0.092962	2.908191	0.004831
200974_at	59	-0.313324	0.108	-2.901144	0.004929
222531_s_at	55745	0.154497	0.053323	2.897396	0.004982

204080_at	114034	-0.078706	0.027169	-2.896869	0.004989
238021_s_at	388279	0.199344	0.06884	2.895754	0.005005
202814_s_at	10614	0.244543	0.084557	2.892037	0.005058
243303_at	55862	0.271694	0.094217	2.883692	0.00518
222530_s_at	8195	0.21524	0.074693	2.881665	0.00521
229549_at	611	0.230929	0.080205	2.879258	0.005246
203105_s_at	10059	0.157927	0.054855	2.878978	0.00525
64883_at	158747	0.205719	0.071539	2.87563	0.0053
218129_s_at	4801	0.214702	0.074747	2.872386	0.005349
219646_at	54849	0.15127	0.052702	2.870279	0.005381
212080_at	143941	-0.122136	0.042602	-2.866907	0.005432
203788_s_at	10512	0.407752	0.142293	2.865578	0.005453
219960_s_at	51377	0.145773	0.050887	2.864635	0.005467
230449_x_at	NA	0.167404	0.058531	2.860081	0.005538
203979_at	1593	-0.124497	0.043609	-2.854865	0.005621
219825_at	56603	0.369351	0.129842	2.844619	0.005785
217599_s_at	29969	0.393157	0.138529	2.838072	0.005893
222403_at	23788	0.226396	0.079868	2.834638	0.00595
223599_at	117854	0.298254	0.105224	2.834466	0.005953
226731_at	53918	0.148557	0.052507	2.82927	0.00604
203740_at	10200	0.107649	0.038071	2.827554	0.00607
213027_at	6738	0.126134	0.044637	2.825805	0.0061
200015_s_at	4735	0.072848	0.025786	2.825101	0.006112
222426_at	79109	0.153448	0.05441	2.820236	0.006195
225781_at	5601	0.119646	0.042424	2.820212	0.006196
203327_at	3416	0.168184	0.059706	2.816894	0.006254
201561_s_at	22883	-0.154699	0.054966	-2.81444	0.006297
217496_s_at	3416	0.211851	0.075307	2.813158	0.006319
218732_at	51651	0.1613	0.057376	2.811296	0.006352
203925_at	2730	0.259692	0.092508	2.807226	0.006425
202159_at	2193	-0.082376	0.029411	-2.800857	0.00654
227451_s_at	NA	0.203033	0.072564	2.797975	0.006593
224847_at	1021	0.165062	0.059011	2.797118	0.006609
230032_at	64172	0.133775	0.047924	2.791406	0.006715
232235_at	92126	0.226879	0.081291	2.790945	0.006723
214194_at	22894	0.099454	0.035645	2.790139	0.006739
238504_at	135154	0.233837	0.083826	2.78954	0.00675
209628_at	55916	0.229567	0.082322	2.788628	0.006767
202950_at	1429	0.194689	0.069826	2.78822	0.006775
204839_at	51367	0.175366	0.062926	2.786864	0.0068
226238_at	84693	0.181907	0.065305	2.785484	0.006826
227623_at	781	0.269938	0.096917	2.785264	0.006831
212625_at	8677	0.111642	0.040098	2.784215	0.00685
212214_at	4976	0.150176	0.054108	2.775511	0.007018
219029_at	64417	0.225374	0.081211	2.775154	0.007025
221808_at	9367	0.203583	0.073382	2.774294	0.007042
222622_at	283871	0.13874	0.050053	2.771871	0.007089
203789_s_at	10512	0.216347	0.078097	2.770236	0.007121
230061_at	116441	0.264631	0.09563	2.767223	0.007181
203918_at	5097	-0.10522	0.03804	-2.766042	0.007204
214748_at	88523	0.181297	0.065558	2.765457	0.007216

216942_s_at	965	0.239289	0.086648	2.761622	0.007293
218003_s_at	2287	0.141186	0.051136	2.760972	0.007306
202717_s_at	8881	0.126164	0.045724	2.759268	0.007341
212174_at	204	0.115291	0.041799	2.758209	0.007362
227413_at	134510	0.15577	0.056615	2.751381	0.007502
209796_s_at	10330	0.137239	0.049922	2.749067	0.00755
218099_at	55852	0.147392	0.053633	2.748143	0.007569
215165_x_at	7372	0.145527	0.052971	2.747275	0.007588
223960_s_at	29965	-0.122761	0.044709	-2.745797	0.007619
208724_s_at	5861	0.069263	0.025245	2.743637	0.007664
204057_at	3394	0.309323	0.112827	2.741561	0.007708
201821_s_at	10440	0.185177	0.067551	2.741304	0.007713
201957_at	4660	-0.199156	0.072821	-2.734881	0.007851
200621_at	1465	-0.228292	0.083548	-2.732464	0.007903
213918_s_at	25836	0.130507	0.047835	2.728276	0.007994
221573_at	79020	0.13576	0.049776	2.727396	0.008014
228239_at	54065	0.144971	0.05316	2.727046	0.008021
213227_at	10424	0.148524	0.054478	2.726305	0.008038
200978_at	4190	0.281669	0.103364	2.725005	0.008066
210737_at	7275	-0.126414	0.046417	-2.723459	0.008101
205222_at	1962	0.258845	0.095044	2.723423	0.008101
229181_s_at	55142	0.124013	0.045569	2.721428	0.008146
238678_at	388312	-0.199937	0.073473	-2.721219	0.00815
208626_s_at	10493	-0.105595	0.038823	-2.719884	0.00818
236004_at	NA	0.120463	0.044322	2.717878	0.008225
221449_s_at	81533	0.125582	0.04621	2.717612	0.008231
230172_at	122509	0.12103	0.04469	2.708197	0.008446
229025_s_at	196294	0.220084	0.081371	2.704687	0.008527
224958_at	57532	0.138652	0.051269	2.704399	0.008534
212381_at	23358	0.196251	0.072577	2.704024	0.008543
229595_at	131474	0.136692	0.050594	2.70177	0.008595
222132_s_at	55750	0.073508	0.027233	2.699174	0.008656
221229_s_at	55006	0.137114	0.05082	2.698004	0.008684
204171_at	6198	0.125485	0.046515	2.697728	0.00869
218534_s_at	55109	0.150897	0.055938	2.697588	0.008694
208679_s_at	10109	0.124805	0.046274	2.697089	0.008706
229144_at	23254	0.170069	0.063114	2.694657	0.008763
217881_s_at	996	0.146581	0.054457	2.6917	0.008834
227117_at	11260	0.164534	0.061184	2.689151	0.008895
213836_s_at	55062	0.156375	0.058193	2.687177	0.008943
207735_at	54941	-0.239072	0.08899	-2.686514	0.008959
212572_at	23012	0.185699	0.069139	2.685892	0.008975
226824_at	119587	-0.255148	0.095024	-2.685105	0.008994
222745_s_at	79768	0.123715	0.046118	2.682575	0.009056
203436_at	10556	0.136266	0.050864	2.67901	0.009144
205201_at	2737	-0.139435	0.052085	-2.677063	0.009192
200632_s_at	10397	-0.119804	0.044754	-2.676959	0.009195
213300_at	23130	-0.164888	0.061602	-2.676671	0.009202
226242_at	128061	0.106125	0.039683	2.674294	0.009261
220416_at	79895	0.220433	0.082476	2.672686	0.009302
204234_s_at	7748	0.151262	0.05666	2.669644	0.009379

229905_at	NA	0.150962	0.056598	2.667256	0.009439
202020_s_at	10314	0.147981	0.055488	2.666915	0.009448
204785_x_at	3455	-0.094393	0.035499	-2.659009	0.009652
204615_x_at	3422	0.133124	0.050099	2.657221	0.009699
218487_at	210	0.197164	0.074201	2.657149	0.0097
221492_s_at	64422	0.105608	0.039775	2.655131	0.009753
204163_at	11117	-0.180571	0.068036	-2.65403	0.009782
209320_at	109	-0.110891	0.041829	-2.651057	0.009861
214151_s_at	9236	0.176625	0.066626	2.651009	0.009862
57532_at	1856	-0.091148	0.034394	-2.650065	0.009887
219454_at	25975	1.271855	0.480091	2.649194	0.009911
229444_at	64771	0.200977	0.075932	2.646806	0.009974
202453_s_at	2965	0.111344	0.042093	2.645179	0.010018
201241_at	1653	0.1008	0.038137	2.643069	0.010075
212598_at	23001	0.131514	0.049763	2.642807	0.010082
225769_at	57511	0.160133	0.060608	2.642089	0.010102
228641_at	22900	0.158354	0.05994	2.641875	0.010107
212513_s_at	23032	0.122221	0.046294	2.640116	0.010155
203208_s_at	9650	0.125343	0.04749	2.639345	0.010176
218668_s_at	57826	0.19008	0.072121	2.635562	0.01028
229717_at	63891	-0.090328	0.034281	-2.634933	0.010298
223650_s_at	29982	0.165267	0.062725	2.634766	0.010302
34858_at	23510	-0.074625	0.028327	-2.634431	0.010311
201832_s_at	8615	0.097028	0.036848	2.633157	0.010347
218423_x_at	51542	0.119323	0.045348	2.631269	0.010399
207302_at	6445	0.276907	0.105253	2.630873	0.01041
213005_s_at	23189	0.191723	0.072903	2.629823	0.01044
210980_s_at	427	0.187952	0.071488	2.629153	0.010458
238020_at	375611	0.179663	0.068382	2.62735	0.010509
225562_at	22821	0.190624	0.07256	2.627104	0.010516
202295_s_at	1512	-0.27156	0.103586	-2.621589	0.010672
208664_s_at	7267	0.232877	0.088851	2.620998	0.010689
219006_at	29078	0.167267	0.063869	2.618922	0.010748
205182_s_at	25799	-0.137072	0.052343	-2.618708	0.010755
228489_at	116441	0.255629	0.097617	2.618704	0.010755
211090_s_at	8899	0.207097	0.079106	2.617979	0.010776
205784_x_at	421	-0.09708	0.037101	-2.616627	0.010814
214431_at	8833	-0.096934	0.037095	-2.613161	0.010915
228822_s_at	10600	0.108782	0.041667	2.610787	0.010984
203323_at	858	0.159961	0.061279	2.610394	0.010996
218299_at	53838	0.111439	0.042706	2.609455	0.011023
32094_at	9469	0.17434	0.066816	2.609254	0.011029
235096_at	123169	0.127847	0.049031	2.607473	0.011082
211574_s_at	4179	0.259749	0.099689	2.605586	0.011138
202195_s_at	50999	0.271498	0.104226	2.604892	0.011158
211137_s_at	27032	0.206844	0.079466	2.602919	0.011217
210544_s_at	224	0.17083	0.065639	2.60257	0.011227
AFFX-HUMISGF3A/M97935_3_at	6772	0.18633	0.071607	2.602103	0.011241
223211_at	26061	0.193609	0.074424	2.601442	0.011261
235509_at	137682	0.157231	0.060483	2.599596	0.011316
210875_s_at	6935	0.211279	0.081283	2.599301	0.011325

220926_s_at	80267	0.186002	0.071587	2.598265	0.011357
223804_s_at	25917	0.110861	0.042683	2.597319	0.011385
226020_s_at	1600	0.225577	0.086852	2.59725	0.011387
222547_at	9448	0.163787	0.06311	2.59526	0.011448
201012_at	301	0.128804	0.049631	2.595217	0.011449
209090_s_at	51100	0.11612	0.044785	2.592831	0.011522
202815_s_at	10614	0.159103	0.061367	2.59267	0.011527
204226_at	27067	0.137768	0.053271	2.586166	0.011727
236804_at	1312	-0.097473	0.037698	-2.585634	0.011744
214126_at	92014	0.203448	0.078699	2.585142	0.011759
203952_at	22926	0.095252	0.036883	2.582554	0.01184
203711_s_at	26275	0.20055	0.077664	2.58229	0.011848
209035_at	4192	-0.184583	0.071503	-2.581495	0.011873
242304_at	84305	0.115732	0.044832	2.581435	0.011875
203359_s_at	26292	0.254143	0.098565	2.578421	0.01197
219161_s_at	51192	0.188758	0.07321	2.578314	0.011973
202200_s_at	6732	0.122795	0.047631	2.578037	0.011982
222989_s_at	29979	0.14175	0.05499	2.577761	0.011991
236798_at	NA	0.175457	0.068114	2.575945	0.012049
233124_s_at	55862	0.235443	0.091456	2.574375	0.012099
202643_s_at	7128	0.156025	0.060618	2.573894	0.012114
227665_at	92014	0.16768	0.065176	2.572736	0.012151
218404_at	29887	0.352435	0.137105	2.570545	0.012222
212987_at	26268	0.106187	0.041311	2.570445	0.012225
207621_s_at	10400	0.413943	0.161149	2.568693	0.012281
219543_at	64081	0.204835	0.079754	2.56832	0.012294
226696_at	10741	0.092518	0.036028	2.567939	0.012306
229517_at	138639	0.116234	0.045269	2.567633	0.012316
238890_at	NA	0.14819	0.05786	2.561189	0.012527
213138_at	10865	-0.133569	0.052165	-2.560498	0.01255
212306_at	23122	0.131473	0.051399	2.557903	0.012636
228263_at	160622	-0.110587	0.043254	-2.556712	0.012675
223180_s_at	29090	0.16469	0.064425	2.556331	0.012688
203891_s_at	1613	-0.108038	0.042311	-2.553409	0.012786
225018_at	56907	0.149396	0.05852	2.552923	0.012802
229415_at	54205	0.188988	0.074058	2.551905	0.012836
229480_at	402560	0.213495	0.083703	2.550614	0.01288
205367_at	10603	-0.137484	0.053907	-2.550386	0.012888
204565_at	55856	0.233922	0.091733	2.550024	0.0129
208712_at	595	0.323612	0.126963	2.548875	0.012939
218664_at	51102	0.183396	0.071973	2.548099	0.012965
219030_at	51002	0.147833	0.058082	2.545259	0.013062
218095_s_at	55858	0.135799	0.053388	2.543653	0.013117
47530_at	51531	0.090872	0.035727	2.543522	0.013122
235538_at	5087	-0.193165	0.075995	-2.541813	0.013181
226860_at	55266	0.198602	0.078148	2.54136	0.013196
225630_at	80820	0.155994	0.061387	2.541174	0.013203
210568_s_at	5965	0.196939	0.077559	2.539209	0.013271
221058_s_at	51192	0.15283	0.060267	2.535869	0.013388
235305_s_at	55268	-0.211282	0.08337	-2.534261	0.013444
205434_s_at	22848	-0.11032	0.043545	-2.533483	0.013471

209137_s_at	9100	0.107926	0.042605	2.533193	0.013482
233827_s_at	11198	0.254942	0.100673	2.532376	0.01351
204001_at	6619	0.105346	0.041601	2.532284	0.013514
203486_s_at	25852	0.104801	0.041389	2.532081	0.013521
202729_s_at	4052	-0.290594	0.114822	-2.530823	0.013565
209473_at	953	-0.179324	0.070877	-2.53006	0.013592
243459_x_at	NA	-0.12898	0.050998	-2.529108	0.013626
213203_at	10302	0.114268	0.0452	2.528054	0.013664
234135_x_at	23022	-0.111667	0.04418	-2.527534	0.013682
205474_at	51379	0.105619	0.041796	2.527014	0.013701
219374_s_at	79796	0.169279	0.067013	2.52604	0.013736
226196_s_at	112752	0.120617	0.047753	2.525866	0.013742
203658_at	788	0.167961	0.066546	2.523999	0.013809
226742_at	51128	0.098004	0.038851	2.522573	0.013861
203426_s_at	3488	-0.16402	0.065034	-2.522056	0.013879
228217_s_at	389362	0.182717	0.072458	2.521686	0.013893
225110_at	55239	0.118863	0.047152	2.520841	0.013923
224702_at	153339	0.151489	0.06012	2.519788	0.013961
200638_s_at	7534	0.163984	0.065124	2.518016	0.014026
227808_at	29103	0.216216	0.085935	2.516031	0.014099
201627_s_at	3638	0.309277	0.122937	2.515737	0.01411
203970_s_at	8504	0.149671	0.059628	2.51007	0.014319
208264_s_at	8669	0.15587	0.062101	2.509947	0.014324
202728_s_at	4052	-0.258317	0.102924	-2.509788	0.01433
220342_x_at	80267	0.204369	0.081454	2.50902	0.014358
233431_x_at	80012	-0.132307	0.052734	-2.50893	0.014362
225537_at	122553	0.138348	0.055143	2.508873	0.014364
235174_s_at	NA	0.190752	0.076127	2.505699	0.014483
224469_s_at	84800	0.202215	0.080883	2.500097	0.014695
218597_s_at	55847	0.149947	0.059979	2.499997	0.014698
215883_at	NA	-0.121171	0.048479	-2.499446	0.014719
210716_s_at	6249	0.171766	0.068804	2.496453	0.014834
226331_at	56987	0.117769	0.047181	2.496083	0.014848
202541_at	9255	0.124654	0.049941	2.496006	0.014851
202496_at	23644	-0.127867	0.051308	-2.492171	0.014999
225882_at	84912	0.126387	0.050726	2.491539	0.015024
242565_x_at	54059	0.191875	0.077015	2.491401	0.015029
205335_s_at	6728	0.149444	0.059993	2.491022	0.015044
209349_at	10111	0.144885	0.058172	2.490638	0.015059
204298_s_at	4015	0.269836	0.108352	2.490366	0.015069
225638_at	388753	0.223456	0.089746	2.489872	0.015089
209841_s_at	54674	0.299018	0.120136	2.488994	0.015123
202828_s_at	4323	-0.143833	0.057789	-2.488929	0.015125
224960_at	55681	0.13695	0.055038	2.488264	0.015151
213671_s_at	4141	0.129677	0.052126	2.487763	0.015171
213513_x_at	10109	0.096885	0.038945	2.487749	0.015172
226339_at	142940	0.190777	0.076691	2.487602	0.015177
218768_at	57122	0.104823	0.04215	2.486883	0.015206
233759_s_at	57223	0.142329	0.05725	2.486085	0.015237
241359_at	NA	0.324257	0.130556	2.483653	0.015333
202955_s_at	10565	0.115321	0.046445	2.482969	0.01536

219224_x_at	79797	-0.083391	0.033639	-2.479019	0.015517
232436_at	10782	-0.131974	0.053241	-2.478799	0.015526
222665_at	51115	0.11112	0.044842	2.478055	0.015556
225788_at	88745	0.089775	0.036261	2.475816	0.015646
218909_at	26750	0.121205	0.04896	2.475598	0.015655
225341_at	80298	0.126824	0.051232	2.475481	0.015659
225674_at	55973	0.153197	0.061909	2.474558	0.015697
213861_s_at	25895	0.136746	0.055314	2.472172	0.015793
225748_at	84946	0.100497	0.040663	2.471476	0.015822
212065_s_at	9736	0.156364	0.063303	2.470089	0.015878
226958_s_at	400569	0.095783	0.038787	2.469475	0.015903
201775_s_at	9813	0.113798	0.046084	2.469378	0.015907
203031_s_at	7390	0.140648	0.056988	2.468024	0.015963
220065_at	64102	0.514315	0.208587	2.465712	0.016058
210018_x_at	10892	0.122498	0.04969	2.465232	0.016078
225153_at	85476	0.131712	0.053438	2.464759	0.016097
212264_s_at	23063	0.158603	0.064351	2.464658	0.016101
212213_x_at	4976	0.082293	0.033391	2.464555	0.016106
238477_at	10749	0.194006	0.078757	2.463366	0.016155
224436_s_at	25934	0.205518	0.083468	2.462221	0.016202
204500_s_at	23287	0.140358	0.057008	2.462068	0.016209
201198_s_at	5707	0.129334	0.052594	2.459091	0.016333
210104_at	10001	0.137104	0.055828	2.455818	0.016471
213305_s_at	5527	0.143532	0.058478	2.454447	0.016529
224735_at	220002	0.175678	0.071673	2.451106	0.016671
203387_s_at	9882	0.134231	0.054766	2.450994	0.016675
237154_at	NA	0.2684	0.109567	2.449653	0.016733
210990_s_at	3910	0.242009	0.098826	2.448837	0.016768
217751_at	373156	0.104988	0.042883	2.448234	0.016794
212333_at	25940	0.139737	0.057115	2.446611	0.016863
209141_at	7326	0.103025	0.042114	2.446358	0.016874
205241_at	9997	0.213278	0.087203	2.445745	0.016901
224831_at	80315	0.223891	0.091604	2.444112	0.016971
202142_at	10920	0.105799	0.043321	2.442246	0.017052
201014_s_at	10606	0.117219	0.048002	2.441942	0.017066
220768_s_at	1456	0.166018	0.06803	2.440354	0.017135
229253_at	117145	0.14624	0.059945	2.439565	0.01717
224773_at	89796	0.190328	0.078106	2.436785	0.017292
225061_at	55466	0.139233	0.057139	2.436747	0.017293
212643_at	93487	-0.092067	0.037789	-2.436357	0.017311
229018_at	84190	0.121635	0.049926	2.436332	0.017312
213205_s_at	23132	-0.115656	0.047497	-2.435034	0.017369
227862_at	388610	-0.189422	0.077858	-2.432929	0.017462
205084_at	55973	0.201865	0.082974	2.43286	0.017465
215236_s_at	8301	0.18158	0.074675	2.431601	0.017522
201831_s_at	8615	0.20176	0.082977	2.431534	0.017525
209421_at	4436	0.209163	0.086057	2.430529	0.017569
225143_at	119559	0.179943	0.074041	2.430311	0.017579
225040_s_at	6120	0.153166	0.063031	2.430031	0.017592
227292_at	144097	-0.123144	0.050687	-2.429473	0.017617
227501_at	26118	-0.174006	0.071625	-2.429404	0.01762

217916_s_at	51571	0.192102	0.079089	2.428947	0.01764
204019_s_at	26751	0.14458	0.059529	2.428735	0.01765
217879_at	996	0.103133	0.042489	2.427287	0.017715
241467_at	114879	-0.133118	0.054868	-2.426152	0.017766
209208_at	9526	0.142317	0.058714	2.423893	0.017868
209834_at	9469	0.182039	0.075113	2.423549	0.017884
225670_at	134145	0.164764	0.067993	2.423254	0.017897
208881_x_at	3422	0.145104	0.059969	2.419639	0.018062
203597_s_at	11193	0.145524	0.060157	2.419068	0.018089
214682_at	5310	-0.197241	0.081561	-2.418322	0.018123
222981_s_at	10890	0.169751	0.070218	2.417473	0.018162
203193_at	2101	-0.089068	0.036846	-2.417275	0.018171
223020_at	81037	0.160627	0.066478	2.416233	0.018219
219603_s_at	7769	0.210424	0.087091	2.416124	0.018224
203008_x_at	10190	0.182243	0.075447	2.415522	0.018252
226161_at	55676	0.143022	0.059271	2.413019	0.018368
213994_s_at	10418	0.32834	0.136189	2.410922	0.018466
201573_s_at	2107	0.175455	0.0728	2.410079	0.018505
208855_s_at	8428	-0.111047	0.046081	-2.409805	0.018518
202415_s_at	23640	-0.080481	0.033402	-2.409488	0.018533
233025_at	23037	0.257126	0.106714	2.409483	0.018533
227022_at	132789	0.168135	0.069868	2.406479	0.018675
202983_at	6596	0.132376	0.055011	2.406364	0.01868
225400_at	116461	0.101778	0.042317	2.405104	0.01874
222637_at	51397	0.234957	0.097723	2.404319	0.018777
207334_s_at	7048	0.24027	0.099982	2.403134	0.018833
223087_at	55862	0.224417	0.093404	2.402638	0.018857
205807_s_at	7286	0.186793	0.077758	2.402235	0.018876
213357_at	404672	0.210251	0.087614	2.399727	0.018996
212770_at	7090	0.129695	0.054085	2.397958	0.019081
226183_at	2932	0.151283	0.063143	2.395872	0.019182
205880_at	5587	0.158462	0.066191	2.393996	0.019272
227480_at	56241	-0.262336	0.109584	-2.393934	0.019275
213581_at	5134	0.141349	0.05907	2.392883	0.019327
209437_s_at	10418	0.290369	0.121373	2.392357	0.019352
212720_at	10914	0.179701	0.075119	2.392223	0.019359
210480_s_at	4646	0.131546	0.054997	2.391888	0.019375
213220_at	92482	0.114151	0.047746	2.390794	0.019429
58780_s_at	55701	0.172828	0.072357	2.388541	0.019539
209533_s_at	9373	0.120325	0.05039	2.387891	0.019571
204616_at	7347	0.232203	0.097265	2.387314	0.019599
225171_at	93663	0.140303	0.058775	2.387106	0.01961
241091_at	NA	0.105382	0.044148	2.387015	0.019614
214252_s_at	1203	0.176953	0.074136	2.386867	0.019621
218196_at	28962	0.206056	0.086332	2.386794	0.019625
201517_at	22916	0.081954	0.034341	2.386486	0.01964
202511_s_at	9474	0.125876	0.052747	2.386412	0.019644
201508_at	3487	-0.161409	0.067637	-2.386408	0.019644
213126_at	112950	0.126823	0.053146	2.386302	0.019649
227548_at	94101	0.291193	0.122046	2.385934	0.019668
212795_at	23325	0.136328	0.057162	2.384941	0.019717

202900_s_at	4927	0.14667	0.061517	2.384233	0.019752
210473_s_at	166647	0.132565	0.055636	2.382727	0.019827
223026_s_at	51699	0.139459	0.058543	2.382177	0.019854
209280_at	9902	-0.106961	0.044912	-2.381561	0.019885
215462_at	1263	-0.132198	0.055525	-2.380856	0.01992
233315_at	1488	-0.14924	0.062733	-2.378975	0.020014
218605_at	64216	0.133347	0.056061	2.378619	0.020032
218712_at	54955	0.122459	0.051488	2.378405	0.020043
220430_at	79927	-0.140111	0.058911	-2.378361	0.020045
209268_at	11311	0.105792	0.044502	2.377271	0.0201
1552310_at	123207	0.11922	0.050163	2.376663	0.020131
212343_at	286451	0.110102	0.046332	2.37637	0.020146
218722_s_at	79714	0.13014	0.054765	2.376359	0.020146
233449_at	NA	-0.217503	0.091542	-2.375983	0.020165
208071_s_at	3903	0.213932	0.09005	2.375688	0.02018
228415_at	8905	0.120182	0.050602	2.375037	0.020213
207238_s_at	5788	0.285768	0.120332	2.374837	0.020223
211563_s_at	8725	0.124267	0.052334	2.374492	0.020241
214004_s_at	9686	-0.118993	0.050117	-2.374299	0.020251
226757_at	3433	0.217994	0.091873	2.372777	0.020328
217843_s_at	29079	0.123638	0.052109	2.372684	0.020333
219096_at	79637	-0.097943	0.041311	-2.370855	0.020426
213860_x_at	1452	0.064871	0.027372	2.370007	0.020469
223019_at	64855	-0.121258	0.051174	-2.36951	0.020495
201068_s_at	5701	0.114976	0.048527	2.369331	0.020504
225904_at	126731	0.159373	0.06728	2.368787	0.020532
209512_at	84263	0.257752	0.108829	2.368405	0.020552
226727_at	284106	0.096235	0.040645	2.367696	0.020588
203951_at	1264	-0.40109	0.169435	-2.367223	0.020613
234915_s_at	8562	0.168509	0.071197	2.366794	0.020635
235028_at	NA	-0.227768	0.096286	-2.365541	0.020699
226106_at	50862	0.184477	0.078007	2.364887	0.020733
211547_s_at	5048	0.251654	0.106469	2.363634	0.020798
228841_at	90624	0.111055	0.047006	2.362581	0.020853
211139_s_at	4664	0.185008	0.078339	2.361641	0.020902
222607_s_at	22894	0.14362	0.060822	2.361294	0.02092
201025_at	9669	0.146737	0.062173	2.360134	0.020981
222867_s_at	51003	0.181047	0.07673	2.359538	0.021012
227110_at	3183	0.145296	0.061615	2.358142	0.021086
228967_at	10209	0.097318	0.04128	2.357484	0.02112
236075_s_at	7775	0.258127	0.109535	2.356581	0.021168
212905_at	23283	0.148408	0.062999	2.355727	0.021213
201098_at	9276	0.119205	0.050617	2.355038	0.021249
214033_at	368	0.363601	0.154487	2.353611	0.021325
210075_at	51257	0.200725	0.085284	2.353606	0.021325
201488_x_at	10657	-0.07962	0.03383	-2.353555	0.021328
219974_x_at	55862	0.250973	0.106639	2.35349	0.021332
226034_at	1846	0.362688	0.154135	2.353055	0.021355
221782_at	54431	0.186274	0.079163	2.353046	0.021355
227160_s_at	79133	0.218169	0.092722	2.352938	0.021361
201936_s_at	8672	0.117429	0.04991	2.352808	0.021368

227476_at	9926	0.216776	0.092188	2.351471	0.021439
212344_at	23213	-0.21251	0.090464	-2.349115	0.021565
203481_at	55719	0.165117	0.070322	2.347999	0.021625
221547_at	8559	0.142814	0.060841	2.347314	0.021662
203622_s_at	56902	0.155855	0.066408	2.346934	0.021683
225928_at	10490	0.196821	0.083952	2.344457	0.021817
211043_s_at	1212	0.089154	0.038052	2.342953	0.021898
213702_x_at	427	0.151108	0.064641	2.337636	0.022189
209534_x_at	11214	-0.138257	0.059149	-2.337446	0.0222
206108_s_at	6431	0.410992	0.175916	2.33629	0.022264
203367_at	11072	0.21879	0.093652	2.336195	0.022269
228142_at	29796	0.141815	0.060771	2.333611	0.022412
202293_at	10274	0.142222	0.060996	2.331641	0.022522
224744_at	54928	0.275937	0.118399	2.33057	0.022581
223568_s_at	84513	0.110784	0.04756	2.329349	0.02265
212543_at	202	0.178937	0.076968	2.324824	0.022905
218391_at	11267	0.096681	0.041594	2.324384	0.02293
204155_s_at	23387	-0.079045	0.034022	-2.323379	0.022987
210145_at	5321	0.322663	0.138888	2.323193	0.022997
206550_s_at	9631	0.108375	0.046667	2.322317	0.023047
224774_s_at	89796	0.163581	0.070501	2.320266	0.023164
240557_at	NA	0.15627	0.067374	2.319425	0.023212
201768_s_at	9685	0.122551	0.052843	2.319167	0.023227
51146_at	55650	0.129097	0.055703	2.317594	0.023317
226360_at	84133	0.141763	0.061197	2.316494	0.023381
206342_x_at	3423	0.125562	0.054221	2.315752	0.023424
228647_at	4815	0.144558	0.062487	2.313423	0.023559
208945_s_at	8678	0.100689	0.043525	2.313381	0.023561
217884_at	55226	-0.088843	0.03843	-2.311832	0.023651
203676_at	2799	0.234375	0.10139	2.311616	0.023664
213543_at	6444	0.137548	0.059517	2.311064	0.023696
218297_at	80013	0.105288	0.045578	2.310059	0.023755
220933_s_at	79670	0.094528	0.040924	2.309828	0.023768
223347_at	84939	0.125021	0.054141	2.309184	0.023806
223516_s_at	29964	0.124965	0.054122	2.308948	0.02382
242263_at	50999	0.226067	0.097975	2.307395	0.023911
223213_s_at	11244	0.153435	0.066511	2.306922	0.023939
212815_at	10973	0.152824	0.06625	2.306784	0.023947
220739_s_at	26505	-0.133929	0.05807	-2.306337	0.023973
203647_s_at	2230	0.228638	0.099153	2.305908	0.023998
213725_x_at	283824	0.177748	0.077182	2.302975	0.024172
201359_at	1315	0.080717	0.035068	2.301747	0.024245
223880_x_at	55969	0.210386	0.091406	2.301671	0.02425
218789_s_at	54494	0.142388	0.06189	2.300678	0.024309
200889_s_at	6745	0.197172	0.085703	2.300651	0.02431
225783_at	140739	0.160002	0.069552	2.300468	0.024321
228716_at	7068	0.161963	0.07042	2.299976	0.024351
232180_at	7360	0.170362	0.074081	2.29966	0.02437
229173_at	80856	0.153597	0.066794	2.299568	0.024375
221545_x_at	10025	-0.145799	0.063409	-2.299323	0.02439
201659_s_at	400	0.184786	0.08038	2.298907	0.024415

225953_at	55197	0.160338	0.069759	2.298453	0.024442
208072_s_at	8527	0.101316	0.044081	2.298395	0.024445
220386_s_at	27436	0.169227	0.073649	2.297737	0.024485
227443_at	286343	0.151506	0.065961	2.296902	0.024535
204209_at	5130	0.114319	0.049777	2.296612	0.024553
223186_at	387521	0.165443	0.07205	2.296217	0.024576
205408_at	8028	0.111559	0.04859	2.295928	0.024594
218455_at	9054	0.086655	0.037744	2.295897	0.024596
228255_at	65062	0.138606	0.060379	2.295586	0.024615
210879_s_at	26056	-0.096617	0.042096	-2.295159	0.02464
205530_at	2110	0.192395	0.083847	2.294613	0.024673
202539_s_at	3156	0.190569	0.083059	2.294367	0.024688
201701_s_at	10424	0.137948	0.060142	2.293715	0.024728
225392_at	84340	0.109462	0.047746	2.292573	0.024797
200692_s_at	3313	0.11585	0.050551	2.291771	0.024846
202737_s_at	25804	0.179394	0.07829	2.29141	0.024868
202685_s_at	558	-0.168093	0.073378	-2.290796	0.024905
203641_s_at	22837	0.211437	0.0923	2.290762	0.024907
226685_at	6645	0.101751	0.044474	2.287906	0.025082
209096_at	7336	0.159025	0.069518	2.287531	0.025105
217777_s_at	51495	0.195982	0.085697	2.286908	0.025143
232570_s_at	80332	-0.251077	0.109805	-2.286576	0.025164
210768_x_at	54499	0.124467	0.054458	2.285563	0.025226
204084_s_at	1203	0.1565	0.068482	2.285266	0.025244
221847_at	NA	0.138666	0.060736	2.283076	0.02538
206271_at	7098	0.155642	0.068181	2.282786	0.025398
217746_s_at	10015	0.076514	0.033521	2.28258	0.025411
204257_at	3995	0.196453	0.086068	2.282542	0.025413
205808_at	444	0.349211	0.153055	2.281607	0.025471
212857_x_at	10923	0.152875	0.067016	2.281169	0.025498
205452_at	9488	0.150152	0.065833	2.280804	0.025521
228188_at	2355	-0.224348	0.098376	-2.28053	0.025538
225581_s_at	54534	0.209294	0.091781	2.280355	0.025549
212438_at	11017	0.147273	0.064617	2.27918	0.025623
209935_at	27032	0.178934	0.078525	2.278668	0.025655
231324_at	6598	-0.128661	0.056469	-2.278459	0.025668
227594_at	9204	0.147811	0.06494	2.276118	0.025815
200728_at	10097	0.134221	0.058983	2.275585	0.025848
221727_at	10923	0.255628	0.112419	2.273891	0.025955
215171_s_at	10440	0.142089	0.062503	2.273304	0.025992
203502_at	669	0.197979	0.087122	2.27243	0.026048
208873_s_at	7905	0.080499	0.035465	2.269781	0.026216
217757_at	2	-0.086762	0.038227	-2.269632	0.026226
211958_at	3488	-0.210633	0.092857	-2.268359	0.026307
200653_s_at	808	0.120902	0.05332	2.26747	0.026364
209822_s_at	7436	0.302561	0.13346	2.267062	0.02639
209954_x_at	6760	0.112731	0.049744	2.266233	0.026444
201389_at	3678	-0.146764	0.064783	-2.265471	0.026493
214383_x_at	116138	0.118466	0.052294	2.265395	0.026498
235940_at	84267	0.129944	0.057387	2.264349	0.026565
202322_s_at	9453	0.12165	0.053733	2.263968	0.02659

209311_at	599	0.0994	0.043906	2.263917	0.026593
209128_s_at	9733	-0.076439	0.033787	-2.262388	0.026692
212160_at	11260	0.12166	0.053779	2.262231	0.026702
200027_at	4677	0.071021	0.031407	2.261315	0.026761
223328_at	83787	0.124054	0.054867	2.26102	0.026781
213704_at	5876	0.162815	0.072019	2.260717	0.0268
209788_s_at	51752	0.246687	0.109135	2.26038	0.026822
210312_s_at	90410	0.101594	0.044949	2.260205	0.026834
235408_x_at	7670	0.160749	0.07116	2.258989	0.026913
213478_at	23254	0.140438	0.062252	2.255972	0.027111
212789_at	23310	0.118159	0.052399	2.254997	0.027175
214036_at	NA	0.118725	0.052668	2.254211	0.027227
222613_at	57102	0.124529	0.055275	2.25291	0.027313
218289_s_at	79876	0.1066	0.047317	2.252878	0.027315
59625_at	8996	0.169153	0.075101	2.252326	0.027351
221005_s_at	81490	-0.088291	0.039206	-2.251996	0.027373
211692_s_at	27113	-0.107895	0.047912	-2.251974	0.027375
222498_at	64343	0.118984	0.052836	2.251931	0.027377
225305_at	123096	-0.129522	0.057516	-2.251914	0.027379
202088_at	25800	0.139121	0.061781	2.251843	0.027383
223088_x_at	55862	0.202189	0.089863	2.249977	0.027507
228593_at	339483	-0.16054	0.071353	-2.249936	0.02751
208971_at	7389	0.136606	0.060735	2.249218	0.027558
218386_x_at	10600	0.088258	0.039247	2.248787	0.027587
206662_at	2745	0.161687	0.071963	2.246805	0.027719
221962_s_at	7328	0.180903	0.080517	2.246764	0.027722
209840_s_at	54674	0.275616	0.122739	2.245549	0.027803
219865_at	29092	0.263014	0.117134	2.245418	0.027812
226946_at	133686	0.181088	0.080652	2.245306	0.02782
222725_s_at	54873	0.19857	0.088451	2.244964	0.027843
227466_at	285550	0.134776	0.060037	2.244891	0.027848
224938_at	57532	0.117104	0.052193	2.243659	0.027931
218713_at	79664	0.117005	0.052187	2.242063	0.028039
221489_s_at	81848	0.173144	0.077233	2.241838	0.028054
210666_at	3423	-0.099391	0.044347	-2.241227	0.028095
218622_at	79023	0.17564	0.078415	2.239885	0.028186
227068_at	5230	0.185063	0.082622	2.239866	0.028188
235181_at	129450	0.090646	0.040478	2.239375	0.028221
242022_at	5087	-0.17154	0.076608	-2.239183	0.028234
203218_at	5601	0.10866	0.048534	2.238829	0.028258
217834_s_at	10492	0.136243	0.060866	2.238408	0.028287
226777_at	8038	0.425276	0.190014	2.238125	0.028306
222502_s_at	51569	0.203276	0.090838	2.237794	0.028329
223091_x_at	56947	0.105605	0.047215	2.236699	0.028404
238919_at	5101	0.245875	0.109946	2.23632	0.02843
213786_at	8887	0.143876	0.064359	2.235519	0.028485
201626_at	3638	0.277626	0.12421	2.235143	0.028511
55583_at	57572	0.172088	0.077005	2.234758	0.028537
225101_s_at	57231	0.109766	0.049129	2.234255	0.028572
201634_s_at	80777	0.173825	0.077812	2.233907	0.028596
222150_s_at	54103	0.120835	0.054109	2.233173	0.028646

200729_s_at	10097	0.176469	0.079076	2.231649	0.028752
210218_s_at	6672	0.152878	0.068515	2.231325	0.028774
227988_s_at	23230	0.112783	0.050579	2.22984	0.028877
218842_at	79657	0.162222	0.072783	2.22884	0.028947
227188_at	59271	-0.174766	0.078427	-2.228391	0.028978
218711_s_at	8436	0.160825	0.07226	2.225658	0.029169
224931_at	54946	-0.098312	0.04418	-2.22525	0.029197
213552_at	26035	0.212566	0.095538	2.224927	0.02922
201067_at	5701	0.11022	0.049564	2.223778	0.029301
209666_s_at	1147	0.141233	0.063515	2.223633	0.029311
224233_s_at	55154	0.201774	0.090768	2.222972	0.029358
204118_at	962	0.182379	0.082049	2.222812	0.029369
220327_at	389136	0.293652	0.132259	2.220277	0.029548
226343_at	54878	0.078026	0.035156	2.219433	0.029608
223907_s_at	54984	-0.091512	0.041234	-2.219347	0.029614
202137_s_at	10771	0.134924	0.060809	2.218812	0.029652
231166_at	151556	0.169712	0.076542	2.217241	0.029764
223054_at	51726	0.082675	0.037297	2.216645	0.029807
221044_s_at	53840	0.11934	0.053846	2.216324	0.02983
228494_at	55607	0.197373	0.089077	2.215762	0.02987
204278_s_at	9166	0.102876	0.046446	2.214965	0.029927
33494_at	2110	0.175017	0.079024	2.214728	0.029944
202070_s_at	3419	0.153351	0.069323	2.212122	0.030131
225213_at	160760	0.113589	0.051357	2.211768	0.030157
203207_s_at	9650	0.167502	0.075734	2.211734	0.030159
208911_s_at	5162	0.136972	0.06193	2.211722	0.03016
208711_s_at	595	0.275213	0.124489	2.210751	0.03023
229618_at	64089	0.214007	0.096855	2.209565	0.030316
202054_s_at	224	0.109187	0.04942	2.209373	0.03033
226935_s_at	81037	0.110276	0.049992	2.205853	0.030587
203403_s_at	6049	0.120358	0.054576	2.205333	0.030625
201662_s_at	2181	0.151873	0.068873	2.20511	0.030641
218304_s_at	114885	0.183502	0.083257	2.204046	0.030719
231101_at	5529	0.178238	0.080883	2.203644	0.030748
211985_s_at	808	0.153099	0.069514	2.20243	0.030838
203487_s_at	25852	0.111874	0.050796	2.202405	0.030839
206101_at	1842	0.106802	0.048505	2.201883	0.030878
226810_at	79940	0.140992	0.064041	2.201606	0.030898
209896_s_at	5781	0.226456	0.102883	2.201092	0.030936
201611_s_at	23463	0.112289	0.051022	2.200796	0.030958
222990_at	29979	0.094831	0.043093	2.200615	0.030971
210028_s_at	23595	0.079058	0.03593	2.20034	0.030992
216396_s_at	9538	0.105859	0.048114	2.200151	0.031006
221532_s_at	80349	0.123498	0.056161	2.199008	0.03109
213551_x_at	7703	-0.107561	0.048929	-2.198301	0.031143
226923_at	152579	0.086234	0.039252	2.196936	0.031244
214483_s_at	27236	0.208282	0.094839	2.19617	0.031301
218139_s_at	55745	0.144115	0.065627	2.195973	0.031316
201652_at	10987	0.125643	0.057232	2.195313	0.031365
201725_at	8872	0.090499	0.04123	2.19498	0.03139
208656_s_at	10983	-0.0891	0.040605	-2.194289	0.031442

225161_at	85476	0.12857	0.058594	2.19426	0.031444
225841_at	113802	0.127332	0.058041	2.193838	0.031476
212835_at	23172	0.116066	0.052906	2.193818	0.031477
202203_s_at	267	0.47858	0.218177	2.193543	0.031498
242181_at	NA	0.268758	0.122579	2.192537	0.031573
226805_at	128486	0.213615	0.097434	2.192407	0.031583
230282_at	10099	0.21301	0.097186	2.191787	0.03163
213587_s_at	155066	0.140725	0.064224	2.191149	0.031678
223620_at	2857	0.287303	0.13113	2.190986	0.03169
204971_at	1475	0.307883	0.140533	2.190831	0.031702
202816_s_at	6760	0.146174	0.066737	2.190308	0.031741
213440_at	5861	0.097466	0.044514	2.189539	0.031799
212138_at	23244	0.105352	0.048118	2.18943	0.031808
223100_s_at	11164	0.145227	0.066359	2.188494	0.031879
200598_s_at	7184	0.135196	0.061784	2.188193	0.031901
224898_at	80232	0.162027	0.074068	2.18756	0.031949
216899_s_at	8935	0.197634	0.090353	2.187349	0.031965
208743_s_at	7529	0.11269	0.051526	2.187062	0.031987
238756_at	283431	0.206042	0.09424	2.186342	0.032042
211368_s_at	834	0.172349	0.078844	2.185951	0.032072
218918_at	57134	-0.168368	0.077084	-2.184208	0.032205
235301_at	222223	0.125616	0.057514	2.184106	0.032213
217835_x_at	55969	0.194043	0.088856	2.183794	0.032236
225981_at	283987	-0.112783	0.051654	-2.183426	0.032265
204141_at	7280	0.255666	0.117112	2.183098	0.03229
207108_s_at	25836	0.115568	0.052938	2.183077	0.032291
209142_s_at	7326	0.146781	0.06729	2.181332	0.032425
215699_x_at	9814	-0.099384	0.045566	-2.181084	0.032444
223035_s_at	10056	0.063834	0.02927	2.180853	0.032462
210042_s_at	1522	0.233124	0.106908	2.180608	0.032481
212692_s_at	987	0.120867	0.055446	2.179894	0.032536
218636_s_at	11253	-0.081206	0.037272	-2.178728	0.032626
238032_at	9249	-0.160283	0.073595	-2.177902	0.03269
222687_s_at	55331	0.159392	0.073189	2.177809	0.032697
218078_s_at	51304	0.123925	0.05691	2.177556	0.032717
221803_s_at	29982	0.143638	0.06599	2.176654	0.032787
217907_at	29074	0.1377	0.063279	2.176072	0.032832
219146_at	79736	0.123784	0.056898	2.175545	0.032873
213548_s_at	55573	0.238471	0.109617	2.17549	0.032877
214202_at	5229	0.113345	0.052104	2.175369	0.032887
222526_at	54815	0.120341	0.055351	2.174157	0.032981
201013_s_at	10606	0.133619	0.061482	2.173308	0.033048
200900_s_at	4074	0.140968	0.064864	2.173293	0.033049
224878_at	56061	0.070642	0.032511	2.172855	0.033083
221563_at	11221	0.15933	0.073336	2.172608	0.033102
205608_s_at	284	0.230957	0.106341	2.171845	0.033162
238320_at	283131	0.432128	0.198972	2.171808	0.033165
241650_x_at	256158	-0.104795	0.048258	-2.171552	0.033185
91920_at	63827	-0.096628	0.044501	-2.171347	0.033201
224888_at	85465	0.089788	0.041392	2.169192	0.033371
210540_s_at	8702	0.099574	0.045912	2.168769	0.033404

218435_at	29103	0.204264	0.094195	2.168527	0.033423
218019_s_at	8566	0.192736	0.088896	2.168103	0.033457
223341_s_at	60592	0.196424	0.090626	2.16742	0.033511
226501_at	63929	0.140692	0.064913	2.167389	0.033513
1598_g_at	2621	-0.133933	0.061826	-2.166288	0.033601
208803_s_at	6731	0.104622	0.048297	2.166221	0.033606
221227_x_at	51805	0.086122	0.039761	2.165998	0.033624
228937_at	144811	0.170107	0.078542	2.165802	0.033639
218549_s_at	51115	0.220691	0.101966	2.164352	0.033755
221020_s_at	81034	0.126855	0.058633	2.163556	0.033818
201742_x_at	6426	0.108258	0.050057	2.162704	0.033886
227040_at	387921	0.144382	0.066779	2.162092	0.033935
203162_s_at	10300	0.103614	0.04793	2.16177	0.033961
223178_s_at	221294	0.157647	0.072931	2.161588	0.033975
226191_at	2932	0.122052	0.056485	2.160791	0.034039
226135_at	54887	0.11605	0.053711	2.16063	0.034052
46665_at	54910	-0.099456	0.046035	-2.160461	0.034066
221882_s_at	58986	0.131175	0.060725	2.160165	0.03409
201724_s_at	2589	0.185943	0.086089	2.159887	0.034112
203981_s_at	5826	-0.11561	0.053527	-2.159846	0.034115
220027_s_at	54922	-0.153329	0.071046	-2.158173	0.03425
202113_s_at	6643	0.131459	0.060928	2.157613	0.034295
208955_at	1854	0.118006	0.054706	2.157103	0.034336
225003_at	374882	0.13101	0.060782	2.155397	0.034475
202068_s_at	3949	0.301676	0.139983	2.155085	0.0345
209276_s_at	2745	0.219845	0.102146	2.152262	0.03473
207791_s_at	5861	0.151998	0.070672	2.150753	0.034853
217718_s_at	7529	0.063141	0.029359	2.150682	0.034859
212206_s_at	94239	0.097418	0.045308	2.150117	0.034905
220419_s_at	29761	0.081449	0.037882	2.15006	0.03491
218547_at	79947	0.188231	0.087557	2.149814	0.03493
208946_s_at	8678	0.089476	0.041626	2.1495	0.034956
224864_at	10011	0.089576	0.041675	2.149387	0.034965
226510_at	25938	0.111225	0.051753	2.149145	0.034985
222586_s_at	114885	0.152054	0.070768	2.148619	0.035028
37590_g_at	374655	0.220887	0.10283	2.148081	0.035073
200769_s_at	4144	0.157173	0.073211	2.146856	0.035174
221511_x_at	9236	0.148712	0.069307	2.145718	0.035268
213165_at	9857	0.111756	0.052085	2.145625	0.035275
221787_at	387263	0.13928	0.06492	2.145424	0.035292
221864_at	93129	-0.097551	0.045475	-2.145157	0.035314
205218_at	10621	0.07481	0.034878	2.144887	0.035337
220036_s_at	55716	-0.09789	0.045641	-2.144759	0.035347
205018_s_at	10150	0.278876	0.130029	2.144717	0.035351
201991_s_at	3799	0.11964	0.055787	2.144568	0.035363
202587_s_at	203	0.110304	0.051435	2.14451	0.035368
227268_at	51136	0.186278	0.086885	2.143964	0.035413
242137_at	27303	0.213367	0.09953	2.143754	0.035431
57163_at	64834	0.098899	0.046141	2.143384	0.035461
231968_at	56886	0.09013	0.042067	2.142513	0.035534
1557411_s_at	203427	0.185616	0.086654	2.142033	0.035574

205499_at	27286	0.269364	0.12576	2.141883	0.035586
235349_at	151393	0.098153	0.045833	2.141531	0.035616
205392_s_at	6359	-0.186948	0.087304	-2.141355	0.03563
233558_s_at	60684	0.09466	0.044212	2.141054	0.035656
213853_at	120526	0.086542	0.040425	2.140825	0.035675
201843_s_at	2202	0.155796	0.072776	2.140779	0.035679
212602_at	23001	0.111863	0.052263	2.1404	0.03571
224662_at	3799	0.156286	0.073039	2.139759	0.035764
209436_at	10418	0.252517	0.118016	2.13968	0.035771
225378_at	137492	0.124427	0.058159	2.139429	0.035792
239135_at	55313	0.161611	0.075577	2.138376	0.03588
236664_at	NA	0.172065	0.080506	2.137284	0.035972
205078_at	5281	0.201719	0.094382	2.137267	0.035973
218296_x_at	55154	0.185969	0.087055	2.136238	0.03606
223214_s_at	11244	0.129413	0.060585	2.136038	0.036077
207069_s_at	4091	0.182762	0.085573	2.135752	0.036101
211959_at	3488	-0.110642	0.051815	-2.135334	0.036136
223286_at	23587	0.124495	0.058307	2.135168	0.03615
226394_at	54708	0.125607	0.058863	2.133886	0.036259
230329_s_at	11162	0.162391	0.076107	2.133716	0.036273
201396_s_at	6449	-0.056645	0.026549	-2.133549	0.036288
226208_at	57688	0.113401	0.053153	2.133478	0.036294
222907_x_at	757	0.174615	0.081887	2.132387	0.036386
219064_at	80760	0.263663	0.123664	2.132102	0.036411
201823_s_at	9604	0.204029	0.095714	2.131643	0.03645
228228_at	147906	-0.148184	0.069517	-2.131629	0.036451
208655_at	10983	-0.081352	0.038176	-2.130988	0.036505
207988_s_at	10109	0.09235	0.043358	2.129967	0.036593
203100_s_at	9425	0.101903	0.047848	2.129724	0.036613
207549_x_at	4179	0.223782	0.105094	2.129354	0.036645
218721_s_at	54953	0.110916	0.052091	2.129281	0.036651
222775_s_at	51318	0.114732	0.053887	2.129131	0.036664
212008_at	23190	0.167712	0.078845	2.127092	0.036839
225987_at	79689	-0.151036	0.071035	-2.126225	0.036914
201684_s_at	9878	-0.063704	0.029966	-2.125902	0.036941
202049_s_at	9202	0.118248	0.055637	2.125357	0.036989
203972_s_at	8504	0.108262	0.050946	2.125052	0.037015
212184_s_at	23118	0.117745	0.055419	2.124621	0.037052
214626_s_at	23193	-0.081491	0.038358	-2.12449	0.037063
210089_s_at	3910	0.167708	0.078946	2.124339	0.037076
219657_s_at	51274	0.096236	0.045323	2.123315	0.037165
212439_at	9807	-0.090049	0.042414	-2.123109	0.037183
215128_at	NA	0.12801	0.060326	2.121958	0.037283
202069_s_at	3419	0.168305	0.079323	2.121782	0.037298
225881_at	84912	0.159365	0.075123	2.121383	0.037333
202297_s_at	11079	0.072413	0.034138	2.121152	0.037353
208800_at	6731	0.105208	0.049604	2.120937	0.037372
224614_at	1783	0.114195	0.053843	2.120905	0.037374
229544_at	NA	0.184854	0.087168	2.12066	0.037396
218852_at	55012	0.101132	0.04769	2.120614	0.0374
226994_at	10294	0.10544	0.049724	2.120515	0.037408

203500_at	2639	0.113497	0.053532	2.120161	0.037439
219664_s_at	26063	0.103639	0.048886	2.120019	0.037451
224180_x_at	51057	0.17674	0.083381	2.119678	0.037481
228037_at	5914	-0.097988	0.046245	-2.118868	0.037552
214011_s_at	51491	0.113344	0.053494	2.118798	0.037558
235919_at	NA	0.156748	0.073986	2.118613	0.037574
200887_s_at	6772	0.157547	0.074392	2.117786	0.037647
219489_s_at	64359	-0.144135	0.068072	-2.117392	0.037681
222617_s_at	63877	0.109158	0.051563	2.117002	0.037715
221531_at	80349	0.179817	0.084957	2.116566	0.037754
222805_at	79694	0.175417	0.082912	2.115697	0.03783
229497_at	348094	0.172605	0.081598	2.115296	0.037865
218578_at	79577	0.150566	0.071207	2.114491	0.037936
205427_at	6940	0.108226	0.051205	2.113576	0.038017
227442_at	285521	0.081262	0.038462	2.112758	0.038089
224641_at	84248	0.114351	0.054125	2.112702	0.038094
226909_at	85460	0.170119	0.08055	2.111969	0.038159
213353_at	23461	0.129593	0.061373	2.111557	0.038196
222503_s_at	55255	0.146168	0.069234	2.111218	0.038226
228283_at	152100	0.167541	0.079359	2.111178	0.03823
209135_at	444	0.128271	0.060758	2.111158	0.038231
228540_at	9444	0.140637	0.06663	2.110707	0.038271
218989_x_at	64924	0.139294	0.066001	2.110474	0.038292
223240_at	26269	0.16742	0.079376	2.109213	0.038404
202639_s_at	8498	-0.071965	0.034134	-2.108295	0.038486
219979_s_at	51501	0.259418	0.12307	2.107883	0.038523
213224_s_at	92482	0.113549	0.053871	2.107777	0.038533
201876_at	5445	0.130064	0.061716	2.107461	0.038561
218133_s_at	60491	0.096706	0.045914	2.106244	0.03867
203823_at	5998	-0.208553	0.099126	-2.103905	0.03888
222278_at	NA	0.257593	0.122436	2.103898	0.038881
212271_at	5594	0.154435	0.073408	2.103783	0.038891
236144_at	119587	-0.18204	0.086542	-2.103478	0.038918
202112_at	7450	-0.167832	0.079788	-2.103473	0.038919
212217_at	9581	0.132971	0.063228	2.103052	0.038957
209047_at	358	-0.161066	0.076622	-2.102077	0.039045
225200_at	285381	0.16256	0.07737	2.101075	0.039136
209409_at	2887	-0.10136	0.048244	-2.100996	0.039143
224957_at	6139	0.148234	0.070559	2.100859	0.039155
201898_s_at	7319	0.108179	0.051494	2.100809	0.03916
219933_at	51022	0.19519	0.092922	2.100576	0.039181
210529_s_at	9747	0.144745	0.068912	2.100428	0.039194
223337_at	10283	0.098533	0.046918	2.100122	0.039222
203189_s_at	4728	0.209525	0.099771	2.100067	0.039227
223356_s_at	219402	0.109147	0.052001	2.098955	0.039328
218439_s_at	51397	0.189588	0.090332	2.098785	0.039344
219091_s_at	79812	-0.14832	0.070672	-2.09871	0.03935
213644_at	201134	0.128131	0.06106	2.098432	0.039376
225204_at	160760	0.118884	0.056662	2.098106	0.039405
223300_s_at	79780	0.114472	0.054563	2.097967	0.039418
225747_at	93058	0.101686	0.048472	2.097812	0.039432

223184_s_at	56894	0.159293	0.075937	2.097705	0.039442
212610_at	5781	0.126781	0.060441	2.097582	0.039453
209575_at	3588	0.105862	0.050471	2.097503	0.03946
225051_at	2035	0.149445	0.071254	2.097357	0.039474
218193_s_at	51026	0.212753	0.101485	2.0964	0.039561
225766_s_at	3842	0.161179	0.076897	2.096043	0.039594
218086_at	56654	-0.152992	0.073008	-2.095548	0.039639
203434_s_at	4311	0.33685	0.160815	2.094643	0.039722
225810_at	54893	0.112135	0.053555	2.093819	0.039798
214830_at	145389	0.211206	0.100874	2.093747	0.039804
203435_s_at	4311	0.228162	0.108995	2.093336	0.039842
220176_at	80224	0.128781	0.061542	2.092567	0.039913
209797_at	10330	0.10036	0.047967	2.092281	0.039939
211527_x_at	7422	0.194176	0.092819	2.091985	0.039967
212652_s_at	8723	0.097073	0.046404	2.091897	0.039975
201709_s_at	8508	-0.065872	0.031492	-2.091708	0.039992
208116_s_at	4121	0.263673	0.126074	2.091409	0.04002
225538_at	84240	0.096894	0.046344	2.090753	0.040081
206790_s_at	4707	0.145896	0.069785	2.090637	0.040091
218751_s_at	55294	0.129345	0.061909	2.089266	0.040218
225967_s_at	284184	0.139671	0.06686	2.089004	0.040243
205792_at	8839	-0.298903	0.143115	-2.088553	0.040285
39817_s_at	10591	0.117752	0.056388	2.08824	0.040314
212833_at	91137	0.12174	0.058299	2.088213	0.040316
202346_at	3093	0.127075	0.060853	2.088212	0.040316
233800_at	163	-0.100326	0.048058	-2.087613	0.040372
201842_s_at	2202	0.120208	0.057616	2.086375	0.040487
226381_at	NA	0.107251	0.05142	2.085791	0.040542
214152_at	9236	0.135619	0.065027	2.085568	0.040563
202242_at	7102	-0.200268	0.09605	-2.085029	0.040613
203888_at	7056	-0.182927	0.087748	-2.084694	0.040644
217493_x_at	9436	-0.103782	0.049812	-2.083474	0.040759
201124_at	3693	0.148569	0.071355	2.082106	0.040887
219679_s_at	51322	0.145092	0.06969	2.081962	0.040901
203374_s_at	7174	0.117967	0.05667	2.081658	0.04093
203752_s_at	3727	-0.098943	0.04755	-2.080819	0.041009
213504_at	10980	0.070993	0.034128	2.080203	0.041067
224860_at	90871	0.157736	0.075914	2.077828	0.041292
205688_at	7023	-0.091301	0.043948	-2.077477	0.041325
203517_at	10651	0.107457	0.051729	2.077325	0.041339
203428_s_at	25842	0.153837	0.07406	2.077203	0.041351
214434_at	259217	0.217049	0.104512	2.076787	0.041391
220199_s_at	64853	0.12147	0.058501	2.076396	0.041428
204134_at	5138	-0.11511	0.05545	-2.075935	0.041472
52741_at	115708	-0.085728	0.041297	-2.075904	0.041475
215439_x_at	171024	-0.107719	0.051903	-2.075404	0.041522
227791_at	285195	0.130112	0.062709	2.074847	0.041575
218536_at	57380	0.113281	0.054605	2.074561	0.041603
217791_s_at	5832	0.115806	0.055839	2.073947	0.041661
220956_s_at	112398	-0.070392	0.033945	-2.073707	0.041684
222623_s_at	51193	0.090492	0.043666	2.072382	0.041811

202499_s_at	6515	-0.209985	0.10134	-2.072088	0.04184
214845_s_at	813	0.221012	0.10669	2.071533	0.041893
203840_at	8548	0.133212	0.064312	2.07135	0.04191
221992_at	441773	0.361326	0.174452	2.07121	0.041924
242989_at	NA	0.133606	0.064508	2.071149	0.04193
201295_s_at	26118	0.212949	0.102821	2.071063	0.041938
220690_s_at	25979	0.132199	0.063848	2.070529	0.041989
217826_s_at	51465	0.133814	0.064641	2.0701	0.042031
212650_at	23301	0.207673	0.100338	2.06974	0.042065
209549_s_at	1716	0.10252	0.049542	2.069367	0.042101
201722_s_at	2589	0.137352	0.066382	2.069121	0.042125
227678_at	91419	0.279832	0.135278	2.068567	0.042179
227672_at	619348	-0.092551	0.044757	-2.067863	0.042247
209000_s_at	23176	0.104988	0.050775	2.067711	0.042262
201801_s_at	2030	0.132275	0.063976	2.067574	0.042275
218219_s_at	55915	0.112504	0.054416	2.06747	0.042285
221600_s_at	28971	0.156471	0.075695	2.067133	0.042318
219242_at	80254	0.1033	0.050004	2.065842	0.042443
213086_s_at	1452	0.077151	0.037361	2.065018	0.042523
227333_at	123879	0.145952	0.070713	2.064019	0.04262
202662_s_at	3709	0.182702	0.08852	2.063969	0.042625
201236_s_at	7832	-0.114999	0.055726	-2.063656	0.042656
208653_s_at	8763	0.291035	0.141076	2.062972	0.042723
222686_s_at	55313	0.131348	0.063716	2.061443	0.042872
227145_at	84171	-0.093825	0.045524	-2.060978	0.042918
222688_at	55331	0.136376	0.066174	2.060885	0.042927
227414_at	84236	0.113559	0.055118	2.060271	0.042987
222631_at	55300	0.107704	0.052299	2.059394	0.043074
210418_s_at	3420	0.099763	0.048451	2.059058	0.043107
216008_s_at	10425	0.065546	0.031839	2.058702	0.043142
223712_at	84105	0.09597	0.046625	2.058324	0.043179
239272_at	79148	0.151556	0.073637	2.058151	0.043196
200872_at	6281	0.073687	0.035809	2.057791	0.043232
201860_s_at	5327	-0.28201	0.137049	-2.05773	0.043238
204485_s_at	10040	0.1964	0.095463	2.057332	0.043277
40829_at	23038	-0.08538	0.041501	-2.057305	0.04328
225857_s_at	388796	-0.115311	0.056055	-2.057083	0.043302
200756_x_at	813	0.126849	0.061676	2.056692	0.04334
201274_at	5686	0.131486	0.063932	2.056658	0.043344
225988_at	26091	0.10837	0.052697	2.056478	0.043361
202202_s_at	3910	0.092613	0.045046	2.055964	0.043412
221276_s_at	81493	0.27045	0.131557	2.055768	0.043432
205384_at	5348	-0.121805	0.059254	-2.055633	0.043445
224905_at	80232	0.118473	0.057651	2.055002	0.043508
208870_x_at	509	0.125935	0.06135	2.052733	0.043734
227029_at	283635	0.132813	0.064711	2.052408	0.043766
234660_s_at	22894	0.158685	0.077324	2.052193	0.043788
203834_s_at	10618	0.148642	0.072442	2.051884	0.043818
222103_at	466	0.17685	0.08619	2.051859	0.043821
213131_at	10439	-0.180587	0.088088	-2.050062	0.044001
209119_x_at	7026	-0.214338	0.104559	-2.049924	0.044015

209377_s_at	9324	0.089462	0.043645	2.049775	0.044029
235374_at	4190	0.186452	0.09098	2.049366	0.044071
218756_s_at	79154	0.296769	0.144821	2.049212	0.044086
38069_at	1186	-0.088412	0.043155	-2.048685	0.044139
201504_s_at	7247	0.192198	0.093839	2.048172	0.044191
233849_s_at	394	0.181405	0.088624	2.046922	0.044317
223288_at	84640	0.150711	0.073633	2.046778	0.044331
203258_at	10589	0.108877	0.053224	2.045645	0.044445
232024_at	26157	0.190736	0.093242	2.045591	0.044451
201609_x_at	23463	0.087747	0.042898	2.045448	0.044465
218955_at	55290	-0.097122	0.047489	-2.045168	0.044494
202808_at	54838	0.093263	0.045603	2.045078	0.044503
202515_at	1739	0.120369	0.058875	2.044489	0.044563
218493_at	79622	0.135465	0.066266	2.044263	0.044585
213936_x_at	6439	-0.113488	0.055545	-2.04319	0.044694
218233_s_at	29964	0.079594	0.038964	2.042736	0.044741
222396_at	51155	0.10167	0.049778	2.042468	0.044768
212250_at	92140	0.101519	0.049705	2.04244	0.044771
231779_at	3656	-0.152981	0.074904	-2.042373	0.044777
212879_x_at	51588	-0.104444	0.051146	-2.042069	0.044808
226894_at	23443	0.110034	0.053904	2.041294	0.044887
219423_x_at	8718	0.239704	0.117438	2.041118	0.044905
201069_at	4313	-0.191978	0.094085	-2.040471	0.044971
210257_x_at	8450	0.123536	0.060544	2.040437	0.044975
203642_s_at	22837	0.160574	0.078743	2.039227	0.045099
209029_at	50813	0.082261	0.040343	2.039027	0.045119
224130_s_at	10011	0.11075	0.054317	2.038964	0.045126
209694_at	83875	0.130033	0.063792	2.038399	0.045184
220985_s_at	81790	0.138771	0.068097	2.037849	0.04524
203845_at	8850	0.125641	0.061661	2.037598	0.045266
203261_at	10671	0.1369	0.067204	2.037088	0.045318
212571_at	57680	-0.087416	0.042921	-2.036682	0.04536
219783_at	54978	-0.101965	0.050075	-2.036229	0.045407
227503_at	NA	0.14788	0.072629	2.036102	0.04542
205801_s_at	25780	0.173753	0.085369	2.035305	0.045502
203150_at	10244	0.066811	0.032831	2.034967	0.045537
238480_at	619463	0.16632	0.081744	2.034633	0.045571
222427_s_at	51520	0.119649	0.058817	2.034277	0.045608
210284_s_at	23118	0.168454	0.082811	2.034202	0.045616
211098_x_at	54499	0.128975	0.063404	2.034163	0.04562
209969_s_at	6772	0.208185	0.10243	2.032461	0.045796
232080_at	57520	0.238766	0.117477	2.032443	0.045798
203071_at	7869	-0.132714	0.065298	-2.032422	0.0458
218341_at	79717	0.102334	0.050355	2.032239	0.045819
227291_s_at	388962	0.183948	0.090518	2.032163	0.045827
212785_s_at	51574	0.097834	0.048146	2.032016	0.045842
234985_at	143458	0.157191	0.077357	2.032011	0.045843
229829_at	147525	0.097808	0.048146	2.031498	0.045896
211536_x_at	6885	0.110237	0.054266	2.031408	0.045906
208617_s_at	8073	0.093596	0.046088	2.030829	0.045966
229164_s_at	80325	-0.100345	0.049412	-2.030795	0.045969

204906_at	6196	-0.111795	0.055059	-2.03045	0.046005
223204_at	51313	0.171018	0.084229	2.030387	0.046012
212296_at	10213	0.152958	0.075371	2.029398	0.046115
209115_at	9039	0.098262	0.04842	2.029368	0.046118
201614_s_at	8607	0.092186	0.045431	2.029136	0.046142
226896_at	118487	0.162396	0.080069	2.02821	0.046239
216755_at	114884	-0.109564	0.05402	-2.028208	0.046239
213365_at	123879	0.154542	0.076218	2.02764	0.046299
222719_s_at	56034	0.193612	0.095493	2.027509	0.046313
202151_s_at	10422	0.087182	0.043003	2.027344	0.04633
204068_at	6788	0.134403	0.066299	2.027225	0.046342
226235_at	339290	0.127377	0.062847	2.02679	0.046388
210896_s_at	444	0.167234	0.082529	2.026366	0.046432
222786_at	55501	-0.09611	0.047433	-2.026239	0.046446
227322_s_at	56647	0.098594	0.048692	2.024856	0.046591
219579_at	5866	-0.113138	0.05589	-2.02431	0.046649
221876_at	55537	-0.065399	0.032308	-2.02423	0.046657
218118_s_at	10431	0.163139	0.080603	2.023972	0.046685
201750_s_at	1889	-0.087692	0.043329	-2.023873	0.046695
202593_s_at	51573	0.167966	0.083024	2.0231	0.046777
211758_x_at	10190	0.140975	0.069706	2.022421	0.046848
222393_s_at	80218	0.198439	0.098193	2.020905	0.047009
207826_s_at	3399	-0.175727	0.086955	-2.020891	0.04701
213627_at	10916	-0.094339	0.046683	-2.020818	0.047018
201811_x_at	9467	-0.07467	0.036953	-2.020653	0.047036
218992_at	55848	0.147829	0.073193	2.019719	0.047135
212122_at	284988	0.194116	0.096112	2.019689	0.047138
225885_at	440107	0.143935	0.071322	2.018106	0.047307
240830_at	4645	-0.11023	0.05463	-2.017734	0.047347
204125_at	51103	0.109491	0.054272	2.017453	0.047377
224395_s_at	9616	0.156114	0.077405	2.016834	0.047443
229817_at	57507	0.108889	0.05401	2.016078	0.047524
224772_at	89796	0.12169	0.060367	2.015824	0.047551
234339_s_at	29997	-0.104801	0.052	-2.015412	0.047595
201647_s_at	950	0.226764	0.112516	2.015393	0.047597
218642_s_at	79145	0.110076	0.054632	2.014872	0.047653
241936_x_at	NA	-0.181094	0.089906	-2.014249	0.04772
213142_x_at	54103	0.104117	0.051693	2.014151	0.047731
224682_at	54467	0.083783	0.041603	2.013895	0.047758
201447_at	7072	0.105222	0.05225	2.013829	0.047765
202278_s_at	10558	0.153943	0.076469	2.013141	0.047839
217825_s_at	51465	0.13238	0.065778	2.012511	0.047907
236947_at	10512	0.211247	0.104982	2.012216	0.047939
208787_at	11222	0.11225	0.055796	2.011796	0.047984
220547_s_at	54537	0.07764	0.038594	2.011706	0.047994
231921_at	80067	0.118351	0.058854	2.010921	0.048079
222798_at	9317	0.120433	0.059901	2.01054	0.04812
215346_at	958	-0.087482	0.043525	-2.009942	0.048185
226457_at	NA	0.115768	0.057601	2.009821	0.048198
201418_s_at	6659	-0.112822	0.056162	-2.008868	0.048302
235566_at	7110	0.085904	0.042773	2.008369	0.048356

203142_s_at	8546	0.064588	0.032163	2.008155	0.048379
223451_s_at	51192	0.149022	0.074229	2.007611	0.048438
203625_x_at	6502	0.142648	0.071087	2.006665	0.048541
218505_at	79726	-0.088258	0.043987	-2.00647	0.048563
213023_at	7402	0.150356	0.074941	2.006318	0.048579
225805_at	3192	0.13986	0.069726	2.005857	0.04863
200776_s_at	9689	0.182213	0.090873	2.005145	0.048707
214045_at	11019	0.119632	0.059666	2.005042	0.048719
203714_s_at	6905	0.069395	0.034624	2.004251	0.048805
212648_at	54505	0.085415	0.042646	2.002898	0.048954
213374_x_at	26275	0.130981	0.065406	2.002605	0.048986
234414_at	441457	-0.10649	0.053177	-2.002553	0.048992
216080_s_at	3995	0.212269	0.106007	2.002411	0.049007
227075_at	55140	0.10186	0.050871	2.002306	0.049019
206468_s_at	51603	0.102223	0.051056	2.002154	0.049036
203767_s_at	412	0.20815	0.103977	2.001895	0.049064
205609_at	284	0.173808	0.086866	2.000869	0.049177
209041_s_at	7327	0.130934	0.065455	2.000362	0.049233
225871_at	261729	0.157399	0.078744	1.998869	0.049398
206030_at	443	0.147698	0.073899	1.998646	0.049423
204238_s_at	10591	0.15989	0.080021	1.998089	0.049485
200737_at	5230	0.152667	0.076409	1.998036	0.049491
202670_at	5604	0.151029	0.075601	1.99772	0.049526
231766_s_at	1303	0.196344	0.098293	1.997536	0.049546
219209_at	64135	0.171703	0.085962	1.997439	0.049557
208985_s_at	8669	0.09844	0.049293	1.99703	0.049602
213875_x_at	81688	0.157795	0.079051	1.996118	0.049704
243801_x_at	51263	-0.104162	0.052186	-1.995991	0.049718
222689_at	55331	0.163585	0.081985	1.995303	0.049795
227658_s_at	65977	0.11908	0.059705	1.994472	0.049887
219173_at	80022	-0.177994	0.089252	-1.994292	0.049907
