# Supplementary material for: A Systems Genetics Approach Implicates USF1, FADS3, and Other Causal Candidate Genes for Familial Combined Hyperlipidemia
Source: PLoS Genet. 2009 Sep 11;5(9):e1000642. doi: 10.1371/journal.pgen.1000642 (PMC2730565; doi:10.1371/journal.pgen.1000642)
Supplement: Table S3 — Genes differentially expressed between FCHL cases and normolipidemic controls in Mexican FCHL case/control fat biopsies (p-value≤0.05). (1.42 MB PDF) [file pgen.1000642.s005.pdf]

Table S3. Genes differentially expressed between FCHL cases and normolipidemic controls in Mexican FCHL case/control fat biopsies (p-value ≤ 0.05).

Probe ID	Entrez ID	Effect Estimate	Std. Error	T-statistic	P-value
213142_x_at	54103	0.29	0.05	6.34	1.95E-08
222150_s_at	54103	0.29	0.05	5.98	8.57E-08
225704_at	57666	0.29	0.05	5.52	5.34E-07
202155_s_at	8021	0.23	0.05	5.06	3.19E-06
211577_s_at	3479	-0.62	0.13	-4.81	8.25E-06
207677_s_at	4689	0.54	0.11	4.80	8.56E-06
219809_at	54853	0.18	0.04	4.79	9.03E-06
212528_at	27351	0.27	0.06	4.73	1.15E-05
232489_at	54482	-0.55	0.12	-4.64	1.60E-05
228098_s_at	29116	-0.27	0.06	-4.61	1.74E-05
213811_x_at	6929	0.19	0.04	4.59	1.88E-05
231166_at	151556	0.33	0.07	4.58	1.96E-05
226777_at	8038	0.86	0.19	4.54	2.26E-05
201002_s_at	7335	0.20	0.04	4.51	2.59E-05
208003_s_at	10725	-0.45	0.10	-4.49	2.78E-05
202728_s_at	4052	-0.46	0.10	-4.47	2.92E-05
212757_s_at	818	0.22	0.05	4.44	3.29E-05
238472_at	26268	0.19	0.04	4.43	3.35E-05
202341_s_at	23321	-0.45	0.10	-4.43	3.40E-05
229115_at	1778	-0.38	0.09	-4.34	4.63E-05
210474_s_at	985	0.26	0.06	4.33	4.96E-05
227837_at	54891	0.14	0.03	4.32	5.04E-05
212561_at	23258	0.17	0.04	4.31	5.17E-05
227082_at	NA	-0.54	0.13	-4.31	5.27E-05
225074_at	84932	0.16	0.04	4.27	5.94E-05
209542_x_at	3479	-0.58	0.14	-4.27	6.02E-05
201860_s_at	5327	-0.57	0.13	-4.26	6.18E-05
242022_at	5087	-0.32	0.07	-4.24	6.70E-05
230180_at	10521	-0.57	0.13	-4.24	6.78E-05
219064_at	80760	0.52	0.12	4.22	7.15E-05
232940_s_at	58508	-0.27	0.07	-4.21	7.36E-05
241702_at	3184	-0.43	0.10	-4.20	7.64E-05
210719_s_at	10362	0.24	0.06	4.20	7.64E-05
219078_at	55105	0.29	0.07	4.18	8.34E-05
208789_at	284119	0.19	0.05	4.17	8.70E-05
203388_at	409	0.36	0.09	4.16	8.84E-05
202998_s_at	4017	0.49	0.12	4.15	9.24E-05
202957_at	3059	0.36	0.09	4.13	9.78E-05
216250_s_at	9404	0.28	0.07	4.13	9.95E-05
229699_at	NA	0.22	0.05	4.12	0.0001025
204169_at	3614	0.21	0.05	4.08	0.0001163
217746_s_at	10015	0.13	0.03	4.08	0.0001165
235233_s_at	NA	0.22	0.05	4.08	0.0001192
239901_at	378805	-0.36	0.09	-4.08	0.0001197
208914_at	23062	0.16	0.04	4.07	0.0001228
213998_s_at	10521	-0.54	0.13	-4.06	0.0001271
204735_at	5141	0.25	0.06	4.04	0.0001362
241359_at	NA	0.52	0.13	4.03	0.0001391

213790_at	8038	0.71	0.18	4.03	0.0001419
200702_s_at	57062	-0.26	0.06	-4.02	0.0001448
230424_at	9315	-0.43	0.11	-4.02	0.0001462
244876_at	1879	-0.32	0.08	-4.02	0.0001463
225750_at	30001	0.19	0.05	4.01	0.0001479
236254_at	157680	-0.36	0.09	-4.01	0.0001487
230337_at	6654	-0.45	0.11	-4.01	0.0001495
217736_s_at	27102	0.18	0.05	4.00	0.0001571
235427_at	8837	0.24	0.06	4.00	0.0001572
232852_at	9079	-0.44	0.11	-3.99	0.0001585
219261_at	79034	0.20	0.05	3.95	0.0001818
226607_at	25943	0.19	0.05	3.95	0.0001852
213448_at	2629	0.32	0.08	3.94	0.0001904
226393_at	113612	-0.15	0.04	-3.94	0.0001915
228847_at	11336	0.24	0.06	3.93	0.0001941
212629_s_at	5586	-0.31	0.08	-3.93	0.0001961
233261_at	1879	-0.60	0.15	-3.93	0.0001997
225637_at	54849	0.18	0.05	3.92	0.0002051
235308_at	26137	-0.21	0.05	-3.91	0.0002102
229302_at	130733	0.47	0.12	3.91	0.000212
225045_at	55704	0.26	0.07	3.90	0.000216
218282_at	55741	0.15	0.04	3.90	0.0002195
217838_s_at	51466	0.29	0.08	3.90	0.0002216
229948_at	NA	0.32	0.08	3.89	0.0002274
205270_s_at	3937	0.33	0.09	3.86	0.0002499
218241_at	9950	0.16	0.04	3.85	0.0002579
226168_at	130617	0.15	0.04	3.85	0.0002581
244420_at	862	-0.56	0.14	-3.85	0.0002598
216212_s_at	1736	-0.12	0.03	-3.84	0.0002698
239135_at	55313	0.29	0.08	3.83	0.0002734
215684_s_at	84164	0.26	0.07	3.83	0.0002745
235757_at	54906	-0.44	0.12	-3.82	0.0002822
225565_at	1385	-0.24	0.06	-3.81	0.0002923
224563_at	10163	-0.27	0.07	-3.81	0.0002944
230375_at	25957	-0.47	0.12	-3.81	0.0002969
208636_at	87	0.25	0.07	3.80	0.0003097
234104_at	1634	-0.53	0.14	-3.80	0.0003099
228224_at	5549	0.33	0.09	3.79	0.000321
218480_at	60509	0.23	0.06	3.78	0.0003227
227456_s_at	221545	0.22	0.06	3.77	0.0003399
235408_x_at	7670	0.27	0.07	3.77	0.0003417
212890_at	124565	0.28	0.08	3.76	0.000344
212468_at	9043	-0.33	0.09	-3.76	0.0003541
239571_at	4205	-0.38	0.10	-3.75	0.000355
209907_s_at	50618	0.18	0.05	3.75	0.0003578
219183_s_at	27128	0.33	0.09	3.75	0.0003583
219866_at	53405	-0.31	0.08	-3.75	0.00036
226975_at	55599	-0.29	0.08	-3.74	0.0003673
225883_at	89849	0.28	0.08	3.74	0.0003723
200710_at	37	0.28	0.08	3.74	0.0003784
204674_at	4033	0.20	0.05	3.73	0.0003801
212007_at	23190	-0.24	0.07	-3.73	0.0003807

220173_at	80127	0.19	0.05	3.73	0.0003895
212625_at	8677	0.15	0.04	3.72	0.0003952
235630_at	NA	0.25	0.07	3.71	0.0004063
227138_at	10491	0.27	0.07	3.71	0.0004103
239673_at	4306	-0.42	0.11	-3.71	0.0004123
218020_s_at	60685	0.22	0.06	3.71	0.0004176
205283_at	2218	0.20	0.05	3.71	0.0004187
117_at	3310	0.54	0.15	3.70	0.0004212
211358_s_at	25792	0.23	0.06	3.70	0.0004218
229036_at	23112	-0.21	0.06	-3.70	0.0004246
225219_at	4090	-0.29	0.08	-3.70	0.0004257
229765_at	NA	-0.55	0.15	-3.69	0.0004374
223111_x_at	51742	-0.20	0.05	-3.69	0.0004381
201702_s_at	5514	-0.28	0.08	-3.69	0.0004434
222824_at	55176	0.23	0.06	3.69	0.0004448
242652_at	2037	-0.34	0.09	-3.68	0.0004513
236368_at	23392	-0.35	0.10	-3.68	0.0004538
209216_at	11152	0.19	0.05	3.68	0.0004547
227327_at	90198	0.22	0.06	3.67	0.0004682
212794_s_at	23325	-0.36	0.10	-3.67	0.0004716
204193_at	1120	0.20	0.06	3.67	0.0004733
205606_at	4040	-0.22	0.06	-3.66	0.0004789
47560_at	22859	0.33	0.09	3.66	0.0004841
230925_at	54518	0.35	0.10	3.66	0.0004848
232929_at	NA	-0.37	0.10	-3.66	0.0004864
206030_at	443	0.28	0.08	3.66	0.0004883
239185_at	10350	-0.55	0.15	-3.65	0.0004945
213302_at	5198	0.26	0.07	3.65	0.0004982
212841_s_at	8495	0.20	0.05	3.65	0.0004987
224598_at	11282	0.21	0.06	3.64	0.0005098
200035_at	23399	0.15	0.04	3.64	0.0005099
236000_s_at	3184	-0.40	0.11	-3.64	0.0005111
209879_at	6404	0.28	0.08	3.64	0.0005138
243908_at	27332	-0.40	0.11	-3.64	0.0005145
228590_at	55037	-0.18	0.05	-3.64	0.0005146
235674_at	23240	0.25	0.07	3.64	0.0005196
212239_at	5295	-0.34	0.09	-3.64	0.0005209
232125_at	NA	-0.47	0.13	-3.63	0.0005288
226792_s_at	90990	0.20	0.06	3.63	0.0005367
221210_s_at	80896	0.61	0.17	3.63	0.0005384
203424_s_at	3488	-0.57	0.16	-3.62	0.0005494
208806_at	1107	0.26	0.07	3.62	0.0005541
203126_at	3613	0.37	0.10	3.62	0.0005543
236841_at	374666	-0.36	0.10	-3.62	0.0005618
231281_at	9779	-0.45	0.12	-3.61	0.0005703
220189_s_at	11282	0.22	0.06	3.61	0.0005743
206649_s_at	7030	0.27	0.07	3.61	0.0005754
224583_at	23406	0.40	0.11	3.61	0.0005776
224568_x_at	378938	-0.71	0.20	-3.60	0.0005841
218821_at	79716	0.24	0.07	3.60	0.0005949
201064_s_at	8761	0.20	0.05	3.60	0.0005968
223578_x_at	29005	-0.49	0.14	-3.59	0.00061

225282_at	64744	0.25	0.07	3.59	0.0006137
209518_at	6602	0.20	0.06	3.58	0.0006217
224451_x_at	64333	0.39	0.11	3.58	0.0006222
204731_at	7049	-0.24	0.07	-3.58	0.000625
226157_at	7029	0.22	0.06	3.58	0.0006273
205090_s_at	51172	0.20	0.06	3.58	0.0006279
236124_at	153546	0.16	0.05	3.57	0.0006452
212156_at	23339	0.20	0.06	3.57	0.0006466
228566_at	55197	0.21	0.06	3.57	0.0006481
224726_at	57534	-0.40	0.11	-3.57	0.0006518
226068_at	6850	0.37	0.10	3.57	0.0006555
228298_at	91523	0.25	0.07	3.57	0.0006576
213364_s_at	6642	0.15	0.04	3.56	0.0006627
214464_at	8476	-0.28	0.08	-3.56	0.0006673
223577_x_at	29005	-0.23	0.06	-3.56	0.0006674
213039_at	23370	0.18	0.05	3.55	0.0006825
212822_at	57493	-0.25	0.07	-3.55	0.0006835
223519_at	51776	-0.30	0.08	-3.55	0.0006891
229410_at	5047	0.22	0.06	3.55	0.0007015
203025_at	8260	0.16	0.05	3.55	0.0007023
232097_at	9878	-0.28	0.08	-3.54	0.0007057
212034_s_at	23265	0.15	0.04	3.54	0.0007093
202412_s_at	7398	-0.26	0.07	-3.54	0.0007124
221698_s_at	64581	0.45	0.13	3.54	0.0007188
235804_at	NA	-0.21	0.06	-3.54	0.000724
212913_at	4439	0.32	0.09	3.54	0.0007248
227811_at	89846	0.25	0.07	3.53	0.0007278
212208_at	23389	-0.20	0.06	-3.53	0.0007288
213593_s_at	29896	-0.39	0.11	-3.53	0.0007296
204265_s_at	63940	0.29	0.08	3.53	0.0007315
235538_at	5087	-0.28	0.08	-3.53	0.0007369
220178_at	126321	0.33	0.09	3.52	0.0007602
225364_at	6789	0.14	0.04	3.52	0.0007623
228622_s_at	3338	0.17	0.05	3.51	0.0007807
238447_at	27303	-0.28	0.08	-3.51	0.0007808
235392_at	3667	-0.49	0.14	-3.51	0.0007836
209244_s_at	10749	0.19	0.05	3.51	0.0007838
229274_at	2778	-0.24	0.07	-3.51	0.0007862
223405_at	80896	0.44	0.13	3.51	0.0007889
204732_s_at	373	-0.34	0.10	-3.51	0.0007896
202796_at	11346	0.35	0.10	3.51	0.0007906
211742_s_at	2124	0.40	0.11	3.50	0.0008054
204446_s_at	240	0.40	0.12	3.50	0.0008088
236862_at	57120	-0.49	0.14	-3.50	0.0008095
229384_at	NA	0.18	0.05	3.50	0.000816
221274_s_at	81562	-0.16	0.05	-3.49	0.0008415
223454_at	58191	0.34	0.10	3.49	0.0008419
227442_at	285521	0.14	0.04	3.49	0.0008419
219706_at	55317	0.18	0.05	3.49	0.000844
212922_s_at	56950	0.21	0.06	3.49	0.0008516
241955_at	25831	-0.45	0.13	-3.48	0.0008608
231906_at	3234	-0.19	0.06	-3.48	0.0008618

232978_at	10150	-0.37	0.11	-3.48	0.0008631
216235_s_at	1909	-0.35	0.10	-3.48	0.0008639
222787_s_at	54664	-0.39	0.11	-3.48	0.00087
205995_x_at	9657	0.13	0.04	3.48	0.0008719
229586_at	80205	-0.25	0.07	-3.48	0.0008751
200625_s_at	10487	0.13	0.04	3.48	0.0008751
218243_at	80230	0.15	0.04	3.48	0.0008786
244592_at	2313	-0.33	0.09	-3.47	0.0008914
228845_at	196463	0.26	0.07	3.47	0.0009083
212071_s_at	56969	0.15	0.04	3.47	0.0009089
225975_at	54510	-0.25	0.07	-3.46	0.0009184
211503_s_at	51552	-0.24	0.07	-3.46	0.0009198
212249_at	5295	-0.40	0.12	-3.46	0.0009202
235811_at	4154	-0.45	0.13	-3.46	0.0009212
213418_at	3310	0.62	0.18	3.46	0.0009221
205147_x_at	4689	0.39	0.11	3.46	0.0009227
211074_at	2348	-0.53	0.15	-3.46	0.0009257
214791_at	93349	0.19	0.05	3.46	0.0009274
223940_x_at	378938	-0.67	0.19	-3.46	0.000928
205499_at	27286	0.42	0.12	3.45	0.0009439
210943_s_at	1130	-0.39	0.11	-3.45	0.0009477
219999_at	4122	0.29	0.08	3.45	0.0009481
242343_x_at	9849	-0.37	0.11	-3.45	0.000949
208998_at	7351	0.43	0.12	3.45	0.0009513
209522_s_at	1384	0.20	0.06	3.45	0.0009552
238722_x_at	222236	-0.22	0.06	-3.45	0.0009662
213215_at	10239	0.23	0.07	3.44	0.0009716
213015_at	56987	-0.33	0.09	-3.44	0.0009812
202812_at	2548	0.22	0.06	3.44	0.0009826
240399_at	7204	-0.19	0.06	-3.44	0.0009831
221059_s_at	23406	0.32	0.09	3.44	0.0009922
233449_at	NA	-0.31	0.09	-3.44	0.0009949
235213_at	3707	-0.33	0.10	-3.44	0.0009968
203047_at	6793	0.22	0.07	3.43	0.0010041
40420_at	6793	0.21	0.06	3.43	0.0010095
214375_at	8496	-0.32	0.09	-3.42	0.0010394
232834_at	NA	-0.21	0.06	-3.42	0.0010432
227778_at	377711	0.21	0.06	3.42	0.0010587
202974_at	4354	0.23	0.07	3.42	0.001065
217878_s_at	996	-0.22	0.06	-3.41	0.0010673
201910_at	10160	0.16	0.05	3.41	0.0010762
207085_x_at	1438	0.62	0.18	3.41	0.0010769
231959_at	91750	-0.18	0.05	-3.41	0.0010796
208919_s_at	65220	0.18	0.05	3.41	0.0010872
235435_at	132949	-0.37	0.11	-3.40	0.0011053
228160_at	400642	0.33	0.10	3.40	0.0011067
203839_s_at	10188	0.18	0.05	3.40	0.0011072
210042_s_at	1522	0.37	0.11	3.40	0.0011085
206848_at	3204	-0.27	0.08	-3.40	0.0011107
221050_s_at	54676	0.17	0.05	3.40	0.0011108
202756_s_at	2817	0.34	0.10	3.40	0.0011213
226976_at	23633	0.20	0.06	3.40	0.001125

235623_at	55250	-0.17	0.05	-3.39	0.001144
201125_s_at	3693	0.21	0.06	3.39	0.0011456
202477_s_at	10844	0.13	0.04	3.39	0.00115
203206_at	9679	0.17	0.05	3.39	0.001153
202845_s_at	10928	0.14	0.04	3.39	0.0011566
211858_x_at	2778	0.12	0.04	3.39	0.0011568
218017_s_at	138050	0.17	0.05	3.39	0.0011573
239955_at	NA	-0.34	0.10	-3.39	0.0011577
226252_at	NA	-0.28	0.08	-3.39	0.0011609
218223_s_at	51177	0.26	0.08	3.38	0.0011808
226957_x_at	10928	0.17	0.05	3.38	0.0011856
213204_at	23113	0.18	0.05	3.38	0.0012057
223712_at	84105	0.16	0.05	3.37	0.0012235
201917_s_at	55186	-0.43	0.13	-3.37	0.0012282
222665_at	51115	0.16	0.05	3.37	0.0012284
210776_x_at	6929	0.13	0.04	3.36	0.0012507
203718_at	10908	0.24	0.07	3.36	0.0012581
241508_at	23253	-0.30	0.09	-3.36	0.0012634
223031_s_at	84231	0.17	0.05	3.36	0.0012642
204788_s_at	5498	0.16	0.05	3.36	0.0012671
242108_at	53373	-0.24	0.07	-3.36	0.0012739
208757_at	54732	-0.17	0.05	-3.36	0.0012767
218473_s_at	79709	0.15	0.05	3.36	0.0012771
204480_s_at	79095	0.21	0.06	3.36	0.0012784
221016_s_at	83439	0.27	0.08	3.35	0.0012866
224622_at	57533	0.12	0.03	3.35	0.0013193
226445_s_at	90933	0.14	0.04	3.34	0.0013301
228409_at	114782	0.43	0.13	3.34	0.0013337
204303_s_at	9811	0.39	0.12	3.34	0.0013349
218843_at	64838	0.22	0.07	3.34	0.0013433
236664_at	NA	0.27	0.08	3.34	0.0013605
210666_at	3423	-0.15	0.05	-3.33	0.0013741
242349_at	25831	-0.24	0.07	-3.33	0.0013857
237400_at	27109	0.27	0.08	3.32	0.0014184
204463_s_at	1909	-0.44	0.13	-3.32	0.0014232
214352_s_at	3265	-0.27	0.08	-3.32	0.0014245
212873_at	23526	0.25	0.07	3.32	0.0014395
223161_at	57189	-0.27	0.08	-3.32	0.0014445
215336_at	11215	-0.29	0.09	-3.32	0.0014454
226301_at	116843	0.26	0.08	3.31	0.0014548
227853_at	196463	0.33	0.10	3.31	0.0014653
224768_at	55677	0.15	0.05	3.30	0.0015019
59625_at	8996	0.26	0.08	3.30	0.0015105
214767_s_at	126393	0.52	0.16	3.30	0.0015177
35626_at	6448	0.19	0.06	3.30	0.0015205
226219_at	257106	0.42	0.13	3.30	0.0015244
210809_s_at	10631	0.30	0.09	3.30	0.0015259
228900_at	NA	0.26	0.08	3.30	0.001545
222116_s_at	125058	0.30	0.09	3.29	0.0015511
242471_at	NA	-0.33	0.10	-3.28	0.0016091
209667_at	8824	0.24	0.07	3.28	0.0016123
227501_at	26118	-0.24	0.07	-3.28	0.0016229

226880_at	64710	-0.22	0.07	-3.28	0.0016239
225360_at	80305	0.17	0.05	3.28	0.0016312
217796_s_at	55666	0.25	0.08	3.28	0.0016414
210740_s_at	3705	0.27	0.08	3.28	0.001643
201580_s_at	56255	-0.26	0.08	-3.28	0.0016435
223455_at	84260	0.16	0.05	3.27	0.0016528
211824_x_at	22861	0.23	0.07	3.27	0.0016688
209171_at	3704	0.12	0.04	3.27	0.0016723
200670_at	7494	-0.26	0.08	-3.27	0.001675
227931_at	NA	-0.44	0.13	-3.27	0.0016782
226542_at	11237	0.29	0.09	3.27	0.0016839
209530_at	784	0.19	0.06	3.27	0.0016876
225347_at	127829	0.15	0.04	3.27	0.0016943
242025_at	6660	-0.38	0.12	-3.26	0.0016974
226997_at	NA	0.28	0.09	3.26	0.0017035
202124_s_at	66008	-0.26	0.08	-3.26	0.0017085
222052_at	284325	0.20	0.06	3.26	0.0017186
226422_at	51290	-0.16	0.05	-3.26	0.0017254
225051_at	2035	0.24	0.07	3.26	0.0017294
204535_s_at	5978	-0.17	0.05	-3.26	0.0017337
219919_s_at	54961	0.18	0.06	3.26	0.0017365
220065_at	64102	0.67	0.21	3.25	0.0017512
203555_at	26469	0.20	0.06	3.25	0.0017544
201842_s_at	2202	0.19	0.06	3.25	0.0017621
218296_x_at	55154	0.29	0.09	3.25	0.0017672
225890_at	92667	0.21	0.06	3.25	0.0017739
203286_at	22838	0.20	0.06	3.25	0.0017888
208885_at	3936	0.38	0.12	3.25	0.001791
226696_at	10741	0.12	0.04	3.25	0.0017927
201230_s_at	10425	0.14	0.04	3.25	0.0017947
212785_s_at	51574	0.16	0.05	3.25	0.0018008
212775_at	23363	0.25	0.08	3.25	0.0018009
229997_at	81839	-0.16	0.05	-3.24	0.0018057
223150_s_at	25930	0.24	0.07	3.24	0.0018318
232134_at	NA	-0.19	0.06	-3.24	0.001848
223802_s_at	5930	-0.24	0.07	-3.24	0.0018502
41047_at	79095	0.17	0.05	3.23	0.0018701
213279_at	115817	0.20	0.06	3.23	0.0018754
225640_at	401504	-0.31	0.10	-3.23	0.0018885
200660_at	6282	0.16	0.05	3.23	0.0018885
217805_at	3609	0.17	0.05	3.23	0.0018919
212134_at	23187	0.20	0.06	3.23	0.0018951
219505_at	51816	0.45	0.14	3.23	0.0018967
213373_s_at	841	0.26	0.08	3.23	0.001904
239891_x_at	201475	-0.16	0.05	-3.23	0.0019041
235010_at	NA	0.22	0.07	3.23	0.0019148
209217_s_at	11152	0.13	0.04	3.22	0.0019209
231017_at	6794	0.18	0.06	3.22	0.0019253
226053_at	5609	0.15	0.05	3.22	0.0019593
211982_x_at	23214	0.14	0.04	3.22	0.0019615
215001_s_at	2752	-0.28	0.09	-3.22	0.0019729
215735_s_at	7249	0.23	0.07	3.21	0.0020008

222633_at	79718	-0.47	0.15	-3.21	0.0020011
201830_s_at	10276	-0.26	0.08	-3.21	0.0020033
225251_at	53917	0.13	0.04	3.21	0.0020173
219626_at	79649	0.16	0.05	3.21	0.0020212
222728_s_at	79101	-0.21	0.06	-3.21	0.0020267
210172_at	7536	-0.40	0.13	-3.20	0.0020356
209060_x_at	8202	-0.25	0.08	-3.20	0.0020391
202692_s_at	7343	0.15	0.05	3.20	0.0020489
239937_at	7756	-0.26	0.08	-3.20	0.0020553
220319_s_at	29116	-0.20	0.06	-3.20	0.0020584
212969_x_at	256364	0.22	0.07	3.20	0.0020745
203907_s_at	9922	0.18	0.05	3.20	0.0020905
208705_s_at	1983	-0.16	0.05	-3.19	0.0021189
202913_at	9826	0.18	0.06	3.19	0.0021355
209889_at	25956	0.25	0.08	3.18	0.0021641
213939_s_at	22902	-0.30	0.09	-3.18	0.0021647
209087_x_at	4162	0.26	0.08	3.18	0.0021759
228647_at	4815	0.21	0.07	3.18	0.0021842
205529_s_at	862	-0.21	0.07	-3.18	0.0021888
223279_s_at	55075	0.17	0.05	3.18	0.0021889
202587_s_at	203	0.17	0.05	3.18	0.0021929
212390_at	9659	0.25	0.08	3.18	0.0021949
201606_s_at	11137	-0.20	0.06	-3.18	0.0022027
221526_x_at	56288	-0.15	0.05	-3.18	0.0022197
224943_at	55727	-0.25	0.08	-3.18	0.00222
232778_at	54906	-0.26	0.08	-3.18	0.0022205
213065_at	196441	0.13	0.04	3.17	0.0022315
218175_at	80212	0.25	0.08	3.17	0.0022336
204789_at	752	0.24	0.08	3.17	0.0022342
231873_at	659	-0.38	0.12	-3.17	0.0022423
205404_at	3290	0.52	0.16	3.17	0.0022482
209920_at	659	-0.19	0.06	-3.17	0.002276
219799_s_at	10170	0.40	0.13	3.17	0.0022773
227298_at	401264	0.14	0.04	3.17	0.0022849
225440_at	56894	0.27	0.08	3.17	0.0022956
243295_at	54439	-0.15	0.05	-3.16	0.0023119
226435_at	89932	0.35	0.11	3.16	0.0023132
219282_s_at	51393	0.24	0.08	3.16	0.0023202
227223_at	9584	-0.34	0.11	-3.16	0.0023275
213156_at	NA	-0.21	0.07	-3.16	0.0023392
208931_s_at	3609	0.18	0.06	3.16	0.002341
AFFX-M27830_M_at	NA	-0.20	0.06	-3.16	0.0023456
203442_x_at	256364	0.21	0.07	3.16	0.0023609
201332_s_at	6778	0.23	0.07	3.16	0.0023611
236620_at	55183	-0.36	0.11	-3.15	0.0023661
204257_at	3995	0.29	0.09	3.15	0.0023691
210720_s_at	63941	0.18	0.06	3.15	0.0023806
202952_s_at	8038	0.48	0.15	3.15	0.0023823
212401_s_at	985	0.16	0.05	3.15	0.0023878
202484_s_at	8932	0.20	0.06	3.15	0.0023944
212566_at	4134	0.20	0.06	3.15	0.0024067
239597_at	255967	-0.36	0.11	-3.15	0.0024144

39705_at	23309	0.19	0.06	3.15	0.0024246
227143_s_at	637	0.14	0.04	3.15	0.0024317
218971_s_at	29062	0.15	0.05	3.15	0.0024322
227592_at	126133	0.17	0.05	3.14	0.0024458
55081_at	85377	0.25	0.08	3.14	0.0024493
222462_s_at	23621	0.12	0.04	3.14	0.0024545
227400_at	4784	0.22	0.07	3.14	0.0024545
47608_at	93643	0.11	0.04	3.14	0.0024667
238970_at	51663	-0.37	0.12	-3.14	0.0024777
221735_at	57599	-0.15	0.05	-3.14	0.0024832
232784_at	6645	-0.20	0.06	-3.14	0.0024845
213160_at	1794	0.32	0.10	3.14	0.0024936
202197_at	8897	0.14	0.05	3.14	0.0024941
221030_s_at	83478	0.22	0.07	3.14	0.0025005
200808_s_at	7791	0.29	0.09	3.13	0.0025287
228213_at	55766	0.23	0.07	3.13	0.0025354
242233_at	57148	-0.30	0.10	-3.13	0.0025422
212104_s_at	23543	-0.19	0.06	-3.13	0.0025523
228094_at	120425	0.24	0.08	3.13	0.0025554
203573_s_at	5875	0.24	0.08	3.13	0.0025554
219543_at	64081	0.26	0.08	3.13	0.0025676
238668_at	3071	0.36	0.12	3.13	0.0025693
204964_s_at	8082	-0.35	0.11	-3.13	0.0025694
228866_at	29123	-0.19	0.06	-3.13	0.0025699
235396_at	128989	0.15	0.05	3.13	0.0025705
228532_at	128346	0.36	0.11	3.13	0.0025795
207992_s_at	272	0.32	0.10	3.12	0.0025924
201070_x_at	23451	-0.25	0.08	-3.12	0.0025952
232935_at	10186	-0.48	0.15	-3.12	0.0026053
232662_x_at	84293	0.34	0.11	3.12	0.0026058
232458_at	1281	-0.40	0.13	-3.12	0.0026096
227402_s_at	84294	-0.17	0.05	-3.12	0.0026099
207838_x_at	57326	0.23	0.07	3.12	0.0026353
225664_at	1303	0.19	0.06	3.12	0.0026379
209953_s_at	11140	0.18	0.06	3.12	0.0026523
219680_at	79671	0.15	0.05	3.11	0.0026857
209178_at	9785	0.16	0.05	3.11	0.0026927
223423_at	26996	0.31	0.10	3.11	0.0026972
218682_s_at	22950	0.11	0.04	3.11	0.0026978
226152_at	145567	0.27	0.09	3.11	0.0027151
200785_s_at	4035	0.28	0.09	3.11	0.002716
226371_at	5927	-0.19	0.06	-3.11	0.0027166
202916_s_at	9917	0.13	0.04	3.11	0.0027214
50374_at	339229	0.14	0.05	3.11	0.0027311
212330_at	7027	0.11	0.03	3.11	0.0027367
238317_x_at	5937	-0.34	0.11	-3.10	0.0027505
214004_s_at	9686	-0.16	0.05	-3.10	0.0027524
223125_s_at	81563	-0.23	0.07	-3.10	0.0027671
205497_at	7728	-0.13	0.04	-3.10	0.0027692
212830_at	1955	0.29	0.09	3.10	0.0027728
239978_at	23760	-0.25	0.08	-3.10	0.0027867
226304_at	126393	0.44	0.14	3.10	0.0027937

208713_at	11100	0.18	0.06	3.10	0.002795
201738_at	10289	-0.13	0.04	-3.10	0.0027979
203879_at	5293	0.21	0.07	3.10	0.0027997
219090_at	57419	0.42	0.14	3.10	0.0028051
219314_s_at	51222	0.23	0.07	3.10	0.002809
203215_s_at	4646	-0.30	0.10	-3.10	0.0028123
209380_s_at	10057	0.22	0.07	3.10	0.0028185
241997_at	NA	-0.17	0.05	-3.10	0.0028199
216080_s_at	3995	0.34	0.11	3.09	0.0028353
212860_at	84243	0.16	0.05	3.09	0.0028439
241681_at	4154	-0.40	0.13	-3.09	0.0028482
203592_s_at	10272	0.29	0.09	3.09	0.0028587
236241_at	51003	-0.32	0.10	-3.09	0.0028653
225512_at	253461	-0.20	0.07	-3.09	0.0028679
211980_at	1282	0.24	0.08	3.09	0.002869
213328_at	4750	-0.22	0.07	-3.09	0.0028781
212601_at	23140	0.19	0.06	3.09	0.0028976
226436_at	83937	0.30	0.10	3.09	0.002908
203426_s_at	3488	-0.21	0.07	-3.08	0.0029259
201124_at	3693	0.23	0.07	3.08	0.0029279
224939_at	57532	-0.21	0.07	-3.08	0.0029319
210627_s_at	7841	0.22	0.07	3.08	0.0029374
202681_at	7375	0.12	0.04	3.08	0.0029376
218838_s_at	64427	0.14	0.05	3.08	0.0029404
222175_s_at	51586	0.19	0.06	3.08	0.0029442
223406_x_at	80011	0.11	0.04	3.08	0.0029686
227930_at	192670	0.14	0.05	3.08	0.0029697
216237_s_at	4174	0.18	0.06	3.08	0.0029713
209834_at	9469	0.25	0.08	3.08	0.0029825
241789_at	27303	-0.33	0.11	-3.08	0.0029919
243963_at	10806	-0.28	0.09	-3.07	0.0030021
222111_at	54629	-0.26	0.09	-3.07	0.0030127
203775_at	10165	0.18	0.06	3.07	0.0030249
244674_at	10180	-0.28	0.09	-3.07	0.003029
228857_at	285831	0.19	0.06	3.07	0.0030615
214784_x_at	23214	0.13	0.04	3.07	0.0030623
222789_at	54665	-0.20	0.06	-3.07	0.0030708
220272_at	54796	-0.23	0.08	-3.07	0.0030856
209048_s_at	23613	0.19	0.06	3.07	0.0030901
211964_at	1284	0.28	0.09	3.06	0.0030938
229858_at	26057	-0.34	0.11	-3.06	0.0030984
226381_at	NA	0.16	0.05	3.06	0.0031169
226999_at	55599	-0.28	0.09	-3.06	0.003117
213720_s_at	6597	0.25	0.08	3.06	0.0031302
212054_x_at	23061	0.17	0.06	3.06	0.0031346
52005_at	58525	0.19	0.06	3.06	0.0031488
202153_s_at	23636	0.18	0.06	3.06	0.0031515
221829_s_at	3842	-0.33	0.11	-3.06	0.0031583
226412_at	25957	-0.31	0.10	-3.06	0.0031695
203656_at	9896	0.12	0.04	3.06	0.0031809
218699_at	8934	0.20	0.07	3.05	0.0031856
204366_s_at	2976	0.13	0.04	3.05	0.0031879

218547_at	79947	0.28	0.09	3.05	0.0032055
217617_at	5087	-0.37	0.12	-3.05	0.0032057
228253_at	84695	0.19	0.06	3.05	0.0032102
212493_s_at	29072	0.15	0.05	3.05	0.0032239
212053_at	23042	0.10	0.03	3.05	0.0032242
212856_at	23151	0.19	0.06	3.05	0.0032322
220597_s_at	51329	0.11	0.04	3.05	0.0032372
205528_s_at	862	-0.22	0.07	-3.05	0.0032473
223592_s_at	84282	0.13	0.04	3.05	0.0032666
201846_s_at	23429	-0.20	0.06	-3.04	0.0032793
213045_at	23031	0.22	0.07	3.04	0.0032916
215338_s_at	4820	-0.27	0.09	-3.04	0.0033016
228083_at	93589	0.22	0.07	3.04	0.0033118
202568_s_at	4140	0.10	0.03	3.04	0.0033134
224925_at	57580	0.20	0.07	3.04	0.0033193
208776_at	5717	0.20	0.07	3.04	0.003321
227274_at	55333	0.14	0.05	3.04	0.003336
235879_at	4154	-0.44	0.14	-3.04	0.0033389
222482_at	23648	0.16	0.05	3.04	0.0033502
222791_at	54665	-0.44	0.15	-3.04	0.0033502
201908_at	1857	0.22	0.07	3.04	0.0033571
242144_at	167153	-0.33	0.11	-3.04	0.0033675
209113_s_at	10362	0.18	0.06	3.03	0.003384
209166_s_at	4125	0.23	0.08	3.03	0.0033946
207657_x_at	3842	-0.31	0.10	-3.03	0.003414
222131_x_at	89941	0.14	0.05	3.03	0.0034315
203057_s_at	7799	0.15	0.05	3.03	0.0034364
200766_at	1509	0.29	0.10	3.03	0.0034538
218697_at	51517	0.17	0.06	3.03	0.0034685
203085_s_at	7040	0.28	0.09	3.03	0.0034693
205315_s_at	6645	-0.20	0.07	-3.03	0.0034726
222790_s_at	54665	-0.33	0.11	-3.03	0.0034735
201829_at	10276	-0.27	0.09	-3.02	0.0034885
230200_at	221078	-0.19	0.06	-3.02	0.0034977
228120_at	26523	0.20	0.07	3.02	0.0035067
222850_s_at	79982	-0.29	0.10	-3.02	0.0035146
218080_x_at	11124	0.11	0.04	3.02	0.003518
217802_s_at	64710	-0.12	0.04	-3.02	0.0035248
213666_at	23157	0.17	0.05	3.02	0.0035394
232221_x_at	222194	-0.15	0.05	-3.02	0.0035459
51200_at	55049	0.14	0.05	3.02	0.0035514
202378_s_at	54741	-0.25	0.08	-3.02	0.0035645
203769_s_at	412	0.31	0.10	3.02	0.0035744
223194_s_at	63027	0.21	0.07	3.01	0.0036143
239545_at	64921	-0.39	0.13	-3.01	0.0036157
210463_x_at	55621	0.17	0.05	3.01	0.0036182
229394_s_at	2909	0.14	0.05	3.01	0.0036267
231990_at	9958	0.22	0.07	3.01	0.003651
229468_at	1018	0.17	0.06	3.01	0.0036677
213244_at	113178	0.23	0.08	3.01	0.0036716
201182_s_at	1108	-0.21	0.07	-3.01	0.0036721
40149_at	25970	0.15	0.05	3.01	0.0036734

201025_at	9669	0.19	0.06	3.00	0.0036955
225080_at	4641	0.25	0.08	3.00	0.0037008
210461_s_at	3983	-0.33	0.11	-3.00	0.0037074
236216_at	6934	-0.16	0.05	-3.00	0.0037241
227336_at	1840	0.28	0.09	3.00	0.0037333
223907_s_at	54984	-0.12	0.04	-3.00	0.0037475
227962_at	51	0.30	0.10	3.00	0.0037687
226468_at	27246	0.13	0.04	3.00	0.0037741
207196_s_at	10318	0.30	0.10	3.00	0.0037773
202275_at	2539	0.24	0.08	2.99	0.0037938
229958_at	619435	0.23	0.08	2.99	0.0038003
221734_at	133619	-0.15	0.05	-2.99	0.0038119
223187_s_at	94101	0.15	0.05	2.99	0.0038198
230212_at	10252	-0.17	0.06	-2.99	0.003838
218803_at	55743	0.16	0.05	2.99	0.0038392
243286_at	8454	-0.26	0.09	-2.99	0.003841
229810_at	NA	0.23	0.08	2.99	0.0038583
202249_s_at	50717	0.13	0.04	2.99	0.0038587
218822_s_at	79716	0.16	0.05	2.99	0.003862
214181_x_at	7940	0.26	0.09	2.99	0.0038735
201101_s_at	9774	-0.26	0.09	-2.99	0.003897
207428_x_at	985	0.18	0.06	2.98	0.003907
46256_at	90864	0.14	0.05	2.98	0.0039112
201885_s_at	1727	0.14	0.05	2.98	0.0039286
226324_s_at	26160	0.15	0.05	2.98	0.0039294
228105_at	55291	-0.35	0.12	-2.98	0.0039295
229145_at	119504	0.17	0.06	2.98	0.0039308
203124_s_at	4891	0.19	0.06	2.98	0.0039348
219191_s_at	51411	0.26	0.09	2.98	0.0039519
212076_at	4297	-0.15	0.05	-2.98	0.0039574
203503_s_at	5195	0.18	0.06	2.98	0.0039606
214718_at	57798	-0.18	0.06	-2.98	0.0039637
227120_at	116113	0.28	0.09	2.98	0.0039707
212780_at	6654	0.29	0.10	2.98	0.0039759
242646_at	51111	-0.17	0.06	-2.98	0.0039765
212758_s_at	6935	-0.24	0.08	-2.98	0.0040099
209949_at	4688	0.32	0.11	2.97	0.004019
228040_at	389741	-0.24	0.08	-2.97	0.0040192
229144_at	23254	0.20	0.07	2.97	0.0040199
226728_at	376497	0.20	0.07	2.97	0.0040208
201058_s_at	10398	0.22	0.08	2.97	0.0040565
228545_at	7707	-0.35	0.12	-2.97	0.0040612
242021_at	NA	-0.22	0.07	-2.97	0.004063
209179_s_at	79143	0.17	0.06	2.97	0.0040698
213733_at	4542	0.31	0.11	2.97	0.0040723
242849_at	23328	-0.28	0.09	-2.97	0.0040784
219281_at	4482	0.15	0.05	2.97	0.0040869
222158_s_at	51029	-0.33	0.11	-2.97	0.0041112
221270_s_at	81890	0.16	0.05	2.97	0.0041123
203894_at	27175	0.22	0.07	2.97	0.0041133
202644_s_at	7128	0.17	0.06	2.97	0.0041222
221794_at	57572	0.27	0.09	2.97	0.0041312

207788_s_at	10174	0.25	0.08	2.97	0.0041352
236144_at	119587	-0.27	0.09	-2.96	0.0041398
204642_at	1901	-0.16	0.05	-2.96	0.0041452
218164_at	64847	0.22	0.07	2.96	0.0041498
229436_x_at	79184	0.11	0.04	2.96	0.0041545
225451_at	56850	0.14	0.05	2.96	0.0041605
32094_at	9469	0.21	0.07	2.96	0.0041696
37996_s_at	1760	0.25	0.08	2.96	0.0041865
220980_s_at	83440	0.15	0.05	2.96	0.0041995
243993_at	5128	-0.33	0.11	-2.96	0.0042138
212512_s_at	10498	0.22	0.07	2.96	0.0042229
219460_s_at	55654	0.12	0.04	2.96	0.0042253
235322_at	388526	-0.16	0.05	-2.96	0.00425
210807_s_at	9194	-0.47	0.16	-2.96	0.004254
226858_at	1454	0.19	0.06	2.95	0.0042636
208632_at	9921	0.18	0.06	2.95	0.0042643
217782_s_at	2873	0.14	0.05	2.95	0.0042709
200762_at	1808	-0.14	0.05	-2.95	0.0042871
218447_at	56942	0.20	0.07	2.95	0.0043153
221235_s_at	9392	0.14	0.05	2.95	0.0043449
227062_at	283131	-0.32	0.11	-2.95	0.004349
203181_x_at	6733	-0.17	0.06	-2.95	0.0043548
232543_x_at	64333	0.30	0.10	2.95	0.0043552
228937_at	144811	0.24	0.08	2.94	0.0043862
225157_at	22877	0.19	0.07	2.94	0.0044171
217943_s_at	55700	0.33	0.11	2.94	0.0044232
221927_s_at	83451	0.13	0.04	2.94	0.0044242
231773_at	9068	-0.31	0.11	-2.94	0.0044283
221985_at	54800	-0.29	0.10	-2.94	0.0044289
227833_s_at	114785	0.21	0.07	2.94	0.004482
225969_at	84964	0.15	0.05	2.94	0.004503
203701_s_at	55621	0.17	0.06	2.93	0.0045255
219452_at	64174	0.28	0.10	2.93	0.0045259
203355_s_at	23362	-0.42	0.14	-2.93	0.0045281
219648_at	55686	0.34	0.12	2.93	0.0045506
212052_s_at	23061	0.18	0.06	2.93	0.0045972
238596_at	118924	-0.12	0.04	-2.93	0.0046
202791_s_at	9701	0.12	0.04	2.93	0.0046024
226440_at	56940	0.12	0.04	2.93	0.0046115
202650_s_at	9772	0.18	0.06	2.93	0.0046143
218148_at	80152	0.19	0.06	2.93	0.0046209
209929_s_at	8517	0.14	0.05	2.93	0.0046368
214748_at	88523	0.20	0.07	2.92	0.0046402
212240_s_at	5295	-0.30	0.10	-2.92	0.0046683
213742_at	9295	-0.37	0.13	-2.92	0.0046888
213032_at	4781	-0.23	0.08	-2.92	0.0047019
223439_at	79576	0.14	0.05	2.92	0.0047124
226207_at	353116	0.14	0.05	2.92	0.0047401
236752_at	8502	-0.35	0.12	-2.92	0.0047454
221677_s_at	29980	0.20	0.07	2.92	0.0047463
217856_at	9939	0.20	0.07	2.92	0.0047504
202250_s_at	50717	0.16	0.05	2.92	0.0047642

228356_at	29123	0.18	0.06	2.92	0.0047652
227565_at	51088	0.22	0.07	2.92	0.0047679
215773_x_at	10038	0.15	0.05	2.91	0.0047887
219507_at	51319	-0.20	0.07	-2.91	0.0048032
221046_s_at	29083	-0.21	0.07	-2.91	0.004823
219646_at	54849	0.16	0.05	2.91	0.0048397
34221_at	22993	0.13	0.04	2.91	0.0048507
232311_at	567	-0.27	0.09	-2.91	0.0048777
242225_at	23041	-0.29	0.10	-2.91	0.0048828
226846_at	254295	0.18	0.06	2.91	0.0048833
211707_s_at	9657	0.10	0.04	2.91	0.0048879
201593_s_at	55854	-0.17	0.06	-2.91	0.0048904
202754_at	23518	0.16	0.06	2.91	0.0049003
219394_at	9489	0.12	0.04	2.91	0.0049009
212715_s_at	57553	0.18	0.06	2.91	0.0049034
211779_x_at	161	0.15	0.05	2.91	0.0049091
229101_at	150166	0.23	0.08	2.90	0.00492
226824_at	119587	-0.29	0.10	-2.90	0.0049613
230235_at	NA	0.18	0.06	2.90	0.004967
226883_at	55175	-0.20	0.07	-2.90	0.004967
235085_at	157285	0.27	0.09	2.90	0.0049813
228077_at	6787	0.23	0.08	2.90	0.0049841
212024_x_at	2314	0.13	0.04	2.90	0.0049857
218988_at	55508	0.11	0.04	2.90	0.0050008
222139_at	57612	0.43	0.15	2.90	0.0050079
229353_s_at	64710	-0.19	0.06	-2.90	0.0050247
203753_at	6925	-0.20	0.07	-2.90	0.0050306
203412_at	8216	0.15	0.05	2.90	0.0050488
224887_at	84572	0.12	0.04	2.89	0.0050626
227622_at	51585	0.17	0.06	2.89	0.0050668
211271_x_at	5725	0.17	0.06	2.89	0.0050702
213701_at	91298	-0.21	0.07	-2.89	0.0051217
236395_at	4208	-0.41	0.14	-2.89	0.0051228
211340_s_at	4162	0.21	0.07	2.89	0.0051286
223931_s_at	55743	0.13	0.05	2.89	0.0051312
221897_at	84851	0.15	0.05	2.89	0.0051324
201503_at	10146	-0.24	0.08	-2.89	0.0051359
204181_s_at	23099	-0.14	0.05	-2.89	0.0051523
214719_at	283537	0.16	0.06	2.89	0.0051561
57539_at	84619	0.16	0.05	2.89	0.0051641
212820_at	23312	0.21	0.07	2.89	0.005196
207168_s_at	9555	0.11	0.04	2.88	0.0052155
204858_s_at	1890	0.27	0.09	2.88	0.0052263
218107_at	80232	0.12	0.04	2.88	0.0052266
225904_at	126731	0.21	0.07	2.88	0.0052332
201752_s_at	120	-0.25	0.09	-2.88	0.0052382
243_g_at	4134	0.18	0.06	2.88	0.0052413
212382_at	6925	-0.31	0.11	-2.88	0.0052524
212567_s_at	4134	0.18	0.06	2.88	0.005255
217908_s_at	55827	0.16	0.06	2.88	0.0052619
225146_at	203259	0.16	0.06	2.88	0.005265
226595_at	90203	0.14	0.05	2.88	0.0052709

205786_s_at	3684	0.30	0.11	2.88	0.0052887
213349_at	23023	0.16	0.06	2.88	0.0052961
227144_at	23313	0.20	0.07	2.88	0.005298
206959_s_at	65110	0.14	0.05	2.88	0.0052987
205226_at	5157	-0.43	0.15	-2.88	0.0053008
200842_s_at	2058	-0.20	0.07	-2.88	0.0053126
218581_at	63874	0.18	0.06	2.88	0.005313
217754_at	54606	0.12	0.04	2.88	0.0053223
201199_s_at	5707	0.12	0.04	2.88	0.0053419
222357_at	26137	-0.28	0.10	-2.87	0.0053591
218144_s_at	64423	0.21	0.07	2.87	0.0053611
223226_x_at	170463	0.16	0.05	2.87	0.0053673
244533_at	5784	-0.23	0.08	-2.87	0.0053676
219582_at	79627	0.21	0.07	2.87	0.0054067
219242_at	80254	0.14	0.05	2.87	0.0054196
203506_s_at	9968	0.21	0.07	2.87	0.0054319
227112_at	23023	0.19	0.07	2.87	0.0054545
203257_s_at	79096	0.11	0.04	2.87	0.005464
225020_at	153090	0.21	0.07	2.86	0.0055284
213136_at	5771	0.11	0.04	2.86	0.0055385
203920_at	10062	0.18	0.06	2.86	0.0055447
219033_at	79668	0.21	0.08	2.86	0.0055577
205258_at	3625	0.30	0.10	2.86	0.0055751
213043_s_at	9862	0.18	0.06	2.86	0.0055764
224076_s_at	54904	0.10	0.04	2.86	0.0055795
210785_s_at	9473	0.31	0.11	2.86	0.0055835
201986_at	9969	-0.21	0.07	-2.86	0.0056064
225258_at	54751	0.21	0.07	2.86	0.0056076
203488_at	22859	0.20	0.07	2.86	0.0056198
214853_s_at	6464	0.14	0.05	2.86	0.0056294
200661_at	5476	0.18	0.06	2.86	0.0056322
221216_s_at	22955	0.17	0.06	2.86	0.0056525
209267_s_at	64116	0.39	0.14	2.85	0.0056714
235181_at	129450	0.12	0.04	2.85	0.0056743
202803_s_at	3689	0.46	0.16	2.85	0.0056778
213334_x_at	11219	0.12	0.04	2.85	0.0056838
210764_s_at	3491	0.44	0.15	2.85	0.0056854
202255_s_at	26037	0.17	0.06	2.85	0.0056883
232174_at	2131	-0.35	0.12	-2.85	0.0056978
233417_at	54622	-0.38	0.13	-2.85	0.0057396
221766_s_at	55603	-0.33	0.12	-2.85	0.005761
205546_s_at	7297	0.19	0.07	2.85	0.0057898
203298_s_at	3720	0.15	0.05	2.85	0.005816
223049_at	2885	0.14	0.05	2.84	0.0058257
219689_at	56920	0.38	0.13	2.84	0.0058293
202452_at	10444	0.15	0.05	2.84	0.0058358
224567_x_at	378938	-0.24	0.08	-2.84	0.0058369
211780_x_at	1639	0.14	0.05	2.84	0.0058486
229746_x_at	10153	0.19	0.07	2.84	0.0058659
200788_s_at	8682	0.15	0.05	2.84	0.0058897
224912_at	57217	0.16	0.05	2.84	0.0058989
221276_s_at	81493	0.39	0.14	2.84	0.0058991

212863_x_at	1487	0.14	0.05	2.84	0.0059069
202729_s_at	4052	-0.35	0.12	-2.84	0.005911
226465_s_at	6651	-0.18	0.06	-2.84	0.0059117
227518_at	79939	0.14	0.05	2.84	0.0059388
212895_s_at	29	0.14	0.05	2.84	0.005954
203274_at	474383	0.22	0.08	2.84	0.0059548
201632_at	1967	0.08	0.03	2.84	0.005981
225454_at	115098	0.19	0.07	2.83	0.0059938
228410_at	139716	0.14	0.05	2.83	0.0059939
226518_at	83892	0.13	0.04	2.83	0.0060023
235482_at	400960	-0.19	0.07	-2.83	0.0060127
218844_at	80221	0.21	0.08	2.83	0.0060235
212273_x_at	2778	0.11	0.04	2.83	0.0060373
232615_at	388685	-0.39	0.14	-2.83	0.0060387
217813_s_at	10927	-0.31	0.11	-2.83	0.0060387
235884_at	NA	0.25	0.09	2.83	0.0060478
226052_at	23476	0.16	0.06	2.83	0.0060587
214499_s_at	9774	-0.25	0.09	-2.83	0.0060608
202572_s_at	22839	0.24	0.08	2.83	0.0060681
218669_at	57826	0.14	0.05	2.83	0.0061006
226924_at	400657	0.17	0.06	2.83	0.0061016
224896_s_at	150465	0.14	0.05	2.83	0.0061113
209933_s_at	11314	0.27	0.10	2.83	0.0061139
203665_at	3162	0.28	0.10	2.83	0.0061143
218660_at	8291	0.21	0.07	2.83	0.0061227
203254_s_at	7094	0.30	0.11	2.83	0.0061331
224905_at	80232	0.17	0.06	2.83	0.0061376
223129_x_at	29116	-0.17	0.06	-2.83	0.0061445
223186_at	387521	0.22	0.08	2.83	0.0061449
219114_at	51161	0.13	0.05	2.83	0.0061477
221511_x_at	9236	0.21	0.07	2.83	0.0061509
203477_at	1306	0.16	0.06	2.82	0.0061792
201500_s_at	6992	0.13	0.04	2.82	0.006202
33850_at	4134	0.17	0.06	2.82	0.0062391
236109_at	84881	-0.23	0.08	-2.82	0.0062579
202625_at	4067	0.20	0.07	2.82	0.0062699
235041_at	9570	-0.18	0.06	-2.82	0.0062826
233411_at	5583	-0.16	0.06	-2.82	0.0062887
212451_at	9728	-0.19	0.07	-2.82	0.0062894
231809_x_at	10081	-0.16	0.06	-2.82	0.0062917
228817_at	NA	0.19	0.07	2.82	0.0063057
240383_at	7323	-0.34	0.12	-2.82	0.0063281
226040_at	81844	0.21	0.07	2.81	0.0063324
203384_s_at	2800	0.14	0.05	2.81	0.0063495
233315_at	1488	-0.17	0.06	-2.81	0.0063502
202747_s_at	9452	-0.35	0.12	-2.81	0.0063605
208686_s_at	6046	0.18	0.06	2.81	0.0064121
225311_at	3712	0.14	0.05	2.81	0.006421
202545_at	5580	0.38	0.14	2.81	0.0064282
209539_at	9459	0.20	0.07	2.81	0.0064287
217604_at	23549	0.23	0.08	2.81	0.0064314
232670_at	149401	-0.22	0.08	-2.81	0.0064319

210136_at	4155	-0.32	0.11	-2.81	0.0064369
206061_s_at	23405	-0.32	0.11	-2.81	0.0064496
235458_at	84868	0.35	0.12	2.81	0.0064659
200937_s_at	6125	-0.16	0.06	-2.81	0.0065031
218248_at	63901	0.15	0.05	2.80	0.0065153
225326_at	54439	-0.23	0.08	-2.80	0.0065204
209054_s_at	7468	0.12	0.04	2.80	0.0065331
224631_at	80829	-0.20	0.07	-2.80	0.006536
203392_s_at	1487	0.21	0.08	2.80	0.0065534
210859_x_at	1201	0.14	0.05	2.80	0.0065619
222154_s_at	26010	0.14	0.05	2.80	0.0065687
224792_at	85456	0.18	0.06	2.80	0.0065855
226250_at	NA	-0.22	0.08	-2.80	0.006619
217961_at	54977	-0.13	0.05	-2.80	0.0066302
230320_at	84897	0.15	0.05	2.80	0.0066395
202669_s_at	1948	-0.22	0.08	-2.80	0.00664
227935_s_at	84333	0.30	0.11	2.80	0.0066446
220041_at	80235	0.23	0.08	2.80	0.0066761
201129_at	6432	-0.24	0.08	-2.80	0.0066826
225104_at	90850	0.20	0.07	2.80	0.0066833
201387_s_at	7345	0.47	0.17	2.79	0.0066931
240221_at	1452	-0.25	0.09	-2.79	0.0067334
205659_at	9734	0.21	0.07	2.79	0.006763
209489_at	10658	-0.16	0.06	-2.79	0.0067921
236836_at	6902	-0.31	0.11	-2.79	0.0068022
226758_at	51631	0.09	0.03	2.79	0.0068545
210210_at	9019	-0.16	0.06	-2.79	0.0068626
206176_at	654	0.25	0.09	2.79	0.0068788
212205_at	94239	-0.12	0.04	-2.78	0.0068881
203262_s_at	9130	0.17	0.06	2.78	0.0069206
209271_at	2186	0.12	0.04	2.78	0.0069359
38149_at	9938	0.17	0.06	2.78	0.0069499
201810_s_at	9467	-0.13	0.05	-2.78	0.0069574
215411_s_at	10758	0.14	0.05	2.78	0.0069636
203332_s_at	3635	0.17	0.06	2.78	0.0069783
229674_at	56256	-0.21	0.07	-2.78	0.0069797
204647_at	9454	0.16	0.06	2.78	0.0069804
213318_s_at	7917	0.14	0.05	2.78	0.0069807
222275_at	10884	-0.23	0.08	-2.78	0.0069846
224626_at	113829	0.15	0.05	2.78	0.0070272
233595_at	9736	-0.26	0.09	-2.78	0.0070376
202788_at	7867	0.13	0.05	2.78	0.0070433
243305_at	57542	-0.17	0.06	-2.78	0.0070499
221567_at	8996	0.20	0.07	2.78	0.007055
209509_s_at	1798	0.16	0.06	2.78	0.007059
233559_s_at	57590	-0.18	0.06	-2.78	0.0070654
212443_at	23218	0.19	0.07	2.78	0.0070676
242467_at	1452	-0.38	0.14	-2.77	0.0071056
201083_s_at	9774	-0.28	0.10	-2.77	0.0071092
211966_at	1284	0.33	0.12	2.77	0.0071311
204358_s_at	23768	-0.23	0.08	-2.77	0.0071584
242794_at	55534	0.19	0.07	2.77	0.0071925

56821_at	55238	0.14	0.05	2.77	0.0071948
205352_at	5274	-0.32	0.12	-2.77	0.007196
223113_at	51524	0.19	0.07	2.77	0.0072436
221490_at	51271	0.12	0.04	2.77	0.0072481
202677_at	5921	0.11	0.04	2.77	0.0072486
208835_s_at	51747	-0.21	0.08	-2.77	0.0072502
225848_at	155061	0.14	0.05	2.76	0.0072842
207057_at	9194	-0.46	0.16	-2.76	0.0073403
212771_at	221061	0.15	0.05	2.76	0.0073953
243050_at	5376	-0.19	0.07	-2.76	0.007399
209352_s_at	23309	0.21	0.08	2.76	0.0074134
224807_at	57655	0.17	0.06	2.76	0.0074184
228476_at	57577	0.17	0.06	2.76	0.0074404
222439_s_at	9967	-0.23	0.08	-2.76	0.0074445
238563_at	10006	-0.30	0.11	-2.76	0.0074505
241457_at	23194	-0.34	0.12	-2.76	0.0074639
212886_at	26112	0.24	0.09	2.75	0.0074792
200984_s_at	966	-0.20	0.07	-2.75	0.0075091
212159_x_at	161	0.14	0.05	2.75	0.0075258
45297_at	30846	0.24	0.09	2.75	0.007594
220088_at	728	0.35	0.13	2.75	0.0076203
226171_at	57245	0.19	0.07	2.75	0.0076511
232510_s_at	10072	0.16	0.06	2.75	0.0076622
230332_at	84186	-0.38	0.14	-2.74	0.00773
220973_s_at	81858	0.11	0.04	2.74	0.0077446
213686_at	NA	0.14	0.05	2.74	0.0077545
224233_s_at	55154	0.26	0.10	2.74	0.0077551
229167_at	5813	-0.19	0.07	-2.74	0.0077688
222620_s_at	64215	-0.15	0.05	-2.74	0.0077719
226722_at	56975	0.18	0.07	2.74	0.0077723
218840_s_at	55191	0.12	0.04	2.74	0.0077899
234710_s_at	56965	0.12	0.04	2.74	0.0078457
226820_at	149076	0.13	0.05	2.74	0.0078623
232568_at	158295	-0.38	0.14	-2.74	0.0078775
224611_s_at	80331	0.14	0.05	2.73	0.007898
212778_at	23241	0.20	0.07	2.73	0.0079086
202893_at	10497	0.12	0.04	2.73	0.0079287
209668_x_at	8824	0.15	0.06	2.73	0.0079299
202746_at	9452	-0.37	0.14	-2.73	0.0079374
206431_x_at	23061	0.16	0.06	2.73	0.0079432
210547_x_at	3382	0.14	0.05	2.73	0.0079458
221571_at	7187	0.18	0.07	2.73	0.0079574
225294_s_at	58485	0.17	0.06	2.73	0.0079608
219765_at	79673	0.16	0.06	2.73	0.0079733
214086_s_at	10038	0.15	0.06	2.73	0.0080068
224998_at	146223	0.20	0.07	2.73	0.0080129
221752_at	54434	0.17	0.06	2.73	0.0080152
222278_at	NA	0.36	0.13	2.73	0.0080221
209270_at	3914	0.32	0.12	2.73	0.0080515
226365_at	NA	-0.27	0.10	-2.73	0.0080583
217912_at	64118	0.16	0.06	2.73	0.008082
201556_s_at	6844	0.23	0.08	2.72	0.0081274

208983_s_at	5175	-0.19	0.07	-2.72	0.0081351
222834_s_at	55970	-0.40	0.15	-2.72	0.0081355
201916_s_at	11231	0.13	0.05	2.72	0.008148
226805_at	128486	0.27	0.10	2.72	0.0081585
212832_s_at	9793	0.10	0.04	2.72	0.0081715
205580_s_at	3269	-0.20	0.07	-2.72	0.0081857
218975_at	50509	0.30	0.11	2.72	0.0081969
213730_x_at	6929	0.11	0.04	2.72	0.0082059
224186_s_at	63891	0.20	0.07	2.72	0.008206
209275_s_at	1201	0.14	0.05	2.72	0.0082404
235493_at	27332	-0.26	0.10	-2.72	0.0082505
217903_at	29888	0.23	0.08	2.72	0.0082688
225636_at	6773	0.15	0.06	2.72	0.008279
40225_at	2580	0.13	0.05	2.72	0.008282
202813_at	6894	0.16	0.06	2.72	0.0082903
223462_at	84286	0.12	0.05	2.72	0.0082931
209167_at	2824	-0.34	0.12	-2.72	0.0082961
200784_s_at	4035	0.24	0.09	2.72	0.0083215
224681_at	2768	0.14	0.05	2.72	0.0083381
209289_at	4781	-0.14	0.05	-2.71	0.0083452
206283_s_at	6886	0.17	0.06	2.71	0.0083477
218380_at	22861	0.17	0.06	2.71	0.0083686
222877_at	8828	0.41	0.15	2.71	0.0083878
223135_s_at	56987	-0.15	0.06	-2.71	0.0083969
235079_at	NA	-0.27	0.10	-2.71	0.0084002
203005_at	4055	0.16	0.06	2.71	0.0084099
239393_at	255967	-0.26	0.09	-2.71	0.0084129
209467_s_at	8569	0.16	0.06	2.71	0.0084159
220367_s_at	79595	0.16	0.06	2.71	0.0084477
226667_x_at	29924	0.13	0.05	2.71	0.0084498
242448_at	8491	-0.18	0.07	-2.71	0.0084713
220326_s_at	55701	0.25	0.09	2.71	0.0084781
207722_s_at	55643	0.17	0.06	2.71	0.0084903
213435_at	23314	0.15	0.05	2.71	0.008515
202104_s_at	6687	0.21	0.08	2.71	0.0085167
208672_s_at	6428	-0.15	0.06	-2.71	0.0085236
202861_at	5187	0.39	0.14	2.71	0.0085512
227384_s_at	57234	-0.31	0.12	-2.71	0.0085522
213861_s_at	25895	0.16	0.06	2.70	0.0085751
201056_at	2804	0.12	0.04	2.70	0.0085775
221188_s_at	27141	0.19	0.07	2.70	0.0085825
203416_at	963	0.29	0.11	2.70	0.0085837
218417_s_at	55652	0.15	0.05	2.70	0.0085856
222584_at	55154	0.22	0.08	2.70	0.0085914
223332_x_at	55658	0.15	0.06	2.70	0.008592
228535_at	5810	0.13	0.05	2.70	0.0085945
243589_at	284058	-0.32	0.12	-2.70	0.008607
203737_s_at	23082	0.20	0.08	2.70	0.0086199
204387_x_at	78988	-0.13	0.05	-2.70	0.0086235
210208_x_at	7917	0.14	0.05	2.70	0.0086405
223130_s_at	29116	-0.17	0.06	-2.70	0.0086562
204863_s_at	3572	-0.35	0.13	-2.70	0.0086661

209558_s_at	9026	0.21	0.08	2.70	0.008675
212333_at	25940	0.17	0.06	2.70	0.0086886
206019_at	9904	0.19	0.07	2.70	0.0086939
214220_s_at	7840	0.14	0.05	2.70	0.0086944
228071_at	168537	-0.25	0.09	-2.70	0.0086958
201587_s_at	3654	0.16	0.06	2.70	0.008708
218781_at	79677	0.13	0.05	2.70	0.008713
202401_s_at	6722	0.12	0.04	2.70	0.0087246
222538_s_at	26060	-0.22	0.08	-2.70	0.0087275
225800_at	221895	0.20	0.08	2.70	0.0087469
218842_at	79657	0.20	0.08	2.70	0.0087526
212642_s_at	3097	-0.14	0.05	-2.70	0.0087539
224830_at	11051	-0.23	0.08	-2.70	0.0087556
232878_at	NA	-0.28	0.10	-2.70	0.0087739
209226_s_at	3842	-0.29	0.11	-2.70	0.0087849
233365_at	5592	-0.37	0.14	-2.70	0.0088021
208997_s_at	7351	0.35	0.13	2.69	0.0088084
205642_at	11064	0.16	0.06	2.69	0.0088311
214728_x_at	6597	0.16	0.06	2.69	0.0088358
207238_s_at	5788	0.34	0.13	2.69	0.0088518
212137_at	23367	0.16	0.06	2.69	0.0088683
227106_at	440104	0.18	0.07	2.69	0.0088706
221489_s_at	81848	0.22	0.08	2.69	0.0088711
210681_s_at	9958	0.14	0.05	2.69	0.0088719
202775_s_at	6433	0.18	0.07	2.69	0.0088719
223166_x_at	55684	0.14	0.05	2.69	0.0089031
202391_at	10409	0.30	0.11	2.69	0.0089085
201918_at	55186	-0.24	0.09	-2.69	0.0089189
203402_at	8514	0.26	0.10	2.69	0.0089291
227280_s_at	151195	0.15	0.05	2.69	0.0089471
202080_s_at	22906	0.13	0.05	2.69	0.0089892
235138_at	23369	-0.50	0.19	-2.69	0.0089999
224735_at	220002	0.21	0.08	2.69	0.0090252
219825_at	56603	0.38	0.14	2.68	0.009054
222749_at	51684	0.11	0.04	2.68	0.009056
209336_at	5822	0.13	0.05	2.68	0.0090579
204363_at	2152	0.25	0.09	2.68	0.0090713
200981_x_at	2778	0.10	0.04	2.68	0.0090844
211342_x_at	9968	0.19	0.07	2.68	0.0090894
235109_at	84327	0.27	0.10	2.68	0.0091116
202679_at	4864	0.15	0.05	2.68	0.0091121
90610_at	4034	0.15	0.06	2.68	0.0091122
213671_s_at	4141	0.15	0.05	2.68	0.0091208
201085_s_at	6651	-0.24	0.09	-2.68	0.0091249
200780_x_at	2778	0.11	0.04	2.68	0.0091617
213852_at	9939	0.14	0.05	2.68	0.0091653
40569_at	7593	0.15	0.06	2.68	0.009172
201544_x_at	8106	0.13	0.05	2.68	0.0091802
213531_s_at	22930	-0.10	0.04	-2.68	0.0091976
223474_at	64207	0.18	0.07	2.68	0.0092245
204306_s_at	977	0.22	0.08	2.68	0.0092367
200616_s_at	9761	-0.25	0.09	-2.68	0.0092547

223189_x_at	55904	-0.18	0.07	-2.68	0.0092731
208712_at	595	0.36	0.13	2.68	0.0092924
219095_at	8681	0.17	0.06	2.67	0.0093075
226605_at	1609	0.17	0.06	2.67	0.0093142
244826_at	23760	-0.31	0.12	-2.67	0.0093412
226663_at	55608	-0.24	0.09	-2.67	0.009356
217788_s_at	2590	0.14	0.05	2.67	0.0093683
228454_at	84458	-0.38	0.14	-2.67	0.0093971
211950_at	23352	0.14	0.05	2.67	0.0094051
225824_at	8812	0.16	0.06	2.67	0.0094238
213603_s_at	5880	0.25	0.09	2.67	0.0094602
208622_s_at	7430	0.22	0.08	2.67	0.0094668
219243_at	55303	-0.19	0.07	-2.67	0.0094881
54970_at	83637	0.12	0.05	2.67	0.0095334
231280_at	NA	0.25	0.10	2.67	0.0095389
202801_at	5566	0.16	0.06	2.66	0.0095635
221905_at	1540	-0.28	0.11	-2.66	0.0095646
227594_at	9204	0.18	0.07	2.66	0.0095924
218058_at	30827	0.16	0.06	2.66	0.0095933
218419_s_at	79089	0.12	0.05	2.66	0.0096136
213977_s_at	25792	0.17	0.06	2.66	0.0096202
235279_at	NA	0.19	0.07	2.66	0.009622
213080_x_at	6125	-0.12	0.05	-2.66	0.0096794
209025_s_at	10492	0.16	0.06	2.66	0.0097061
240146_at	830	-0.40	0.15	-2.66	0.0097306
221741_s_at	54915	0.09	0.03	2.66	0.0097404
242972_at	NA	-0.20	0.08	-2.66	0.0098046
214617_at	5551	0.21	0.08	2.65	0.0098208
214047_s_at	8930	0.12	0.04	2.65	0.0098228
209427_at	6525	0.21	0.08	2.65	0.0098379
215092_s_at	10725	-0.20	0.08	-2.65	0.0098394
213227_at	10424	0.16	0.06	2.65	0.0098402
225925_s_at	84196	0.13	0.05	2.65	0.0098596
223139_s_at	170506	-0.19	0.07	-2.65	0.009877
227627_at	23678	0.12	0.04	2.65	0.0098942
212207_at	23389	-0.17	0.06	-2.65	0.0099045
209265_s_at	56339	0.13	0.05	2.65	0.009957
212882_at	23276	0.13	0.05	2.65	0.0099617
226679_at	284129	0.24	0.09	2.65	0.0099648
214548_x_at	2778	0.11	0.04	2.65	0.0099654
244185_at	10988	-0.25	0.10	-2.65	0.0100087
224797_at	57561	-0.33	0.12	-2.65	0.0100282
212048_s_at	8565	0.10	0.04	2.65	0.0100484
202118_s_at	8895	-0.39	0.15	-2.65	0.0100519
218150_at	26225	-0.22	0.08	-2.65	0.0100636
213033_s_at	4781	-0.16	0.06	-2.64	0.0101096
229694_at	55717	-0.31	0.12	-2.64	0.0101192
226759_at	64375	0.13	0.05	2.64	0.0101264
214696_at	84981	0.20	0.08	2.64	0.0101332
38671_at	23129	0.16	0.06	2.64	0.010135
210473_s_at	166647	0.16	0.06	2.64	0.01014
200955_at	10989	0.13	0.05	2.64	0.010141

213415_at	1193	-0.24	0.09	-2.64	0.0101452
222522_x_at	55173	-0.17	0.06	-2.64	0.0101482
206571_s_at	9448	0.13	0.05	2.64	0.0101532
209193_at	5292	0.15	0.06	2.64	0.0101756
203794_at	8476	0.09	0.03	2.64	0.0101868
241938_at	9444	-0.22	0.08	-2.64	0.0102022
208018_s_at	3055	0.29	0.11	2.64	0.0102062
219192_at	55833	0.15	0.06	2.64	0.0102092
225558_at	9815	0.11	0.04	2.64	0.0102634
227101_at	168850	-0.17	0.06	-2.64	0.0102977
243933_at	4781	-0.28	0.11	-2.64	0.0103002
240314_at	9611	-0.23	0.09	-2.64	0.0103203
233380_s_at	80230	0.12	0.05	2.63	0.0103683
217892_s_at	51474	0.13	0.05	2.63	0.0103719
212262_at	9444	0.14	0.05	2.63	0.010398
224978_s_at	57602	0.16	0.06	2.63	0.0103985
218546_at	79762	0.15	0.06	2.63	0.0104029
52255_s_at	50509	0.29	0.11	2.63	0.0104214
212235_at	23129	0.15	0.06	2.63	0.0104272
238751_at	8470	-0.46	0.17	-2.63	0.0104451
204671_s_at	22881	0.17	0.06	2.63	0.0104466
218984_at	54517	0.15	0.06	2.63	0.0104495
210417_s_at	5298	0.16	0.06	2.63	0.0104673
216331_at	3679	0.22	0.08	2.63	0.0104733
201012_at	301	0.14	0.05	2.63	0.0104832
242558_at	1499	-0.29	0.11	-2.63	0.0104943
219360_s_at	54795	0.23	0.09	2.63	0.0104982
212056_at	23199	0.20	0.08	2.63	0.0105032
228434_at	153579	0.24	0.09	2.63	0.0105616
232584_at	128553	-0.22	0.08	-2.63	0.0105841
229344_x_at	57494	0.15	0.06	2.63	0.0105903
219237_s_at	79982	-0.22	0.09	-2.63	0.0106017
57588_at	57419	0.35	0.13	2.63	0.0106219
218301_at	57140	0.15	0.06	2.62	0.0106498
225929_s_at	57674	0.16	0.06	2.62	0.010668
222406_s_at	55629	-0.25	0.09	-2.62	0.0107105
210314_x_at	407977	0.17	0.06	2.62	0.0107124
243469_at	1998	-0.22	0.08	-2.62	0.0107651
55872_at	57473	0.20	0.08	2.62	0.010787
221495_s_at	22980	0.10	0.04	2.62	0.0108047
212582_at	114882	-0.29	0.11	-2.62	0.0108235
205273_s_at	10531	0.13	0.05	2.62	0.0108298
202971_s_at	8445	-0.37	0.14	-2.62	0.0108312
204046_at	5330	0.21	0.08	2.62	0.0108401
212369_at	171017	0.12	0.05	2.62	0.0108443
232946_s_at	55191	0.14	0.05	2.62	0.0108575
219747_at	79625	-0.33	0.12	-2.62	0.0108716
225703_at	57666	0.20	0.07	2.62	0.0108833
215714_s_at	6597	0.20	0.08	2.62	0.0109014
218079_s_at	79893	0.12	0.04	2.62	0.0109033
237239_at	1387	-0.18	0.07	-2.61	0.0109299
225957_at	153222	-0.32	0.12	-2.61	0.0109314

226215_s_at	84678	0.14	0.06	2.61	0.0109492
233364_s_at	399959	-0.30	0.12	-2.61	0.0109599
232113_at	399959	-0.32	0.12	-2.61	0.0109973
209268_at	11311	0.12	0.05	2.61	0.0110265
219862_s_at	26502	0.12	0.05	2.61	0.0110412
202170_s_at	60496	-0.21	0.08	-2.61	0.0110644
214305_s_at	23451	-0.21	0.08	-2.61	0.0110932
224518_s_at	84527	0.20	0.08	2.61	0.0110972
212386_at	6925	-0.14	0.06	-2.61	0.0110991
230387_at	27032	-0.31	0.12	-2.61	0.0111043
221554_at	92335	0.11	0.04	2.61	0.011123
213958_at	923	0.13	0.05	2.61	0.0111339
225505_s_at	64773	0.18	0.07	2.61	0.0111359
217742_s_at	51322	0.10	0.04	2.61	0.0111628
231806_s_at	27148	0.15	0.06	2.61	0.0111861
204147_s_at	7027	0.11	0.04	2.61	0.0111926
240869_at	NA	0.29	0.11	2.61	0.0111964
207765_s_at	80256	0.13	0.05	2.61	0.011202
233422_at	253738	-0.19	0.07	-2.61	0.0112095
202059_s_at	3836	0.19	0.07	2.60	0.0112515
230872_s_at	26140	0.16	0.06	2.60	0.0112529
204336_s_at	10287	0.16	0.06	2.60	0.0112687
226731_at	53918	0.15	0.06	2.60	0.0112851
213664_at	6505	0.25	0.10	2.60	0.0113053
238756_at	283431	0.26	0.10	2.60	0.0113394
224780_at	84991	0.10	0.04	2.60	0.0113547
201475_x_at	4141	0.14	0.06	2.60	0.0113598
215743_at	9397	0.28	0.11	2.60	0.0113654
204575_s_at	4327	0.29	0.11	2.60	0.0114231
230972_at	122416	0.21	0.08	2.60	0.0114317
202068_s_at	3949	0.39	0.15	2.60	0.0114449
225878_at	23095	-0.15	0.06	-2.60	0.0114725
236679_x_at	1818	-0.21	0.08	-2.60	0.0114773
230391_at	NA	0.32	0.12	2.60	0.0114963
220975_s_at	114897	0.22	0.09	2.60	0.0115074
201320_at	6601	0.16	0.06	2.59	0.0115172
205089_at	7553	0.11	0.04	2.59	0.0115487
218652_s_at	54872	0.12	0.04	2.59	0.011555
229261_at	6654	-0.38	0.14	-2.59	0.011561
224756_s_at	7920	0.13	0.05	2.59	0.0115925
235056_at	2120	0.27	0.10	2.59	0.0116018
201683_x_at	9878	-0.14	0.05	-2.59	0.0116564
215994_x_at	23061	0.15	0.06	2.59	0.0116771
227740_at	127933	-0.36	0.14	-2.59	0.0116895
201605_x_at	1265	0.14	0.05	2.59	0.0116967
201769_at	9685	0.09	0.03	2.59	0.0117118
203530_s_at	6810	0.16	0.06	2.59	0.011784
206081_at	9187	0.17	0.06	2.59	0.0117858
203318_s_at	7707	-0.16	0.06	-2.58	0.01184
201247_at	6721	0.13	0.05	2.58	0.011866
224339_s_at	9068	-0.26	0.10	-2.58	0.0118845
231713_s_at	55250	-0.11	0.04	-2.58	0.0118966

204703_at	8100	0.12	0.04	2.58	0.0119364
219396_s_at	79661	0.17	0.06	2.58	0.0119489
205225_at	2099	-0.30	0.12	-2.58	0.0119524
228257_at	283373	0.15	0.06	2.58	0.0119712
211518_s_at	652	-0.15	0.06	-2.58	0.0120056
215385_at	79068	-0.24	0.09	-2.58	0.0120131
236561_at	7046	-0.32	0.12	-2.58	0.0120188
208634_s_at	23499	0.10	0.04	2.58	0.0120262
228488_at	125058	0.26	0.10	2.58	0.0120342
236184_at	10350	-0.24	0.09	-2.58	0.0120686
224773_at	89796	0.22	0.08	2.58	0.0120785
226116_at	NA	0.13	0.05	2.58	0.0121068
201721_s_at	7805	0.28	0.11	2.58	0.0121126
204135_at	11259	0.21	0.08	2.58	0.0121213
224971_at	51263	0.14	0.05	2.58	0.0121316
224836_at	58476	0.18	0.07	2.57	0.0121492
202898_at	9672	0.27	0.11	2.57	0.0122066
218763_at	53407	0.08	0.03	2.57	0.0122213
241395_at	9191	0.16	0.06	2.57	0.0122287
220761_s_at	51347	0.16	0.06	2.57	0.0122309
230063_at	9422	0.14	0.05	2.57	0.0122358
212031_at	58517	0.12	0.05	2.57	0.0122373
213644_at	201134	0.16	0.06	2.57	0.0122402
224927_at	170954	0.16	0.06	2.57	0.0122788
226085_at	23468	-0.31	0.12	-2.57	0.012292
203801_at	63931	0.19	0.07	2.57	0.0122963
203054_s_at	6988	0.16	0.06	2.57	0.0123065
224658_x_at	55690	0.15	0.06	2.57	0.0123319
211121_s_at	1796	0.18	0.07	2.57	0.0123787
227409_at	57594	0.15	0.06	2.57	0.012386
219251_s_at	55112	0.12	0.05	2.57	0.0123865
214494_s_at	6687	0.17	0.06	2.57	0.012404
203064_s_at	3607	0.13	0.05	2.57	0.0124147
223082_at	30011	0.17	0.07	2.57	0.0124454
222526_at	54815	0.15	0.06	2.57	0.0124606
34406_at	23241	0.16	0.06	2.56	0.0124756
223145_s_at	55122	0.20	0.08	2.56	0.0125486
228311_at	255877	-0.16	0.06	-2.56	0.0125565
227282_at	57526	-0.22	0.09	-2.56	0.0125782
228841_at	90624	0.12	0.05	2.56	0.0126017
203203_s_at	11103	-0.23	0.09	-2.56	0.012612
203859_s_at	5064	0.15	0.06	2.56	0.0126362
209361_s_at	57060	0.18	0.07	2.56	0.0126512
204258_at	1105	-0.14	0.05	-2.56	0.0126632
209090_s_at	51100	0.12	0.05	2.56	0.0126774
201294_s_at	26118	-0.32	0.12	-2.56	0.0126779
201289_at	3491	0.41	0.16	2.56	0.012697
221711_s_at	29086	0.11	0.04	2.56	0.0127075
215706_x_at	7791	0.21	0.08	2.56	0.0127346
209902_at	545	0.20	0.08	2.56	0.0127612
229831_at	5067	0.32	0.13	2.56	0.0127933
208072_s_at	8527	0.12	0.05	2.56	0.0127942

223184_s_at	56894	0.20	0.08	2.55	0.0128
226657_at	256306	0.27	0.11	2.55	0.0128074
218455_at	9054	0.10	0.04	2.55	0.0128373
201221_s_at	6625	0.18	0.07	2.55	0.012851
228539_at	NA	0.20	0.08	2.55	0.0128524
229083_at	10949	-0.13	0.05	-2.55	0.0128661
222759_at	51111	0.12	0.05	2.55	0.0128873
226363_at	10057	0.20	0.08	2.55	0.0129017
223261_at	51426	-0.20	0.08	-2.55	0.0129017
212147_at	23381	0.10	0.04	2.55	0.0129097
229862_x_at	84878	0.13	0.05	2.55	0.0129103
223303_at	83706	0.26	0.10	2.55	0.0129195
204719_at	10351	-0.19	0.07	-2.55	0.0129407
AFFX-HUMRGE/M10098_5_at	#N/A	-0.33	0.13	-2.55	0.0129488
201697_s_at	1786	0.17	0.07	2.55	0.0129573
209083_at	11151	0.29	0.12	2.55	0.012959
224728_at	64756	0.13	0.05	2.55	0.0129617
236685_at	NA	-0.25	0.10	-2.55	0.0129673
212005_at	26099	0.18	0.07	2.55	0.0129722
206342_x_at	3423	0.15	0.06	2.55	0.0129724
228315_at	NA	-0.33	0.13	-2.55	0.0129795
226416_at	90459	0.15	0.06	2.55	0.0129932
213575_at	29896	-0.23	0.09	-2.55	0.0130003
218917_s_at	8289	0.14	0.05	2.55	0.0130058
217891_at	64755	0.19	0.07	2.55	0.0130892
204663_at	10873	0.18	0.07	2.55	0.0130986
215792_s_at	55735	0.13	0.05	2.54	0.0131414
201190_s_at	5306	0.08	0.03	2.54	0.0131621
213566_at	6039	0.34	0.14	2.54	0.0131752
225530_at	126308	0.18	0.07	2.54	0.0131896
215392_at	9960	-0.23	0.09	-2.54	0.0132014
222679_s_at	54165	-0.23	0.09	-2.54	0.0132144
212576_at	23295	0.15	0.06	2.54	0.0132211
227396_at	5795	0.21	0.08	2.54	0.0132409
201843_s_at	2202	0.20	0.08	2.54	0.0132528
218930_s_at	54664	-0.27	0.11	-2.54	0.0132611
1294_at	7318	0.18	0.07	2.54	0.0132682
224247_s_at	55173	-0.20	0.08	-2.54	0.0132737
207801_s_at	9921	0.13	0.05	2.54	0.0132786
201911_s_at	10160	0.16	0.06	2.54	0.013307
201454_s_at	9520	-0.14	0.05	-2.54	0.0133109
201771_at	10067	0.11	0.04	2.54	0.0133313
221870_at	30846	0.21	0.08	2.54	0.0133613
227286_at	283899	0.16	0.06	2.54	0.0133778
225320_at	90550	0.09	0.03	2.54	0.0133801
200746_s_at	2782	0.09	0.03	2.54	0.0134166
218909_at	26750	0.13	0.05	2.54	0.0134198
209663_s_at	3679	0.26	0.10	2.54	0.0134388
210480_s_at	4646	0.15	0.06	2.54	0.0134512
212937_s_at	1291	0.25	0.10	2.54	0.0134619
202128_at	9870	0.12	0.05	2.54	0.0134636
200954_at	527	0.15	0.06	2.54	0.0134759

211000_s_at	3572	-0.37	0.14	-2.54	0.0134799
211716_x_at	396	0.17	0.07	2.53	0.0135079
202962_at	23303	0.13	0.05	2.53	0.0135647
223598_at	5887	0.19	0.08	2.53	0.0135967
209088_s_at	29855	-0.16	0.06	-2.53	0.0136048
203572_s_at	6878	0.15	0.06	2.53	0.0136494
218523_at	64077	0.17	0.07	2.53	0.013656
229449_at	8459	0.23	0.09	2.53	0.0136565
201656_at	3655	-0.21	0.08	-2.53	0.0136609
204116_at	3561	0.28	0.11	2.53	0.013669
1729_at	8717	0.07	0.03	2.53	0.0136953
203459_s_at	64601	0.09	0.04	2.53	0.0137065
203185_at	9770	0.24	0.10	2.53	0.0137352
37966_at	29780	0.18	0.07	2.53	0.0137488
202776_at	30836	0.12	0.05	2.53	0.013762
204206_at	4335	0.16	0.06	2.53	0.0137783
202643_s_at	7128	0.16	0.07	2.53	0.0138297
207163_s_at	207	0.16	0.06	2.52	0.0138535
222065_s_at	2314	0.13	0.05	2.52	0.0138849
209023_s_at	10735	-0.24	0.10	-2.52	0.0138858
227510_x_at	29005	-0.32	0.13	-2.52	0.0138859
226497_s_at	2321	-0.20	0.08	-2.52	0.013894
204638_at	54	0.43	0.17	2.52	0.0139715
236313_at	1030	0.33	0.13	2.52	0.0140007
230892_at	51071	-0.26	0.10	-2.52	0.0140139
212417_at	9522	-0.28	0.11	-2.52	0.0140451
228180_at	55234	-0.23	0.09	-2.52	0.0140498
212542_s_at	55023	-0.22	0.09	-2.52	0.0140597
230779_at	23112	-0.28	0.11	-2.52	0.0140667
204294_at	275	0.20	0.08	2.52	0.0140739
231735_s_at	29005	-0.19	0.08	-2.52	0.0141448
209042_s_at	7327	0.10	0.04	2.52	0.0141579
227117_at	11260	0.17	0.07	2.51	0.0142219
228980_at	117584	0.16	0.06	2.51	0.0142443
201865_x_at	2908	-0.22	0.09	-2.51	0.0142584
207320_x_at	6780	0.07	0.03	2.51	0.0142861
214919_s_at	8637	0.11	0.04	2.51	0.0142899
213567_at	NA	0.12	0.05	2.51	0.014312
213229_at	23405	-0.24	0.10	-2.51	0.0143198
AFFX-HSAC07/X00351_3_at	60	0.08	0.03	2.51	0.0143259
223347_at	84939	0.14	0.06	2.51	0.0143264
211981_at	1282	0.29	0.11	2.51	0.0143339
218387_s_at	25796	0.13	0.05	2.51	0.0143371
221704_s_at	79720	0.14	0.06	2.51	0.0143819
225871_at	261729	0.21	0.08	2.51	0.0143877
222628_s_at	51455	-0.11	0.04	-2.51	0.0144253
213628_at	23155	0.10	0.04	2.51	0.0144815
213901_x_at	23543	-0.18	0.07	-2.51	0.0144886
202060_at	9646	0.12	0.05	2.51	0.0145213
215464_s_at	30851	0.14	0.06	2.51	0.0145282
201412_at	26020	0.12	0.05	2.51	0.0145305
203853_s_at	9846	0.17	0.07	2.51	0.0145372

211582_x_at	7940	0.21	0.08	2.50	0.0145764
204076_at	9583	0.15	0.06	2.50	0.0145783
213018_at	57798	0.12	0.05	2.50	0.0146214
207417_s_at	7730	0.17	0.07	2.50	0.0146274
207542_s_at	358	-0.23	0.09	-2.50	0.014716
235129_at	5502	0.34	0.14	2.50	0.0147267
212081_x_at	7916	0.17	0.07	2.50	0.014789
225110_at	55239	0.12	0.05	2.50	0.014792
232168_x_at	23499	-0.17	0.07	-2.50	0.0148425
203202_at	11103	-0.13	0.05	-2.50	0.0148755
235061_at	152926	-0.27	0.11	-2.50	0.0148792
243184_at	7082	-0.23	0.09	-2.50	0.0148864
223343_at	58475	0.34	0.14	2.50	0.0149145
224733_at	123920	0.13	0.05	2.50	0.0149449
218386_x_at	10600	0.10	0.04	2.49	0.0149834
230712_at	55672	-0.23	0.09	-2.49	0.0149958
213980_s_at	1487	0.16	0.07	2.49	0.0150274
201341_at	8507	0.21	0.08	2.49	0.0150457
205308_at	51101	-0.21	0.08	-2.49	0.0150858
222802_at	1906	0.32	0.13	2.49	0.0151118
203550_s_at	10712	0.10	0.04	2.49	0.0151134
202646_s_at	7812	0.12	0.05	2.49	0.0151172
232406_at	182	-0.23	0.09	-2.49	0.0151188
202317_s_at	10277	0.09	0.04	2.49	0.0151413
202908_at	7466	0.19	0.08	2.49	0.0151557
205640_at	221	0.13	0.05	2.49	0.0151575
242403_at	7323	-0.23	0.09	-2.49	0.0151646
212201_at	23141	0.11	0.04	2.49	0.0151747
209350_s_at	2874	0.10	0.04	2.49	0.0152069
220072_at	79848	-0.18	0.07	-2.49	0.0152195
212634_at	23376	-0.18	0.07	-2.49	0.0152289
222706_at	54883	0.14	0.05	2.49	0.0152745
215248_at	2887	-0.29	0.11	-2.49	0.01531
221769_at	90864	0.12	0.05	2.49	0.0153142
230435_at	375190	0.12	0.05	2.48	0.0153581
221519_at	6468	0.13	0.05	2.48	0.0153668
213675_at	NA	0.15	0.06	2.48	0.015381
203319_s_at	7707	-0.18	0.07	-2.48	0.0153884
204431_at	7089	0.17	0.07	2.48	0.0153988
239250_at	147947	0.15	0.06	2.48	0.0154175
213117_at	55958	-0.15	0.06	-2.48	0.015451
200636_s_at	5792	0.31	0.13	2.48	0.0154543
228347_at	6495	0.47	0.19	2.48	0.0154849
212091_s_at	1291	0.22	0.09	2.48	0.0154894
233607_at	NA	-0.30	0.12	-2.48	0.0154923
205269_at	3937	0.25	0.10	2.48	0.0155265
222133_s_at	51105	-0.20	0.08	-2.48	0.0155282
221656_s_at	55160	0.14	0.06	2.48	0.0155779
224953_at	81555	-0.29	0.11	-2.48	0.0155785
229121_at	NA	0.21	0.09	2.48	0.0156051
202173_s_at	7716	-0.13	0.05	-2.48	0.0156085
212064_x_at	4150	0.14	0.06	2.48	0.0156085

202899_s_at	6428	-0.26	0.10	-2.48	0.0156407
218306_s_at	8925	0.08	0.03	2.48	0.0156482
225763_at	92241	0.15	0.06	2.48	0.0157
226298_at	146923	0.19	0.08	2.48	0.015734
235152_at	NA	-0.16	0.07	-2.47	0.0157642
208670_s_at	23741	-0.13	0.05	-2.47	0.0157764
217731_s_at	9445	-0.09	0.04	-2.47	0.0157987
218064_s_at	26993	0.16	0.06	2.47	0.0157992
207198_s_at	3987	-0.20	0.08	-2.47	0.0158314
225701_at	80709	0.16	0.06	2.47	0.0158424
204436_at	80301	0.18	0.07	2.47	0.0158574
202152_x_at	7392	0.13	0.05	2.47	0.0158739
242680_at	NA	-0.29	0.12	-2.47	0.0158946
228411_at	117583	0.17	0.07	2.47	0.0159084
226334_s_at	130872	0.17	0.07	2.47	0.0159534
232683_s_at	56965	0.11	0.04	2.47	0.015957
200600_at	4478	0.12	0.05	2.47	0.0159945
202486_at	10939	0.10	0.04	2.47	0.0159946
225504_at	79618	0.08	0.03	2.47	0.0160204
223041_at	83692	0.14	0.06	2.47	0.0160786
228694_at	NA	-0.25	0.10	-2.47	0.0160993
209345_s_at	55361	0.15	0.06	2.47	0.0161065
234074_at	NA	-0.27	0.11	-2.47	0.0161156
210102_at	4013	0.26	0.11	2.47	0.0161309
233611_at	5592	-0.24	0.10	-2.47	0.0161314
213118_at	23074	0.14	0.06	2.47	0.0161319
212518_at	23396	0.15	0.06	2.46	0.016168
242480_at	7994	-0.17	0.07	-2.46	0.0161819
223591_at	84282	0.13	0.05	2.46	0.0161914
213527_s_at	146542	0.10	0.04	2.46	0.0162082
218545_at	55297	0.15	0.06	2.46	0.016238
213478_at	23254	0.17	0.07	2.46	0.0162549
232617_at	1520	0.24	0.10	2.46	0.0162863
215936_s_at	23325	-0.29	0.12	-2.46	0.0162967
215737_x_at	7392	0.11	0.05	2.46	0.0163008
209734_at	3071	0.22	0.09	2.46	0.016303
229574_at	29896	-0.23	0.09	-2.46	0.0163044
224566_at	283131	0.14	0.06	2.46	0.0163201
32091_at	9673	0.12	0.05	2.46	0.0163258
223741_s_at	94015	0.12	0.05	2.46	0.0163431
202151_s_at	10422	0.11	0.05	2.46	0.0163448
212151_at	5087	-0.12	0.05	-2.46	0.0163651
222369_at	79829	0.19	0.08	2.46	0.016373
202600_s_at	8204	-0.32	0.13	-2.46	0.0163988
201415_at	2937	0.10	0.04	2.46	0.0164425
238148_s_at	390963	0.22	0.09	2.46	0.0164741
209675_s_at	11100	0.21	0.08	2.46	0.0164799
200648_s_at	2752	-0.39	0.16	-2.46	0.0165731
216306_x_at	5725	0.14	0.06	2.45	0.0165805
213422_s_at	54587	0.12	0.05	2.45	0.0165841
208838_at	55832	-0.20	0.08	-2.45	0.0166321
205882_x_at	120	-0.20	0.08	-2.45	0.0166537

218958_at	55049	0.13	0.05	2.45	0.0166618
1552310_at	123207	0.13	0.05	2.45	0.0166754
209354_at	8764	0.14	0.06	2.45	0.0167035
227663_at	NA	-0.30	0.12	-2.45	0.0167181
200704_at	9516	0.17	0.07	2.45	0.0167581
209655_s_at	83604	-0.23	0.09	-2.45	0.016763
212188_at	115207	-0.28	0.11	-2.45	0.0167909
212852_s_at	6738	-0.23	0.09	-2.45	0.0168045
235293_at	11145	0.24	0.10	2.45	0.0168534
233674_at	2309	-0.16	0.07	-2.45	0.0168536
201282_at	4967	0.14	0.06	2.45	0.0168681
241769_at	3685	-0.31	0.13	-2.45	0.0168682
244153_at	7756	-0.14	0.06	-2.45	0.0168731
204505_s_at	2039	0.26	0.10	2.45	0.016875
203446_s_at	4952	0.10	0.04	2.45	0.0168772
215909_x_at	50488	0.19	0.08	2.45	0.016905
201715_s_at	22985	0.18	0.07	2.45	0.0169068
227451_s_at	NA	0.19	0.08	2.45	0.016928
212420_at	1997	-0.15	0.06	-2.45	0.016944
208661_s_at	7267	-0.20	0.08	-2.45	0.0169609
217836_s_at	55249	0.11	0.04	2.45	0.0169751
225751_at	84991	-0.11	0.05	-2.44	0.0170185
214151_s_at	9236	0.18	0.07	2.44	0.0170245
207738_s_at	10787	-0.16	0.06	-2.44	0.0170711
203139_at	1612	0.15	0.06	2.44	0.0170762
225827_at	27161	-0.19	0.08	-2.44	0.0171196
210844_x_at	1495	0.09	0.04	2.44	0.0171391
203782_s_at	5442	0.21	0.09	2.44	0.017174
221006_s_at	81609	-0.13	0.05	-2.44	0.0171757
37254_at	7692	0.10	0.04	2.44	0.0172131
220757_s_at	80700	0.13	0.06	2.44	0.0172602
213242_x_at	283638	0.16	0.07	2.44	0.0172604
208420_x_at	6830	0.15	0.06	2.44	0.0172718
213507_s_at	3837	0.11	0.05	2.44	0.0172728
201598_s_at	3636	0.12	0.05	2.44	0.0173164
213352_at	23023	0.20	0.08	2.44	0.01732
204314_s_at	1385	-0.24	0.10	-2.44	0.0173296
203599_s_at	11193	0.13	0.05	2.44	0.0174258
239151_at	255326	-0.17	0.07	-2.43	0.0174629
211725_s_at	637	0.15	0.06	2.43	0.0175093
220392_at	64641	-0.18	0.08	-2.43	0.0175233
226249_at	401548	0.16	0.07	2.43	0.0175815
239723_at	30061	-0.31	0.13	-2.43	0.017586
202140_s_at	1198	0.13	0.05	2.43	0.0176302
221566_s_at	8996	0.21	0.09	2.43	0.0176333
204882_at	9938	0.14	0.06	2.43	0.0176567
215982_s_at	1797	0.15	0.06	2.43	0.017677
223644_s_at	1427	0.12	0.05	2.43	0.0176832
221779_at	85377	0.14	0.06	2.43	0.0176899
215329_s_at	985	0.13	0.05	2.43	0.0177353
214211_at	2495	0.20	0.08	2.43	0.0177382
227208_at	338657	0.14	0.06	2.43	0.017769

226459_at	118788	0.28	0.12	2.43	0.0177852
204141_at	7280	0.30	0.13	2.43	0.0178192
203558_at	9820	0.13	0.05	2.43	0.0178264
213934_s_at	7571	0.16	0.06	2.43	0.017837
201378_s_at	9898	0.12	0.05	2.43	0.0178557
212776_s_at	23363	0.17	0.07	2.43	0.0178566
230777_s_at	63977	0.11	0.05	2.42	0.0178928
202822_at	4026	-0.16	0.07	-2.42	0.0179053
203513_at	80208	0.08	0.03	2.42	0.0179241
226519_s_at	85007	0.11	0.04	2.42	0.0179356
219902_at	23743	0.24	0.10	2.42	0.0179384
208270_s_at	6051	0.10	0.04	2.42	0.0179719
201655_s_at	3339	0.18	0.07	2.42	0.0179914
203491_s_at	9702	-0.23	0.10	-2.42	0.0180392
202715_at	790	0.15	0.06	2.42	0.0180446
217944_at	541468	0.14	0.06	2.42	0.0180491
214792_x_at	6844	0.22	0.09	2.42	0.0180539
217904_s_at	23621	0.11	0.04	2.42	0.0180923
205247_at	4855	0.19	0.08	2.42	0.0180956
212414_s_at	84656	0.15	0.06	2.42	0.0181288
32032_at	8220	0.11	0.05	2.42	0.0181361
204925_at	1497	0.17	0.07	2.42	0.0181379
209375_at	7508	0.12	0.05	2.42	0.0182013
217983_s_at	8635	0.20	0.08	2.42	0.0182329
205428_s_at	794	0.33	0.13	2.42	0.0182483
234991_at	79364	-0.20	0.08	-2.42	0.0182508
223201_s_at	84187	0.21	0.09	2.42	0.018253
209381_x_at	8175	0.19	0.08	2.42	0.0182581
202687_s_at	8743	-0.17	0.07	-2.42	0.0182582
202253_s_at	1785	0.14	0.06	2.42	0.0182805
234491_s_at	60485	-0.18	0.07	-2.42	0.0183469
204661_at	1043	0.43	0.18	2.41	0.0183695
227379_at	154141	0.17	0.07	2.41	0.0183804
202251_at	9129	0.12	0.05	2.41	0.0184092
230350_at	NA	0.11	0.05	2.41	0.0184283
203471_s_at	5341	0.27	0.11	2.41	0.0184344
203580_s_at	9057	0.19	0.08	2.41	0.0184525
221986_s_at	54800	-0.27	0.11	-2.41	0.0184666
202368_s_at	9697	-0.26	0.11	-2.41	0.0185013
207173_x_at	1009	0.20	0.08	2.41	0.0185345
202984_s_at	9529	-0.26	0.11	-2.41	0.0185452
230426_at	1738	-0.23	0.09	-2.41	0.0185526
239484_at	54822	0.16	0.07	2.41	0.0185733
239171_at	120	-0.24	0.10	-2.41	0.0186259
204022_at	11060	0.11	0.05	2.41	0.0186799
AFFX-HUMRGE/M10098_M_at	2857	-0.25	0.11	-2.41	0.0187055
202161_at	5585	0.12	0.05	2.41	0.018728
213199_at	26005	0.12	0.05	2.41	0.0187524
225582_at	85450	0.12	0.05	2.41	0.0187632
227429_at	283229	0.18	0.08	2.41	0.0187662
214672_at	23093	0.12	0.05	2.41	0.0187763
209536_s_at	30844	0.14	0.06	2.41	0.0187784

208791_at	1191	0.16	0.07	2.40	0.0188222
222600_s_at	55236	-0.16	0.07	-2.40	0.0188433
225226_at	85369	0.10	0.04	2.40	0.0189052
53720_at	55337	0.17	0.07	2.40	0.0189705
223333_s_at	51129	0.35	0.15	2.40	0.0189707
209257_s_at	9126	-0.31	0.13	-2.40	0.0189813
215749_s_at	64689	0.11	0.05	2.40	0.0189938
218941_at	26190	0.10	0.04	2.40	0.0190009
233442_at	23024	-0.22	0.09	-2.40	0.0190237
203231_s_at	6310	-0.28	0.12	-2.40	0.0190441
217911_s_at	9531	0.19	0.08	2.40	0.0190651
222339_x_at	NA	-0.12	0.05	-2.40	0.0190798
238890_at	NA	0.15	0.06	2.40	0.0190997
227742_at	54102	0.46	0.19	2.40	0.0191034
201469_s_at	6464	0.13	0.06	2.40	0.019155
36829_at	5187	0.27	0.11	2.40	0.0191587
223500_at	10815	0.32	0.13	2.40	0.0191941
228723_at	283677	-0.23	0.10	-2.40	0.019219
201831_s_at	8615	-0.21	0.09	-2.40	0.0192233
229434_at	3184	-0.23	0.10	-2.40	0.0192547
232008_s_at	56987	-0.14	0.06	-2.40	0.01928
226853_at	55589	0.18	0.07	2.39	0.019309
225187_at	57805	0.15	0.06	2.39	0.0193351
231969_at	56977	-0.23	0.10	-2.39	0.0193393
217840_at	51428	0.10	0.04	2.39	0.0193558
201328_at	2114	-0.20	0.08	-2.39	0.0193822
212772_s_at	20	0.17	0.07	2.39	0.0194063
222453_at	79901	-0.15	0.06	-2.39	0.0194085
201007_at	3032	0.10	0.04	2.39	0.0194171
208683_at	824	0.10	0.04	2.39	0.0194319
239815_at	57727	-0.18	0.08	-2.39	0.0194425
222303_at	2114	-0.35	0.15	-2.39	0.019448
241905_at	5286	-0.24	0.10	-2.39	0.0194635
212701_at	83660	0.24	0.10	2.39	0.0194925
209685_s_at	5579	0.23	0.09	2.39	0.0195023
201255_x_at	7917	0.12	0.05	2.39	0.0195112
227564_at	138050	0.09	0.04	2.39	0.0195276
236907_at	26986	-0.20	0.08	-2.39	0.0195342
222872_x_at	64859	0.25	0.10	2.39	0.0195424
226153_s_at	246175	-0.21	0.09	-2.39	0.0195719
241391_at	NA	-0.25	0.11	-2.39	0.0195774
233323_at	143187	-0.16	0.07	-2.39	0.0196414
218379_at	10179	-0.18	0.08	-2.39	0.0196496
221500_s_at	8675	0.10	0.04	2.39	0.0196621
226625_at	7049	-0.13	0.05	-2.39	0.0196707
227558_at	8535	0.17	0.07	2.39	0.0196788
224002_s_at	51661	-0.28	0.12	-2.39	0.0196842
227576_at	NA	-0.17	0.07	-2.39	0.0197332
230987_at	NA	-0.25	0.11	-2.39	0.0197366
208890_s_at	23654	0.17	0.07	2.39	0.019763
60471_at	79890	0.16	0.07	2.39	0.0197639
223029_s_at	84231	0.10	0.04	2.38	0.0198396

208955_at	1854	0.14	0.06	2.38	0.0198434
222040_at	3178	0.15	0.06	2.38	0.0198444
222761_at	54841	0.11	0.05	2.38	0.0198505
236524_at	83941	-0.20	0.09	-2.38	0.019889
218424_s_at	55240	0.18	0.07	2.38	0.0198956
200721_s_at	10121	0.13	0.05	2.38	0.0199187
224907_s_at	56904	0.16	0.07	2.38	0.0199332
228445_at	84883	0.22	0.09	2.38	0.0199825
213184_at	205564	0.10	0.04	2.38	0.0199888
65521_at	51619	0.09	0.04	2.38	0.0200753
203683_s_at	7423	0.20	0.09	2.38	0.0200775
207390_s_at	6525	0.20	0.09	2.38	0.0200904
38340_at	9026	0.15	0.06	2.38	0.0201123
203668_at	4123	0.17	0.07	2.38	0.020148
201935_s_at	8672	0.14	0.06	2.38	0.0201604
209311_at	599	0.11	0.05	2.38	0.0201693
238712_at	NA	-0.24	0.10	-2.38	0.0201843
218328_at	51117	0.10	0.04	2.38	0.020188
202734_at	9322	0.14	0.06	2.38	0.0202323
203933_at	9727	0.11	0.05	2.38	0.0202705
222512_at	51667	0.12	0.05	2.37	0.0202936
225507_at	25957	-0.20	0.08	-2.37	0.0202961
209112_at	1027	-0.14	0.06	-2.37	0.0203213
214426_x_at	10036	0.17	0.07	2.37	0.0203471
203118_at	9159	0.12	0.05	2.37	0.0203559
229800_at	9201	-0.32	0.13	-2.37	0.0203687
225729_at	221477	0.08	0.03	2.37	0.0203763
203921_at	9435	0.21	0.09	2.37	0.0203957
222670_s_at	9935	0.14	0.06	2.37	0.0203983
202074_s_at	10133	0.13	0.05	2.37	0.0204095
221867_at	9683	0.16	0.07	2.37	0.0204301
222826_at	26258	-0.24	0.10	-2.37	0.0204335
203940_s_at	22846	0.16	0.07	2.37	0.0204739
229356_x_at	54617	0.09	0.04	2.37	0.0204831
204284_at	5507	-0.28	0.12	-2.37	0.0204909
204760_s_at	9572	0.37	0.16	2.37	0.0205153
217966_s_at	116496	-0.24	0.10	-2.37	0.0205879
215364_s_at	23334	0.14	0.06	2.37	0.0206076
213235_at	400506	-0.13	0.05	-2.37	0.0206404
201611_s_at	23463	0.13	0.05	2.37	0.0207104
213016_at	56987	-0.18	0.08	-2.37	0.0207334
202027_at	25829	0.13	0.06	2.37	0.0207534
227951_s_at	147965	0.09	0.04	2.37	0.0207851
228750_at	NA	-0.44	0.19	-2.37	0.0207948
238494_at	26146	0.12	0.05	2.37	0.0208004
212787_at	56252	0.11	0.05	2.36	0.0208251
211105_s_at	4772	0.16	0.07	2.36	0.020847
214574_x_at	7940	0.18	0.08	2.36	0.0208656
212793_at	23500	0.22	0.09	2.36	0.0210045
226012_at	29123	0.12	0.05	2.36	0.02101
224949_at	81555	-0.19	0.08	-2.36	0.0210535
227670_at	7627	0.15	0.06	2.36	0.0210612

238119_at	56947	-0.25	0.11	-2.36	0.0210673
38043_at	60343	0.12	0.05	2.36	0.0210979
202830_s_at	2542	0.09	0.04	2.36	0.0211296
243318_at	50717	-0.21	0.09	-2.36	0.0211404
232264_at	51366	-0.25	0.11	-2.36	0.0211441
226198_at	146691	0.12	0.05	2.36	0.0211614
226344_at	84460	0.17	0.07	2.36	0.0212182
65630_at	283232	0.13	0.06	2.36	0.0212214
201639_s_at	29894	0.24	0.10	2.36	0.0212979
229743_at	220929	0.09	0.04	2.35	0.0213338
229113_s_at	199990	0.15	0.06	2.35	0.0213502
224921_at	10066	0.13	0.06	2.35	0.0213593
218364_at	9209	0.10	0.04	2.35	0.021449
212260_at	26058	0.12	0.05	2.35	0.0214695
210276_s_at	11078	0.13	0.06	2.35	0.0214996
219241_x_at	54961	0.13	0.06	2.35	0.0215414
34210_at	1043	0.50	0.21	2.35	0.0215489
209378_s_at	54462	0.12	0.05	2.35	0.0215602
200759_x_at	4779	0.14	0.06	2.35	0.0215632
231777_at	1460	0.23	0.10	2.35	0.0215827
203714_s_at	6905	0.09	0.04	2.35	0.0215959
204963_at	8082	-0.17	0.07	-2.35	0.0216059
225977_at	54510	-0.16	0.07	-2.35	0.0216243
235507_at	115294	-0.17	0.07	-2.35	0.0216308
203278_s_at	51317	0.09	0.04	2.35	0.0216349
212496_s_at	23030	0.14	0.06	2.35	0.0216677
241701_at	57584	-0.22	0.09	-2.35	0.0216735
244052_at	84869	0.12	0.05	2.35	0.0217143
226369_at	338799	0.17	0.07	2.35	0.0217291
207988_s_at	10109	0.10	0.04	2.35	0.0217404
210944_s_at	825	0.16	0.07	2.35	0.0217656
215287_at	NA	-0.23	0.10	-2.35	0.0217938
202326_at	10919	0.14	0.06	2.35	0.0218428
201050_at	23646	0.20	0.09	2.35	0.0218481
223442_at	84276	0.16	0.07	2.35	0.0218523
227444_at	158947	0.17	0.07	2.35	0.0218524
218059_at	51123	0.09	0.04	2.35	0.0218632
217627_at	126231	-0.16	0.07	-2.34	0.0219134
222667_s_at	55870	-0.15	0.06	-2.34	0.0219299
203570_at	4016	0.24	0.10	2.34	0.0219493
240238_at	25771	-0.12	0.05	-2.34	0.021985
228664_at	NA	0.14	0.06	2.34	0.0219989
224846_at	92799	0.14	0.06	2.34	0.0220116
205817_at	6495	0.31	0.13	2.34	0.0220125
208979_at	23054	0.12	0.05	2.34	0.0220335
224875_at	134553	-0.25	0.11	-2.34	0.0220824
212015_x_at	5725	0.12	0.05	2.34	0.0220879
228129_at	26135	-0.24	0.10	-2.34	0.0220895
234972_at	339231	0.14	0.06	2.34	0.0221049
202911_at	2956	-0.11	0.05	-2.34	0.0221242
209283_at	1410	0.25	0.11	2.34	0.0221658
244292_at	NA	-0.20	0.08	-2.34	0.0221866

1552277_a_at	91283	0.13	0.06	2.34	0.0222905
230722_at	54796	-0.29	0.12	-2.34	0.0223366
213069_at	57493	-0.17	0.07	-2.34	0.022357
229366_at	51185	0.15	0.07	2.34	0.0223682
221009_s_at	51129	0.33	0.14	2.34	0.0223752
228306_at	29097	0.13	0.05	2.34	0.0223761
218617_at	54802	0.10	0.04	2.34	0.0223844
58994_at	54862	0.18	0.08	2.34	0.0223895
201545_s_at	8106	0.14	0.06	2.34	0.0224056
208927_at	8405	-0.11	0.05	-2.34	0.0224094
208674_x_at	1650	-0.14	0.06	-2.33	0.0224519
218231_at	55577	0.12	0.05	2.33	0.0224788
219007_at	348995	0.12	0.05	2.33	0.0225076
225058_at	56927	0.11	0.05	2.33	0.0225127
209711_at	23169	-0.16	0.07	-2.33	0.0225145
218422_s_at	64062	-0.14	0.06	-2.33	0.0225658
228670_at	7011	0.14	0.06	2.33	0.0226567
227039_at	11214	-0.19	0.08	-2.33	0.0227025
217751_at	373156	0.11	0.05	2.33	0.0227253
203308_x_at	3257	0.11	0.05	2.33	0.0227709
222593_s_at	65244	-0.13	0.06	-2.33	0.0227985
202406_s_at	7073	0.08	0.04	2.33	0.022834
218486_at	8462	0.14	0.06	2.33	0.0228419
205843_x_at	1384	0.13	0.05	2.33	0.0228663
202626_s_at	4067	0.17	0.07	2.33	0.0228673
237390_at	148	0.53	0.23	2.33	0.0228839
203883_s_at	22841	-0.15	0.06	-2.33	0.0228885
212127_at	5905	0.12	0.05	2.33	0.0229154
218771_at	55229	0.11	0.05	2.33	0.0229536
212796_s_at	23102	0.11	0.05	2.33	0.0229649
221809_at	57610	0.13	0.06	2.32	0.0230233
227584_at	89796	0.14	0.06	2.32	0.0230321
202944_at	4668	0.16	0.07	2.32	0.0230481
222426_at	79109	0.14	0.06	2.32	0.0230704
210428_s_at	9146	0.16	0.07	2.32	0.0230713
224931_at	54946	-0.11	0.05	-2.32	0.0230823
208611_s_at	6709	0.13	0.06	2.32	0.0230948
235692_at	389840	0.15	0.07	2.32	0.0231442
212341_at	286451	-0.21	0.09	-2.32	0.0231814
214092_x_at	10147	0.13	0.05	2.32	0.0231865
221606_s_at	79366	0.18	0.08	2.32	0.0231882
243435_at	10984	-0.17	0.07	-2.32	0.0231957
217985_s_at	11177	-0.17	0.07	-2.32	0.0232105
218524_at	1877	0.11	0.05	2.32	0.023244
1552274_at	54899	-0.14	0.06	-2.32	0.0232625
217853_at	64759	0.21	0.09	2.32	0.0232774
208109_s_at	81698	-0.26	0.11	-2.32	0.0233036
212339_at	2036	0.26	0.11	2.32	0.0233368
214212_x_at	10979	-0.24	0.10	-2.32	0.0233685
236361_at	117248	-0.26	0.11	-2.32	0.0233931
57082_at	26119	0.12	0.05	2.32	0.0234077
227383_at	57234	-0.24	0.10	-2.32	0.0234234

205341_at	30846	0.16	0.07	2.32	0.0234238
225744_at	29801	0.14	0.06	2.32	0.0234398
233173_x_at	9328	0.10	0.04	2.32	0.023548
230454_at	130026	0.16	0.07	2.31	0.0235817
207438_s_at	10073	0.08	0.03	2.31	0.0235848
224469_s_at	84800	0.20	0.09	2.31	0.0236103
212611_at	23220	0.20	0.09	2.31	0.0236155
204790_at	4092	0.25	0.11	2.31	0.0236271
207667_s_at	5606	0.14	0.06	2.31	0.0236293
207761_s_at	25840	0.11	0.05	2.31	0.0236338
206592_s_at	8943	0.14	0.06	2.31	0.0236591
213026_at	9140	-0.15	0.07	-2.31	0.0236963
230097_at	2618	-0.27	0.12	-2.31	0.0237101
202215_s_at	4802	0.12	0.05	2.31	0.0237179
208673_s_at	6428	-0.22	0.10	-2.31	0.0237354
221882_s_at	58986	0.15	0.06	2.31	0.0237607
227064_at	91369	0.14	0.06	2.31	0.0237663
242358_at	NA	0.16	0.07	2.31	0.023776
202361_at	9632	0.12	0.05	2.31	0.0237942
217992_s_at	79180	0.18	0.08	2.31	0.0238133
203247_s_at	7572	-0.23	0.10	-2.31	0.0238226
238468_at	23112	-0.21	0.09	-2.31	0.0238346
218197_s_at	55074	-0.18	0.08	-2.31	0.0238844
231879_at	1303	0.24	0.10	2.31	0.0239231
228284_at	7088	0.21	0.09	2.31	0.0239541
217844_at	58190	0.07	0.03	2.31	0.0239661
221802_s_at	57698	0.19	0.08	2.31	0.024016
242699_at	23414	-0.23	0.10	-2.31	0.0240676
200051_at	9092	0.12	0.05	2.31	0.0240766
218466_at	79735	0.12	0.05	2.30	0.0241387
215012_at	26036	-0.19	0.08	-2.30	0.0241767
223422_s_at	83478	0.13	0.06	2.30	0.0242141
202073_at	10133	0.15	0.06	2.30	0.0242387
223607_x_at	90204	0.14	0.06	2.30	0.0242783
55065_at	57787	0.16	0.07	2.30	0.0242914
207172_s_at	1009	0.18	0.08	2.30	0.0243052
226214_at	51573	0.13	0.06	2.30	0.0243099
204300_at	5188	0.16	0.07	2.30	0.0243258
203353_s_at	4152	0.10	0.04	2.30	0.0243612
226408_at	8463	0.11	0.05	2.30	0.0243619
201654_s_at	3339	0.21	0.09	2.30	0.0243671
205427_at	6940	0.12	0.05	2.30	0.0243791
211043_s_at	1212	0.09	0.04	2.30	0.0243869
57532_at	1856	0.08	0.04	2.30	0.0244463
218307_at	55316	0.10	0.04	2.30	0.0244829
208751_at	8775	0.16	0.07	2.30	0.024487
227540_at	60678	0.10	0.04	2.30	0.0245057
224952_at	26115	0.17	0.07	2.30	0.0245082
228506_at	54780	0.17	0.08	2.30	0.0245099
205787_x_at	9877	-0.16	0.07	-2.30	0.0245361
228466_at	NA	0.11	0.05	2.30	0.0245634
215439_x_at	171024	-0.13	0.05	-2.30	0.0246275

213037_x_at	6780	0.07	0.03	2.30	0.0246318
204912_at	3587	0.24	0.10	2.30	0.0246497
219833_s_at	114327	0.17	0.07	2.30	0.0246946
226742_at	51128	0.10	0.04	2.30	0.0247081
210592_s_at	6303	0.15	0.06	2.30	0.0247217
215498_s_at	5606	0.18	0.08	2.30	0.0247238
212948_at	23125	0.15	0.06	2.30	0.024728
203905_at	5073	0.08	0.04	2.30	0.0247302
229817_at	57507	0.13	0.06	2.29	0.0247815
215235_at	6709	0.14	0.06	2.29	0.0247859
203169_at	9827	0.10	0.04	2.29	0.0247995
48106_at	55652	0.11	0.05	2.29	0.024908
227649_s_at	23380	0.13	0.06	2.29	0.024927
239331_at	NA	-0.20	0.09	-2.29	0.0249501
218240_at	28511	0.12	0.05	2.29	0.0249902
232716_at	1902	-0.29	0.13	-2.29	0.0249971
239619_at	55893	-0.27	0.12	-2.29	0.0250167
225571_at	3977	-0.42	0.19	-2.29	0.0250171
235190_at	808	-0.20	0.09	-2.29	0.0250179
223467_at	51655	0.30	0.13	2.29	0.0250192
243428_at	10984	-0.15	0.06	-2.29	0.0250255
220925_at	60560	-0.19	0.08	-2.29	0.0250739
222550_at	55156	-0.11	0.05	-2.29	0.0250828
212146_at	23207	0.12	0.05	2.29	0.025095
219571_s_at	7559	-0.21	0.09	-2.29	0.0251179
238538_at	29123	0.12	0.05	2.29	0.025143
55583_at	57572	0.18	0.08	2.29	0.0251628
209420_s_at	6609	0.12	0.05	2.29	0.0252201
212095_s_at	57509	-0.23	0.10	-2.29	0.0252277
220419_s_at	29761	0.09	0.04	2.29	0.0252314
214273_x_at	8131	0.15	0.06	2.29	0.0252456
202315_s_at	613	0.20	0.09	2.29	0.0252548
237337_at	NA	0.14	0.06	2.29	0.0252982
223773_s_at	85028	0.18	0.08	2.29	0.0253118
202771_at	9780	0.17	0.07	2.29	0.0253225
1552295_a_at	91252	0.12	0.05	2.29	0.025338
201455_s_at	9520	0.10	0.04	2.29	0.0253424
226849_at	57706	0.15	0.07	2.28	0.0253821
227547_at	NA	0.12	0.05	2.28	0.0253858
211284_s_at	2896	0.14	0.06	2.28	0.0254057
222266_at	8725	-0.23	0.10	-2.28	0.0254106
217861_s_at	10113	0.15	0.07	2.28	0.0254177
204114_at	22795	0.17	0.07	2.28	0.0254218
200839_s_at	1508	0.19	0.08	2.28	0.0255239
208325_s_at	11214	-0.22	0.10	-2.28	0.0255809
228738_at	257054	0.12	0.05	2.28	0.0256065
219603_s_at	7769	0.21	0.09	2.28	0.0256689
203474_at	10788	0.28	0.12	2.28	0.0257223
206782_s_at	3338	0.13	0.06	2.28	0.0257681
215559_at	368	0.38	0.17	2.28	0.0258152
227093_at	57602	0.15	0.07	2.28	0.0258193
230266_at	338382	0.17	0.07	2.28	0.0258343

230637_at	119559	-0.16	0.07	-2.28	0.0258389
201834_at	5564	0.12	0.05	2.28	0.0258523
74694_s_at	79874	0.17	0.07	2.28	0.0258633
233800_at	163	-0.12	0.05	-2.28	0.0258708
212394_at	23065	0.10	0.04	2.28	0.0258878
215031_x_at	55658	0.11	0.05	2.28	0.025892
223126_s_at	81563	-0.16	0.07	-2.28	0.0258923
217550_at	22926	-0.15	0.07	-2.28	0.0258999
200914_x_at	3895	-0.12	0.05	-2.28	0.0259302
218985_at	29988	0.11	0.05	2.28	0.0259471
228402_at	84327	0.18	0.08	2.28	0.0259509
202380_s_at	4820	0.12	0.05	2.28	0.0259627
212027_at	58517	-0.24	0.10	-2.27	0.0260031
200977_s_at	8887	0.09	0.04	2.27	0.0260183
209118_s_at	7846	-0.20	0.09	-2.27	0.0260185
201979_s_at	5536	0.12	0.05	2.27	0.026028
213073_at	23503	0.11	0.05	2.27	0.0260291
201017_at	1964	-0.24	0.11	-2.27	0.0260669
228054_at	93109	0.15	0.07	2.27	0.0260674
224571_at	359948	-0.20	0.09	-2.27	0.0260967
38964_r_at	7454	0.14	0.06	2.27	0.0261042
226793_at	283267	0.13	0.06	2.27	0.026125
227961_at	1508	0.26	0.12	2.27	0.0261688
226656_at	10491	0.19	0.09	2.27	0.0261899
233085_s_at	64859	0.27	0.12	2.27	0.0261944
218632_at	79654	0.15	0.06	2.27	0.0262259
52164_at	53838	0.12	0.05	2.27	0.0262266
215111_s_at	8848	-0.15	0.06	-2.27	0.0262266
224849_at	55761	0.10	0.04	2.27	0.0262479
204520_x_at	23774	0.10	0.05	2.27	0.0262515
221063_x_at	63891	0.16	0.07	2.27	0.0262888
219818_s_at	55094	0.13	0.06	2.27	0.026302
202764_at	6786	0.10	0.04	2.27	0.0263753
224804_s_at	57184	0.11	0.05	2.27	0.0264964
218573_at	28986	-0.14	0.06	-2.27	0.0265429
242485_at	5747	-0.13	0.06	-2.27	0.0265956
223138_s_at	170506	-0.15	0.07	-2.26	0.0266166
225926_at	10490	0.20	0.09	2.26	0.0266387
203175_at	391	0.13	0.06	2.26	0.026664
225947_at	80179	0.16	0.07	2.26	0.0267008
36566_at	1497	0.12	0.05	2.26	0.0267602
237383_at	NA	-0.15	0.06	-2.26	0.0268236
219372_at	28981	0.14	0.06	2.26	0.026853
205752_s_at	2949	-0.23	0.10	-2.26	0.026885
229066_at	NA	0.15	0.06	2.26	0.0269687
232597_x_at	9169	-0.16	0.07	-2.26	0.0269809
225120_at	5814	0.09	0.04	2.26	0.0269843
205821_at	22914	0.25	0.11	2.26	0.0270095
219627_at	79970	0.14	0.06	2.26	0.0270365
200055_at	6881	0.13	0.06	2.26	0.0270457
224686_x_at	474170	-0.13	0.06	-2.26	0.0270969
231968_at	56886	0.10	0.05	2.26	0.0271047

239050_s_at	NA	-0.18	0.08	-2.26	0.0271158
229497_at	348094	0.20	0.09	2.26	0.027128
204057_at	3394	0.28	0.12	2.26	0.027131
209342_s_at	3551	0.11	0.05	2.26	0.0271454
223389_s_at	51545	0.10	0.04	2.26	0.0271511
218970_s_at	51076	0.14	0.06	2.26	0.0271776
235670_at	NA	0.25	0.11	2.26	0.0271932
213649_at	6432	-0.15	0.07	-2.26	0.0271934
239243_at	27332	-0.32	0.14	-2.26	0.0272304
1552309_a_at	91624	0.21	0.09	2.26	0.0272545
235642_at	148	0.52	0.23	2.25	0.0272769
208625_s_at	1981	0.14	0.06	2.25	0.0272855
209024_s_at	10492	-0.16	0.07	-2.25	0.0273845
209199_s_at	4208	-0.13	0.06	-2.25	0.0274199
224605_at	401152	-0.14	0.06	-2.25	0.0274282
226786_at	5989	0.15	0.07	2.25	0.0274315
229253_at	117145	0.14	0.06	2.25	0.0274645
202202_s_at	3910	0.11	0.05	2.25	0.0275222
227538_at	9441	0.14	0.06	2.25	0.0275247
202106_at	2802	0.12	0.05	2.25	0.0275349
206555_s_at	55623	-0.15	0.07	-2.25	0.0276273
224690_at	116151	0.11	0.05	2.25	0.0276503
236545_at	5530	-0.38	0.17	-2.25	0.0276548
210178_x_at	10772	-0.17	0.07	-2.25	0.0277161
217829_s_at	10713	0.08	0.04	2.25	0.0277366
215049_x_at	9332	0.32	0.14	2.25	0.0277458
243801_x_at	51263	-0.13	0.06	-2.25	0.0278715
204568_at	22863	0.12	0.05	2.25	0.0278763
218793_s_at	6322	0.14	0.06	2.25	0.0278899
221196_x_at	79184	0.11	0.05	2.24	0.027931
203143_s_at	9674	0.18	0.08	2.24	0.027997
227009_at	NA	0.11	0.05	2.24	0.0280527
225576_at	116254	-0.10	0.04	-2.24	0.0280793
204454_at	23641	0.16	0.07	2.24	0.0280997
227841_at	51005	0.13	0.06	2.24	0.0281181
212968_at	5986	0.11	0.05	2.24	0.0281297
217437_s_at	6867	-0.16	0.07	-2.24	0.0282163
221856_s_at	55793	-0.13	0.06	-2.24	0.0282644
36004_at	8517	0.10	0.05	2.24	0.0283246
213463_s_at	317662	0.12	0.05	2.24	0.0283288
209127_s_at	9733	-0.18	0.08	-2.24	0.0283824
224378_x_at	84557	0.10	0.05	2.24	0.0284203
209241_x_at	50488	0.18	0.08	2.24	0.0284269
226738_at	124997	0.11	0.05	2.24	0.0284385
212465_at	84193	0.09	0.04	2.24	0.0284443
213300_at	23130	0.14	0.06	2.24	0.0284761
235046_at	NA	-0.32	0.14	-2.24	0.0285977
204461_x_at	5810	-0.16	0.07	-2.23	0.0286294
222156_x_at	9236	0.21	0.09	2.23	0.0286916
235810_at	7569	0.15	0.07	2.23	0.0287024
223394_at	29950	-0.11	0.05	-2.23	0.0287061
210542_s_at	28232	0.11	0.05	2.23	0.0287092

228754_at	6533	0.21	0.09	2.23	0.02872
239039_at	23452	0.19	0.08	2.23	0.0287712
208913_at	23062	0.10	0.04	2.23	0.0287811
237778_at	1730	-0.24	0.11	-2.23	0.0288144
215038_s_at	29072	0.12	0.05	2.23	0.0288204
229404_at	117581	0.16	0.07	2.23	0.028842
200976_s_at	8887	0.09	0.04	2.23	0.0288473
218006_s_at	7570	-0.19	0.08	-2.23	0.0288825
225959_s_at	84937	0.12	0.06	2.23	0.0288917
232471_at	4643	-0.15	0.07	-2.23	0.0289402
223464_at	114879	0.10	0.05	2.23	0.0289406
219639_x_at	56965	0.07	0.03	2.23	0.028944
202550_s_at	9217	0.09	0.04	2.23	0.0290108
218378_s_at	79706	0.11	0.05	2.23	0.0290395
229126_at	55266	0.12	0.05	2.23	0.0290687
212987_at	26268	0.10	0.04	2.23	0.0290834
226076_s_at	114785	0.18	0.08	2.23	0.0291012
233571_x_at	79144	0.21	0.09	2.23	0.0291298
216036_x_at	23038	0.15	0.07	2.23	0.0291432
219025_at	57124	0.21	0.09	2.23	0.0291473
241376_at	NA	-0.26	0.11	-2.23	0.0291637
244648_at	54520	-0.21	0.09	-2.23	0.0292213
206491_s_at	8775	0.15	0.07	2.23	0.0292292
231199_at	80218	-0.24	0.11	-2.23	0.0292418
223640_at	10870	0.19	0.09	2.23	0.0292648
230099_at	6459	-0.23	0.10	-2.23	0.0292817
238039_at	9522	0.13	0.06	2.23	0.0293017
221229_s_at	55006	0.13	0.06	2.23	0.0293032
226834_at	79827	0.21	0.10	2.22	0.0293171
212677_s_at	23177	-0.19	0.09	-2.22	0.0293409
216863_s_at	22880	0.13	0.06	2.22	0.0293463
228722_at	7180	0.12	0.05	2.22	0.0293931
206833_s_at	98	0.23	0.10	2.22	0.0294631
203547_at	920	0.17	0.08	2.22	0.0294856
206958_s_at	65110	0.14	0.06	2.22	0.0294889
225083_at	112495	0.11	0.05	2.22	0.0294957
236439_at	604	-0.41	0.19	-2.22	0.0294973
200705_s_at	1933	-0.19	0.09	-2.22	0.0295297
207625_s_at	9139	0.09	0.04	2.22	0.0296289
222151_s_at	80254	0.18	0.08	2.22	0.0296963
222995_s_at	57414	0.14	0.06	2.22	0.0297075
226498_at	2321	-0.19	0.09	-2.22	0.029721
225124_at	84687	0.16	0.07	2.22	0.0297505
220933_s_at	79670	0.10	0.04	2.22	0.0297606
212748_at	57591	0.14	0.06	2.22	0.0297677
225401_at	112770	0.15	0.07	2.22	0.0297805
226315_at	91603	-0.09	0.04	-2.22	0.029785
238619_at	NA	-0.22	0.10	-2.22	0.0297886
202645_s_at	4221	0.13	0.06	2.22	0.0298054
200613_at	1173	0.10	0.04	2.22	0.0298089
201788_at	11325	0.11	0.05	2.22	0.029837
209122_at	123	0.21	0.09	2.22	0.0298749

204881_s_at	7357	-0.26	0.12	-2.22	0.0298847
226156_at	NA	0.13	0.06	2.22	0.0298933
204242_s_at	8310	0.12	0.05	2.22	0.0299373
221988_at	79086	0.11	0.05	2.22	0.0299488
235252_at	8844	0.14	0.06	2.22	0.0299985
204774_at	2123	0.29	0.13	2.22	0.03
209820_s_at	10607	0.09	0.04	2.22	0.0300012
229007_at	283788	0.30	0.14	2.22	0.0300015
238835_at	NA	-0.26	0.12	-2.22	0.0300086
203758_at	1519	-0.13	0.06	-2.21	0.0300181
202332_at	1454	0.14	0.06	2.21	0.0300189
218142_s_at	51185	-0.15	0.07	-2.21	0.0301163
212332_at	5934	-0.15	0.07	-2.21	0.0301428
208610_s_at	23524	-0.20	0.09	-2.21	0.030149
201225_s_at	10250	0.11	0.05	2.21	0.0301694
228700_at	159013	0.11	0.05	2.21	0.0301945
226242_at	128061	0.09	0.04	2.21	0.0302222
205583_s_at	79868	0.13	0.06	2.21	0.0302279
210649_s_at	8289	0.17	0.08	2.21	0.0302492
215293_s_at	27315	0.11	0.05	2.21	0.0302811
211663_x_at	5730	0.23	0.11	2.21	0.0303649
206159_at	2662	0.27	0.12	2.21	0.0303867
222807_at	56946	-0.14	0.07	-2.21	0.0304083
222033_s_at	2321	-0.15	0.07	-2.21	0.03044
204225_at	9759	-0.15	0.07	-2.21	0.0304435
206028_s_at	10461	0.21	0.10	2.21	0.0305406
201013_s_at	10606	0.13	0.06	2.21	0.0305717
209187_at	1810	-0.19	0.09	-2.21	0.0305752
205236_x_at	6649	0.20	0.09	2.21	0.0306024
212197_x_at	23164	0.12	0.05	2.21	0.0306455
203055_s_at	9138	0.13	0.06	2.21	0.0307139
213274_s_at	1508	0.25	0.11	2.21	0.0307249
229033_s_at	84939	-0.15	0.07	-2.21	0.0307342
222544_s_at	54904	-0.10	0.05	-2.21	0.0307349
219511_s_at	9627	0.18	0.08	2.21	0.0307402
201371_s_at	8452	0.07	0.03	2.20	0.0307601
226345_at	221079	-0.15	0.07	-2.20	0.0307813
204342_at	29957	-0.20	0.09	-2.20	0.0308274
33132_at	29894	0.24	0.11	2.20	0.0308377
205478_at	5502	0.27	0.12	2.20	0.0308551
239404_at	7150	-0.19	0.09	-2.20	0.0308813
226074_at	132160	0.10	0.04	2.20	0.030899
208829_at	6892	0.17	0.08	2.20	0.0309028
214081_at	57125	0.27	0.12	2.20	0.0309092
225697_at	51755	-0.12	0.05	-2.20	0.0309119
205809_s_at	8976	-0.19	0.08	-2.20	0.0309585
41858_at	27315	0.11	0.05	2.20	0.0310151
210128_s_at	1241	0.14	0.06	2.20	0.0310434
220610_s_at	9209	0.09	0.04	2.20	0.0310567
232144_at	5087	-0.19	0.09	-2.20	0.0310649
33778_at	25771	-0.08	0.04	-2.20	0.0311293
225577_at	414777	0.14	0.07	2.20	0.0311309

52169_at	92335	0.08	0.04	2.20	0.0311309
211168_s_at	5976	0.11	0.05	2.20	0.0311661
224982_at	84335	0.16	0.07	2.20	0.0311826
224847_at	1021	0.14	0.06	2.20	0.0312183
200948_at	8079	0.14	0.06	2.20	0.0312452
62212_at	79078	-0.09	0.04	-2.20	0.031286
209170_s_at	2824	-0.26	0.12	-2.20	0.031289
217066_s_at	1760	0.16	0.07	2.20	0.0313468
209481_at	54861	-0.14	0.06	-2.20	0.0313479
229467_at	5094	-0.13	0.06	-2.20	0.0314201
40489_at	1822	0.17	0.08	2.19	0.0314926
225041_at	54737	-0.17	0.08	-2.19	0.0316132
208792_s_at	1191	0.15	0.07	2.19	0.0316337
223251_s_at	55608	0.16	0.07	2.19	0.0316726
225860_at	NA	0.18	0.08	2.19	0.0317194
225464_at	122786	-0.23	0.11	-2.19	0.0317334
213091_at	23373	0.15	0.07	2.19	0.0318038
205452_at	9488	0.15	0.07	2.19	0.031806
203517_at	10651	0.12	0.06	2.19	0.0318125
201589_at	8243	-0.14	0.06	-2.19	0.0318237
218780_at	29911	0.25	0.11	2.19	0.0318372
58780_s_at	55701	0.17	0.08	2.19	0.0318663
51192_at	54961	0.10	0.05	2.19	0.0319409
232160_s_at	79155	0.09	0.04	2.19	0.0319531
202689_at	29890	0.11	0.05	2.19	0.0319675
221269_s_at	83442	0.17	0.08	2.19	0.0319789
209164_s_at	1534	-0.13	0.06	-2.19	0.0319976
234005_x_at	27148	0.11	0.05	2.19	0.0320243
218604_at	23592	-0.15	0.07	-2.19	0.0321161
228568_at	145781	0.25	0.11	2.19	0.0321196
222143_s_at	64419	0.08	0.03	2.19	0.0321573
232898_at	1601	-0.22	0.10	-2.19	0.0321882
212385_at	6925	-0.18	0.08	-2.18	0.0322391
203636_at	4281	0.16	0.07	2.18	0.0322589
212329_at	22937	0.11	0.05	2.18	0.0323781
213145_at	144699	-0.12	0.05	-2.18	0.0323929
243310_at	NA	-0.15	0.07	-2.18	0.0324763
227406_at	2553	-0.11	0.05	-2.18	0.0325402
203633_at	1374	0.18	0.08	2.18	0.032545
203977_at	6901	0.14	0.06	2.18	0.0326063
233986_s_at	64857	0.15	0.07	2.18	0.0326205
223852_s_at	83931	0.16	0.07	2.18	0.0326221
201514_s_at	10146	-0.13	0.06	-2.18	0.0326378
204355_at	22907	0.12	0.06	2.18	0.0326465
222105_s_at	28511	0.12	0.06	2.18	0.0326512
239296_at	9839	-0.23	0.11	-2.18	0.0326701
232808_at	84168	-0.16	0.07	-2.18	0.0327319
226195_at	112752	0.11	0.05	2.18	0.0327354
236901_at	9509	0.28	0.13	2.18	0.032752
212457_at	7030	0.13	0.06	2.18	0.0327613
200089_s_at	6124	-0.10	0.04	-2.18	0.0327855
208819_at	4218	0.10	0.05	2.18	0.0327913

242500_at	NA	-0.24	0.11	-2.18	0.0327943
225605_at	90313	0.13	0.06	2.18	0.032819
229531_at	401612	0.14	0.06	2.18	0.0328214
215028_at	57556	-0.31	0.14	-2.18	0.0328658
212358_at	25999	0.19	0.09	2.18	0.0329073
202189_x_at	5725	0.11	0.05	2.18	0.0329477
218032_at	8303	0.16	0.07	2.17	0.0330867
229218_at	1278	-0.27	0.12	-2.17	0.0331006
203831_at	22864	0.09	0.04	2.17	0.0331031
224858_at	25921	0.08	0.04	2.17	0.0331364
218593_at	55131	0.13	0.06	2.17	0.0331583
240134_at	55841	-0.10	0.05	-2.17	0.0331925
235901_at	NA	0.17	0.08	2.17	0.0332189
228601_at	401022	0.18	0.09	2.17	0.0332342
204425_at	393	0.15	0.07	2.17	0.0332361
225562_at	22821	0.17	0.08	2.17	0.0332549
229544_at	NA	0.20	0.09	2.17	0.0333467
215177_s_at	3655	-0.21	0.10	-2.17	0.0334091
202896_s_at	140885	0.15	0.07	2.17	0.0334319
218961_s_at	11284	0.14	0.06	2.17	0.0334811
230606_at	125111	0.14	0.07	2.17	0.0334882
218740_s_at	80279	0.13	0.06	2.17	0.0335114
209376_x_at	9169	-0.16	0.07	-2.17	0.0335728
226035_at	57478	-0.23	0.11	-2.17	0.033665
201687_s_at	8539	-0.10	0.05	-2.17	0.0336915
213154_s_at	23299	0.13	0.06	2.17	0.0337077
213488_at	25992	0.17	0.08	2.17	0.0337269
218615_s_at	55254	-0.15	0.07	-2.17	0.033782
227454_at	57551	-0.23	0.11	-2.16	0.0338333
222554_s_at	65083	0.13	0.06	2.16	0.0338862
200765_x_at	1495	0.09	0.04	2.16	0.033893
235054_at	131870	0.14	0.07	2.16	0.0339545
213445_at	23144	0.15	0.07	2.16	0.0339595
236300_at	5139	-0.24	0.11	-2.16	0.033969
203761_at	6503	0.20	0.09	2.16	0.0339885
208831_x_at	6830	0.13	0.06	2.16	0.0339977
225102_at	11343	0.18	0.08	2.16	0.0340024
202762_at	9475	0.12	0.05	2.16	0.0340079
209253_at	10174	0.16	0.07	2.16	0.0340219
227281_at	222962	0.22	0.10	2.16	0.0340642
212176_at	25957	0.13	0.06	2.16	0.0340663
222708_s_at	55014	-0.12	0.06	-2.16	0.0341137
219027_s_at	4649	-0.14	0.07	-2.16	0.0341174
242181_at	NA	0.29	0.13	2.16	0.0341324
226501_at	63929	0.13	0.06	2.16	0.0341487
200045_at	23	0.14	0.07	2.16	0.0341564
201108_s_at	7057	0.31	0.14	2.16	0.0341564
211932_at	220988	-0.12	0.06	-2.16	0.0341574
213428_s_at	1291	0.15	0.07	2.16	0.0341612
202231_at	10480	-0.15	0.07	-2.16	0.0341797
208981_at	5175	-0.13	0.06	-2.16	0.0342127
203221_at	7088	0.18	0.08	2.16	0.0342234

228562_at	65986	-0.20	0.09	-2.16	0.0343514
219431_at	79658	0.11	0.05	2.16	0.0343621
200678_x_at	2896	0.15	0.07	2.16	0.0343722
201714_at	7283	0.21	0.10	2.16	0.0343848
202135_s_at	10120	0.07	0.03	2.16	0.0344216
211769_x_at	10955	-0.15	0.07	-2.16	0.034435
224455_s_at	83440	0.12	0.06	2.16	0.0344405
226833_at	124637	0.13	0.06	2.16	0.0344516
202963_at	5993	0.11	0.05	2.16	0.0345285
225383_at	10838	0.13	0.06	2.16	0.0345308
214771_x_at	23164	0.14	0.07	2.16	0.0345382
212202_s_at	25963	-0.12	0.06	-2.16	0.0345879
201208_s_at	7126	-0.10	0.05	-2.15	0.0346555
236154_at	9444	0.17	0.08	2.15	0.0346634
225077_at	283680	0.10	0.05	2.15	0.0346734
222465_at	51187	-0.21	0.10	-2.15	0.0347322
224577_at	57222	0.14	0.07	2.15	0.0347553
212675_s_at	23177	-0.12	0.05	-2.15	0.0347962
214703_s_at	23324	0.15	0.07	2.15	0.0348004
205666_at	2326	0.21	0.10	2.15	0.034843
201534_s_at	5412	-0.15	0.07	-2.15	0.0348608
205125_at	5333	0.11	0.05	2.15	0.0348776
230319_at	79625	-0.38	0.17	-2.15	0.0349842
238613_at	51776	-0.17	0.08	-2.15	0.0351255
230405_at	NA	0.11	0.05	2.15	0.0351273
222907_x_at	757	-0.18	0.08	-2.15	0.0351794
204313_s_at	1385	-0.20	0.09	-2.15	0.0353351
201299_s_at	55233	-0.21	0.10	-2.15	0.0353782
235616_at	128553	-0.16	0.08	-2.15	0.035413
50376_at	55311	0.09	0.04	2.14	0.0354749
225017_at	64770	0.15	0.07	2.14	0.0355129
213654_at	27097	0.07	0.03	2.14	0.0355292
210346_s_at	57396	-0.14	0.06	-2.14	0.0355359
222589_at	51701	0.15	0.07	2.14	0.0355791
228494_at	55607	0.21	0.10	2.14	0.0355987
221759_at	92579	0.12	0.06	2.14	0.0356049
200838_at	1508	0.20	0.09	2.14	0.0356077
226676_at	25925	-0.16	0.07	-2.14	0.0356405
50277_at	26088	0.08	0.04	2.14	0.0356435
227065_at	54476	0.10	0.04	2.14	0.0357526
232312_at	55291	-0.15	0.07	-2.14	0.0357633
216246_at	6224	-0.11	0.05	-2.14	0.0357786
226818_at	219972	0.27	0.13	2.14	0.0358168
217984_at	8635	0.14	0.07	2.14	0.0358287
229351_at	29964	0.10	0.05	2.14	0.0358312
202172_at	7716	-0.11	0.05	-2.14	0.0358625
226594_at	51004	0.11	0.05	2.14	0.03587
212702_s_at	23299	0.13	0.06	2.14	0.0359138
227852_at	6100	-0.11	0.05	-2.14	0.0359233
202048_s_at	23466	0.08	0.04	2.14	0.0359343
223420_at	85406	0.10	0.05	2.14	0.0359769
219197_s_at	57758	0.30	0.14	2.14	0.0360112

218559_s_at	9935	0.16	0.08	2.14	0.0360172
238902_at	115294	-0.29	0.14	-2.14	0.0360289
1552287_s_at	172	0.15	0.07	2.14	0.0361719
211671_s_at	2908	-0.21	0.10	-2.14	0.0361736
210443_x_at	11054	0.12	0.06	2.14	0.0361993
201667_at	2697	-0.24	0.11	-2.14	0.0362245
235044_at	116159	-0.15	0.07	-2.13	0.0362673
225660_at	57556	-0.17	0.08	-2.13	0.0363352
218016_s_at	55718	0.10	0.05	2.13	0.0363711
203600_s_at	8603	0.14	0.07	2.13	0.0364028
202795_x_at	11078	0.10	0.05	2.13	0.0364141
208851_s_at	7070	0.20	0.10	2.13	0.0364594
224813_at	8976	-0.11	0.05	-2.13	0.0364726
209488_s_at	11030	0.21	0.10	2.13	0.0364799
221564_at	7180	0.09	0.04	2.13	0.0364874
201516_at	6723	0.11	0.05	2.13	0.0365268
201895_at	369	0.11	0.05	2.13	0.0365344
202188_at	9688	0.08	0.04	2.13	0.036544
222732_at	56658	0.10	0.05	2.13	0.0365458
242143_at	10048	-0.20	0.09	-2.13	0.0365607
213784_at	11020	0.12	0.06	2.13	0.0365929
223446_s_at	84062	0.11	0.05	2.13	0.0366281
244287_at	140890	-0.14	0.07	-2.13	0.0366301
225770_at	89970	-0.09	0.04	-2.13	0.0366365
213097_s_at	27000	0.12	0.06	2.13	0.036683
220486_x_at	84187	0.17	0.08	2.13	0.0366836
227792_at	162073	0.09	0.04	2.13	0.0366986
200024_at	6193	-0.21	0.10	-2.13	0.0367006
211783_s_at	9112	0.15	0.07	2.13	0.036721
202602_s_at	27336	0.09	0.04	2.13	0.0367782
203104_at	1436	0.22	0.10	2.13	0.0368142
212485_at	23131	0.09	0.04	2.13	0.0368238
208929_x_at	6137	-0.12	0.06	-2.13	0.0368329
235349_at	151393	0.10	0.05	2.13	0.0368719
229086_at	148898	0.18	0.08	2.13	0.0368885
217858_s_at	51566	-0.09	0.04	-2.13	0.036931
234969_s_at	80314	-0.10	0.05	-2.13	0.036935
204755_x_at	3131	-0.29	0.13	-2.13	0.0369451
208697_s_at	3646	-0.12	0.06	-2.13	0.0370112
201309_x_at	9315	-0.19	0.09	-2.13	0.0370693
207705_s_at	22981	0.12	0.05	2.13	0.0370804
209500_x_at	407977	0.13	0.06	2.12	0.0371551
214152_at	9236	0.15	0.07	2.12	0.0372149
200936_at	6132	-0.18	0.08	-2.12	0.0372746
201939_at	10769	-0.23	0.11	-2.12	0.0372781
210438_x_at	6738	-0.18	0.08	-2.12	0.0372848
223562_at	64098	0.20	0.10	2.12	0.0373442
209395_at	1116	0.76	0.36	2.12	0.0374097
229211_at	285193	0.10	0.05	2.12	0.0374109
223314_at	81619	0.16	0.07	2.12	0.0374229
225949_at	340371	0.15	0.07	2.12	0.0374622
201419_at	8314	0.10	0.05	2.12	0.0374725

229342_at	NA	0.12	0.06	2.12	0.0374849
225223_at	4090	-0.16	0.07	-2.12	0.0374889
218287_s_at	26523	0.12	0.05	2.12	0.0375056
244535_at	27086	-0.18	0.08	-2.12	0.0375213
208758_at	471	0.13	0.06	2.12	0.0375379
232925_at	1956	-0.16	0.08	-2.12	0.0375447
238868_at	55075	0.21	0.10	2.12	0.037571
226032_at	835	0.09	0.04	2.12	0.0375791
231876_at	81844	0.14	0.07	2.12	0.0375959
201790_s_at	1717	0.23	0.11	2.12	0.037596
219041_s_at	29803	0.14	0.07	2.12	0.0376302
229741_at	78993	0.20	0.09	2.12	0.0376391
237333_at	81493	-0.19	0.09	-2.12	0.0376797
202136_at	10771	-0.07	0.03	-2.12	0.0377228
202367_at	1523	0.18	0.09	2.12	0.0377276
228256_s_at	114915	0.11	0.05	2.12	0.0377585
AFFX-HSAC07/X00351_M_at	60	0.10	0.05	2.12	0.0377795
209727_at	2760	0.15	0.07	2.12	0.0378168
203117_s_at	9924	0.13	0.06	2.12	0.0378869
242476_at	NA	-0.29	0.14	-2.12	0.0379009
208907_s_at	28973	-0.15	0.07	-2.12	0.0379323
210075_at	51257	0.19	0.09	2.12	0.0379377
206846_s_at	10013	0.11	0.05	2.12	0.0379669
225257_at	90324	0.08	0.04	2.11	0.0380017
227485_at	203522	0.14	0.07	2.11	0.0380099
46665_at	54910	-0.10	0.05	-2.11	0.0380133
225819_at	84897	0.11	0.05	2.11	0.0380592
224973_at	55603	-0.31	0.15	-2.11	0.0380777
216264_s_at	3913	0.13	0.06	2.11	0.0382325
214241_at	4714	0.16	0.08	2.11	0.0382328
212506_at	8301	0.08	0.04	2.11	0.038277
212012_at	7837	0.18	0.09	2.11	0.038301
208255_s_at	23770	0.14	0.07	2.11	0.0383053
209156_s_at	1292	0.21	0.10	2.11	0.038357
238519_at	6248	-0.16	0.07	-2.11	0.0384088
219423_x_at	8718	0.26	0.12	2.11	0.0384331
208692_at	6188	-0.21	0.10	-2.11	0.0384392
218185_s_at	55156	-0.14	0.07	-2.11	0.0384428
228318_s_at	285464	0.16	0.08	2.11	0.0384765
215223_s_at	6648	0.24	0.11	2.11	0.0384864
201704_at	955	0.09	0.04	2.11	0.0385197
39854_r_at	57104	0.20	0.09	2.11	0.038529
243134_at	440309	-0.22	0.10	-2.11	0.0385624
203955_at	9858	0.11	0.05	2.11	0.0385694
40850_at	23770	0.13	0.06	2.11	0.0385958
209421_at	4436	0.20	0.09	2.11	0.0387007
201488_x_at	10657	-0.08	0.04	-2.11	0.0387399
204752_x_at	10038	0.11	0.05	2.11	0.038778
232369_at	10150	-0.26	0.13	-2.11	0.0387827
218137_s_at	60682	0.10	0.05	2.11	0.0388245
217830_s_at	55968	0.11	0.05	2.11	0.0388343
218450_at	50865	0.12	0.06	2.11	0.038837

204993_at	2781	0.15	0.07	2.11	0.0388678
241408_at	285464	0.14	0.07	2.11	0.0388714
202382_s_at	10007	0.13	0.06	2.11	0.0388727
222024_s_at	11214	-0.16	0.08	-2.11	0.0388759
235595_at	9181	-0.14	0.07	-2.10	0.038891
222653_at	55163	0.14	0.07	2.10	0.038924
233265_at	56288	-0.11	0.05	-2.10	0.0389655
203906_at	9922	0.12	0.05	2.10	0.0389815
216071_x_at	9968	0.15	0.07	2.10	0.0389953
222165_x_at	79095	0.08	0.04	2.10	0.0390151
228953_at	123720	0.14	0.06	2.10	0.0390246
214686_at	10781	-0.17	0.08	-2.10	0.0390547
226626_at	57187	0.10	0.05	2.10	0.039083
213746_s_at	2316	0.19	0.09	2.10	0.0391065
223068_at	27436	0.11	0.05	2.10	0.0391429
218415_at	26276	0.09	0.04	2.10	0.039206
227414_at	84236	0.13	0.06	2.10	0.0392682
209132_s_at	54939	0.11	0.05	2.10	0.0393352
204464_s_at	1909	-0.22	0.11	-2.10	0.039457
204537_s_at	2564	0.20	0.09	2.10	0.0394651
222686_s_at	55313	0.14	0.07	2.10	0.0395734
218757_s_at	65109	0.11	0.05	2.10	0.0396759
208675_s_at	1650	-0.11	0.05	-2.10	0.0396972
203249_at	2145	0.12	0.05	2.10	0.0397078
214794_at	5036	0.11	0.05	2.10	0.0397323
209665_at	11068	0.09	0.04	2.10	0.0397369
222709_at	10533	0.13	0.06	2.09	0.0397914
225573_at	84129	0.14	0.07	2.09	0.0398038
218249_at	64429	0.09	0.04	2.09	0.0398077
222146_s_at	6925	-0.16	0.08	-2.09	0.0398285
212334_at	2799	0.15	0.07	2.09	0.03984
209580_s_at	8930	0.11	0.05	2.09	0.039879
200836_s_at	4134	0.13	0.06	2.09	0.039957
238477_at	10749	0.17	0.08	2.09	0.0400046
203455_s_at	6303	0.14	0.07	2.09	0.0400621
230360_at	342035	0.28	0.13	2.09	0.0401008
226072_at	197258	0.09	0.04	2.09	0.040107
204779_s_at	3217	0.11	0.05	2.09	0.0401249
206600_s_at	9121	0.15	0.07	2.09	0.0401263
221005_s_at	81490	0.09	0.04	2.09	0.040127
219426_at	192669	-0.15	0.07	-2.09	0.0401297
202909_at	9852	-0.14	0.07	-2.09	0.0402114
214830_at	145389	0.22	0.10	2.09	0.040248
203321_s_at	22850	-0.13	0.06	-2.09	0.040399
243403_x_at	1368	0.31	0.15	2.09	0.0404787
228992_at	80306	-0.17	0.08	-2.09	0.0404843
215905_s_at	9410	-0.10	0.05	-2.09	0.0404904
225764_at	2120	0.16	0.08	2.09	0.0405607
34868_at	23381	0.09	0.04	2.09	0.04057
217771_at	51280	0.16	0.07	2.09	0.040605
227183_at	NA	0.20	0.10	2.09	0.0406539
200741_s_at	6232	-0.09	0.04	-2.09	0.0407044

202622_s_at	6311	0.12	0.06	2.08	0.0407403
209428_s_at	7542	0.10	0.05	2.08	0.0407957
242352_at	25836	-0.15	0.07	-2.08	0.0408035
219154_at	144404	0.26	0.13	2.08	0.0408091
202342_s_at	23321	-0.22	0.11	-2.08	0.0408351
213687_s_at	6165	-0.20	0.10	-2.08	0.0408663
201439_at	8729	0.15	0.07	2.08	0.0408764
221744_at	10238	-0.11	0.05	-2.08	0.0409159
203642_s_at	22837	0.17	0.08	2.08	0.0409273
235276_at	94240	0.28	0.14	2.08	0.0409623
212338_at	4642	0.28	0.13	2.08	0.0410385
208022_s_at	8555	-0.17	0.08	-2.08	0.0410705
229350_x_at	84875	0.10	0.05	2.08	0.0410746
210640_s_at	2852	0.15	0.07	2.08	0.0410812
212090_at	2907	0.16	0.08	2.08	0.0410945
219649_at	29929	0.13	0.06	2.08	0.0411497
203315_at	8440	0.09	0.04	2.08	0.0411581
213089_at	153561	-0.21	0.10	-2.08	0.0411711
204474_at	7701	0.13	0.06	2.08	0.041266
219922_s_at	4054	0.14	0.06	2.08	0.041272
221530_s_at	79365	0.20	0.10	2.08	0.0413007
208646_at	6208	-0.18	0.09	-2.08	0.0413101
228775_at	55831	0.14	0.07	2.08	0.0413721
218507_at	29923	0.23	0.11	2.08	0.0413765
226196_s_at	112752	0.10	0.05	2.08	0.041411
203766_s_at	25802	0.25	0.12	2.08	0.0414264
229563_s_at	4736	-0.13	0.06	-2.08	0.0414476
202390_s_at	3064	0.10	0.05	2.08	0.0414518
202055_at	3836	0.10	0.05	2.08	0.0415797
215947_s_at	84908	0.09	0.04	2.08	0.04158
212041_at	9114	0.11	0.05	2.08	0.0415896
213046_at	8106	0.13	0.06	2.08	0.0415922
202469_s_at	11052	0.10	0.05	2.08	0.0416282
231866_at	4012	-0.31	0.15	-2.08	0.0416329
203122_at	51112	0.11	0.05	2.07	0.041674
210573_s_at	10623	-0.10	0.05	-2.07	0.0416903
52975_at	89853	0.08	0.04	2.07	0.0417374
219384_s_at	23536	0.11	0.05	2.07	0.0417618
228023_x_at	55599	0.13	0.06	2.07	0.0417887
203346_s_at	22823	-0.13	0.06	-2.07	0.0417926
203338_at	5529	-0.14	0.07	-2.07	0.0417968
217869_at	51144	-0.15	0.07	-2.07	0.041823
205098_at	1230	0.30	0.14	2.07	0.0418343
203932_at	3109	0.14	0.07	2.07	0.0418557
224764_at	57584	0.11	0.05	2.07	0.0419501
221755_at	254102	0.14	0.07	2.07	0.0420849
201388_at	5709	0.11	0.06	2.07	0.0421385
208965_s_at	3428	-0.19	0.09	-2.07	0.0421632
232307_at	23389	-0.20	0.10	-2.07	0.0423029
224592_x_at	50809	0.08	0.04	2.07	0.0423135
204382_at	26151	0.10	0.05	2.07	0.0423172
235727_at	54813	-0.18	0.09	-2.07	0.0424007

213838_at	51406	0.08	0.04	2.07	0.0424091
210645_s_at	7267	0.09	0.04	2.07	0.0424554
241388_at	83452	-0.15	0.07	-2.07	0.0424775
212784_at	23152	0.14	0.07	2.07	0.0424776
210657_s_at	5414	0.15	0.07	2.07	0.0425097
226705_at	2260	0.09	0.04	2.07	0.0425244
212813_at	83700	-0.18	0.09	-2.07	0.0425406
201468_s_at	1728	0.33	0.16	2.07	0.0425771
207069_s_at	4091	0.19	0.09	2.07	0.0426177
219566_at	79156	0.13	0.06	2.07	0.0426266
201952_at	214	0.26	0.12	2.06	0.0426554
202633_at	11073	0.09	0.04	2.06	0.0427676
207092_at	3952	0.39	0.19	2.06	0.0427727
223097_at	54936	0.11	0.05	2.06	0.0428108
65591_at	57599	-0.08	0.04	-2.06	0.0428642
208313_s_at	7536	0.10	0.05	2.06	0.0428726
218250_s_at	29883	-0.15	0.07	-2.06	0.0428803
231403_at	7204	-0.24	0.11	-2.06	0.0429713
227704_at	83636	0.22	0.11	2.06	0.0429896
48531_at	79155	0.10	0.05	2.06	0.0430113
212770_at	7090	0.12	0.06	2.06	0.0430438
35820_at	2760	0.18	0.09	2.06	0.0431025
236699_at	10150	-0.19	0.09	-2.06	0.0431473
201517_at	22916	0.08	0.04	2.06	0.0432173
222459_at	79647	0.16	0.08	2.06	0.0432596
218934_s_at	27129	0.34	0.17	2.06	0.0432646
207624_s_at	6103	0.23	0.11	2.06	0.0432743
210266_s_at	51592	-0.11	0.05	-2.06	0.0433407
212259_s_at	57326	0.13	0.06	2.06	0.0433866
32836_at	10554	0.13	0.06	2.06	0.0434305
203522_at	9973	0.09	0.04	2.06	0.0434527
233557_s_at	22879	0.10	0.05	2.06	0.04349
202223_at	3703	-0.12	0.06	-2.06	0.0435089
219289_at	55027	0.14	0.07	2.06	0.0435134
211698_at	23741	-0.16	0.08	-2.06	0.0435341
227906_s_at	9489	-0.07	0.03	-2.06	0.0435621
225247_at	91304	0.11	0.05	2.06	0.0436051
43511_s_at	NA	0.12	0.06	2.05	0.0436401
40446_at	5252	0.09	0.04	2.05	0.0436458
212840_at	26043	-0.17	0.08	-2.05	0.0436596
200618_at	3927	0.09	0.05	2.05	0.0437089
206200_s_at	311	0.09	0.04	2.05	0.0437604
241472_at	1657	-0.26	0.13	-2.05	0.0437622
208629_s_at	3030	-0.14	0.07	-2.05	0.0437801
242974_at	961	-0.13	0.07	-2.05	0.0438124
90265_at	11033	0.15	0.07	2.05	0.0438606
202032_s_at	4122	0.14	0.07	2.05	0.0438685
225303_at	55243	0.12	0.06	2.05	0.0438717
219952_s_at	57192	0.12	0.06	2.05	0.0438776
214177_s_at	57326	0.10	0.05	2.05	0.0438944
212880_at	23335	0.12	0.06	2.05	0.0439065
227623_at	781	0.22	0.11	2.05	0.0439072

202054_s_at	224	0.11	0.05	2.05	0.043975
221855_at	NA	-0.07	0.04	-2.05	0.044038
1552501_a_at	151306	0.18	0.09	2.05	0.0440916
224673_at	114823	0.21	0.10	2.05	0.044099
212244_at	145781	-0.17	0.08	-2.05	0.0441026
218124_at	54884	0.23	0.11	2.05	0.0441059
37950_at	5550	0.09	0.04	2.05	0.0441238
59375_at	80022	0.19	0.09	2.05	0.0442241
206918_s_at	8904	0.11	0.06	2.05	0.0442371
203956_at	22880	0.10	0.05	2.05	0.0442845
225332_at	11339	-0.15	0.07	-2.05	0.0443313
232253_at	441108	-0.10	0.05	-2.05	0.0443864
227278_at	6884	0.16	0.08	2.05	0.0444111
206138_s_at	5298	0.07	0.03	2.05	0.0444178
214454_at	9509	0.16	0.08	2.05	0.0444682
236953_s_at	387921	-0.24	0.12	-2.05	0.0444699
224366_s_at	85021	0.10	0.05	2.05	0.0444702
225212_at	114789	0.12	0.06	2.05	0.0444815
230282_at	10099	0.21	0.10	2.05	0.0444835
210094_s_at	56288	-0.11	0.06	-2.05	0.0444896
207551_s_at	10943	0.06	0.03	2.05	0.0444936
228731_at	NA	-0.37	0.18	-2.05	0.0445528
206158_s_at	7555	-0.12	0.06	-2.05	0.044584
209410_s_at	2887	0.10	0.05	2.05	0.0446095
225133_at	51274	-0.14	0.07	-2.04	0.0446405
210106_at	5959	0.26	0.13	2.04	0.0446687
203501_at	10404	-0.14	0.07	-2.04	0.0447124
208830_s_at	6830	0.14	0.07	2.04	0.0447504
203602_s_at	7709	0.11	0.05	2.04	0.0447576
241425_at	9818	-0.23	0.11	-2.04	0.0447628
201644_at	7264	0.09	0.04	2.04	0.044781
200752_s_at	823	0.09	0.05	2.04	0.0447879
205516_x_at	25792	0.12	0.06	2.04	0.0447923
231205_at	50807	-0.21	0.10	-2.04	0.0448243
211148_s_at	285	0.20	0.10	2.04	0.0448252
209608_s_at	39	0.13	0.06	2.04	0.0448474
216836_s_at	2064	0.13	0.06	2.04	0.0448675
226675_s_at	378938	-0.23	0.11	-2.04	0.0448865
228189_at	9530	-0.22	0.11	-2.04	0.0448925
225330_at	145815	-0.19	0.09	-2.04	0.0449123
206132_at	4163	-0.12	0.06	-2.04	0.0449142
AFFX-M27830_5_at	54345	-0.20	0.10	-2.04	0.0449406
242304_at	84305	0.10	0.05	2.04	0.0450254
203370_s_at	9260	0.20	0.10	2.04	0.0450502
229642_at	8874	-0.17	0.08	-2.04	0.0450751
232262_at	9487	0.18	0.09	2.04	0.045083
224933_s_at	221037	-0.15	0.07	-2.04	0.0450968
204976_s_at	9949	-0.21	0.10	-2.04	0.0451011
231766_s_at	1303	0.21	0.10	2.04	0.0451314
204937_s_at	10782	0.10	0.05	2.04	0.0451581
201720_s_at	7805	0.25	0.12	2.04	0.0451615
207559_s_at	9203	0.10	0.05	2.04	0.0451835

207836_s_at	11030	0.16	0.08	2.04	0.0452584
213486_at	26958	0.36	0.17	2.04	0.0452585
228986_at	114882	-0.19	0.09	-2.04	0.0452616
218028_at	64834	0.10	0.05	2.04	0.0452978
223050_s_at	54461	0.11	0.05	2.04	0.0453163
219550_at	64221	0.17	0.08	2.04	0.0453447
226376_at	85451	0.10	0.05	2.04	0.0454027
218945_at	79091	0.09	0.05	2.04	0.0454489
216484_x_at	3068	0.07	0.04	2.04	0.045464
203645_s_at	9332	0.30	0.15	2.04	0.0454743
31837_at	91289	0.10	0.05	2.04	0.0454848
223445_at	84062	0.08	0.04	2.04	0.0455036
201658_at	400	-0.12	0.06	-2.04	0.0455127
223196_s_at	83667	0.13	0.06	2.04	0.0455766
232017_at	9414	-0.18	0.09	-2.04	0.0455776
212788_x_at	2512	0.10	0.05	2.04	0.0455891
225380_at	91461	0.13	0.06	2.04	0.0455999
225177_at	80223	0.10	0.05	2.04	0.0456311
222497_x_at	51068	-0.14	0.07	-2.03	0.0456808
212400_at	399665	0.12	0.06	2.03	0.0456877
219413_at	79777	0.10	0.05	2.03	0.0456959
213592_at	187	-0.21	0.11	-2.03	0.0457406
225286_at	414	0.14	0.07	2.03	0.0457439
221135_s_at	28990	-0.10	0.05	-2.03	0.0458651
201385_at	1665	-0.07	0.04	-2.03	0.0459086
212926_at	23137	-0.16	0.08	-2.03	0.0459169
203045_at	4814	0.15	0.07	2.03	0.0459793
201367_s_at	678	0.19	0.10	2.03	0.0460425
230343_at	NA	0.20	0.10	2.03	0.0462169
233813_at	26051	-0.18	0.09	-2.03	0.046229
204347_at	387851	-0.25	0.12	-2.03	0.0463224
201260_s_at	6856	-0.13	0.07	-2.03	0.0463825
219186_at	51341	0.15	0.07	2.03	0.046389
219097_x_at	79086	-0.12	0.06	-2.03	0.0464697
219826_at	79744	0.11	0.05	2.03	0.0465025
201443_s_at	10159	-0.13	0.07	-2.03	0.0465068
222468_at	79932	0.12	0.06	2.03	0.0465782
40560_at	6909	0.19	0.09	2.03	0.0466259
223253_at	54749	0.20	0.10	2.02	0.0467177
226065_at	144165	-0.14	0.07	-2.02	0.046742
212231_at	23014	-0.11	0.05	-2.02	0.0467617
225371_at	2733	0.08	0.04	2.02	0.0467876
227025_at	51535	0.12	0.06	2.02	0.0468403
230791_at	4781	-0.30	0.15	-2.02	0.0468573
202379_s_at	4820	-0.16	0.08	-2.02	0.046862
201642_at	3460	0.09	0.05	2.02	0.0468643
222619_at	23528	-0.13	0.06	-2.02	0.0469004
210983_s_at	4176	0.11	0.05	2.02	0.0469638
219145_at	22859	0.12	0.06	2.02	0.0469852
218533_s_at	54963	0.13	0.07	2.02	0.0470433
225832_s_at	221955	0.13	0.06	2.02	0.0471059
204308_s_at	9895	0.11	0.05	2.02	0.0471307

214843_s_at	23032	-0.14	0.07	-2.02	0.0471329
227779_at	NA	-0.26	0.13	-2.02	0.0471393
210973_s_at	2260	0.14	0.07	2.02	0.0471589
202079_s_at	22906	0.12	0.06	2.02	0.0472263
211505_s_at	6780	0.10	0.05	2.02	0.0472473
230381_at	440712	0.18	0.09	2.02	0.0472626
228144_at	91975	0.14	0.07	2.02	0.047292
222221_x_at	10938	0.14	0.07	2.02	0.0473506
219593_at	51296	0.14	0.07	2.02	0.0473733
222413_s_at	58508	-0.17	0.08	-2.02	0.0474185
218010_x_at	79144	0.18	0.09	2.02	0.04748
225317_at	84320	0.10	0.05	2.02	0.0474874
205740_s_at	79171	0.11	0.06	2.02	0.0475607
200947_s_at	2746	-0.09	0.04	-2.02	0.0476001
227197_at	26084	-0.27	0.13	-2.02	0.0476541
202624_s_at	23523	0.13	0.07	2.02	0.04769
228158_at	1818	-0.17	0.08	-2.02	0.047694
212122_at	284988	0.21	0.10	2.02	0.047726
217221_x_at	8241	0.13	0.06	2.02	0.047734
228555_at	817	0.13	0.07	2.01	0.0477829
223235_s_at	64094	-0.29	0.14	-2.01	0.0478058
212192_at	115207	-0.15	0.07	-2.01	0.0478272
218122_s_at	59343	-0.09	0.04	-2.01	0.0478393
221899_at	10443	-0.24	0.12	-2.01	0.0478519
225061_at	55466	0.13	0.06	2.01	0.047926
203241_at	7405	0.12	0.06	2.01	0.0479357
235405_at	2941	-0.15	0.07	-2.01	0.0479443
223922_x_at	64231	0.23	0.11	2.01	0.0479492
226895_at	4782	0.14	0.07	2.01	0.0479547
224923_at	57217	0.11	0.06	2.01	0.0479696
213088_s_at	23234	0.10	0.05	2.01	0.0480161
218429_s_at	55337	0.14	0.07	2.01	0.048018
219770_at	79712	0.11	0.06	2.01	0.0480313
222434_at	55740	-0.15	0.08	-2.01	0.0480931
212313_at	91782	0.08	0.04	2.01	0.0481412
222718_at	58986	0.12	0.06	2.01	0.048168
228131_at	10849	0.14	0.07	2.01	0.0481737
204977_at	1662	0.09	0.05	2.01	0.0482378
207277_at	30835	0.26	0.13	2.01	0.0482454
225248_at	56928	0.07	0.04	2.01	0.0483592
203127_s_at	9517	0.09	0.04	2.01	0.0483673
202228_s_at	27020	-0.08	0.04	-2.01	0.0483755
228462_at	153572	0.23	0.12	2.01	0.0483947
224964_s_at	54331	0.19	0.09	2.01	0.048407
214658_at	51014	-0.24	0.12	-2.01	0.0484149
211270_x_at	5725	0.09	0.05	2.01	0.048417
209221_s_at	9885	0.07	0.04	2.01	0.0484666
218873_at	54856	0.10	0.05	2.01	0.048467
200743_s_at	1200	0.15	0.07	2.01	0.0485082
218973_at	79631	0.09	0.05	2.01	0.0485445
202960_s_at	4594	-0.11	0.06	-2.01	0.0485619
227791_at	285195	0.13	0.06	2.01	0.0485842

201763_s_at	1616	0.09	0.04	2.01	0.0486573
228264_at	84680	0.15	0.07	2.01	0.0487026
200968_s_at	5479	-0.17	0.09	-2.01	0.0487513
225931_s_at	57674	0.15	0.08	2.01	0.0487715
213694_at	54665	-0.19	0.09	-2.01	0.0487821
219771_at	54885	-0.14	0.07	-2.01	0.0488144
225620_at	11021	0.09	0.04	2.01	0.0488262
218337_at	64760	0.10	0.05	2.00	0.0488368
201692_at	10280	0.09	0.04	2.00	0.0488398
218530_at	29109	0.13	0.07	2.00	0.0488838
225475_at	57708	-0.16	0.08	-2.00	0.0489128
232797_at	3685	-0.26	0.13	-2.00	0.0489138
209082_s_at	80781	0.21	0.10	2.00	0.0489349
213581_at	5134	0.12	0.06	2.00	0.0489931
241114_s_at	NA	0.17	0.09	2.00	0.0489937
209540_at	3479	-0.25	0.13	-2.00	0.0490144
215513_at	57061	-0.26	0.13	-2.00	0.0490744
205641_s_at	8717	0.07	0.03	2.00	0.0490764
233870_at	89796	0.12	0.06	2.00	0.0490994
230653_at	NA	-0.27	0.13	-2.00	0.0491027
230550_at	64231	0.27	0.13	2.00	0.0491222
201814_at	9779	0.07	0.04	2.00	0.0491828
212219_at	23198	0.11	0.06	2.00	0.0492495
202702_at	7726	0.10	0.05	2.00	0.0492691
235037_at	90407	-0.08	0.04	-2.00	0.049311
232865_at	27125	-0.16	0.08	-2.00	0.0493166
225961_at	57542	-0.16	0.08	-2.00	0.0494105
204049_s_at	9749	-0.16	0.08	-2.00	0.0494568
208783_s_at	4179	0.07	0.04	2.00	0.0494838
214661_s_at	8602	0.10	0.05	2.00	0.0495177
227142_at	57449	0.19	0.09	2.00	0.0495716
235451_at	4090	-0.20	0.10	-2.00	0.0495907
227102_at	23087	0.14	0.07	2.00	0.0496551
226017_at	112616	0.12	0.06	2.00	0.0497368
200994_at	10527	-0.11	0.05	-2.00	0.0497411
208895_s_at	8886	-0.14	0.07	-2.00	0.0498028
208794_s_at	6597	0.14	0.07	2.00	0.0498128
228927_at	84307	-0.13	0.06	-2.00	0.0498658
225508_at	57614	0.11	0.05	2.00	0.0498791
201303_at	9775	-0.09	0.05	-2.00	0.0499347
217854_s_at	5434	0.13	0.07	2.00	0.0499352
230172_at	122509	0.10	0.05	1.99	0.0499697
226164_x_at	57494	0.14	0.07	1.99	0.0499971
