# Supplementary material for: A Systems Genetics Approach Implicates USF1, FADS3, and Other Causal Candidate Genes for Familial Combined Hyperlipidemia
Source: PLoS Genet. 2009 Sep 11;5(9):e1000642. doi: 10.1371/journal.pgen.1000642 (PMC2730565; doi:10.1371/journal.pgen.1000642)
Supplement: Table S5 — Functionally enriched annotation terms for the set of genes comprising each co-expression module (Benjamini-Hochberg corrected p-value≤0.05). (0.11 MB PDF) [file pgen.1000642.s007.pdf]

Table S5. Functionally enriched annotation terms for the set of genes comprising each co-expression module (Benjamini-Hochberg corrected p-value≤ 0.05).

Module	Category	Term	Count	PValue	Fold Enrichment	Bonferroni	Benjamini	FDR
Turquoise	GOTERM_CC_ALL	GO:0005622~intracellular	874	1.5E-07	1.07	1.2E-04	1.2E-04	2.3E-04
Turquoise	GOTERM_BP_ALL	GO:0046907~intracellular transport	113	2.7E-08	1.65	1.2E-04	1.2E-04	5.0E-05
Turquoise	SP_PIR_KEYWORDS	Ubl conjugation pathway	75	1.9E-07	1.81	1.7E-04	1.7E-04	2.9E-04
Turquoise	GOTERM_CC_ALL	GO:0044424~intracellular part	841	4.6E-07	1.08	3.5E-04	1.8E-04	7.0E-04
Turquoise	GOTERM_BP_ALL	GO:0051649~establishment of cellular localization	122	1.1E-07	1.57	4.8E-04	2.4E-04	2.1E-04
Turquoise	GOTERM_BP_ALL	GO:0051641~cellular localization	123	2.1E-07	1.55	8.9E-04	3.0E-04	3.9E-04
Turquoise	GOTERM_BP_ALL	GO:0006512~ubiquitin cycle	85	4.7E-07	1.70	2.0E-03	5.1E-04	8.8E-04
Turquoise	GOTERM_BP_ALL	GO:0033036~macromolecule localization	126	8.1E-07	1.51	3.5E-03	7.0E-04	1.5E-03
Turquoise	GOTERM_BP_ALL	GO:0043687~post-translational protein modification	176	2.1E-06	1.37	9.2E-03	1.5E-03	4.0E-03
Turquoise	GOTERM_BP_ALL	GO:0045184~establishment of protein localization	114	3.2E-06	1.50	1.4E-02	1.6E-03	6.1E-03
Turquoise	GOTERM_BP_ALL	GO:0043412~biopolymer modification	206	4.4E-06	1.32	1.9E-02	1.7E-03	8.2E-03
Turquoise	GOTERM_BP_ALL	GO:0008104~protein localization	118	3.2E-06	1.49	1.4E-02	1.8E-03	6.1E-03
Turquoise	GOTERM_BP_ALL	GO:0015031~protein transport	110	4.3E-06	1.51	1.9E-02	1.9E-03	8.1E-03
Turquoise	GOTERM_BP_ALL	GO:0006464~protein modification process	200	3.0E-06	1.33	1.3E-02	1.9E-03	5.7E-03
Turquoise	GOTERM_BP_ALL	GO:0016043~cellular component organization and biogenesis	285	5.4E-06	1.25	2.3E-02	2.0E-03	1.0E-02
Turquoise	GOTERM_BP_ALL	GO:0044260~cellular macromolecule metabolic process	330	6.5E-06	1.22	2.8E-02	2.2E-03	1.2E-02
Turquoise	GOTERM_BP_ALL	GO:0044267~cellular protein metabolic process	325	7.7E-06	1.22	3.3E-02	2.4E-03	1.4E-02
Turquoise	GOTERM_BP_ALL	GO:0019538~protein metabolic process	343	1.3E-05	1.20	5.3E-02	3.6E-03	2.3E-02
Turquoise	SP_PIR_KEYWORDS	protein transport	74	1.9E-05	1.62	1.7E-02	8.4E-03	2.9E-02
Turquoise	GOTERM_MF_ALL	GO:0005488~binding	921	9.2E-06	1.05	2.1E-02	2.1E-02	1.6E-02
Turquoise	GOTERM_BP_ALL	GO:0043283~biopolymer metabolic process	458	1.1E-04	1.13	3.7E-01	2.9E-02	2.0E-01
Turquoise	GOTERM_BP_ALL	GO:0006886~intracellular protein transport	68	1.6E-04	1.55	5.0E-01	4.0E-02	3.0E-01
Blue	GOTERM_BP_ALL	GO:0032787~monocarboxylic acid metabolic process	56	7.4E-18	3.47	3.2E-14	1.6E-14	1.4E-14
Blue	GOTERM_BP_ALL	GO:0019752~carboxylic acid metabolic process	93	1.5E-17	2.47	6.3E-14	2.1E-14	2.7E-14
Blue	GOTERM_BP_ALL	GO:0006082~organic acid metabolic process	94	6.9E-18	2.48	3.0E-14	3.0E-14	1.3E-14
Blue	GOTERM_BP_ALL	GO:0006629~lipid metabolic process	104	6.5E-16	2.22	2.9E-12	7.2E-13	1.2E-12
Blue	SP_PIR_KEYWORDS	oxidoreductase	82	7.2E-13	2.25	6.5E-10	6.5E-10	1.1E-09
Blue	GOTERM_BP_ALL	GO:0044255~cellular lipid metabolic process	86	1.0E-12	2.18	4.5E-09	9.0E-10	1.9E-09
Blue	GOTERM_BP_ALL	GO:0006631~fatty acid metabolic process	41	1.4E-12	3.35	6.3E-09	1.0E-09	2.7E-09
Blue	GOTERM_BP_ALL	GO:0006091~generation of precursor metabolites and energy	84	2.1E-11	2.09	9.2E-08	1.3E-08	4.0E-08
Blue	KEGG_PATHWAY	hsa00071:Fatty acid metabolism	23	2.0E-10	4.21	3.9E-08	3.9E-08	2.4E-07
Blue	KEGG_PATHWAY	hsa00640:Propanoate metabolism	19	9.2E-10	4.68	1.8E-07	6.0E-08	1.1E-06
Blue	GOTERM_MF_ALL	GO:0016491~oxidoreductase activity	105	3.2E-11	1.90	7.2E-08	7.2E-08	5.5E-08
Blue	KEGG_PATHWAY	hsa00280:Valine, leucine and isoleucine degradation	23	9.2E-10	3.98	1.8E-07	9.1E-08	1.1E-06
Blue	KEGG_PATHWAY	hsa03320:PPAR signaling pathway	23	1.9E-09	3.88	3.7E-07	9.2E-08	2.3E-06
Blue	GOTERM_MF_ALL	GO:0048037~cofactor binding	43	1.2E-10	2.89	2.7E-07	1.4E-07	2.1E-07
Blue	GOTERM_CC_ALL	GO:0005737~cytoplasm	534	2.0E-09	1.18	1.5E-06	1.5E-06	3.0E-06
Blue	SP_PIR_KEYWORDS	Fatty acid metabolism	19	3.6E-09	4.83	3.3E-06	1.6E-06	5.6E-06
Blue	GOTERM_CC_ALL	GO:0005739~mitochondrion	129	1.4E-08	1.61	1.1E-05	5.3E-06	2.1E-05
Blue	GOTERM_MF_ALL	GO:0050662~coenzyme binding	34	9.0E-09	2.94	2.0E-05	6.8E-06	1.6E-05
Blue	GOTERM_BP_ALL	GO:0051186~cofactor metabolic process	44	1.5E-08	2.47	6.6E-05	8.2E-06	2.8E-05
Blue	KEGG_PATHWAY	hsa00010:Glycolysis / Gluconeogenesis	19	3.8E-07	3.58	7.6E-05	1.5E-05	4.8E-04
Blue	SP_PIR_KEYWORDS	transit peptide	72	5.8E-08	1.90	5.3E-05	1.8E-05	9.1E-05
Blue	GOTERM_BP_ALL	GO:0006066~alcohol metabolic process	45	4.9E-08	2.35	2.1E-04	2.4E-05	9.2E-05
Blue	KEGG_PATHWAY	hsa00650:Butanoate metabolism	17	8.4E-07	3.76	1.7E-04	2.8E-05	1.0E-03
Blue	SP_PIR_KEYWORDS	Mitochondrion	102	1.5E-07	1.66	1.3E-04	3.3E-05	2.3E-04
Blue	SP_PIR_KEYWORDS	lipid metabolism	24	1.9E-07	3.30	1.7E-04	3.4E-05	2.9E-04
Blue	GOTERM_BP_ALL	GO:0006732~coenzyme metabolic process	36	1.7E-07	2.55	7.3E-04	7.3E-05	3.2E-04
Blue	KEGG_PATHWAY	hsa00620:Pyruvate metabolism	17	2.7E-06	3.51	5.4E-04	7.7E-05	3.4E-03
Blue	COG_ONTOLOGY	Lipid metabolism	21	1.2E-06	3.22	8.2E-05	8.2E-05	1.2E-03
Blue	GOTERM_CC_ALL	GO:0044444~cytoplasmic part	338	4.7E-07	1.24	3.6E-04	1.2E-04	7.1E-04
Blue	SP_PIR_KEYWORDS	peroxisome	22	1.0E-06	3.24	9.0E-04	1.5E-04	1.6E-03
Blue	GOTERM_BP_ALL	GO:0006635~fatty acid beta-oxidation	12	3.8E-07	5.95	1.7E-03	1.5E-04	7.2E-04
Blue	KEGG_PATHWAY	hsa01040:Polyunsaturated fatty acid biosynthesis	10	7.0E-06	5.34	1.4E-03	1.7E-04	8.7E-03
Blue	SP_PIR_KEYWORDS	multifunctional enzyme	18	1.6E-06	3.68	1.4E-03	2.0E-04	2.5E-03
Blue	KEGG_PATHWAY	hsa00020:Citrate cycle (TCA cycle)	14	1.2E-05	3.74	2.5E-03	2.7E-04	1.6E-02
Blue	GOTERM_CC_ALL	GO:0042579~microbody	23	1.6E-06	3.06	1.2E-03	3.1E-04	2.5E-03

Blue	GOTERM_CC_ALL	GO:0005777~peroxisome	23	1.6E-06	3.06	1.2E-03	3.1E-04	2.5E-03
Blue	SP_PIR_KEYWORDS	acetylation	85	3.1E-06	1.64	2.8E-03	3.5E-04	4.9E-03
Blue	GOTERM_BP_ALL	GO:0006084~acetyl-CoA metabolic process	14	1.1E-06	4.72	4.8E-03	4.0E-04	2.1E-03
Blue	GOTERM_BP_ALL	GO:0009109~coenzyme catabolic process	13	1.5E-06	4.98	6.4E-03	5.0E-04	2.8E-03
Blue	UP_SEQ_FEATURE	transit peptide:Mitochondrion	57	5.0E-08	2.09	5.3E-04	5.3E-04	1.0E-04
Blue	SP_PIR_KEYWORDS	disease mutation	123	6.2E-06	1.46	5.6E-03	6.2E-04	9.7E-03
Blue	KEGG_PATHWAY	hsa00720:Reductive carboxylate cycle (CO2 fixation)	9	3.4E-05	5.24	6.8E-03	6.8E-04	4.3E-02
Blue	GOTERM_BP_ALL	GO:0019395~fatty acid oxidation	13	2.8E-06	4.76	1.2E-02	8.6E-04	5.2E-03
Blue	GOTERM_BP_ALL	GO:0044262~cellular carbohydrate metabolic process	46	3.9E-06	2.02	1.7E-02	1.1E-03	7.3E-03
Blue	GOTERM_MF_ALL	GO:0003824~catalytic activity	420	2.0E-06	1.18	4.5E-03	1.1E-03	3.5E-03
Blue	GOTERM_BP_ALL	GO:0006100~tricarboxylic acid cycle intermediate metabolic process	11	4.6E-06	5.45	2.0E-02	1.3E-03	8.7E-03
Blue	SP_PIR_KEYWORDS	fad	21	1.4E-05	2.89	1.3E-02	1.3E-03	2.2E-02
Blue	SP_PIR_KEYWORDS	flavoprotein	21	1.8E-05	2.84	1.6E-02	1.5E-03	2.9E-02
Blue	GOTERM_MF_ALL	GO:0016903~oxidoreductase activity, acting on the aldehyde or oxo group of donors	12	3.6E-06	5.08	8.1E-03	1.6E-03	6.2E-03
Blue	GOTERM_MF_ALL	GO:0016616~oxidoreductase activity, acting on the CH-OH group of donors, NAD or NADP as acceptor	23	4.4E-06	2.90	9.9E-03	1.7E-03	7.6E-03
Blue	GOTERM_CC_ALL	GO:0044429~mitochondrial part	77	1.3E-05	1.62	1.0E-02	1.7E-03	2.0E-02
Blue	GOTERM_BP_ALL	GO:0006006~glucose metabolic process	22	7.7E-06	2.90	3.3E-02	1.8E-03	1.4E-02
Blue	GOTERM_BP_ALL	GO:0006099~tricarboxylic acid cycle	12	7.0E-06	4.81	3.0E-02	1.8E-03	1.3E-02
Blue	GOTERM_BP_ALL	GO:0046356~acetyl-CoA catabolic process	12	7.0E-06	4.81	3.0E-02	1.8E-03	1.3E-02
Blue	GOTERM_BP_ALL	GO:0051187~cofactor catabolic process	13	8.5E-06	4.38	3.6E-02	1.8E-03	1.6E-02
Blue	GOTERM_BP_ALL	GO:0008610~lipid biosynthetic process	39	1.2E-05	2.08	5.0E-02	2.4E-03	2.2E-02
Blue	KEGG_PATHWAY	hsa00380:Tryptophan metabolism	15	1.8E-04	2.91	3.6E-02	3.0E-03	2.3E-01
Blue	KEGG_PATHWAY	hsa04910:Insulin signaling pathway	29	1.8E-04	2.02	3.6E-02	3.3E-03	2.3E-01
Blue	GOTERM_BP_ALL	GO:0006725~aromatic compound metabolic process	20	2.1E-05	2.90	8.9E-02	4.0E-03	4.0E-02
Blue	GOTERM_BP_ALL	GO:0045333~cellular respiration	15	2.0E-05	3.61	8.5E-02	4.0E-03	3.8E-02
Blue	SP_PIR_KEYWORDS	hydro-lyase	7	6.4E-05	7.34	5.6E-02	4.8E-03	9.9E-02
Blue	SP_PIR_KEYWORDS	NAD	31	7.0E-05	2.13	6.1E-02	4.8E-03	1.1E-01
Blue	GOTERM_BP_ALL	GO:0005975~carbohydrate metabolic process	55	2.8E-05	1.76	1.2E-01	5.1E-03	5.3E-02
Blue	GOTERM_MF_ALL	GO:0016614~oxidoreductase activity, acting on CH-OH group of donors	23	1.6E-05	2.70	3.6E-02	5.2E-03	2.8E-02
Blue	SP_PIR_KEYWORDS	Tricarboxylic acid cycle	10	8.3E-05	4.66	7.2E-02	5.3E-03	1.3E-01
Blue	GOTERM_MF_ALL	GO:0016829~lyase activity	25	2.4E-05	2.52	5.2E-02	6.7E-03	4.1E-02
Blue	GOTERM_CC_ALL	GO:0005759~mitochondrial matrix	34	6.3E-05	2.05	4.7E-02	6.9E-03	9.6E-02
Blue	GOTERM_CC_ALL	GO:0031980~mitochondrial lumen	34	6.3E-05	2.05	4.7E-02	6.9E-03	9.6E-02
Blue	SP_PIR_KEYWORDS	Biotin	6	1.3E-04	8.38	1.1E-01	7.7E-03	2.0E-01
Blue	SP_PIR_KEYWORDS	glycolysis	10	1.4E-04	4.41	1.2E-01	7.9E-03	2.2E-01
Blue	GOTERM_BP_ALL	GO:0006096~glycolysis	13	5.4E-05	3.78	2.1E-01	9.3E-03	1.0E-01
Blue	GOTERM_BP_ALL	GO:0019318~hexose metabolic process	25	5.8E-05	2.39	2.2E-01	9.6E-03	1.1E-01
Blue	GOTERM_BP_ALL	GO:0006007~glucose catabolic process	15	6.1E-05	3.32	2.3E-01	9.7E-03	1.1E-01
Blue	GOTERM_BP_ALL	GO:0005996~monosaccharide metabolic process	25	7.1E-05	2.37	2.6E-01	1.1E-02	1.3E-01
Blue	GOTERM_BP_ALL	GO:0009056~catabolic process	76	7.5E-05	1.55	2.8E-01	1.1E-02	1.4E-01
Blue	GOTERM_BP_ALL	GO:0006767~water-soluble vitamin metabolic process	13	8.0E-05	3.65	2.9E-01	1.1E-02	1.5E-01
Blue	GOTERM_BP_ALL	GO:0000003~reproduction	43	8.0E-05	1.85	2.9E-01	1.1E-02	1.5E-01
Blue	GOTERM_CC_ALL	GO:0016020~membrane	407	1.4E-04	1.14	9.9E-02	1.2E-02	2.1E-01
Blue	SP_PIR_KEYWORDS	lipid synthesis	18	2.3E-04	2.65	1.9E-01	1.2E-02	3.6E-01
Blue	GOTERM_BP_ALL	GO:0046164~alcohol catabolic process	16	9.4E-05	3.06	3.3E-01	1.3E-02	1.8E-01
Blue	GOTERM_MF_ALL	GO:0016836~hydro-lyase activity	12	5.4E-05	4.06	1.2E-01	1.4E-02	9.5E-02
Blue	SP_PIR_KEYWORDS	carbon-oxygen lyase	7	3.9E-04	5.87	2.9E-01	1.8E-02	6.0E-01
Blue	SP_PIR_KEYWORDS	nadp	22	3.9E-04	2.28	2.9E-01	1.9E-02	6.0E-01
Blue	GOTERM_BP_ALL	GO:0019320~hexose catabolic process	15	1.6E-04	3.08	4.9E-01	2.0E-02	2.9E-01
Blue	GOTERM_BP_ALL	GO:0044248~cellular catabolic process	66	1.8E-04	1.57	5.4E-01	2.3E-02	3.3E-01
Blue	GOTERM_MF_ALL	GO:0016620~oxidoreductase activity, acting on the aldehyde or oxo group of donors, NAD or NADP as acceptor	9	1.1E-04	5.08	2.2E-01	2.5E-02	1.9E-01
Blue	GOTERM_MF_ALL	GO:0004300~enoyl-CoA hydratase activity	6	1.2E-04	8.46	2.4E-01	2.5E-02	2.1E-01
Blue	GOTERM_BP_ALL	GO:0046365~monosaccharide catabolic process	15	2.1E-04	3.01	6.0E-01	2.6E-02	3.9E-01
Blue	GOTERM_BP_ALL	GO:0009060~aerobic respiration	12	2.8E-04	3.49	7.0E-01	3.3E-02	5.2E-01
Blue	GOTERM_BP_ALL	GO:0006090~pyruvate metabolic process	8	2.9E-04	5.18	7.2E-01	3.3E-02	5.4E-01
Blue	GOTERM_BP_ALL	GO:0009058~biosynthetic process	130	3.1E-04	1.33	7.4E-01	3.5E-02	5.8E-01
Blue	GOTERM_BP_ALL	GO:0006118~electron transport	46	3.3E-04	1.70	7.7E-01	3.7E-02	6.2E-01
Blue	GOTERM_BP_ALL	GO:0006000~fructose metabolic process	6	4.0E-04	7.22	8.2E-01	4.2E-02	7.4E-01
Blue	SP_PIR_KEYWORDS	acyltransferase	21	1.0E-03	2.17	6.1E-01	4.5E-02	1.6E+00

Blue	KEGG_PATHWAY	hsa00030:Pentose phosphate pathway	9	3.3E-03	3.20	4.8E-01	4.9E-02	4.1E+00
Blue	GOTERM_MF_ALL	GO:0016835~carbon-oxygen lyase activity	12	2.7E-04	3.50	4.6E-01	5.0E-02	4.7E-01
Brown	GOTERM_CC_ALL	GO:0005634~nucleus	445	1.1E-21	1.41	8.3E-19	8.3E-19	1.6E-18
Brown	SP_PIR_KEYWORDS	nucleus	377	3.0E-15	1.39	2.7E-12	2.7E-12	4.7E-12
Brown	GOTERM_BP_ALL	GO:0016070~RNA metabolic process	291	6.4E-16	1.49	2.9E-12	2.9E-12	1.2E-12
Brown	GOTERM_MF_ALL	GO:0003676~nucleic acid binding	313	2.1E-15	1.46	4.8E-12	4.8E-12	3.7E-12
Brown	GOTERM_BP_ALL	GO:0043283~biopolymer metabolic process	448	7.2E-15	1.30	3.1E-11	1.6E-11	1.4E-11
Brown	GOTERM_BP_ALL	GO:0006139~nucleobase, nucleoside, nucleotide and nucleic acid metabolic process	350	3.8E-14	1.38	1.7E-10	5.5E-11	7.1E-11
Brown	GOTERM_BP_ALL	GO:0016071~mRNA metabolic process	72	2.7E-13	2.45	1.2E-09	2.9E-10	5.0E-10
Brown	GOTERM_BP_ALL	GO:0006397~mRNA processing	64	1.1E-12	2.54	4.8E-09	9.5E-10	2.1E-09
Brown	GOTERM_BP_ALL	GO:0008380~RNA splicing	60	3.4E-12	2.57	1.5E-08	2.4E-09	6.3E-09
Brown	GOTERM_BP_ALL	GO:0010467~gene expression	316	3.4E-11	1.35	1.5E-07	2.1E-08	6.3E-08
Brown	GOTERM_MF_ALL	GO:0003677~DNA binding	205	3.2E-11	1.52	7.3E-08	3.6E-08	5.6E-08
Brown	GOTERM_BP_ALL	GO:0006396~RNA processing	85	6.8E-11	2.04	3.0E-07	3.7E-08	1.3E-07
Brown	SP_PIR_KEYWORDS	phosphoprotein	466	6.9E-10	1.23	6.2E-07	3.1E-07	1.1E-06
Brown	GOTERM_BP_ALL	GO:0019219~regulation of nucleobase, nucleoside, nucleotide and nucleic acid metabolic process	224	1.3E-09	1.42	5.5E-06	6.1E-07	2.4E-06
Brown	GOTERM_BP_ALL	GO:0006350~transcription	227	2.1E-09	1.41	9.0E-06	9.0E-07	3.9E-06
Brown	SP_PIR_KEYWORDS	mrna processing	53	4.4E-09	2.32	4.0E-06	1.3E-06	6.9E-06
Brown	GOTERM_BP_ALL	GO:0045449~regulation of transcription	217	3.5E-09	1.42	1.5E-05	1.4E-06	6.6E-06
Brown	GOTERM_BP_ALL	GO:0006355~regulation of transcription, DNA-dependent	204	5.8E-09	1.43	2.5E-05	2.0E-06	1.1E-05
Brown	GOTERM_BP_ALL	GO:0010468~regulation of gene expression	229	5.6E-09	1.39	2.4E-05	2.0E-06	1.1E-05
Brown	UP_SEQ_FEATURE	zinc finger region:C2H2-type 6	37	2.5E-10	3.12	2.7E-06	2.7E-06	5.2E-07
Brown	SMART	SM00349:KRAB	35	7.9E-09	2.83	4.3E-06	4.3E-06	1.1E-05
Brown	GOTERM_BP_ALL	GO:0006351~transcription, DNA-dependent	208	1.4E-08	1.41	6.1E-05	4.3E-06	2.6E-05
Brown	GOTERM_BP_ALL	GO:0032774~RNA biosynthetic process	208	1.5E-08	1.41	6.5E-05	4.3E-06	2.8E-05
Brown	INTERPRO	IPR001909:KRAB box	34	1.1E-09	3.15	5.0E-06	5.0E-06	2.1E-06
Brown	GOTERM_BP_ALL	GO:0031323~regulation of cellular metabolic process	235	2.6E-08	1.36	1.1E-04	7.1E-06	4.9E-05
Brown	SP_PIR_KEYWORDS	alternative splicing	457	1.7E-07	1.19	1.5E-04	2.5E-05	2.6E-04
Brown	SP_PIR_KEYWORDS	dna-binding	146	1.2E-07	1.50	1.1E-04	2.6E-05	1.8E-04
Brown	SP_PIR_KEYWORDS	mrna splicing	44	1.5E-07	2.30	1.4E-04	2.7E-05	2.4E-04
Brown	UP_SEQ_FEATURE	zinc finger region:C2H2-type 7	33	5.5E-09	3.05	5.9E-05	3.0E-05	1.1E-05
Brown	UP_SEQ_FEATURE	zinc finger region:C2H2-type 5	39	1.4E-08	2.65	1.5E-04	3.8E-05	2.9E-05
Brown	UP_SEQ_FEATURE	zinc finger region:C2H2-type 8	30	1.1E-08	3.17	1.2E-04	4.0E-05	2.3E-05
Brown	PIR_SUPERFAMILY	PIRSF005559:zinc finger protein ZFP-36	15	2.0E-08	5.92	4.5E-05	4.5E-05	3.4E-05
Brown	GOTERM_BP_ALL	GO:0019222~regulation of metabolic process	239	2.2E-07	1.33	9.4E-04	5.5E-05	4.1E-04
Brown	INTERPRO	IPR007086:Zinc finger, C2H2-subtype	33	2.8E-08	2.87	1.3E-04	6.4E-05	5.4E-05
Brown	SP_PIR_KEYWORDS	rna-binding	74	8.1E-07	1.77	7.3E-04	1.0E-04	1.3E-03
Brown	GOTERM_BP_ALL	GO:0043170~macromolecule metabolic process	517	6.2E-07	1.14	2.7E-03	1.5E-04	1.2E-03
Brown	UP_SEQ_FEATURE	zinc finger region:C2H2-type 9	27	1.1E-07	3.12	1.1E-03	2.3E-04	2.2E-04
Brown	GOTERM_CC_ALL	GO:0043226~organelle	620	1.9E-06	1.11	1.5E-03	3.7E-04	2.9E-03
Brown	INTERPRO	IPR007087:Zinc finger, C2H2-type	71	2.8E-07	1.84	1.2E-03	4.2E-04	5.2E-04
Brown	SP_PIR_KEYWORDS	zinc-finger	149	3.7E-06	1.42	3.4E-03	4.2E-04	5.8E-03
Brown	UP_SEQ_FEATURE	zinc finger region:C2H2-type 4	39	2.4E-07	2.41	2.5E-03	4.2E-04	4.9E-04
Brown	GOTERM_CC_ALL	GO:0043229~intracellular organelle	620	1.7E-06	1.11	1.3E-03	4.3E-04	2.6E-03
Brown	GOTERM_CC_ALL	GO:0044428~nuclear part	135	1.1E-06	1.48	8.7E-04	4.4E-04	1.7E-03
Brown	GOTERM_CC_ALL	GO:0005815~microtubule organizing center	27	3.3E-06	2.66	2.5E-03	5.1E-04	5.1E-03
Brown	UP_SEQ_FEATURE	zinc finger region:C2H2-type 3	43	3.6E-07	2.26	3.8E-03	5.5E-04	7.3E-04
Brown	GOTERM_CC_ALL	GO:0043227~membrane-bound organelle	567	7.4E-06	1.12	5.6E-03	8.1E-04	1.1E-02
Brown	UP_SEQ_FEATURE	domain:KRAB	22	6.1E-07	3.33	6.6E-03	8.2E-04	1.3E-03
Brown	SP_PIR_KEYWORDS	Coiled coil	150	8.3E-06	1.39	7.5E-03	8.3E-04	1.3E-02
Brown	GOTERM_CC_ALL	GO:0043231~intracellular membrane-bound organelle	567	6.6E-06	1.12	5.0E-03	8.4E-04	1.0E-02
Brown	UP_SEQ_FEATURE	zinc finger region:C2H2-type 10	23	9.3E-07	3.16	1.0E-02	1.0E-03	1.9E-03
Brown	UP_SEQ_FEATURE	zinc finger region:C2H2-type 2	43	8.4E-07	2.20	9.0E-03	1.0E-03	1.7E-03
Brown	GOTERM_CC_ALL	GO:0005813~centrosome	25	1.5E-05	2.59	1.1E-02	1.4E-03	2.2E-02
Brown	UP_SEQ_FEATURE	splice variant	306	1.6E-06	1.23	1.7E-02	1.5E-03	3.2E-03
Brown	GOTERM_BP_ALL	GO:0050794~regulation of cellular process	336	7.7E-06	1.20	3.3E-02	1.8E-03	1.4E-02
Brown	GOTERM_BP_ALL	GO:0050658~RNA transport	22	1.2E-05	2.83	4.9E-02	2.4E-03	2.2E-02
Brown	GOTERM_BP_ALL	GO:0050657~nucleic acid transport	22	1.2E-05	2.83	4.9E-02	2.4E-03	2.2E-02
Brown	GOTERM_BP_ALL	GO:0051236~establishment of RNA localization	22	1.2E-05	2.83	4.9E-02	2.4E-03	2.2E-02

Brown	GOTERM_BP_ALL	GO:0006403~RNA localization	22	1.9E-05	2.75	8.1E-02	3.5E-03	3.6E-02
Brown	GOTERM_BP_ALL	GO:0051028~mRNA transport	20	1.9E-05	2.93	7.9E-02	3.6E-03	3.5E-02
Brown	UP_SEQ_FEATURE	repeat:TPR 6	15	5.5E-06	3.99	5.7E-02	4.9E-03	1.1E-02
Brown	UP_SEQ_FEATURE	zinc finger region:C2H2-type 13	17	6.3E-06	3.55	6.6E-02	5.2E-03	1.3E-02
Brown	UP_SEQ_FEATURE	repeat:TPR 5	15	8.4E-06	3.87	8.6E-02	6.0E-03	1.7E-02
Brown	UP_SEQ_FEATURE	zinc finger region:C2H2-type 1	39	7.9E-06	2.11	8.1E-02	6.0E-03	1.6E-02
Brown	GOTERM_BP_ALL	GO:0050789~regulation of biological process	351	3.5E-05	1.18	1.4E-01	6.0E-03	6.5E-02
Brown	GOTERM_CC_ALL	GO:0005694~chromosome	42	8.1E-05	1.86	6.0E-02	6.9E-03	1.2E-01
Brown	SP_PIR_KEYWORDS	Transcription	157	8.7E-05	1.32	7.5E-02	7.8E-03	1.4E-01
Brown	GOTERM_CC_ALL	GO:0044427~chromosomal part	36	1.1E-04	1.96	7.8E-02	8.1E-03	1.6E-01
Brown	UP_SEQ_FEATURE	repeat:TPR 7	13	1.5E-05	4.23	1.5E-01	9.8E-03	3.0E-02
Brown	INTERPRO	IPR000504:RNA recognition motif, RNP-1	37	9.3E-06	2.15	4.1E-02	1.0E-02	1.7E-02
Brown	GOTERM_BP_ALL	GO:0015931~nucleobase, nucleoside, nucleotide and nucleic acid transport	22	6.3E-05	2.56	2.4E-01	1.0E-02	1.2E-01
Brown	GOTERM_CC_ALL	GO:0005654~nucleoplasm	67	1.5E-04	1.57	1.1E-01	1.1E-02	2.3E-01
Brown	INTERPRO	IPR012677:Nucleotide-binding, alpha-beta plait	37	1.3E-05	2.12	5.6E-02	1.1E-02	2.4E-02
Brown	GOTERM_CC_ALL	GO:0005681~spliceosome	29	1.9E-04	2.08	1.3E-01	1.2E-02	2.9E-01
Brown	UP_SEQ_FEATURE	zinc finger region:C2H2-type 11	19	2.0E-05	3.03	1.9E-01	1.2E-02	4.1E-02
Brown	SP_PIR_KEYWORDS	zinc	167	1.8E-04	1.29	1.5E-01	1.3E-02	2.8E-01
Brown	SP_PIR_KEYWORDS	Transcription regulation	150	1.7E-04	1.32	1.4E-01	1.4E-02	2.6E-01
Brown	SP_PIR_KEYWORDS	mrna transport	16	2.5E-04	2.83	2.0E-01	1.7E-02	3.8E-01
Brown	UP_SEQ_FEATURE	repeat:TPR 4	16	3.0E-05	3.35	2.8E-01	1.8E-02	6.3E-02
Brown	GOTERM_CC_ALL	GO:0005622~intracellular	732	3.7E-04	1.05	2.5E-01	2.2E-02	5.7E-01
Brown	INTERPRO	IPR011545:DNA/RNA helicase, DEAD/DEAH box type, N-terminal	17	3.0E-05	3.19	1.3E-01	2.2E-02	5.6E-02
Brown	GOTERM_BP_ALL	GO:0044238~primary metabolic process	559	1.5E-04	1.09	4.9E-01	2.4E-02	2.9E-01
Brown	GOTERM_CC_ALL	GO:0031981~nuclear lumen	83	4.8E-04	1.44	3.1E-01	2.6E-02	7.2E-01
Brown	SP_PIR_KEYWORDS	spliceosome	26	5.3E-04	2.06	3.8E-01	2.8E-02	8.3E-01
Brown	SP_PIR_KEYWORDS	helicase	23	4.4E-04	2.20	3.3E-01	2.8E-02	6.8E-01
Brown	SP_PIR_KEYWORDS	zinc finger	24	4.7E-04	2.15	3.5E-01	2.8E-02	7.4E-01
Brown	SP_PIR_KEYWORDS	Chromosomal protein	16	5.3E-04	2.66	3.8E-01	2.9E-02	8.2E-01
Brown	UP_SEQ_FEATURE	zinc finger region:C2H2-type 12	17	5.9E-05	3.05	4.7E-01	3.3E-02	1.2E-01
Brown	GOTERM_MF_ALL	GO:0008026~ATP-dependent helicase activity	21	8.1E-05	2.59	1.7E-01	3.6E-02	1.4E-01
Brown	SMART	SM00487:DEXDc	24	1.4E-04	2.29	7.4E-02	3.8E-02	2.1E-01
Brown	GOTERM_MF_ALL	GO:0004386~helicase activity	26	1.0E-04	2.27	2.1E-01	3.8E-02	1.8E-01
Brown	GOTERM_MF_ALL	GO:0008270~zinc ion binding	184	7.6E-05	1.29	1.6E-01	4.2E-02	1.3E-01
Brown	GOTERM_MF_ALL	GO:0003723~RNA binding	90	5.9E-05	1.50	1.3E-01	4.4E-02	1.0E-01
Brown	SMART	SM00360:RRM	37	2.6E-04	1.83	1.3E-01	4.5E-02	3.7E-01
Brown	GOTERM_CC_ALL	GO:0044451~nucleoplasm part	57	9.8E-04	1.53	5.3E-01	4.9E-02	1.5E+00
Yellow	GOTERM_CC_ALL	GO:0005739~mitochondrion	227	1.9E-63	3.07	1.5E-60	1.5E-60	3.0E-60
Yellow	SP_PIR_KEYWORDS	Mitochondrion	189	1.1E-61	3.52	1.0E-58	1.0E-58	1.7E-58
Yellow	GOTERM_CC_ALL	GO:0044429~mitochondrial part	155	5.5E-51	3.55	4.2E-48	2.1E-48	8.4E-48
Yellow	KEGG_PATHWAY	hsa00190:Oxidative phosphorylation	74	2.8E-49	6.23	5.5E-47	5.5E-47	3.5E-46
Yellow	GOTERM_CC_ALL	GO:0044455~mitochondrial membrane part	62	6.4E-39	6.45	4.9E-36	1.6E-36	9.8E-36
Yellow	GOTERM_CC_ALL	GO:0005743~mitochondrial inner membrane	97	1.2E-37	4.12	9.2E-35	2.3E-35	1.8E-34
Yellow	GOTERM_CC_ALL	GO:0044444~cytoplasmic part	410	1.3E-35	1.63	1.0E-32	2.1E-33	2.0E-32
Yellow	GOTERM_CC_ALL	GO:0019866~organelle inner membrane	98	2.4E-35	3.88	1.9E-32	2.7E-33	3.7E-32
Yellow	GOTERM_CC_ALL	GO:0005740~mitochondrial envelope	110	2.3E-35	3.54	1.7E-32	2.9E-33	3.5E-32
Yellow	GOTERM_CC_ALL	GO:0031966~mitochondrial membrane	106	3.9E-34	3.54	3.0E-31	3.8E-32	6.0E-31
Yellow	SP_PIR_KEYWORDS	transit peptide	112	8.6E-34	3.40	7.8E-31	3.9E-31	1.3E-30
Yellow	GOTERM_BP_ALL	GO:0006119~oxidative phosphorylation	53	1.1E-34	6.84	4.7E-31	4.7E-31	2.0E-31
Yellow	GOTERM_CC_ALL	GO:0005746~mitochondrial respiratory chain	42	2.7E-28	6.95	2.1E-25	2.3E-26	4.1E-25
Yellow	GOTERM_CC_ALL	GO:0005737~cytoplasm	557	2.2E-27	1.34	1.7E-24	1.7E-25	3.3E-24
Yellow	GOTERM_CC_ALL	GO:0032991~macromolecular complex	271	8.8E-26	1.76	6.7E-23	6.1E-24	1.3E-22
Yellow	GOTERM_MF_ALL	GO:0003954~NADH dehydrogenase activity	34	5.7E-27	8.44	1.3E-23	6.5E-24	1.0E-23
Yellow	GOTERM_MF_ALL	GO:0050136~NADH dehydrogenase (quinone) activity	34	5.7E-27	8.44	1.3E-23	6.5E-24	1.0E-23
Yellow	GOTERM_MF_ALL	GO:0008137~NADH dehydrogenase (ubiquinone) activity	34	5.7E-27	8.44	1.3E-23	6.5E-24	1.0E-23
Yellow	GOTERM_CC_ALL	GO:0031967~organelle envelope	119	1.2E-24	2.62	9.6E-22	8.0E-23	1.9E-21
Yellow	GOTERM_CC_ALL	GO:0031975~envelope	119	1.6E-24	2.62	1.2E-21	9.2E-23	2.4E-21
Yellow	SP_PIR_KEYWORDS	ubiquinone	32	1.2E-24	8.10	1.1E-21	3.5E-22	1.8E-21
Yellow	GOTERM_MF_ALL	GO:0016655~oxidoreductase activity, acting on NADH or NADPH, quinone or similar compound as acceptor	34	5.3E-24	7.51	1.2E-20	3.0E-21	9.3E-21

Yellow	GOTERM_CC_ALL	GO:0005747~mitochondrial respiratory chain complex I	30	2.1E-22	7.72	1.6E-19	9.7E-21	3.3E-19
Yellow	GOTERM_CC_ALL	GO:0030964~NADH dehydrogenase complex (quinone)	30	2.1E-22	7.72	1.6E-19	9.7E-21	3.3E-19
Yellow	GOTERM_CC_ALL	GO:0045271~respiratory chain complex I	30	2.1E-22	7.72	1.6E-19	9.7E-21	3.3E-19
Yellow	GOTERM_CC_ALL	GO:0005761~mitochondrial ribosome	35	2.1E-22	6.62	1.6E-19	1.1E-20	3.1E-19
Yellow	GOTERM_CC_ALL	GO:0000313~organellar ribosome	35	2.1E-22	6.62	1.6E-19	1.1E-20	3.1E-19
Yellow	GOTERM_BP_ALL	GO:0042775~organelle ATP synthesis coupled electron transport	32	1.6E-23	7.74	6.9E-20	3.4E-20	3.0E-20
Yellow	GOTERM_BP_ALL	GO:0042773~ATP synthesis coupled electron transport	32	5.8E-23	7.55	2.5E-19	8.4E-20	1.1E-19
Yellow	GOTERM_BP_ALL	GO:0006120~mitochondrial electron transport, NADH to ubiquinone	29	4.8E-22	8.02	2.1E-18	5.3E-19	9.1E-19
Yellow	GOTERM_MF_ALL	GO:0015078~hydrogen ion transmembrane transporter activity	42	5.1E-21	5.28	1.2E-17	2.3E-18	8.9E-18
Yellow	GOTERM_MF_ALL	GO:0015077~monovalent inorganic cation transmembrane transporter activity	42	3.1E-20	5.09	7.1E-17	1.2E-17	5.4E-17
Yellow	GOTERM_MF_ALL	GO:0016651~oxidoreductase activity, acting on NADH or NADPH	36	5.1E-20	5.86	1.2E-16	1.7E-17	8.9E-17
Yellow	GOTERM_BP_ALL	GO:0006091~generation of precursor metabolites and energy	94	3.4E-20	2.69	1.5E-16	2.9E-17	6.3E-17
Yellow	GOTERM_MF_ALL	GO:0009055~electron carrier activity	55	4.9E-19	3.79	1.1E-15	1.4E-16	8.5E-16
Yellow	GOTERM_CC_ALL	GO:0030529~ribonucleoprotein complex	96	1.1E-17	2.46	8.2E-15	4.3E-16	1.6E-14
Yellow	SP_PIR_KEYWORDS	ribonucleoprotein	68	2.1E-18	3.14	1.9E-15	4.6E-16	3.2E-15
Yellow	SP_PIR_KEYWORDS	ribosomal protein	53	1.6E-17	3.64	1.4E-14	2.9E-15	2.5E-14
Yellow	GOTERM_CC_ALL	GO:0044446~intracellular organelle part	336	6.6E-16	1.43	5.1E-13	2.6E-14	1.0E-12
Yellow	GOTERM_CC_ALL	GO:0044422~organelle part	336	1.4E-15	1.42	1.1E-12	5.3E-14	2.2E-12
Yellow	GOTERM_CC_ALL	GO:0044424~intracellular part	692	3.3E-15	1.13	2.6E-12	1.2E-13	5.1E-12
Yellow	GOTERM_MF_ALL	GO:0022890~inorganic cation transmembrane transporter activity	43	5.6E-16	4.03	1.3E-12	1.4E-13	9.7E-13
Yellow	SP_PIR_KEYWORDS	inner membrane	33	2.4E-15	4.88	2.1E-12	3.5E-13	3.6E-12
Yellow	GOTERM_BP_ALL	GO:0006118~electron transport	67	1.2E-15	2.84	5.3E-12	8.8E-13	2.3E-12
Yellow	SP_PIR_KEYWORDS	acetylation	100	8.2E-15	2.21	7.4E-12	1.1E-12	1.3E-11
Yellow	SP_PIR_KEYWORDS	oxidoreductase	79	1.0E-14	2.49	9.4E-12	1.2E-12	1.6E-11
Yellow	GOTERM_CC_ALL	GO:0005840~ribosome	52	4.1E-14	3.13	3.1E-11	1.4E-12	6.3E-11
Yellow	SP_PIR_KEYWORDS	NAD	45	2.1E-14	3.55	1.9E-11	2.1E-12	3.3E-11
Yellow	GOTERM_CC_ALL	GO:0031090~organelle membrane	175	9.5E-14	1.69	7.3E-11	3.0E-12	1.4E-10
Yellow	UP_SEQ_FEATURE	transit peptide:Mitochondrion	67	3.4E-16	2.90	3.6E-12	3.6E-12	6.9E-13
Yellow	GOTERM_CC_ALL	GO:0043231~intracellular membrane-bound organelle	558	2.6E-13	1.20	2.0E-10	7.9E-12	4.0E-10
Yellow	GOTERM_CC_ALL	GO:0005759~mitochondrial matrix	48	3.2E-13	3.15	2.5E-10	8.8E-12	4.9E-10
Yellow	GOTERM_CC_ALL	GO:0031980~mitochondrial lumen	48	3.2E-13	3.15	2.5E-10	8.8E-12	4.9E-10
Yellow	GOTERM_CC_ALL	GO:0043227~membrane-bound organelle	558	3.1E-13	1.20	2.4E-10	9.1E-12	4.7E-10
Yellow	GOTERM_MF_ALL	GO:0016491~oxidoreductase activity	101	4.4E-14	2.14	1.0E-10	1.0E-11	7.7E-11
Yellow	GOTERM_CC_ALL	GO:0043234~protein complex	194	7.0E-13	1.60	5.4E-10	1.9E-11	1.1E-09
Yellow	GOTERM_MF_ALL	GO:0003735~structural constituent of ribosome	45	5.3E-13	3.29	1.2E-09	1.1E-10	9.3E-10
Yellow	SP_PIR_KEYWORDS	oxidative phosphorylation	18	2.8E-12	7.21	2.5E-09	2.5E-10	4.4E-09
Yellow	GOTERM_CC_ALL	GO:0005622~intracellular	703	1.4E-11	1.10	1.1E-08	3.6E-10	2.1E-08
Yellow	GOTERM_BP_ALL	GO:0044249~cellular biosynthetic process	121	5.9E-13	1.90	2.6E-09	3.7E-10	1.1E-09
Yellow	SP_PIR_KEYWORDS	respiratory chain	16	1.5E-11	7.69	1.3E-08	1.2E-09	2.3E-08
Yellow	GOTERM_CC_ALL	GO:0033279~ribosomal subunit	34	6.5E-11	3.50	5.0E-08	1.6E-09	9.9E-08
Yellow	GOTERM_CC_ALL	GO:0043226~organelle	591	1.2E-10	1.15	9.6E-08	3.0E-09	1.9E-07
Yellow	GOTERM_CC_ALL	GO:0043229~intracellular organelle	590	1.8E-10	1.15	1.4E-07	4.2E-09	2.8E-07
Yellow	GOTERM_CC_ALL	GO:0005762~mitochondrial large ribosomal subunit	14	3.8E-10	7.63	2.9E-07	8.4E-09	5.8E-07
Yellow	GOTERM_CC_ALL	GO:0000315~organellar large ribosomal subunit	14	3.8E-10	7.63	2.9E-07	8.4E-09	5.8E-07
Yellow	GOTERM_MF_ALL	GO:0046933~hydrogen ion transporting ATP synthase activity, rotational mechanism	19	5.3E-11	6.09	1.2E-07	1.0E-08	9.2E-08
Yellow	SP_PIR_KEYWORDS	hydrogen ion transport	20	1.4E-10	5.49	1.2E-07	1.0E-08	2.1E-07
Yellow	GOTERM_MF_ALL	GO:0046961~hydrogen ion transporting ATPase activity, rotational mechanism	19	1.1E-10	5.90	2.5E-07	1.9E-08	1.9E-07
Yellow	GOTERM_CC_ALL	GO:0005839~proteasome core complex (sensu Eukaryota)	14	1.2E-09	7.21	9.5E-07	2.6E-08	1.9E-06
Yellow	GOTERM_BP_ALL	GO:0006412~translation	76	5.1E-11	2.17	2.2E-07	2.8E-08	9.5E-08
Yellow	GOTERM_BP_ALL	GO:0006754~ATP biosynthetic process	20	6.0E-11	5.69	2.6E-07	2.9E-08	1.1E-07
Yellow	GOTERM_BP_ALL	GO:0006753~nucleoside phosphate metabolic process	20	6.0E-11	5.69	2.6E-07	2.9E-08	1.1E-07
Yellow	SP_PIR_KEYWORDS	Threonine protease	14	7.8E-10	7.48	7.1E-07	5.4E-08	1.2E-06
Yellow	KEGG_PATHWAY	hsa03050:Proteasome	16	5.9E-10	6.19	1.2E-07	5.8E-08	7.3E-07
Yellow	GOTERM_BP_ALL	GO:0015986~ATP synthesis coupled proton transport	19	1.7E-10	5.74	7.3E-07	6.7E-08	3.2E-07
Yellow	GOTERM_MF_ALL	GO:0004298~threonine endopeptidase activity	14	5.2E-10	7.73	1.2E-06	8.4E-08	9.0E-07
Yellow	GOTERM_BP_ALL	GO:0009206~purine ribonucleoside triphosphate biosynthetic process	22	3.0E-10	4.84	1.3E-06	8.6E-08	5.6E-07
Yellow	GOTERM_BP_ALL	GO:0009201~ribonucleoside triphosphate biosynthetic process	22	3.0E-10	4.84	1.3E-06	8.6E-08	5.6E-07
Yellow	GOTERM_BP_ALL	GO:0009145~purine nucleoside triphosphate biosynthetic process	22	3.0E-10	4.84	1.3E-06	8.6E-08	5.6E-07
Yellow	GOTERM_BP_ALL	GO:0046034~ATP metabolic process	21	2.7E-10	5.08	1.2E-06	9.9E-08	5.1E-07

Yellow	GOTERM_CC_ALL	GO:0016469~proton-transporting two-sector ATPase complex	20	4.9E-09	4.63	3.8E-06	1.0E-07	7.5E-06
Yellow	GOTERM_BP_ALL	GO:0009142~nucleoside triphosphate biosynthetic process	22	5.0E-10	4.73	2.2E-06	1.4E-07	9.4E-07
Yellow	GOTERM_CC_ALL	GO:0045259~proton-transporting ATP synthase complex	14	9.1E-09	6.48	7.0E-06	1.8E-07	1.4E-05
Yellow	GOTERM_BP_ALL	GO:0009205~purine ribonucleoside triphosphate metabolic process	23	7.9E-10	4.45	3.4E-06	1.9E-07	1.5E-06
Yellow	GOTERM_BP_ALL	GO:0009199~ribonucleoside triphosphate metabolic process	23	7.9E-10	4.45	3.4E-06	1.9E-07	1.5E-06
Yellow	GOTERM_BP_ALL	GO:0009144~purine nucleoside triphosphate metabolic process	23	7.9E-10	4.45	3.4E-06	1.9E-07	1.5E-06
Yellow	GOTERM_BP_ALL	GO:0009058~biosynthetic process	139	9.0E-10	1.63	3.9E-06	1.9E-07	1.7E-06
Yellow	GOTERM_BP_ALL	GO:0009260~ribonucleotide biosynthetic process	25	1.4E-09	4.03	6.2E-06	2.8E-07	2.7E-06
Yellow	GOTERM_BP_ALL	GO:0009259~ribonucleotide metabolic process	27	1.4E-09	3.79	6.0E-06	2.8E-07	2.6E-06
Yellow	GOTERM_MF_ALL	GO:0019829~cation-transporting ATPase activity	20	2.6E-09	4.85	5.8E-06	3.9E-07	4.5E-06
Yellow	GOTERM_BP_ALL	GO:0009141~nucleoside triphosphate metabolic process	23	3.0E-09	4.20	1.3E-05	5.8E-07	5.7E-06
Yellow	SP_PIR_KEYWORDS	proteasome	22	9.5E-09	4.15	8.6E-06	6.1E-07	1.5E-05
Yellow	GOTERM_CC_ALL	GO:0000502~proteasome complex (sensu Eukaryota)	20	3.5E-08	4.21	2.7E-05	6.8E-07	5.3E-05
Yellow	INTERPRO	IPR001353:20S proteasome, A and B subunits	14	1.7E-10	8.14	7.5E-07	7.5E-07	3.2E-07
Yellow	GOTERM_BP_ALL	GO:0009150~purine ribonucleotide metabolic process	25	4.6E-09	3.84	2.0E-05	8.4E-07	8.6E-06
Yellow	SP_PIR_KEYWORDS	membrane-associated complex	14	3.0E-08	6.12	2.7E-05	1.7E-06	4.8E-05
Yellow	SP_PIR_KEYWORDS	mitochondrial inner membrane	11	3.0E-08	8.14	2.7E-05	1.8E-06	4.6E-05
Yellow	GOTERM_BP_ALL	GO:0009152~purine ribonucleotide biosynthetic process	23	1.0E-08	3.97	4.5E-05	1.8E-06	2.0E-05
Yellow	SP_PIR_KEYWORDS	ATP synthesis	14	6.3E-08	5.85	5.7E-05	3.4E-06	9.9E-05
Yellow	GOTERM_MF_ALL	GO:0008324~cation transmembrane transporter activity	46	2.5E-08	2.40	5.7E-05	3.6E-06	4.4E-05
Yellow	GOTERM_BP_ALL	GO:0006163~purine nucleotide metabolic process	25	2.7E-08	3.56	1.2E-04	4.5E-06	5.0E-05
Yellow	GOTERM_MF_ALL	GO:0004129~cytochrome-c oxidase activity	14	4.3E-08	6.05	9.7E-05	5.7E-06	7.4E-05
Yellow	GOTERM_MF_ALL	GO:0016676~oxidoreductase activity, acting on heme group of donors, oxygen as acceptor	14	4.3E-08	6.05	9.7E-05	5.7E-06	7.4E-05
Yellow	GOTERM_MF_ALL	GO:0016675~oxidoreductase activity, acting on heme group of donors	14	4.3E-08	6.05	9.7E-05	5.7E-06	7.4E-05
Yellow	GOTERM_MF_ALL	GO:0015002~heme-copper terminal oxidase activity	14	4.3E-08	6.05	9.7E-05	5.7E-06	7.4E-05
Yellow	GOTERM_BP_ALL	GO:0006164~purine nucleotide biosynthetic process	23	6.5E-08	3.65	2.8E-04	1.1E-05	1.2E-04
Yellow	GOTERM_CC_ALL	GO:0015934~large ribosomal subunit	19	7.1E-07	3.74	5.4E-04	1.4E-05	1.1E-03
Yellow	GOTERM_BP_ALL	GO:0015992~proton transport	20	9.2E-08	4.03	4.0E-04	1.4E-05	1.7E-04
Yellow	GOTERM_BP_ALL	GO:0051186~cofactor metabolic process	39	9.2E-08	2.52	4.0E-04	1.4E-05	1.7E-04
Yellow	GOTERM_BP_ALL	GO:0006818~hydrogen transport	20	1.4E-07	3.95	5.9E-04	2.0E-05	2.6E-04
Yellow	GOTERM_MF_ALL	GO:0015405~P-P-bond-hydrolysis-driven transmembrane transporter activity	27	2.0E-07	3.08	4.5E-04	2.1E-05	3.4E-04
Yellow	GOTERM_MF_ALL	GO:0015399~primary active transmembrane transporter activity	27	2.0E-07	3.08	4.5E-04	2.1E-05	3.4E-04
Yellow	SP_PIR_KEYWORDS	Direct protein sequencing	195	5.1E-07	1.38	4.6E-04	2.6E-05	8.0E-04
Yellow	GOTERM_CC_ALL	GO:0005763~mitochondrial small ribosomal subunit	11	1.9E-06	5.99	1.5E-03	3.6E-05	2.9E-03
Yellow	GOTERM_CC_ALL	GO:0000314~organellar small ribosomal subunit	11	1.9E-06	5.99	1.5E-03	3.6E-05	2.9E-03
Yellow	GOTERM_BP_ALL	GO:0009059~macromolecule biosynthetic process	87	3.7E-07	1.71	1.6E-03	5.2E-05	7.0E-04
Yellow	SP_PIR_KEYWORDS	transport	113	1.8E-06	1.53	1.6E-03	8.5E-05	2.8E-03
Yellow	SP_PIR_KEYWORDS	protein transport	63	2.2E-06	1.83	2.0E-03	1.0E-04	3.5E-03
Yellow	GOTERM_CC_ALL	GO:0033177~proton-transporting two-sector ATPase complex, proton-transporting domain	9	5.9E-06	6.95	4.5E-03	1.0E-04	9.0E-03
Yellow	GOTERM_CC_ALL	GO:0045263~proton-transporting ATP synthase complex, coupling factor F(o)	9	5.9E-06	6.95	4.5E-03	1.0E-04	9.0E-03
Yellow	SP_PIR_KEYWORDS	redox-active center	15	2.9E-06	4.24	2.6E-03	1.2E-04	4.5E-03
Yellow	GOTERM_BP_ALL	GO:0006626~protein targeting to mitochondrion	12	9.2E-07	5.80	4.0E-03	1.3E-04	1.7E-03
Yellow	SP_PIR_KEYWORDS	electron transfer	12	3.4E-06	5.25	3.1E-03	1.4E-04	5.3E-03
Yellow	SP_PIR_KEYWORDS	CF(0)	9	4.4E-06	7.21	4.0E-03	1.7E-04	6.9E-03
Yellow	SP_PIR_KEYWORDS	chaperone	28	4.8E-06	2.59	4.3E-03	1.8E-04	7.4E-03
Yellow	GOTERM_BP_ALL	GO:0009108~coenzyme biosynthetic process	22	1.4E-06	3.22	6.2E-03	1.8E-04	2.7E-03
Yellow	SP_PIR_KEYWORDS	er-golgi transport	21	5.1E-06	3.11	4.6E-03	1.8E-04	8.0E-03
Yellow	GOTERM_BP_ALL	GO:0051188~cofactor biosynthetic process	25	1.4E-06	2.95	6.1E-03	1.9E-04	2.7E-03
Yellow	GOTERM_MF_ALL	GO:0042625~ATPase activity, coupled to transmembrane movement of ions	20	1.9E-06	3.43	4.3E-03	1.9E-04	3.3E-03
Yellow	GOTERM_MF_ALL	GO:0015075~ion transmembrane transporter activity	50	2.0E-06	2.01	4.5E-03	1.9E-04	3.4E-03
Yellow	GOTERM_CC_ALL	GO:0031974~membrane-enclosed lumen	112	1.2E-05	1.47	9.5E-03	2.1E-04	1.9E-02
Yellow	GOTERM_CC_ALL	GO:0043233~organelle lumen	112	1.2E-05	1.47	9.5E-03	2.1E-04	1.9E-02
Yellow	GOTERM_CC_ALL	GO:0005758~mitochondrial intermembrane space	11	1.3E-05	5.09	1.0E-02	2.2E-04	2.1E-02
Yellow	GOTERM_BP_ALL	GO:0043681~protein import into mitochondrion	9	4.2E-06	7.26	1.8E-02	5.2E-04	7.9E-03
Yellow	GOTERM_MF_ALL	GO:0022891~substrate-specific transmembrane transporter activity	57	5.8E-06	1.84	1.3E-02	5.3E-04	1.0E-02
Yellow	GOTERM_CC_ALL	GO:0005753~mitochondrial proton-transporting ATP synthase complex	8	3.7E-05	6.74	2.8E-02	5.9E-04	5.6E-02
Yellow	GOTERM_CC_ALL	GO:0005744~mitochondrial inner membrane presequence translocase complex	8	3.7E-05	6.74	2.8E-02	5.9E-04	5.6E-02
Yellow	GOTERM_CC_ALL	GO:0031970~organelle envelope lumen	11	3.9E-05	4.63	2.9E-02	5.9E-04	5.9E-02
Yellow	GOTERM_CC_ALL	GO:0015935~small ribosomal subunit	16	4.2E-05	3.29	3.1E-02	6.3E-04	6.4E-02

Yellow	GOTERM_BP_ALL	GO:0009165~nucleotide biosynthetic process	26	5.9E-06	2.68	2.5E-02	7.1E-04	1.1E-02
Yellow	GOTERM_BP_ALL	GO:0006511~ubiquitin-dependent protein catabolic process	32	6.7E-06	2.36	2.9E-02	7.8E-04	1.2E-02
Yellow	GOTERM_BP_ALL	GO:0006732~coenzyme metabolic process	30	7.1E-06	2.44	3.1E-02	8.0E-04	1.3E-02
Yellow	GOTERM_BP_ALL	GO:0015031~protein transport	87	7.0E-06	1.59	3.0E-02	8.0E-04	1.3E-02
Yellow	GOTERM_BP_ALL	GO:0008152~metabolic process	539	7.4E-06	1.10	3.2E-02	8.1E-04	1.4E-02
Yellow	GOTERM_CC_ALL	GO:0005623~cell	784	6.7E-05	1.02	5.0E-02	9.7E-04	1.0E-01
Yellow	GOTERM_CC_ALL	GO:0044464~cell part	784	6.7E-05	1.02	5.0E-02	9.7E-04	1.0E-01
Yellow	GOTERM_BP_ALL	GO:0043632~modification-dependent macromolecule catabolic process	32	9.3E-06	2.33	4.0E-02	9.8E-04	1.7E-02
Yellow	GOTERM_BP_ALL	GO:0019941~modification-dependent protein catabolic process	32	9.3E-06	2.33	4.0E-02	9.8E-04	1.7E-02
Yellow	GOTERM_BP_ALL	GO:0051603~proteolysis involved in cellular protein catabolic process	32	1.3E-05	2.29	5.4E-02	1.3E-03	2.4E-02
Yellow	GOTERM_BP_ALL	GO:0044257~cellular protein catabolic process	32	1.5E-05	2.28	6.3E-02	1.5E-03	2.8E-02
Yellow	GOTERM_CC_ALL	GO:0005829~cytosol	63	1.1E-04	1.62	8.1E-02	1.6E-03	1.7E-01
Yellow	GOTERM_BP_ALL	GO:0045184~establishment of protein localization	88	1.9E-05	1.55	7.8E-02	1.8E-03	3.5E-02
Yellow	GOTERM_MF_ALL	GO:0016681~oxidoreductase activity, acting on diphenols and related substances as donors, cytochrome as acceptor	7	2.4E-05	8.69	5.3E-02	2.1E-03	4.1E-02
Yellow	GOTERM_MF_ALL	GO:0016679~oxidoreductase activity, acting on diphenols and related substances as donors	7	2.4E-05	8.69	5.3E-02	2.1E-03	4.1E-02
Yellow	GOTERM_MF_ALL	GO:0008121~ubiquinol-cytochrome-c reductase activity	7	2.4E-05	8.69	5.3E-02	2.1E-03	4.1E-02
Yellow	GOTERM_BP_ALL	GO:0008104~protein localization	90	3.3E-05	1.52	1.3E-01	3.1E-03	6.2E-02
Yellow	GOTERM_BP_ALL	GO:0055086~nucleobase, nucleoside and nucleotide metabolic process	33	3.4E-05	2.16	1.4E-01	3.1E-03	6.4E-02
Yellow	GOTERM_MF_ALL	GO:0042626~ATPase activity, coupled to transmembrane movement of substances	21	4.1E-05	2.74	8.8E-02	3.2E-03	7.1E-02
Yellow	GOTERM_MF_ALL	GO:0016820~hydrolase activity, acting on acid anhydrides, catalyzing transmembrane movement of substances	21	5.0E-05	2.71	1.1E-01	3.8E-03	8.7E-02
Yellow	GOTERM_MF_ALL	GO:0043492~ATPase activity, coupled to movement of substances	21	5.0E-05	2.71	1.1E-01	3.8E-03	8.7E-02
Yellow	GOTERM_BP_ALL	GO:0030163~protein catabolic process	35	4.9E-05	2.06	1.9E-01	4.4E-03	9.1E-02
Yellow	GOTERM_BP_ALL	GO:0007005~mitochondrion organization and biogenesis	20	5.7E-05	2.76	2.2E-01	5.0E-03	1.1E-01
Yellow	GOTERM_MF_ALL	GO:0022857~transmembrane transporter activity	58	1.0E-04	1.67	2.0E-01	7.1E-03	1.7E-01
Yellow	GOTERM_CC_ALL	GO:0042719~mitochondrial intermembrane space protein transporter complex	5	6.1E-04	9.26	3.8E-01	8.5E-03	9.3E-01
Yellow	SMART	SM00651:Sm	8	1.7E-05	8.54	9.0E-03	9.0E-03	2.4E-02
Yellow	GOTERM_MF_ALL	GO:0022892~substrate-specific transporter activity	68	1.3E-04	1.57	2.6E-01	9.2E-03	2.3E-01
Yellow	COG_ONTOLOGY	Translation, ribosomal structure and biogenesis	20	1.5E-04	2.53	1.0E-02	1.0E-02	1.5E-01
Yellow	INTERPRO	IPR012335:Thioredoxin fold	21	5.6E-06	3.10	2.5E-02	1.2E-02	1.0E-02
Yellow	GOTERM_BP_ALL	GO:0006839~mitochondrial transport	13	1.6E-04	3.49	5.0E-01	1.4E-02	3.0E-01
Yellow	SP_PIR_KEYWORDS	electron transport	12	4.3E-04	3.39	3.2E-01	1.5E-02	6.7E-01
Yellow	GOTERM_CC_ALL	GO:0033178~proton-transporting two-sector ATPase complex, catalytic domain	6	1.3E-03	6.18	6.2E-01	1.7E-02	1.9E+00
Yellow	GOTERM_MF_ALL	GO:0003824~catalytic activity	348	2.8E-04	1.15	4.7E-01	1.9E-02	4.9E-01
Yellow	COG_ONTOLOGY	Posttranslational modification, protein turnover, chaperones	23	6.0E-04	2.12	4.1E-02	2.1E-02	6.2E-01
Yellow	GOTERM_BP_ALL	GO:0033036~macromolecule localization	90	2.6E-04	1.44	6.8E-01	2.2E-02	4.9E-01
Yellow	GOTERM_CC_ALL	GO:0005832~chaperonin-containing T-complex	5	1.7E-03	7.72	7.3E-01	2.2E-02	2.5E+00
Yellow	GOTERM_BP_ALL	GO:0045454~cell redox homeostasis	14	3.4E-04	3.08	7.7E-01	2.8E-02	6.3E-01
Yellow	GOTERM_BP_ALL	GO:0009117~nucleotide metabolic process	29	3.7E-04	2.02	8.0E-01	3.0E-02	6.9E-01
Yellow	SMART	SM00440:ZnF_C2C2	5	1.2E-04	15.13	6.4E-02	3.2E-02	1.8E-01
Yellow	GOTERM_BP_ALL	GO:0006886~intracellular protein transport	53	4.2E-04	1.62	8.4E-01	3.3E-02	7.9E-01
Yellow	GOTERM_BP_ALL	GO:0051641~cellular localization	85	4.4E-04	1.43	8.5E-01	3.4E-02	8.2E-01
Yellow	SP_PIR_KEYWORDS	antioxidant	6	1.1E-03	6.41	6.1E-01	3.5E-02	1.6E+00
Yellow	INTERPRO	IPR000243:Peptidase T1A, proteasome beta-subunit	8	2.4E-05	7.19	1.0E-01	3.5E-02	4.5E-02
Yellow	GOTERM_BP_ALL	GO:0045039~protein import into mitochondrial inner membrane	5	5.2E-04	9.67	8.9E-01	3.9E-02	9.7E-01
Yellow	GOTERM_BP_ALL	GO:0051649~establishment of cellular localization	83	5.7E-04	1.43	9.2E-01	4.2E-02	1.1E+00
Yellow	GOTERM_BP_ALL	GO:0046907~intracellular transport	75	5.9E-04	1.46	9.2E-01	4.3E-02	1.1E+00
Yellow	GOTERM_MF_ALL	GO:0045182~translation regulator activity	22	6.9E-04	2.21	7.9E-01	4.4E-02	1.2E+00
Green	GOTERM_CC_ALL	GO:0044444~cytoplasmic part	243	3.8E-05	1.23	2.9E-02	2.9E-02	5.8E-02
Green	KEGG_PATHWAY	hsa00626:Naphthalene and anthracene degradation	7	1.8E-04	7.03	3.4E-02	3.4E-02	2.2E-01
Red	SP_PIR_KEYWORDS	phosphoprotein	265	2.3E-07	1.27	2.1E-04	2.1E-04	3.6E-04
Black	SP_PIR_KEYWORDS	phosphoprotein	259	2.7E-16	1.48	2.0E-13	2.0E-13	3.4E-13
Black	SP_PIR_KEYWORDS	nucleus	189	4.4E-11	1.50	4.0E-08	2.0E-08	6.9E-08
Black	GOTERM_CC_ALL	GO:0005634~nucleus	222	4.8E-11	1.41	3.7E-08	3.7E-08	7.4E-08
Black	GOTERM_MF_ALL	GO:0003676~nucleic acid binding	166	3.1E-11	1.58	7.1E-08	7.1E-08	5.4E-08
Black	GOTERM_BP_ALL	GO:0016070~RNA metabolic process	150	7.7E-10	1.56	3.4E-06	3.4E-06	1.4E-06
Black	SP_PIR_KEYWORDS	dna-binding	84	1.3E-08	1.87	1.1E-05	3.8E-06	2.0E-05
Black	INTERPRO	IPR000504:RNA recognition motif, RNP-1	29	4.5E-09	3.60	2.0E-05	2.0E-05	8.4E-06
Black	GOTERM_BP_ALL	GO:0043283~biopolymer metabolic process	225	1.5E-08	1.32	6.6E-05	3.3E-05	2.9E-05
Black	GOTERM_BP_ALL	GO:0006139~nucleobase, nucleoside, nucleotide and nucleic acid metabolic process	177	2.7E-08	1.41	1.2E-04	3.9E-05	5.1E-05

Black	SMART	SM00360:RRM	30	8.8E-08	3.02	4.7E-05	4.7E-05	1.3E-04
Black	INTERPRO	IPR012677:Nucleotide-binding, alpha-beta plait	28	2.6E-08	3.43	1.2E-04	5.9E-05	4.9E-05
Black	SP_PIR_KEYWORDS	zinc-finger	82	1.9E-06	1.68	1.7E-03	2.8E-04	2.9E-03
Black	SP_PIR_KEYWORDS	rna-binding	43	1.4E-06	2.21	1.3E-03	3.1E-04	2.2E-03
Black	SP_PIR_KEYWORDS	Transcription regulation	87	1.8E-06	1.65	1.6E-03	3.3E-04	2.8E-03
Black	SP_PIR_KEYWORDS	Transcription	89	2.9E-06	1.62	2.6E-03	3.7E-04	4.5E-03
Black	GOTERM_MF_ALL	GO:0003723~RNA binding	59	3.3E-07	2.00	7.4E-04	3.7E-04	5.7E-04
Black	GOTERM_MF_ALL	GO:0003677~DNA binding	105	5.9E-07	1.58	1.3E-03	4.5E-04	1.0E-03
Black	SP_PIR_KEYWORDS	activator	35	7.7E-06	2.29	6.9E-03	8.6E-04	1.2E-02
Black	GOTERM_BP_ALL	GO:0010467~gene expression	160	8.9E-07	1.38	3.8E-03	9.6E-04	1.7E-03
Black	GOTERM_MF_ALL	GO:0008270~zinc ion binding	106	5.1E-06	1.51	1.1E-02	2.9E-03	8.8E-03
Black	GOTERM_BP_ALL	GO:0016071~mRNA metabolic process	34	6.5E-06	2.34	2.8E-02	4.7E-03	1.2E-02
Black	GOTERM_BP_ALL	GO:0006396~RNA processing	43	5.8E-06	2.09	2.5E-02	5.0E-03	1.1E-02
Black	GOTERM_BP_ALL	GO:0008380~RNA splicing	29	9.0E-06	2.52	3.8E-02	5.6E-03	1.7E-02
Black	GOTERM_MF_ALL	GO:0005488~binding	402	1.5E-05	1.08	3.3E-02	6.7E-03	2.6E-02
Black	GOTERM_BP_ALL	GO:0050789~regulation of biological process	188	1.6E-05	1.28	6.8E-02	8.8E-03	3.0E-02
Black	GOTERM_BP_ALL	GO:0006355~regulation of transcription, DNA-dependent	103	2.5E-05	1.46	1.0E-01	1.2E-02	4.6E-02
Black	SP_PIR_KEYWORDS	zinc	88	1.4E-04	1.47	1.2E-01	1.4E-02	2.1E-01
Black	GOTERM_BP_ALL	GO:0019219~regulation of nucleobase, nucleoside, nucleotide and nucleic acid metabolic process	111	3.2E-05	1.43	1.3E-01	1.4E-02	5.9E-02
Black	GOTERM_BP_ALL	GO:0045449~regulation of transcription	108	4.0E-05	1.43	1.6E-01	1.6E-02	7.6E-02
Black	GOTERM_BP_ALL	GO:0006350~transcription	112	5.2E-05	1.41	2.0E-01	1.7E-02	9.7E-02
Black	GOTERM_BP_ALL	GO:0010468~regulation of gene expression	114	4.9E-05	1.40	1.9E-01	1.8E-02	9.2E-02
Black	GOTERM_BP_ALL	GO:0050794~regulation of cellular process	175	6.8E-05	1.27	2.6E-01	1.8E-02	1.3E-01
Black	GOTERM_BP_ALL	GO:0032774~RNA biosynthetic process	104	6.5E-05	1.43	2.4E-01	1.9E-02	1.2E-01
Black	GOTERM_BP_ALL	GO:0006351~transcription, DNA-dependent	104	6.3E-05	1.43	2.4E-01	1.9E-02	1.2E-01
Black	SP_PIR_KEYWORDS	repressor	28	2.5E-04	2.14	2.0E-01	2.2E-02	3.9E-01
Black	GOTERM_BP_ALL	GO:0006397~mRNA processing	28	1.0E-04	2.25	3.6E-01	2.6E-02	1.9E-01
Black	GOTERM_BP_ALL	GO:0065007~biological regulation	196	1.2E-04	1.23	4.0E-01	2.8E-02	2.2E-01
Black	GOTERM_BP_ALL	GO:0031323~regulation of cellular metabolic process	116	1.6E-04	1.36	5.0E-01	3.6E-02	3.0E-01
Black	GOTERM_BP_ALL	GO:0019222~regulation of metabolic process	120	1.7E-04	1.35	5.3E-01	3.7E-02	3.3E-01
Black	SP_PIR_KEYWORDS	mrna splicing	21	5.1E-04	2.36	3.7E-01	3.8E-02	7.9E-01
Black	SP_PIR_KEYWORDS	alternative splicing	212	5.0E-04	1.19	3.6E-01	4.0E-02	7.8E-01
Black	GOTERM_BP_ALL	GO:0043170~macromolecule metabolic process	258	2.3E-04	1.15	6.3E-01	4.7E-02	4.3E-01
Black	GOTERM_CC_ALL	GO:0043226~organelle	312	1.9E-04	1.12	1.3E-01	4.7E-02	2.8E-01
Pink	SP_PIR_KEYWORDS	glycoprotein	217	5.1E-57	2.89	4.6E-54	4.6E-54	8.0E-54
Pink	SP_PIR_KEYWORDS	signal	188	3.6E-51	3.04	3.2E-48	1.6E-48	5.6E-48
Pink	UP_SEQ_FEATURE	signal peptide	158	1.0E-40	2.88	1.1E-36	1.1E-36	2.1E-37
Pink	UP_SEQ_FEATURE	glycosylation site:N-linked (GlcNAc...)	164	2.8E-38	2.68	3.0E-34	1.5E-34	5.7E-35
Pink	SP_PIR_KEYWORDS	Secreted	96	1.4E-31	3.77	1.3E-28	4.2E-29	2.2E-28
Pink	INTERPRO	IPR015492:Protocadherin gamma	21	6.0E-24	16.46	2.7E-20	2.7E-20	1.1E-20
Pink	GOTERM_BP_ALL	GO:0022610~biological adhesion	74	3.5E-21	3.36	1.5E-17	7.6E-18	6.6E-18
Pink	GOTERM_BP_ALL	GO:0007155~cell adhesion	74	3.5E-21	3.36	1.5E-17	7.6E-18	6.6E-18
Pink	UP_SEQ_FEATURE	disulfide bond	104	4.2E-20	2.50	4.5E-16	1.5E-16	8.6E-17
Pink	SP_PIR_KEYWORDS	cell adhesion	48	3.4E-18	4.33	3.1E-15	7.7E-16	5.3E-15
Pink	INTERPRO	IPR013164:Cadherin, N-terminal	21	5.9E-19	12.34	2.6E-15	1.3E-15	1.1E-15
Pink	GOTERM_CC_ALL	GO:0005576~extracellular region	75	2.6E-18	2.99	2.0E-15	2.0E-15	4.0E-15
Pink	SP_PIR_KEYWORDS	extracellular matrix	35	2.5E-17	5.69	2.3E-14	4.6E-15	4.0E-14
Pink	GOTERM_CC_ALL	GO:0031012~extracellular matrix	43	2.3E-17	4.57	1.8E-14	9.0E-15	3.6E-14
Pink	UP_SEQ_FEATURE	domain:Cadherin 5	22	6.0E-18	10.56	6.5E-14	1.6E-14	1.2E-14
Pink	UP_SEQ_FEATURE	domain:Cadherin 6	21	9.6E-18	11.09	1.0E-13	2.1E-14	2.0E-14
Pink	UP_SEQ_FEATURE	domain:Cadherin 4	22	1.5E-17	10.25	1.6E-13	2.7E-14	3.0E-14
Pink	UP_SEQ_FEATURE	domain:Cadherin 3	22	1.5E-17	10.25	1.6E-13	2.7E-14	3.0E-14
Pink	GOTERM_CC_ALL	GO:0005578~proteinaceous extracellular matrix	42	8.7E-17	4.53	8.5E-14	2.8E-14	1.7E-13
Pink	UP_SEQ_FEATURE	domain:Cadherin 2	22	7.9E-17	9.68	1.2E-12	1.3E-13	2.3E-13
Pink	UP_SEQ_FEATURE	domain:Cadherin 1	22	7.9E-17	9.68	1.2E-12	1.3E-13	2.3E-13
Pink	INTERPRO	IPR002126:Cadherin	22	8.8E-17	9.79	5.0E-13	1.7E-13	2.1E-13
Pink	GOTERM_CC_ALL	GO:0044421~extracellular region part	58	2.0E-15	3.20	1.5E-12	3.8E-13	3.1E-12
Pink	SMART	SM00112:CA	22	1.3E-15	8.50	7.2E-13	7.2E-13	1.9E-12
Pink	SP_PIR_KEYWORDS	transmembrane	163	9.9E-14	1.71	8.9E-11	1.5E-11	1.5E-10

Pink	GOTERM_BP_ALL	GO:0007156~homophilic cell adhesion	24	2.0E-14	7.13	8.8E-11	2.9E-11	3.8E-11
Pink	GOTERM_BP_ALL	GO:0016337~cell-cell adhesion	33	1.5E-13	4.65	6.6E-10	1.6E-10	2.8E-10
Pink	GOTERM_CC_ALL	GO:0005886~plasma membrane	126	3.1E-12	1.81	2.4E-09	4.8E-10	4.7E-09
Pink	SP_PIR_KEYWORDS	calcium	52	4.5E-12	2.90	4.1E-09	5.8E-10	7.0E-09
Pink	GOTERM_MF_ALL	GO:0005509~calcium ion binding	64	8.6E-13	2.63	2.0E-09	2.0E-09	1.5E-09
Pink	GOTERM_CC_ALL	GO:0031224~intrinsic to membrane	187	2.2E-11	1.52	1.7E-08	2.8E-09	3.3E-08
Pink	GOTERM_BP_ALL	GO:0032501~multicellular organismal process	145	5.0E-12	1.69	2.2E-08	4.4E-09	9.4E-09
Pink	SP_PIR_KEYWORDS	membrane	198	5.6E-11	1.48	5.1E-08	6.4E-09	8.8E-08
Pink	GOTERM_CC_ALL	GO:0016021~integral to membrane	185	6.3E-11	1.51	4.9E-08	6.9E-09	9.7E-08
Pink	SP_PIR_KEYWORDS	hydroxylation	16	2.5E-10	7.81	2.3E-07	2.5E-08	3.9E-07
Pink	UP_SEQ_FEATURE	topological domain:Extracellular	79	2.8E-11	2.15	3.0E-07	3.0E-08	5.8E-08
Pink	GOTERM_CC_ALL	GO:0016020~membrane	249	2.0E-09	1.33	1.5E-06	1.9E-07	3.0E-06
Pink	UP_SEQ_FEATURE	transmembrane region	125	2.5E-10	1.68	2.7E-06	2.5E-07	5.2E-07
Pink	UP_SEQ_FEATURE	topological domain:Cytoplasmic	97	3.5E-10	1.86	3.8E-06	3.1E-07	7.2E-07
Pink	GOTERM_BP_ALL	GO:0006817~phosphate transport	17	1.6E-09	6.46	7.1E-06	1.2E-06	3.1E-06
Pink	GOTERM_CC_ALL	GO:0044425~membrane part	204	2.2E-08	1.37	1.7E-05	1.9E-06	3.3E-05
Pink	GOTERM_CC_ALL	GO:0044420~extracellular matrix part	19	3.1E-08	4.80	2.4E-05	2.4E-06	4.7E-05
Pink	GOTERM_MF_ALL	GO:0005201~extracellular matrix structural constituent	17	2.8E-09	6.29	6.3E-06	3.1E-06	4.8E-06
Pink	GOTERM_BP_ALL	GO:0048856~anatomical structure development	108	7.4E-09	1.70	3.2E-05	4.6E-06	1.4E-05
Pink	SP_PIR_KEYWORDS	collagen	15	7.5E-08	5.93	6.7E-05	6.7E-06	1.2E-04
Pink	GOTERM_BP_ALL	GO:0007275~multicellular organismal development	111	1.5E-08	1.66	6.7E-05	8.3E-06	2.9E-05
Pink	SP_PIR_KEYWORDS	trimer	10	1.5E-07	9.76	1.4E-04	1.2E-05	2.3E-04
Pink	SP_PIR_KEYWORDS	hydroxylysine	10	2.9E-07	9.22	2.6E-04	2.1E-05	4.5E-04
Pink	GOTERM_BP_ALL	GO:0048731~system development	88	6.3E-08	1.76	2.7E-04	3.0E-05	1.2E-04
Pink	INTERPRO	IPR008160:Collagen triple helix repeat	15	2.8E-08	6.33	1.2E-04	3.1E-05	5.2E-05
Pink	SP_PIR_KEYWORDS	hydroxyproline	10	5.1E-07	8.73	4.6E-04	3.6E-05	8.0E-04
Pink	UP_SEQ_FEATURE	short sequence motif:Cell attachment site	16	6.2E-08	5.51	6.7E-04	5.1E-05	1.3E-04
Pink	SP_PIR_KEYWORDS	egf-like domain	21	9.2E-07	3.59	8.3E-04	5.9E-05	1.4E-03
Pink	INTERPRO	IPR007110:Immunoglobulin-like	28	7.9E-08	3.25	3.6E-04	5.9E-05	1.5E-04
Pink	INTERPRO	IPR003598:Immunoglobulin subtype 2	19	6.6E-08	4.60	3.0E-04	6.0E-05	1.3E-04
Pink	SP_PIR_KEYWORDS	immunoglobulin domain	26	1.1E-06	3.02	9.9E-04	6.6E-05	1.7E-03
Pink	GOTERM_BP_ALL	GO:0015698~inorganic anion transport	18	2.0E-07	4.52	8.9E-04	8.9E-05	3.8E-04
Pink	SMART	SM00408:IGc2	19	4.8E-07	4.00	2.6E-04	1.3E-04	6.9E-04
Pink	GOTERM_BP_ALL	GO:0009605~response to external stimulus	41	3.3E-07	2.38	1.4E-03	1.3E-04	6.2E-04
Pink	KEGG_PATHWAY	hsa04610:Complement and coagulation cascades	13	1.7E-06	5.32	3.5E-04	1.7E-04	2.2E-03
Pink	GOTERM_CC_ALL	GO:0005581~collagen	10	2.9E-06	7.35	2.2E-03	2.0E-04	4.4E-03
Pink	SP_PIR_KEYWORDS	pyroglutamic acid	9	4.1E-06	8.30	3.7E-03	2.3E-04	6.4E-03
Pink	KEGG_PATHWAY	hsa04512:ECM-receptor interaction	16	1.2E-06	4.37	2.4E-04	2.4E-04	1.5E-03
Pink	SP_PIR_KEYWORDS	structural protein	15	5.9E-06	4.29	5.3E-03	3.1E-04	9.1E-03
Pink	SP_PIR_KEYWORDS	triple helix	9	6.8E-06	7.86	6.1E-03	3.4E-04	1.1E-02
Pink	INTERPRO	IPR013098:Immunoglobulin I-set	15	5.3E-07	5.14	2.4E-03	3.4E-04	1.0E-03
Pink	GOTERM_BP_ALL	GO:0032502~developmental process	144	1.4E-06	1.42	6.2E-03	5.2E-04	2.7E-03
Pink	INTERPRO	IPR000372:Leucine-rich repeat, cysteine-rich flanking region, N-terminal	12	1.0E-06	6.37	4.5E-03	5.7E-04	1.9E-03
Pink	GOTERM_CC_ALL	GO:0005615~extracellular space	27	9.8E-06	2.61	7.5E-03	6.3E-04	1.5E-02
Pink	SMART	SM00013:LRRNT	12	3.8E-06	5.54	2.0E-03	6.8E-04	5.5E-03
Pink	GOTERM_BP_ALL	GO:0006820~anion transport	18	2.2E-06	3.87	9.7E-03	7.5E-04	4.2E-03
Pink	SMART	SM00179:EGF_CA	16	7.6E-06	3.88	4.1E-03	1.0E-03	1.1E-02
Pink	KEGG_PATHWAY	hsa01430:Cell Communication	13	1.8E-05	4.35	3.6E-03	1.2E-03	2.3E-02
Pink	INTERPRO	IPR013151:Immunoglobulin	18	2.5E-06	3.85	1.1E-02	1.2E-03	4.7E-03
Pink	INTERPRO	IPR003961:Fibronectin, type III	18	3.0E-06	3.80	1.3E-02	1.4E-03	5.7E-03
Pink	GOTERM_BP_ALL	GO:0009653~anatomical structure morphogenesis	62	5.0E-06	1.80	2.2E-02	1.6E-03	9.4E-03
Pink	SP_PIR_KEYWORDS	sushi	9	3.5E-05	6.49	3.1E-02	1.7E-03	5.5E-02
Pink	GOTERM_BP_ALL	GO:0050776~regulation of immune response	14	6.1E-06	4.57	2.6E-02	1.8E-03	1.1E-02
Pink	SMART	SM00060:FN3	18	1.8E-05	3.30	9.4E-03	1.9E-03	2.6E-02
Pink	INTERPRO	IPR001881:EGF-like calcium-binding	15	5.1E-06	4.33	2.3E-02	2.1E-03	9.7E-03
Pink	GOTERM_BP_ALL	GO:0002682~regulation of immune system process	14	9.7E-06	4.40	4.1E-02	2.6E-03	1.8E-02
Pink	GOTERM_BP_ALL	GO:0006956~complement activation	9	1.2E-05	7.35	5.1E-02	2.9E-03	2.2E-02
Pink	GOTERM_BP_ALL	GO:0002541~activation of plasma proteins during acute inflammatory response	9	1.2E-05	7.35	5.1E-02	2.9E-03	2.2E-02
Pink	GOTERM_BP_ALL	GO:0048513~organ development	64	1.4E-05	1.72	6.0E-02	3.1E-03	2.7E-02

Pink	GOTERM_BP_ALL	GO:0002253~activation of immune response	12	1.4E-05	5.02	5.8E-02	3.1E-03	2.6E-02
Pink	SP_PIR_KEYWORDS	innate immunity	9	7.0E-05	5.97	6.1E-02	3.2E-03	1.1E-01
Pink	SP_PIR_KEYWORDS	acute phase	6	7.9E-05	11.06	6.9E-02	3.4E-03	1.2E-01
Pink	GOTERM_BP_ALL	GO:0006959~humoral immune response	11	1.7E-05	5.44	7.1E-02	3.5E-03	3.2E-02
Pink	GOTERM_BP_ALL	GO:0007166~cell surface receptor linked signal transduction	60	2.2E-05	1.74	9.0E-02	4.1E-03	4.1E-02
Pink	GOTERM_BP_ALL	GO:0009611~response to wounding	29	2.1E-05	2.40	8.8E-02	4.2E-03	4.0E-02
Pink	INTERPRO	IPR008957:Fibronectin, type III-like fold	17	1.1E-05	3.63	4.9E-02	4.2E-03	2.1E-02
Pink	INTERPRO	IPR013783:Immunoglobulin-like fold	25	1.4E-05	2.69	6.2E-02	4.9E-03	2.7E-02
Pink	INTERPRO	IPR008161:Collagen helix repeat	10	1.8E-05	6.10	7.7E-02	5.7E-03	3.4E-02
Pink	SP_PIR_KEYWORDS	complement pathway	7	1.4E-04	7.74	1.2E-01	5.9E-03	2.2E-01
Pink	SP_PIR_KEYWORDS	signal-anchor	24	1.5E-04	2.38	1.3E-01	6.0E-03	2.4E-01
Pink	SP_PIR_KEYWORDS	gamma-carboxyglutamic acid	5	1.8E-04	13.83	1.5E-01	6.3E-03	2.7E-01
Pink	SP_PIR_KEYWORDS	immune response	14	1.7E-04	3.42	1.4E-01	6.4E-03	2.6E-01
Pink	GOTERM_BP_ALL	GO:0050778~positive regulation of immune response	12	3.8E-05	4.56	1.5E-01	6.8E-03	7.1E-02
Pink	KEGG_PATHWAY	hsa04510:Focal adhesion	23	1.4E-04	2.37	2.7E-02	6.9E-03	1.7E-01
Pink	GOTERM_BP_ALL	GO:0002684~positive regulation of immune system process	12	4.7E-05	4.45	1.9E-01	8.2E-03	8.9E-02
Pink	INTERPRO	IPR013032:EGF-like region	22	2.8E-05	2.81	1.2E-01	8.3E-03	5.2E-02
Pink	GOTERM_BP_ALL	GO:0051240~positive regulation of multicellular organismal process	13	5.1E-05	4.08	2.0E-01	8.5E-03	9.5E-02
Pink	SMART	SM00032:CCP	9	9.9E-05	5.60	5.2E-02	8.9E-03	1.4E-01
Pink	GOTERM_MF_ALL	GO:0004888~transmembrane receptor activity	36	1.2E-05	2.21	2.7E-02	9.0E-03	2.1E-02
Pink	GOTERM_BP_ALL	GO:0051239~regulation of multicellular organismal process	21	6.1E-05	2.74	2.3E-01	9.7E-03	1.1E-01
Pink	SP_PIR_KEYWORDS	Zymogen	15	2.9E-04	3.07	2.3E-01	9.8E-03	4.6E-01
Pink	SP_PIR_KEYWORDS	pyrrolidone carboxylic acid	8	2.9E-04	5.77	2.3E-01	9.9E-03	4.5E-01
Pink	SMART	SM00209:TSP1	10	1.3E-04	4.77	7.0E-02	1.0E-02	1.9E-01
Pink	INTERPRO	IPR000436:Sushi/SCR/CCP	9	3.7E-05	6.44	1.5E-01	1.0E-02	7.0E-02
Pink	INTERPRO	IPR001314:Peptidase S1A, chymotrypsin	8	5.1E-05	7.32	2.0E-01	1.3E-02	9.6E-02
Pink	GOTERM_BP_ALL	GO:0006955~immune response	33	8.8E-05	2.09	3.2E-01	1.4E-02	1.6E-01
Pink	SP_PIR_KEYWORDS	Direct protein sequencing	111	4.3E-04	1.35	3.2E-01	1.4E-02	6.7E-01
Pink	INTERPRO	IPR000152:Aspartic acid and asparagine hydroxylation site	13	5.8E-05	4.04	2.3E-01	1.4E-02	1.1E-01
Pink	SP_PIR_KEYWORDS	Developmental protein	31	4.7E-04	1.96	3.5E-01	1.5E-02	7.4E-01
Pink	SMART	SM00082:LRRCT	9	2.6E-04	4.95	1.3E-01	1.8E-02	3.8E-01
Pink	KEGG_PATHWAY	hsa04640:Hematopoietic cell lineage	9	4.5E-04	4.57	8.5E-02	1.8E-02	5.6E-01
Pink	SMART	SM00069:GLA	5	3.1E-04	11.92	1.5E-01	1.8E-02	4.5E-01
Pink	SP_PIR_KEYWORDS	Proteoglycan	8	6.6E-04	5.10	4.5E-01	2.0E-02	1.0E+00
Pink	SP_PIR_KEYWORDS	ehlers-danlos syndrome	5	7.4E-04	10.37	4.9E-01	2.1E-02	1.2E+00
Pink	GOTERM_BP_ALL	GO:0002526~acute inflammatory response	10	1.4E-04	4.80	4.7E-01	2.1E-02	2.7E-01
Pink	GOTERM_BP_ALL	GO:0006958~complement activation, classical pathway	7	1.6E-04	7.62	4.9E-01	2.2E-02	2.9E-01
Pink	SP_PIR_KEYWORDS	serine proteinase	7	8.6E-04	5.81	5.4E-01	2.4E-02	1.3E+00
Pink	SP_PIR_KEYWORDS	tyrosine-specific protein kinase	9	9.0E-04	4.27	5.5E-01	2.4E-02	1.4E+00
Pink	SP_PIR_KEYWORDS	sulfation	6	9.8E-04	7.11	5.9E-01	2.6E-02	1.5E+00
Pink	INTERPRO	IPR000742:EGF-like, type 3	16	1.2E-04	3.17	4.1E-01	2.7E-02	2.2E-01
Pink	SMART	SM00020:Tryp_SPc	8	5.1E-04	5.20	2.4E-01	2.7E-02	7.4E-01
Pink	SP_PIR_KEYWORDS	serine protease	9	1.1E-03	4.15	6.3E-01	2.8E-02	1.7E+00
Pink	UP_SEQ_FEATURE	domain:EGF-like 3; calcium-binding	8	4.2E-05	7.46	3.6E-01	3.1E-02	8.5E-02
Pink	SP_PIR_KEYWORDS	transmembrane protein	29	1.3E-03	1.89	6.9E-01	3.2E-02	2.0E+00
Pink	GOTERM_BP_ALL	GO:0002455~humoral immune response mediated by circulating immunoglobulin	7	2.4E-04	7.14	6.4E-01	3.3E-02	4.4E-01
Pink	GOTERM_BP_ALL	GO:0006954~inflammatory response	21	2.5E-04	2.48	6.6E-01	3.3E-02	4.6E-01
Pink	INTERPRO	IPR006209:EGF-like	14	1.6E-04	3.44	5.1E-01	3.5E-02	2.9E-01
Pink	INTERPRO	IPR000884:Thrombospondin, type I	9	1.8E-04	5.29	5.6E-01	3.5E-02	3.4E-01
Pink	INTERPRO	IPR000294:Vitamin K-dependent carboxylation/gamma-carboxyglutamic region	5	1.8E-04	13.72	5.6E-01	3.6E-02	3.4E-01
Pink	INTERPRO	IPR006210:EGF	16	1.8E-04	3.06	5.5E-01	3.7E-02	3.3E-01
Pink	SMART	SM00409:IG	18	9.1E-04	2.43	3.9E-01	3.7E-02	1.3E+00
Pink	SMART	SM00643:C345C	6	8.4E-04	7.15	3.6E-01	3.7E-02	1.2E+00
Pink	SP_PIR_KEYWORDS	leucine-rich repeat	15	1.7E-03	2.59	7.8E-01	4.0E-02	2.6E+00
Pink	SMART	SM00181:EGF	16	8.4E-04	2.63	3.6E-01	4.0E-02	1.2E+00
Pink	INTERPRO	IPR001254:Peptidase S1 and S6, chymotrypsin/Hap	8	2.2E-04	5.99	6.3E-01	4.0E-02	4.1E-01
Pink	GOTERM_BP_ALL	GO:0001501~skeletal development	18	3.3E-04	2.67	7.6E-01	4.3E-02	6.2E-01
Pink	GOTERM_MF_ALL	GO:0004872~receptor activity	56	1.2E-04	1.68	2.5E-01	4.6E-02	2.2E-01
Pink	SP_PIR_KEYWORDS	beta-hydroxyasparagine	5	2.0E-03	8.30	8.4E-01	4.7E-02	3.1E+00

Pink	GOTERM_CC_ALL	GO:0005583~fibrillar collagen	5	8.2E-04	10.11	4.7E-01	4.7E-02	1.2E+00
Pink	SP_PIR_KEYWORDS	duplication	16	2.1E-03	2.44	8.5E-01	4.8E-02	3.2E+00
Magenta	SP_PIR_KEYWORDS	alternative splicing	240	6.4E-08	1.30	5.8E-05	5.8E-05	1.0E-04
Magenta	SP_PIR_KEYWORDS	phosphoprotein	236	1.5E-07	1.30	1.4E-04	6.9E-05	2.4E-04
Magenta	UP_SEQ_FEATURE	compositionally biased region:Pro-rich	38	1.6E-07	2.55	1.7E-03	1.7E-03	3.2E-04
Magenta	SP_PIR_KEYWORDS	Transcription regulation	85	2.7E-05	1.55	2.4E-02	4.8E-03	4.2E-02
Magenta	SP_PIR_KEYWORDS	cytoskeleton	31	2.7E-05	2.30	2.4E-02	6.0E-03	4.1E-02
Magenta	SP_PIR_KEYWORDS	Transcription	88	2.4E-05	1.54	2.1E-02	7.1E-03	3.7E-02
Magenta	SP_PIR_KEYWORDS	cytoplasm	128	1.7E-04	1.34	1.4E-01	2.2E-02	2.7E-01
Magenta	SP_PIR_KEYWORDS	nucleus	166	1.7E-04	1.27	1.4E-01	2.5E-02	2.6E-01
Purple	GOTERM_CC_ALL	GO:0005634~nucleus	183	2.1E-07	1.35	1.6E-04	1.6E-04	3.2E-04
Purple	SP_PIR_KEYWORDS	alternative splicing	215	1.5E-06	1.28	1.4E-03	1.4E-03	2.4E-03
Purple	SP_PIR_KEYWORDS	nucleus	158	1.8E-05	1.33	1.6E-02	8.1E-03	2.8E-02
Purple	GOTERM_BP_ALL	GO:0006139~nucleobase, nucleoside, nucleotide and nucleic acid metabolic process	157	1.9E-06	1.37	8.2E-03	8.2E-03	3.6E-03
Purple	GOTERM_BP_ALL	GO:0043283~biopolymer metabolic process	195	2.1E-05	1.25	8.7E-02	3.0E-02	3.9E-02
Purple	GOTERM_BP_ALL	GO:0006259~DNA metabolic process	47	1.5E-05	1.93	6.4E-02	3.3E-02	2.9E-02
Purple	GOTERM_BP_ALL	GO:0016070~RNA metabolic process	122	3.7E-05	1.38	1.5E-01	3.9E-02	6.9E-02
Greenyellow	GOTERM_BP_ALL	GO:0050789~regulation of biological process	164	5.7E-10	1.47	2.5E-06	1.2E-06	1.1E-06
Greenyellow	GOTERM_BP_ALL	GO:0050794~regulation of cellular process	157	3.9E-10	1.50	1.7E-06	1.7E-06	7.3E-07
Greenyellow	GOTERM_BP_ALL	GO:0065007~biological regulation	172	1.9E-09	1.42	8.1E-06	2.7E-06	3.5E-06
Greenyellow	SP_PIR_KEYWORDS	phosphoprotein	183	2.1E-08	1.38	1.9E-05	9.6E-06	3.3E-05
Greenyellow	SP_PIR_KEYWORDS	zinc-finger	73	1.1E-08	1.97	1.0E-05	1.0E-05	1.8E-05
Greenyellow	SP_PIR_KEYWORDS	alternative splicing	182	1.1E-07	1.35	1.0E-04	3.3E-05	1.7E-04
Greenyellow	SP_PIR_KEYWORDS	Transcription regulation	73	2.8E-07	1.82	2.5E-04	5.1E-05	4.4E-04
Greenyellow	SP_PIR_KEYWORDS	zinc	80	2.6E-07	1.76	2.4E-04	5.9E-05	4.1E-04
Greenyellow	SP_PIR_KEYWORDS	chromosomal rearrangement	23	8.7E-07	3.39	7.9E-04	9.8E-05	1.4E-03
Greenyellow	SP_PIR_KEYWORDS	Transcription	74	6.8E-07	1.77	6.1E-04	1.0E-04	1.1E-03
Greenyellow	SP_PIR_KEYWORDS	metal-binding	96	8.0E-07	1.61	7.2E-04	1.0E-04	1.2E-03
Greenyellow	SP_PIR_KEYWORDS	nucleus	134	3.5E-06	1.41	3.2E-03	3.2E-04	5.5E-03
Greenyellow	SP_PIR_KEYWORDS	dna-binding	62	3.4E-06	1.82	3.1E-03	3.4E-04	5.4E-03
Greenyellow	GOTERM_CC_ALL	GO:0005634~nucleus	159	9.4E-07	1.36	7.2E-04	7.2E-04	1.4E-03
Greenyellow	GOTERM_MF_ALL	GO:0008270~zinc ion binding	90	4.2E-07	1.66	9.5E-04	9.5E-04	7.3E-04
Greenyellow	GOTERM_BP_ALL	GO:0032774~RNA biosynthetic process	88	3.0E-06	1.59	1.3E-02	2.2E-03	5.6E-03
Greenyellow	GOTERM_BP_ALL	GO:0006351~transcription, DNA-dependent	88	2.9E-06	1.59	1.3E-02	2.5E-03	5.5E-03
Greenyellow	SP_PIR_KEYWORDS	activator	28	3.2E-05	2.42	2.8E-02	2.6E-03	4.9E-02
Greenyellow	GOTERM_BP_ALL	GO:0006355~regulation of transcription, DNA-dependent	86	2.5E-06	1.61	1.1E-02	2.7E-03	4.7E-03
Greenyellow	SP_PIR_KEYWORDS	proto-oncogene	19	4.7E-05	3.03	4.1E-02	3.5E-03	7.3E-02
Greenyellow	GOTERM_MF_ALL	GO:0046914~transition metal ion binding	100	3.5E-06	1.53	8.0E-03	4.0E-03	6.2E-03
Greenyellow	GOTERM_BP_ALL	GO:0043283~biopolymer metabolic process	167	7.7E-06	1.29	3.3E-02	4.7E-03	1.4E-02
Greenyellow	GOTERM_BP_ALL	GO:0031323~regulation of cellular metabolic process	97	9.9E-06	1.50	4.2E-02	5.4E-03	1.9E-02
Greenyellow	GOTERM_BP_ALL	GO:0019222~regulation of metabolic process	100	1.2E-05	1.48	4.9E-02	5.6E-03	2.2E-02
Greenyellow	GOTERM_MF_ALL	GO:0003677~DNA binding	82	7.4E-06	1.60	1.7E-02	5.6E-03	1.3E-02
Greenyellow	GOTERM_BP_ALL	GO:0045449~regulation of transcription	88	1.3E-05	1.54	5.7E-02	5.8E-03	2.5E-02
Greenyellow	GOTERM_MF_ALL	GO:0043167~ion binding	131	1.1E-05	1.38	2.5E-02	6.2E-03	1.9E-02
Greenyellow	GOTERM_BP_ALL	GO:0006350~transcription	91	1.8E-05	1.51	7.5E-02	7.1E-03	3.4E-02
Greenyellow	GOTERM_MF_ALL	GO:0030528~transcription regulator activity	61	1.6E-05	1.74	3.7E-02	7.4E-03	2.9E-02
Greenyellow	GOTERM_BP_ALL	GO:0010468~regulation of gene expression	92	2.4E-05	1.49	9.8E-02	7.9E-03	4.4E-02
Greenyellow	GOTERM_MF_ALL	GO:0043169~cation binding	118	2.5E-05	1.40	5.4E-02	7.9E-03	4.3E-02
Greenyellow	GOTERM_MF_ALL	GO:0046872~metal ion binding	128	2.2E-05	1.38	4.8E-02	8.2E-03	3.8E-02
Greenyellow	GOTERM_BP_ALL	GO:0019219~regulation of nucleobase, nucleoside, nucleotide and nucleic acid metabolic process	89	2.4E-05	1.51	9.7E-02	8.5E-03	4.4E-02
Greenyellow	GOTERM_MF_ALL	GO:0003676~nucleic acid binding	114	3.7E-05	1.40	8.0E-02	1.0E-02	6.4E-02
Greenyellow	GOTERM_BP_ALL	GO:0043687~post-translational protein modification	66	6.1E-05	1.61	2.3E-01	1.8E-02	1.2E-01
Greenyellow	GOTERM_BP_ALL	GO:0009893~positive regulation of metabolic process	27	6.0E-05	2.37	2.3E-01	1.8E-02	1.1E-01
Greenyellow	GOTERM_MF_ALL	GO:0005488~binding	311	8.5E-05	1.08	1.8E-01	2.1E-02	1.5E-01
Greenyellow	GOTERM_MF_ALL	GO:0005515~protein binding	223	1.1E-04	1.18	2.3E-01	2.6E-02	2.0E-01
Greenyellow	GOTERM_MF_ALL	GO:0030234~enzyme regulator activity	39	1.4E-04	1.90	2.7E-01	2.8E-02	2.4E-01
Greenyellow	GOTERM_BP_ALL	GO:0016070~RNA metabolic process	102	1.2E-04	1.40	4.0E-01	3.1E-02	2.2E-01
Tan	GOTERM_BP_ALL	GO:0044255~cellular lipid metabolic process	33	1.0E-05	2.33	4.3E-02	2.2E-02	1.9E-02
Tan	GOTERM_BP_ALL	GO:0006629~lipid metabolic process	37	9.2E-06	2.20	3.9E-02	3.9E-02	1.7E-02

Salmon	SP_PIR_KEYWORDS	signal	74	4.6E-08	1.88	4.1E-05	4.1E-05	7.1E-05
Salmon	SP_PIR_KEYWORDS	glycoprotein	83	1.7E-07	1.74	1.6E-04	7.8E-05	2.7E-04
Salmon	UP_SEQ_FEATURE	topological domain:Extracellular	51	2.7E-07	2.11	2.9E-03	1.4E-03	5.5E-04
Salmon	UP_SEQ_FEATURE	glycosylation site:N-linked (GlcNAc...)	71	6.3E-07	1.77	6.7E-03	2.3E-03	1.3E-03
Salmon	UP_SEQ_FEATURE	signal peptide	67	2.2E-07	1.86	2.4E-03	2.4E-03	4.6E-04
Salmon	GOTERM_MF_ALL	GO:0004872~receptor activity	46	1.6E-06	2.12	3.6E-03	3.6E-03	2.8E-03
Salmon	GOTERM_MF_ALL	GO:0004871~signal transducer activity	57	1.2E-05	1.79	2.7E-02	1.3E-02	2.1E-02
Salmon	GOTERM_MF_ALL	GO:0060089~molecular transducer activity	57	1.2E-05	1.79	2.7E-02	1.3E-02	2.1E-02
Salmon	GOTERM_BP_ALL	GO:0032502~developmental process	96	3.9E-06	1.52	1.7E-02	1.7E-02	7.4E-03
Salmon	UP_SEQ_FEATURE	topological domain:Cytoplasmic	60	9.5E-06	1.75	9.7E-02	2.5E-02	1.9E-02
Salmon	GOTERM_BP_ALL	GO:0007165~signal transduction	91	2.1E-05	1.49	8.9E-02	4.5E-02	4.0E-02
Cyan	UP_SEQ_FEATURE	topological domain:Extracellular	49	2.2E-10	2.64	2.4E-06	2.4E-06	4.6E-07
Cyan	GOTERM_CC_ALL	GO:0044459~plasma membrane part	49	5.2E-09	2.43	4.0E-06	4.0E-06	7.9E-06
Cyan	UP_SEQ_FEATURE	glycosylation site:N-linked (GlcNAc...)	65	1.3E-09	2.11	1.4E-05	4.6E-06	2.7E-06
Cyan	UP_SEQ_FEATURE	topological domain:Cytoplasmic	59	1.1E-09	2.24	1.1E-05	5.7E-06	2.2E-06
Cyan	SP_PIR_KEYWORDS	glycoprotein	71	1.1E-08	1.95	9.5E-06	9.5E-06	1.6E-05
Cyan	GOTERM_CC_ALL	GO:0005886~plasma membrane	62	6.5E-08	1.97	5.0E-05	2.5E-05	1.0E-04
Cyan	SP_PIR_KEYWORDS	signal	60	1.0E-07	2.00	9.4E-05	4.7E-05	1.6E-04
Cyan	UP_SEQ_FEATURE	signal peptide	58	1.9E-08	2.10	2.1E-04	5.2E-05	4.0E-05
Cyan	GOTERM_BP_ALL	GO:0032501~multicellular organismal process	72	1.3E-08	1.89	5.5E-05	5.5E-05	2.4E-05
Cyan	GOTERM_CC_ALL	GO:0031226~intrinsic to plasma membrane	33	1.6E-06	2.53	1.2E-03	3.0E-04	2.4E-03
Cyan	GOTERM_CC_ALL	GO:0005887~integral to plasma membrane	33	1.3E-06	2.55	9.9E-04	3.3E-04	2.0E-03
Cyan	GOTERM_BP_ALL	GO:0007275~multicellular organismal development	57	6.1E-07	1.92	2.6E-03	8.8E-04	1.1E-03
Cyan	GOTERM_BP_ALL	GO:0022610~biological adhesion	28	1.1E-06	2.86	4.9E-03	9.9E-04	2.1E-03
Cyan	GOTERM_BP_ALL	GO:0007155~cell adhesion	28	1.1E-06	2.86	4.9E-03	9.9E-04	2.1E-03
Cyan	GOTERM_BP_ALL	GO:0048513~organ development	39	6.1E-07	2.36	2.6E-03	1.3E-03	1.1E-03
Cyan	INTERPRO	IPR006703:AIG1	6	3.8E-07	29.61	1.7E-03	1.7E-03	7.2E-04
Cyan	GOTERM_CC_ALL	GO:0005856~cytoskeleton	34	1.2E-05	2.26	8.8E-03	1.8E-03	1.8E-02
Cyan	GOTERM_CC_ALL	GO:0016020~membrane	115	1.5E-05	1.36	1.1E-02	1.9E-03	2.2E-02
Cyan	SP_PIR_KEYWORDS	transmembrane	75	7.0E-06	1.62	6.3E-03	2.1E-03	1.1E-02
Cyan	GOTERM_CC_ALL	GO:0016021~integral to membrane	83	2.3E-05	1.50	1.8E-02	2.2E-03	3.5E-02
Cyan	GOTERM_CC_ALL	GO:0031224~intrinsic to membrane	83	2.6E-05	1.49	2.0E-02	2.2E-03	4.0E-02
Cyan	GOTERM_CC_ALL	GO:0044425~membrane part	96	2.1E-05	1.43	1.6E-02	2.3E-03	3.2E-02
Cyan	GOTERM_BP_ALL	GO:0048731~system development	45	4.1E-06	2.03	1.8E-02	2.9E-03	7.6E-03
Cyan	SP_PIR_KEYWORDS	muscle	6	1.3E-05	17.13	1.2E-02	3.0E-03	2.1E-02
Cyan	SP_PIR_KEYWORDS	actin-binding	15	2.3E-05	3.95	2.0E-02	4.1E-03	3.5E-02
Cyan	SP_PIR_KEYWORDS	Muscle protein	6	3.2E-05	14.68	2.9E-02	4.8E-03	5.0E-02
Cyan	UP_SEQ_FEATURE	transmembrane region	65	2.5E-06	1.74	2.6E-02	5.3E-03	5.0E-03
Cyan	GOTERM_BP_ALL	GO:0007517~muscle development	13	1.0E-05	4.92	4.4E-02	6.4E-03	1.9E-02
Cyan	GOTERM_CC_ALL	GO:0030054~cell junction	15	8.9E-05	3.49	6.6E-02	6.8E-03	1.4E-01
Cyan	SP_PIR_KEYWORDS	cell junction	13	6.5E-05	4.12	5.7E-02	8.3E-03	1.0E-01
Cyan	SP_PIR_KEYWORDS	cell adhesion	17	8.4E-05	3.17	7.3E-02	9.5E-03	1.3E-01
Cyan	GOTERM_BP_ALL	GO:0007154~cell communication	74	1.8E-05	1.56	7.5E-02	9.6E-03	3.3E-02
Cyan	GOTERM_CC_ALL	GO:0030016~myofibril	6	2.2E-04	10.24	1.6E-01	1.5E-02	3.4E-01
Cyan	GOTERM_BP_ALL	GO:0032502~developmental process	70	4.4E-05	1.55	1.7E-01	1.9E-02	8.2E-02
Cyan	GOTERM_BP_ALL	GO:0048856~anatomical structure development	50	4.1E-05	1.77	1.6E-01	2.0E-02	7.7E-02
Cyan	GOTERM_BP_ALL	GO:0007165~signal transduction	68	5.6E-05	1.56	2.2E-01	2.2E-02	1.1E-01
Cyan	GOTERM_CC_ALL	GO:0015629~actin cytoskeleton	14	4.5E-04	3.15	2.9E-01	2.6E-02	6.9E-01
Cyan	GOTERM_BP_ALL	GO:0006928~cell motility	18	8.8E-05	3.01	3.2E-01	2.7E-02	1.6E-01
Cyan	GOTERM_BP_ALL	GO:0051674~localization of cell	18	8.8E-05	3.01	3.2E-01	2.7E-02	1.6E-01
Cyan	GOTERM_CC_ALL	GO:0044449~contractile fiber part	6	4.3E-04	8.96	2.8E-01	2.7E-02	6.6E-01
Cyan	GOTERM_BP_ALL	GO:0016477~cell migration	14	8.4E-05	3.73	3.1E-01	3.0E-02	1.6E-01
Cyan	GOTERM_MF_ALL	GO:0060089~molecular transducer activity	44	3.0E-05	1.91	6.6E-02	3.4E-02	5.3E-02
Cyan	GOTERM_MF_ALL	GO:0004871~signal transducer activity	44	3.0E-05	1.91	6.6E-02	3.4E-02	5.3E-02
Cyan	GOTERM_CC_ALL	GO:0043292~contractile fiber	6	6.4E-04	8.27	3.9E-01	3.5E-02	9.8E-01
Cyan	GOTERM_CC_ALL	GO:0030017~sarcomere	5	8.1E-04	11.20	4.6E-01	4.1E-02	1.2E+00
Midnightblue	GOTERM_CC_ALL	GO:0005773~vacuole	27	2.1E-13	5.98	1.6E-10	5.5E-11	3.3E-10
Midnightblue	SP_PIR_KEYWORDS	lysosome	22	6.4E-14	8.40	5.8E-11	5.8E-11	1.0E-10
Midnightblue	GOTERM_CC_ALL	GO:0005764~lysosome	26	1.3E-13	6.40	9.9E-11	9.9E-11	2.0E-10

Midnightblue	GOTERM_CC_ALL	GO:0000323~lytic vacuole	26	1.3E-13	6.40	9.9E-11	9.9E-11	2.0E-10
Midnightblue	SP_PIR_KEYWORDS	glycoprotein	78	2.2E-13	2.29	2.0E-10	1.0E-10	3.5E-10
Midnightblue	GOTERM_BP_ALL	GO:0002376~immune system process	42	8.7E-14	3.79	3.8E-10	3.8E-10	1.6E-10
Midnightblue	SP_PIR_KEYWORDS	signal	67	4.1E-12	2.39	3.7E-09	1.2E-09	6.4E-09
Midnightblue	GOTERM_BP_ALL	GO:0006955~immune response	33	1.1E-12	4.44	4.8E-09	2.4E-09	2.1E-09
Midnightblue	GOTERM_CC_ALL	GO:0044459~plasma membrane part	53	1.3E-10	2.56	1.0E-07	2.6E-08	2.1E-07
Midnightblue	UP_SEQ_FEATURE	glycosylation site:N-linked (GlcNAc...)	63	2.4E-10	2.21	2.6E-06	2.6E-06	5.0E-07
Midnightblue	GOTERM_CC_ALL	GO:0031226~intrinsic to plasma membrane	37	3.0E-08	2.76	2.3E-05	3.3E-06	4.5E-05
Midnightblue	GOTERM_CC_ALL	GO:0005886~plasma membrane	64	2.8E-08	1.99	2.2E-05	3.6E-06	4.3E-05
Midnightblue	GOTERM_CC_ALL	GO:0005887~integral to plasma membrane	37	2.4E-08	2.79	1.8E-05	3.6E-06	3.6E-05
Midnightblue	GOTERM_CC_ALL	GO:0016020~membrane	124	1.6E-07	1.43	1.3E-04	1.6E-05	2.5E-04
Midnightblue	UP_SEQ_FEATURE	disulfide bond	47	7.7E-09	2.43	8.3E-05	2.8E-05	1.6E-05
Midnightblue	UP_SEQ_FEATURE	signal peptide	56	5.8E-09	2.20	6.3E-05	3.1E-05	1.2E-05
Midnightblue	INTERPRO	IPR013106:Immunoglobulin V-set	12	1.3E-08	10.20	5.8E-05	5.8E-05	2.4E-05
Midnightblue	GOTERM_BP_ALL	GO:0050896~response to stimulus	61	6.5E-08	1.99	2.8E-04	9.3E-05	1.2E-04
Midnightblue	GOTERM_CC_ALL	GO:0044425~membrane part	100	5.8E-06	1.45	4.4E-03	4.9E-04	8.8E-03
Midnightblue	SP_PIR_KEYWORDS	Direct protein sequencing	65	3.0E-06	1.75	2.7E-03	6.8E-04	4.7E-03
Midnightblue	GOTERM_CC_ALL	GO:0005576~extracellular region	29	1.0E-05	2.50	7.9E-03	7.9E-04	1.6E-02
Midnightblue	SP_PIR_KEYWORDS	immunoglobulin domain	16	7.0E-06	4.10	6.3E-03	1.1E-03	1.1E-02
Midnightblue	SP_PIR_KEYWORDS	membrane	91	6.3E-06	1.51	5.7E-03	1.1E-03	9.9E-03
Midnightblue	GOTERM_BP_ALL	GO:0042330~taxis	12	1.4E-06	6.61	5.9E-03	1.2E-03	2.6E-03
Midnightblue	GOTERM_BP_ALL	GO:0006935~chemotaxis	12	1.4E-06	6.61	5.9E-03	1.2E-03	2.6E-03
Midnightblue	SP_PIR_KEYWORDS	transmembrane protein	21	1.7E-05	3.03	1.5E-02	2.2E-03	2.7E-02
Midnightblue	GOTERM_CC_ALL	GO:0031224~intrinsic to membrane	84	3.9E-05	1.48	3.0E-02	2.7E-03	6.0E-02
Midnightblue	GOTERM_BP_ALL	GO:0009605~response to external stimulus	24	4.7E-06	2.96	2.0E-02	3.4E-03	8.8E-03
Midnightblue	GOTERM_CC_ALL	GO:0016021~integral to membrane	83	6.4E-05	1.46	4.8E-02	4.1E-03	9.8E-02
Midnightblue	SP_PIR_KEYWORDS	sh2 domain	10	4.5E-05	5.82	4.0E-02	5.1E-03	7.0E-02
Midnightblue	GOTERM_BP_ALL	GO:0007165~signal transduction	74	8.4E-06	1.60	3.6E-02	5.2E-03	1.6E-02
Midnightblue	GOTERM_BP_ALL	GO:0006952~defense response	20	1.0E-05	3.24	4.4E-02	5.7E-03	2.0E-02
Midnightblue	GOTERM_BP_ALL	GO:0045321~leukocyte activation	13	1.2E-05	4.85	5.1E-02	5.8E-03	2.2E-02
Midnightblue	GOTERM_BP_ALL	GO:0002443~leukocyte mediated immunity	9	1.4E-05	7.81	5.8E-02	6.0E-03	2.6E-02
Midnightblue	SP_PIR_KEYWORDS	Zymogen	11	6.2E-05	4.98	5.4E-02	6.2E-03	9.6E-02
Midnightblue	KEGG_PATHWAY	hsa04650:Natural killer cell mediated cytotoxicity	11	3.2E-05	5.10	6.3E-03	6.3E-03	4.0E-02
Midnightblue	GOTERM_BP_ALL	GO:0002252~immune effector process	10	1.9E-05	6.43	7.9E-02	7.5E-03	3.6E-02
Midnightblue	GOTERM_BP_ALL	GO:0007626~locomotory behavior	12	2.2E-05	5.02	9.0E-02	7.8E-03	4.0E-02
Midnightblue	GOTERM_BP_ALL	GO:0001775~cell activation	13	2.5E-05	4.51	1.0E-01	7.8E-03	4.8E-02
Midnightblue	GOTERM_BP_ALL	GO:0007154~cell communication	77	2.4E-05	1.53	1.0E-01	8.1E-03	4.5E-02
Midnightblue	GOTERM_BP_ALL	GO:0007610~behavior	14	2.9E-05	4.12	1.2E-01	8.5E-03	5.5E-02
Midnightblue	SMART	SM00407:IGc1	7	1.9E-05	11.49	1.0E-02	1.0E-02	2.8E-02
Midnightblue	INTERPRO	IPR007110:Immunoglobulin-like	16	5.0E-06	4.21	2.2E-02	1.1E-02	9.3E-03
Midnightblue	SMART	SM00252:SH2	10	6.5E-05	5.47	3.5E-02	1.7E-02	9.4E-02
Midnightblue	GOTERM_CC_ALL	GO:0009986~cell surface	10	3.1E-04	4.54	2.1E-01	1.8E-02	4.7E-01
Midnightblue	INTERPRO	IPR003597:Immunoglobulin C1-set	7	1.3E-05	12.46	5.6E-02	1.9E-02	2.4E-02
Midnightblue	GOTERM_CC_ALL	GO:0005774~vacuolar membrane	8	4.3E-04	5.71	2.8E-01	2.3E-02	6.5E-01
Midnightblue	GOTERM_CC_ALL	GO:0044437~vacuolar part	8	4.3E-04	5.71	2.8E-01	2.3E-02	6.5E-01
Midnightblue	GOTERM_BP_ALL	GO:0002682~regulation of immune system process	9	1.0E-04	6.01	3.5E-01	2.7E-02	1.9E-01
Midnightblue	SP_PIR_KEYWORDS	immunoglobulin	4	3.7E-04	24.43	2.9E-01	3.0E-02	5.8E-01
Midnightblue	GOTERM_BP_ALL	GO:0042221~response to chemical stimulus	22	1.2E-04	2.55	4.1E-01	3.0E-02	2.2E-01
Midnightblue	SP_PIR_KEYWORDS	SH3 domain	12	3.7E-04	3.69	2.8E-01	3.3E-02	5.8E-01
Midnightblue	GOTERM_CC_ALL	GO:0009897~external side of plasma membrane	6	8.6E-04	7.77	4.8E-01	4.0E-02	1.3E+00
Midnightblue	INTERPRO	IPR000980:SH2 motif	10	3.8E-05	5.94	1.6E-01	4.2E-02	7.2E-02
Midnightblue	INTERPRO	IPR013783:Immunoglobulin-like fold	15	5.3E-05	3.67	2.1E-01	4.6E-02	9.9E-02
Midnightblue	SP_PIR_KEYWORDS	transmembrane	64	6.4E-04	1.48	4.4E-01	4.7E-02	9.9E-01
Lightcyan	UP_SEQ_FEATURE	domain:RRM 2	10	4.7E-06	7.58	5.0E-02	2.5E-02	9.7E-03
Lightcyan	UP_SEQ_FEATURE	domain:RRM 1	10	4.7E-06	7.58	5.0E-02	2.5E-02	9.7E-03
Lightcyan	GOTERM_BP_ALL	GO:0043170~macromolecule metabolic process	133	5.9E-06	1.29	2.5E-02	2.5E-02	1.1E-02
Lightcyan	SP_PIR_KEYWORDS	mrna splicing	15	3.3E-05	3.82	3.0E-02	3.0E-02	5.2E-02
Lightcyan	GOTERM_BP_ALL	GO:0006396~RNA processing	25	2.2E-05	2.62	9.1E-02	4.6E-02	4.1E-02
Grey60	SP_PIR_KEYWORDS	nucleus	83	2.2E-05	1.50	2.0E-02	2.0E-02	3.5E-02

Grey60	GOTERM_CC_ALL	GO:0005634~nucleus	91	5.5E-05	1.40	4.1E-02	4.1E-02	8.4E-02
Lightgreen	SP_PIR_KEYWORDS	Direct protein sequencing	56	8.8E-07	1.90	7.9E-04	7.9E-04	1.4E-03
Lightyellow	SP_PIR_KEYWORDS	signal	75	4.5E-24	3.44	4.1E-21	4.1E-21	7.0E-21
Lightyellow	SP_PIR_KEYWORDS	glycoprotein	78	6.1E-21	2.95	5.5E-18	2.8E-18	9.6E-18
Lightyellow	UP_SEQ_FEATURE	signal peptide	65	1.7E-18	3.09	1.8E-14	1.8E-14	3.4E-15
Lightyellow	GOTERM_BP_ALL	GO:0009605~response to external stimulus	36	4.1E-17	5.57	1.8E-13	8.9E-14	7.7E-14
Lightyellow	GOTERM_BP_ALL	GO:0009611~response to wounding	31	3.6E-17	6.84	1.5E-13	1.5E-13	6.7E-14
Lightyellow	GOTERM_BP_ALL	GO:0006954~inflammatory response	25	3.5E-15	7.87	1.5E-11	5.1E-12	6.7E-12
Lightyellow	SP_PIR_KEYWORDS	Secreted	38	5.2E-14	4.23	4.7E-11	1.6E-11	8.2E-11
Lightyellow	GOTERM_BP_ALL	GO:0006952~defense response	29	2.8E-14	5.89	1.2E-10	3.0E-11	5.2E-11
Lightyellow	GOTERM_BP_ALL	GO:0050896~response to stimulus	64	4.8E-14	2.61	2.1E-10	4.2E-11	9.0E-11
Lightyellow	UP_SEQ_FEATURE	glycosylation site:N-linked (GlcNAc...)	62	4.5E-14	2.64	4.9E-10	2.4E-10	9.3E-11
Lightyellow	GOTERM_CC_ALL	GO:0005576~extracellular region	37	6.9E-13	3.99	5.3E-10	5.3E-10	1.1E-09
Lightyellow	UP_SEQ_FEATURE	disulfide bond	49	3.6E-13	3.07	3.9E-09	1.3E-09	7.5E-10
Lightyellow	GOTERM_BP_ALL	GO:0002376~immune system process	35	4.1E-12	3.95	1.8E-08	3.0E-09	7.8E-09
Lightyellow	GOTERM_BP_ALL	GO:0006955~immune response	28	1.9E-11	4.71	8.3E-08	1.2E-08	3.6E-08
Lightyellow	SP_PIR_KEYWORDS	immune response	14	1.2E-09	9.70	1.1E-06	2.7E-07	1.8E-06
Lightyellow	GOTERM_MF_ALL	GO:0004872~receptor activity	37	1.5E-09	3.05	3.5E-06	3.5E-06	2.7E-06
Lightyellow	GOTERM_MF_ALL	GO:0004888~transmembrane receptor activity	24	1.6E-08	4.06	3.6E-05	9.0E-06	2.7E-05
Lightyellow	GOTERM_MF_ALL	GO:0060089~molecular transducer activity	44	1.5E-08	2.48	3.3E-05	1.7E-05	2.5E-05
Lightyellow	GOTERM_MF_ALL	GO:0004871~signal transducer activity	44	1.5E-08	2.48	3.3E-05	1.7E-05	2.5E-05
Lightyellow	SP_PIR_KEYWORDS	inflammatory response	8	1.0E-07	18.84	9.1E-05	1.8E-05	1.6E-04
Lightyellow	GOTERM_BP_ALL	GO:0006950~response to stress	37	4.3E-08	2.68	1.9E-04	2.3E-05	8.0E-05
Lightyellow	SP_PIR_KEYWORDS	membrane	78	3.4E-07	1.66	3.1E-04	5.1E-05	5.3E-04
Lightyellow	GOTERM_CC_ALL	GO:0005886~plasma membrane	52	3.7E-07	2.02	2.8E-04	9.5E-05	5.7E-04
Lightyellow	GOTERM_CC_ALL	GO:0016020~membrane	102	3.2E-07	1.47	2.4E-04	1.2E-04	4.8E-04
Lightyellow	GOTERM_CC_ALL	GO:0044421~extracellular region part	23	6.5E-07	3.44	5.0E-04	1.3E-04	1.0E-03
Lightyellow	SP_PIR_KEYWORDS	transmembrane	59	5.2E-06	1.75	4.7E-03	6.7E-04	8.1E-03
Lightyellow	SP_PIR_KEYWORDS	Direct protein sequencing	53	6.1E-06	1.83	5.5E-03	6.9E-04	9.6E-03
Lightyellow	GOTERM_BP_ALL	GO:0045087~innate immune response	9	2.6E-06	9.77	1.1E-02	1.3E-03	4.9E-03
Lightyellow	GOTERM_CC_ALL	GO:0044425~membrane part	82	1.3E-05	1.49	9.8E-03	2.0E-03	2.0E-02
Lightyellow	GOTERM_CC_ALL	GO:0005764~lysosome	14	1.8E-05	4.32	1.4E-02	2.0E-03	2.8E-02
Lightyellow	GOTERM_CC_ALL	GO:0000323~lytic vacuole	14	1.8E-05	4.32	1.4E-02	2.0E-03	2.8E-02
Lightyellow	GOTERM_CC_ALL	GO:0005615~extracellular space	15	2.4E-05	3.93	1.8E-02	2.3E-03	3.6E-02
Lightyellow	SP_PIR_KEYWORDS	receptor	23	3.3E-05	2.70	2.9E-02	3.0E-03	5.2E-02
Lightyellow	SP_PIR_KEYWORDS	lysosome	11	3.2E-05	5.40	2.8E-02	3.2E-03	4.9E-02
Lightyellow	SP_PIR_KEYWORDS	plasma	7	4.6E-05	10.30	4.1E-02	3.8E-03	7.2E-02
Lightyellow	GOTERM_CC_ALL	GO:0005773~vacuole	14	5.7E-05	3.88	4.3E-02	4.8E-03	8.7E-02
Lightyellow	GOTERM_CC_ALL	GO:0044459~plasma membrane part	34	6.5E-05	2.06	4.9E-02	5.0E-03	9.9E-02
Lightyellow	GOTERM_CC_ALL	GO:0031224~intrinsic to membrane	69	7.5E-05	1.52	5.6E-02	5.2E-03	1.1E-01
Lightyellow	GOTERM_BP_ALL	GO:0007610~behavior	13	1.4E-05	4.79	6.0E-02	6.2E-03	2.7E-02
Lightyellow	GOTERM_CC_ALL	GO:0016021~integral to membrane	68	1.3E-04	1.50	9.5E-02	8.3E-03	2.0E-01
Lightyellow	SP_PIR_KEYWORDS	innate immunity	6	1.5E-04	11.31	1.3E-01	1.1E-02	2.3E-01
Lightyellow	GOTERM_BP_ALL	GO:0050778~positive regulation of immune response	8	4.6E-05	8.08	1.8E-01	1.8E-02	8.7E-02
Lightyellow	GOTERM_BP_ALL	GO:0002684~positive regulation of immune system process	8	5.4E-05	7.90	2.1E-01	1.9E-02	1.0E-01
Lightyellow	GOTERM_CC_ALL	GO:0031226~intrinsic to plasma membrane	24	3.4E-04	2.24	2.3E-01	2.0E-02	5.2E-01
Lightyellow	UP_SEQ_FEATURE	sequence variant	87	8.2E-06	1.44	8.4E-02	2.2E-02	1.7E-02
Lightyellow	GOTERM_BP_ALL	GO:0007626~locomotory behavior	10	1.1E-04	5.23	3.7E-01	3.2E-02	2.0E-01
Lightyellow	GOTERM_BP_ALL	GO:0050776~regulation of immune response	8	1.3E-04	6.95	4.2E-01	3.3E-02	2.3E-01
Lightyellow	GOTERM_BP_ALL	GO:0002526~acute inflammatory response	7	1.0E-04	8.94	3.6E-01	3.4E-02	1.9E-01
Lightyellow	GOTERM_BP_ALL	GO:0007154~cell communication	62	1.2E-04	1.54	4.2E-01	3.5E-02	2.3E-01
Lightyellow	GOTERM_BP_ALL	GO:0002682~regulation of immune system process	8	1.6E-04	6.68	5.0E-01	3.8E-02	3.0E-01
Lightyellow	GOTERM_BP_ALL	GO:0051240~positive regulation of multicellular organismal process	8	1.6E-04	6.68	5.0E-01	3.8E-02	3.0E-01
Lightyellow	SP_PIR_KEYWORDS	hydroxyproline	5	5.8E-04	12.40	4.1E-01	3.9E-02	9.0E-01
Lightyellow	GOTERM_CC_ALL	GO:0005887~integral to plasma membrane	23	7.6E-04	2.17	4.4E-01	4.1E-02	1.2E+00
Lightyellow	SP_PIR_KEYWORDS	heterodimer	7	6.6E-04	6.47	4.5E-01	4.2E-02	1.0E+00
Lightyellow	GOTERM_MF_ALL	GO:0001871~pattern binding	8	1.0E-04	7.19	2.1E-01	4.6E-02	1.8E-01
Royalblue	KEGG_PATHWAY	hsa03010:Ribosome	35	1.2E-48	27.84	2.3E-46	2.3E-46	1.4E-45
Royalblue	GOTERM_MF_ALL	GO:0003735~structural constituent of ribosome	50	1.3E-49	17.38	3.0E-46	3.0E-46	2.3E-46

Royalblue	GOTERM_CC_ALL	GO:0005830~cytosolic ribosome (sensu Eukaryota)	36	6.9E-49	34.17	5.3E-46	5.3E-46	1.0E-45
Royalblue	SP_PIR_KEYWORDS	ribosomal protein	49	1.3E-47	16.89	1.2E-44	6.0E-45	2.1E-44
Royalblue	SP_PIR_KEYWORDS	ribonucleoprotein	56	7.9E-48	12.99	7.1E-45	7.1E-45	1.2E-44
Royalblue	GOTERM_CC_ALL	GO:0005840~ribosome	50	3.6E-47	15.72	2.7E-44	1.4E-44	5.4E-44
Royalblue	SP_PIR_KEYWORDS	ribosome	33	2.6E-45	35.39	2.4E-42	7.9E-43	4.1E-42
Royalblue	GOTERM_CC_ALL	GO:0044445~cytosolic part	39	5.8E-41	20.08	4.5E-38	1.5E-38	8.9E-38
Royalblue	GOTERM_CC_ALL	GO:0030529~ribonucleoprotein complex	61	3.4E-40	8.16	2.6E-37	5.3E-38	5.3E-37
Royalblue	GOTERM_CC_ALL	GO:0033279~ribosomal subunit	38	3.4E-40	20.44	2.6E-37	6.4E-38	5.1E-37
Royalblue	GOTERM_MF_ALL	GO:0005198~structural molecule activity	53	1.2E-31	7.28	2.8E-28	1.4E-28	2.1E-28
Royalblue	GOTERM_BP_ALL	GO:0006412~translation	52	1.3E-29	6.79	5.7E-26	5.7E-26	2.5E-26
Royalblue	GOTERM_CC_ALL	GO:0005843~cytosolic small ribosomal subunit (sensu Eukaryota)	19	1.8E-26	38.32	1.4E-23	2.3E-24	2.7E-23
Royalblue	GOTERM_BP_ALL	GO:0009059~macromolecule biosynthetic process	58	3.7E-27	5.21	1.6E-23	8.1E-24	6.9E-24
Royalblue	SP_PIR_KEYWORDS	protein biosynthesis	33	4.5E-25	11.21	4.1E-22	1.0E-22	7.1E-22
Royalblue	GOTERM_CC_ALL	GO:0032991~macromolecular complex	86	1.9E-24	2.93	1.4E-21	2.1E-22	2.9E-21
Royalblue	GOTERM_CC_ALL	GO:0015935~small ribosomal subunit	21	9.7E-23	22.59	7.4E-20	9.3E-21	1.5E-19
Royalblue	GOTERM_MF_ALL	GO:0003723~RNA binding	52	1.3E-22	4.88	3.0E-19	1.0E-19	2.3E-19
Royalblue	GOTERM_CC_ALL	GO:0005842~cytosolic large ribosomal subunit (sensu Eukaryota)	17	1.9E-21	31.65	1.5E-18	1.7E-19	3.0E-18
Royalblue	GOTERM_CC_ALL	GO:0005829~cytosol	43	3.0E-21	5.77	2.3E-18	2.3E-19	4.5E-18
Royalblue	GOTERM_BP_ALL	GO:0044249~cellular biosynthetic process	58	3.6E-22	4.17	1.6E-18	5.2E-19	6.7E-19
Royalblue	GOTERM_BP_ALL	GO:0009058~biosynthetic process	65	5.0E-21	3.49	2.2E-17	5.5E-18	9.4E-18
Royalblue	GOTERM_CC_ALL	GO:0043232~intracellular non-membrane-bound organelle	64	7.5E-19	3.21	5.8E-16	4.8E-17	1.2E-15
Royalblue	GOTERM_CC_ALL	GO:0043228~non-membrane-bound organelle	64	7.5E-19	3.21	5.8E-16	4.8E-17	1.2E-15
Royalblue	GOTERM_CC_ALL	GO:0015934~large ribosomal subunit	18	1.3E-17	18.54	9.8E-15	7.5E-16	1.9E-14
Royalblue	GOTERM_BP_ALL	GO:0010467~gene expression	87	1.8E-12	1.94	7.7E-09	1.5E-09	3.3E-09
Royalblue	GOTERM_CC_ALL	GO:0044446~intracellular organelle part	84	4.3E-11	1.87	3.3E-08	2.4E-09	6.6E-08
Royalblue	GOTERM_CC_ALL	GO:0044422~organelle part	84	5.5E-11	1.86	4.2E-08	2.8E-09	8.4E-08
Royalblue	GOTERM_CC_ALL	GO:0043229~intracellular organelle	133	7.2E-11	1.36	5.5E-08	3.2E-09	1.1E-07
Royalblue	GOTERM_CC_ALL	GO:0043226~organelle	133	7.6E-11	1.36	5.9E-08	3.3E-09	1.2E-07
Royalblue	GOTERM_CC_ALL	GO:0044444~cytoplasmic part	87	6.8E-11	1.81	5.2E-08	3.3E-09	1.0E-07
Royalblue	GOTERM_CC_ALL	GO:0044424~intracellular part	143	4.4E-09	1.23	3.4E-06	1.7E-07	6.8E-06
Royalblue	GOTERM_CC_ALL	GO:0005622~intracellular	146	4.3E-09	1.20	3.3E-06	1.8E-07	6.6E-06
Royalblue	GOTERM_CC_ALL	GO:0005737~cytoplasm	113	2.1E-08	1.42	1.6E-05	7.8E-07	3.3E-05
Royalblue	GOTERM_BP_ALL	GO:0044260~cellular macromolecule metabolic process	80	2.0E-09	1.80	8.6E-06	1.4E-06	3.7E-06
Royalblue	GOTERM_BP_ALL	GO:0044267~cellular protein metabolic process	77	1.7E-08	1.76	7.3E-05	1.0E-05	3.1E-05
Royalblue	GOTERM_MF_ALL	GO:0003676~nucleic acid binding	69	6.6E-08	1.82	1.5E-04	3.8E-05	1.2E-04
Royalblue	GOTERM_BP_ALL	GO:0019538~protein metabolic process	78	1.5E-07	1.67	6.7E-04	8.3E-05	2.9E-04
Royalblue	GOTERM_CC_ALL	GO:0043234~protein complex	46	3.3E-06	1.99	2.6E-03	1.2E-04	5.1E-03
Royalblue	SP_PIR_KEYWORDS	Direct protein sequencing	54	1.2E-06	1.91	1.0E-03	2.1E-04	1.8E-03
Royalblue	GOTERM_CC_ALL	GO:0005730~nucleolus	13	7.3E-06	5.12	5.6E-03	2.4E-04	1.1E-02
Royalblue	SP_PIR_KEYWORDS	rna-binding	22	6.1E-06	3.11	5.5E-03	9.2E-04	9.5E-03
Royalblue	GOTERM_BP_ALL	GO:0043170~macromolecule metabolic process	115	2.4E-06	1.33	1.0E-02	1.1E-03	4.5E-03
Royalblue	SP_PIR_KEYWORDS	rrna-binding	5	3.3E-05	24.13	2.9E-02	4.3E-03	5.2E-02
Royalblue	GOTERM_BP_ALL	GO:0044238~primary metabolic process	122	3.3E-05	1.24	1.3E-01	1.4E-02	6.2E-02
Royalblue	COG_ONTOLOGY	Translation, ribosomal structure and biogenesis	8	2.6E-04	5.57	1.8E-02	1.8E-02	2.7E-01
Royalblue	SMART	SM00651:Sm	5	3.5E-05	24.68	1.9E-02	1.9E-02	5.1E-02
Royalblue	GOTERM_BP_ALL	GO:0044237~cellular metabolic process	121	5.3E-05	1.24	2.0E-01	2.1E-02	9.9E-02
Royalblue	GOTERM_CC_ALL	GO:0044428~nuclear part	30	9.4E-04	1.87	5.2E-01	3.0E-02	1.4E+00
Royalblue	SP_PIR_KEYWORDS	blocked amino end	8	2.9E-04	6.13	2.3E-01	3.2E-02	4.5E-01
Darkred	GOTERM_CC_ALL	GO:0005634~nucleus	77	3.9E-05	1.46	2.9E-02	2.9E-02	5.9E-02
Darkred	SP_PIR_KEYWORDS	nucleus	70	3.4E-05	1.54	3.0E-02	3.0E-02	5.3E-02
Darkgreen		No significant enrichement (Benjamini <=0.05).						
Darkturquoise		No significant enrichement (Benjamini <=0.05).						
Darkgrey	SP_PIR_KEYWORDS	phosphoprotein	74	1.0E-05	1.50	9.0E-03	9.0E-03	1.6E-02
Orange		No significant enrichement (Benjamini <=0.05).						
Darkorange	SP_PIR_KEYWORDS	phosphoprotein	79	2.6E-06	1.52	2.4E-03	2.4E-03	4.1E-03
White		No significant enrichement (Benjamini <=0.05).						
Skyblue	SP_PIR_KEYWORDS	bromodomain	8	1.3E-07	19.09	1.2E-04	5.8E-05	2.0E-04
Skyblue	SP_PIR_KEYWORDS	phosphoprotein	69	1.1E-07	1.66	9.5E-05	9.5E-05	1.6E-04
Skyblue	SP_PIR_KEYWORDS	dna-binding	28	3.3E-06	2.62	3.0E-03	7.5E-04	5.2E-03

Skyblue	SP_PIR_KEYWORDS	nucleus	53	2.6E-06	1.78	2.3E-03	7.8E-04	4.0E-03
Skyblue	INTERPRO	IPR001487:Bromodomain	8	2.0E-07	17.96	9.0E-04	9.0E-04	3.8E-04
Skyblue	SMART	SM00297:BROMO	8	1.8E-06	12.87	9.5E-04	9.5E-04	2.6E-03
Skyblue	GOTERM_MF_ALL	GO:0003676~nucleic acid binding	49	8.5E-07	1.92	1.9E-03	1.9E-03	1.5E-03
Skyblue	GOTERM_MF_ALL	GO:0003677~DNA binding	36	2.2E-06	2.25	5.0E-03	2.5E-03	3.9E-03
Skyblue	UP_SEQ_FEATURE	compositionally biased region:Poly-Lys	10	8.1E-07	9.39	8.7E-03	8.7E-03	1.7E-03
Skyblue	GOTERM_BP_ALL	GO:0032774~RNA biosynthetic process	35	1.8E-05	2.05	7.3E-02	1.1E-02	3.3E-02
Skyblue	SP_PIR_KEYWORDS	Transcription regulation	28	6.2E-05	2.23	5.4E-02	1.1E-02	9.7E-02
Skyblue	GOTERM_BP_ALL	GO:0006355~regulation of transcription, DNA-dependent	35	8.1E-06	2.13	3.4E-02	1.2E-02	1.5E-02
Skyblue	GOTERM_BP_ALL	GO:0006351~transcription, DNA-dependent	35	1.7E-05	2.06	7.2E-02	1.2E-02	3.2E-02
Skyblue	GOTERM_BP_ALL	GO:0045449~regulation of transcription	36	1.4E-05	2.04	6.1E-02	1.2E-02	2.7E-02
Skyblue	GOTERM_BP_ALL	GO:0010468~regulation of gene expression	37	2.8E-05	1.95	1.1E-01	1.3E-02	5.2E-02
Skyblue	GOTERM_BP_ALL	GO:0006139~nucleobase, nucleoside, nucleotide and nucleic acid metabolic process	50	1.2E-05	1.70	5.2E-02	1.3E-02	2.3E-02
Skyblue	GOTERM_BP_ALL	GO:0006350~transcription	38	6.2E-06	2.04	2.7E-02	1.3E-02	1.2E-02
Skyblue	GOTERM_BP_ALL	GO:0019219~regulation of nucleobase, nucleoside, nucleotide and nucleic acid metabolic process	36	2.7E-05	1.98	1.1E-01	1.5E-02	5.1E-02
Skyblue	GOTERM_BP_ALL	GO:0006325~establishment and/or maintenance of chromatin architecture	12	4.6E-05	4.62	1.8E-01	1.8E-02	8.6E-02
Skyblue	GOTERM_BP_ALL	GO:0006323~DNA packaging	12	5.0E-05	4.57	2.0E-01	1.8E-02	9.4E-02
Skyblue	SP_PIR_KEYWORDS	Transcription	28	1.3E-04	2.14	1.1E-01	1.9E-02	2.0E-01
Skyblue	GOTERM_BP_ALL	GO:0010467~gene expression	46	4.4E-05	1.70	1.7E-01	1.9E-02	8.2E-02
Skyblue	GOTERM_BP_ALL	GO:0016070~RNA metabolic process	43	5.3E-06	1.91	2.3E-02	2.3E-02	1.0E-02
Skyblue	GOTERM_BP_ALL	GO:0050794~regulation of cellular process	51	8.6E-05	1.58	3.1E-01	2.5E-02	1.6E-01
Skyblue	GOTERM_CC_ALL	GO:0005634~nucleus	60	3.4E-05	1.54	2.6E-02	2.6E-02	5.2E-02
Skyblue	GOTERM_BP_ALL	GO:0019222~regulation of metabolic process	38	8.6E-05	1.83	3.1E-01	2.6E-02	1.6E-01
Skyblue	GOTERM_BP_ALL	GO:0031323~regulation of cellular metabolic process	37	8.1E-05	1.86	3.0E-01	2.7E-02	1.5E-01
Skyblue	GOTERM_BP_ALL	GO:0006259~DNA metabolic process	18	1.0E-04	2.89	3.7E-01	2.8E-02	2.0E-01
Skyblue	GOTERM_BP_ALL	GO:0006366~transcription from RNA polymerase II promoter	17	1.2E-04	3.00	4.0E-01	2.9E-02	2.2E-01
Skyblue	GOTERM_BP_ALL	GO:0016568~chromatin modification	10	2.1E-04	4.77	6.0E-01	4.9E-02	3.9E-01
Skyblue	GOTERM_BP_ALL	GO:0051276~chromosome organization and biogenesis	12	2.2E-04	3.87	6.2E-01	5.0E-02	4.2E-01
