# Supplementary material for: A Systems Genetics Approach Implicates USF1, FADS3, and Other Causal Candidate Genes for Familial Combined Hyperlipidemia
Source: PLoS Genet. 2009 Sep 11;5(9):e1000642. doi: 10.1371/journal.pgen.1000642 (PMC2730565; doi:10.1371/journal.pgen.1000642)
Supplement: Table S6 — Trait variance explained by WGCNA co-expression module eigengenes (Adjusted R2). (0.02 MB PDF) [file pgen.1000642.s008.pdf]

Table S6. Trait variance explained by WGCNA co-expression module eigengenes (Adjusted R^2).

Module Eigengene	Percent Variance Explained (Adjusted R^2)			
	FCHL	TC	TG	ApoB
MEturquoise	0	0	0	0
MEblue	1	2	0	0
MEbrown	0	0	0	0
MEyellow	1	0	3	0
MEgreen	5	1	4	0
MEred	0	0	0	0
MEblack	9	4	4	2
MEpink	0	0	0	0
MEmagenta	6	0	2	0
MEpurple	3	0	0	0
MEgreenyellow	3	0	3	0
MEtan	10	6	17	9
MEsalmon	0	0	0	0
MEcyan	0	0	0	0
MEmidnightblue	12	1	26	8
MElightcyan	2	0	0	0
MEgrey60	0	0	0	0
MElightgreen	0	0	1	0
MElightyellow	3	0	10	0
MEroyalblue	0	0	0	0
MEdarkred	0	0	0	1
MEdarkgreen	1	0	1	0
MEdarkturquoise	7	1	8	1
MEdarkgrey	1	0	0	0
MEorange	1	0	0	0
MEdarkorange	0	0	0	0
MEwhite	0	2	0	0
MEskyblue	4	3	3	0
