# Supplementary material for: A Systems Genetics Approach Implicates USF1, FADS3, and Other Causal Candidate Genes for Familial Combined Hyperlipidemia
Source: PLoS Genet. 2009 Sep 11;5(9):e1000642. doi: 10.1371/journal.pgen.1000642 (PMC2730565; doi:10.1371/journal.pgen.1000642)
Supplement: Table S7 — Causality testing results for URFA module genes terminating in FCHL, TC, TG, and ApoB. (0.38 MB PDF) [file pgen.1000642.s009.pdf]

Table S7. Causality testing results for URFA module genes terminating in FCHL, TC, TG, and ApoB.

Probe ID	FCHL		TG		TC		ApoB	
	LEO.NB.singlemarker	Model Fitting Probabiliy	LEO.NB.singlemarker	Model Fitting Probabiliy	LEO.NB.singlemarker	Model Fitting Probabiliy	LEO.NB.singlemarker	Model Fitting Probabiliy
228641_at	0.314	1.00	0.397	0.93	-0.154	0.45	-0.399	0.31
214696_at	0.313	1.00	0.425	0.99	-0.554	0.18	-0.625	0.19
209740_s_at	0.312	1.00	0.428	1.00	-0.431	0.24	-0.406	0.31
236664_at	0.310	0.99	0.415	0.97	-0.478	0.21	-0.342	0.36
205404_at	0.309	0.99	0.397	0.93	-0.380	0.26	-0.414	0.30
205452_at	0.308	0.99	0.280	0.71	-0.208	0.39	-0.135	0.58
214033_at	0.307	0.98	0.419	0.98	-0.427	0.24	-0.370	0.33
212799_at	0.306	0.98	0.423	0.99	-0.519	0.19	-0.393	0.32
214152_at	0.306	0.98	0.367	0.87	-0.475	0.21	-0.369	0.34
203925_at	0.306	0.98	0.334	0.80	-0.497	0.20	-0.477	0.26
204057_at	0.305	0.98	0.398	0.93	-0.296	0.32	-0.458	0.27
227117_at	0.305	0.98	0.392	0.92	-0.512	0.20	-0.500	0.25
214252_s_at	0.303	0.97	0.267	0.69	-0.134	0.47	-0.191	0.50
209268_at	0.303	0.98	0.251	0.67	-0.373	0.27	-0.204	0.49
229144_at	0.301	0.97	0.275	0.70	-0.450	0.23	-0.503	0.25
203625_x_at	0.301	0.97	0.403	0.94	-0.364	0.27	-0.152	0.55
204257_at	0.301	0.97	0.373	0.88	-0.254	0.35	-0.354	0.35
50374_at	0.300	0.97	0.297	0.74	-0.406	0.25	-0.287	0.40
203517_at	0.298	0.96	0.319	0.78	-0.336	0.29	-0.187	0.51
221229_s_at	0.298	0.96	0.201	0.59	-0.455	0.22	-0.211	0.48
229302_at	0.297	0.96	0.305	0.75	-0.224	0.38	-0.252	0.44
201821_s_at	0.296	0.96	0.419	0.98	-0.267	0.34	-0.391	0.32
201876_at	0.295	0.96	0.375	0.88	-0.425	0.24	-0.400	0.31
213790_at	0.295	0.96	0.391	0.92	-0.364	0.27	-0.392	0.32
221511_x_at	0.295	0.96	0.428	1.00	-0.544	0.18	-0.433	0.29
203359_s_at	0.294	0.96	0.419	0.98	-0.478	0.21	-0.345	0.35
228937_at	0.294	0.95	0.383	0.90	-0.274	0.34	-0.212	0.48
208712_at	0.294	0.95	0.407	0.95	-0.383	0.26	-0.389	0.32
213581_at	0.294	0.96	0.230	0.63	-0.268	0.34	-0.266	0.43
222278_at	0.293	0.95	0.414	0.97	-0.434	0.23	-0.402	0.31
227442_at	0.292	0.95	0.275	0.70	-0.400	0.25	-0.397	0.31
221276_s_at	0.292	0.95	0.370	0.87	-0.313	0.31	-0.373	0.33
223186_at	0.292	0.95	0.412	0.96	-0.372	0.27	-0.436	0.29
222426_at	0.292	0.95	0.428	1.00	-0.389	0.26	-0.430	0.29
226742_at	0.290	0.95	0.405	0.95	-0.246	0.36	-0.251	0.44
217835_x_at	0.289	0.94	0.390	0.91	-0.262	0.35	-0.300	0.39
202693_s_at	0.289	0.94	0.343	0.82	-0.241	0.36	-0.254	0.44
238890_at	0.288	0.94	0.306	0.75	-0.633	0.15	-0.504	0.25

226436_at	0.288	0.94	0.402	0.94	-0.399	0.25	-0.363	0.34
213513_x_at	0.287	0.94	0.414	0.97	-0.174	0.43	-0.351	0.35
215983_s_at	0.286	0.94	0.409	0.96	-0.347	0.29	-0.214	0.48
225480_at	0.284	0.93	0.399	0.93	-0.351	0.28	-0.320	0.38
217751_at	0.283	0.93	0.249	0.66	-0.242	0.36	-0.255	0.44
212572_at	0.283	0.93	0.351	0.84	-0.354	0.28	-0.466	0.27
202998_s_at	0.283	0.93	0.352	0.84	-0.367	0.27	-0.365	0.34
210145_at	0.283	0.93	0.246	0.66	-0.230	0.37	-0.294	0.40
219307_at	0.282	0.93	0.416	0.97	-0.514	0.19	-0.379	0.33
203592_s_at	0.281	0.93	0.338	0.81	-0.302	0.32	-0.316	0.38
218296_x_at	0.281	0.93	0.387	0.91	-0.339	0.29	-0.359	0.34
227138_at	0.280	0.92	0.415	0.97	-0.417	0.24	-0.434	0.29
205055_at	0.280	0.92	0.276	0.70	-0.466	0.22	-0.480	0.26
202796_at	0.279	0.92	0.393	0.92	-0.368	0.27	-0.410	0.30
203031_s_at	0.278	0.92	0.396	0.93	-0.169	0.43	-0.226	0.47
213702_x_at	0.277	0.92	0.401	0.94	-0.432	0.24	-0.397	0.31
230925_at	0.277	0.92	0.335	0.81	-0.372	0.27	-0.449	0.28
201387_s_at	0.277	0.92	0.421	0.98	-0.305	0.31	-0.419	0.30
220990_s_at	0.276	0.92	0.308	0.76	-0.227	0.38	-0.204	0.49
47560_at	0.276	0.92	0.291	0.73	-0.417	0.24	-0.386	0.32
217922_at	0.276	0.92	0.385	0.90	-0.411	0.25	-0.415	0.30
218547_at	0.275	0.91	0.377	0.89	-0.497	0.20	-0.454	0.28
206833_s_at	0.275	0.91	0.293	0.73	-0.371	0.27	-0.311	0.38
201843_s_at	0.274	0.91	0.373	0.88	-0.320	0.30	-0.274	0.42
219374_s_at	0.274	0.91	0.396	0.93	-0.320	0.30	-0.293	0.40
206662_at	0.274	0.91	0.423	0.99	-0.300	0.32	-0.378	0.33
202088_at	0.273	0.91	0.308	0.76	-0.455	0.22	-0.261	0.43
219454_at	0.273	0.91	0.310	0.76	-0.270	0.34	-0.416	0.30
226851_at	0.271	0.90	0.261	0.68	-0.395	0.26	-0.276	0.41
203647_s_at	0.270	0.90	0.428	1.00	-0.312	0.31	-0.298	0.39
202814_s_at	0.270	0.90	0.386	0.91	-0.355	0.28	-0.431	0.29
227665_at	0.268	0.90	0.307	0.76	-0.398	0.25	-0.372	0.33
227009_at	0.268	0.90	0.362	0.86	-0.361	0.28	-0.294	0.40
235198_at	0.267	0.90	0.383	0.90	-0.466	0.22	-0.385	0.32
226731_at	0.266	0.90	0.426	0.99	-0.342	0.29	-0.315	0.38
224469_s_at	0.266	0.90	0.404	0.95	-0.215	0.39	-0.294	0.40
225783_at	0.266	0.89	0.334	0.80	-0.163	0.44	-0.152	0.55
223020_at	0.266	0.89	0.349	0.83	-0.336	0.29	-0.329	0.37
205499_at	0.265	0.89	0.404	0.95	-0.297	0.32	-0.326	0.37
226343_at	0.265	0.89	0.356	0.85	-0.266	0.34	-0.298	0.40
223880_x_at	0.265	0.89	0.417	0.97	-0.185	0.41	-0.217	0.48
224831_at	0.264	0.89	0.382	0.90	-0.232	0.37	-0.126	0.59
219543_at	0.263	0.89	0.421	0.98	-0.792	0.10	-0.518	0.24

213045_at	0.263	0.89	0.267	0.69	-0.316	0.31	-0.275	0.42
222665_at	0.263	0.89	0.405	0.95	-0.550	0.18	-0.587	0.20
242304_at	0.263	0.89	0.228	0.63	-0.210	0.39	-0.348	0.35
229126_at	0.262	0.89	0.352	0.84	-0.632	0.15	-0.674	0.17
201012_at	0.262	0.89	0.365	0.86	-0.294	0.32	-0.331	0.37
212333_at	0.262	0.89	0.348	0.83	-0.439	0.23	-0.400	0.31
214202_at	0.261	0.88	0.189	0.58	-0.170	0.43	-0.069	0.67
203545_at	0.261	0.89	0.383	0.90	-0.425	0.24	-0.244	0.45
226416_at	0.260	0.88	0.330	0.80	-0.214	0.39	-0.215	0.48
226810_at	0.259	0.88	0.367	0.87	-0.187	0.41	-0.266	0.42
238032_at	0.259	0.88	0.424	0.99	-0.381	0.26	-0.441	0.28
226935_s_at	0.259	0.88	0.313	0.77	-0.429	0.24	-0.416	0.30
204971_at	0.258	0.88	0.324	0.79	-0.371	0.27	-0.255	0.44
203218_at	0.258	0.88	0.348	0.83	-0.402	0.25	-0.336	0.36
226196_s_at	0.257	0.88	0.346	0.83	-0.284	0.33	-0.259	0.43
203502_at	0.256	0.87	0.296	0.74	-0.174	0.43	-0.180	0.52
202756_s_at	0.255	0.87	0.330	0.80	-0.323	0.30	-0.390	0.32
230282_at	0.255	0.87	0.383	0.90	-0.417	0.24	-0.454	0.28
225841_at	0.255	0.87	0.413	0.97	-0.268	0.34	-0.282	0.41
207988_s_at	0.253	0.87	0.379	0.89	-0.159	0.44	-0.280	0.41
224233_s_at	0.253	0.87	0.350	0.83	-0.301	0.32	-0.268	0.42
226195_at	0.253	0.87	0.257	0.67	-0.200	0.40	-0.141	0.57
215171_s_at	0.251	0.86	0.350	0.83	-0.414	0.24	-0.475	0.26
211725_s_at	0.251	0.86	0.259	0.68	-0.102	0.50	-0.166	0.53
235509_at	0.251	0.87	0.231	0.63	-0.127	0.47	-0.131	0.58
220326_s_at	0.251	0.86	0.303	0.75	-0.387	0.26	-0.424	0.30
204141_at	0.250	0.86	0.396	0.93	-0.202	0.40	-0.316	0.38
201124_at	0.249	0.86	0.362	0.86	-0.356	0.28	-0.345	0.35
214719_at	0.248	0.86	0.361	0.85	-0.274	0.34	-0.218	0.47
225083_at	0.248	0.86	0.261	0.68	-0.201	0.40	-0.200	0.49
201722_s_at	0.247	0.86	0.375	0.88	-0.322	0.30	-0.274	0.42
224960_at	0.247	0.86	0.293	0.73	-0.240	0.37	-0.130	0.58
209903_s_at	0.247	0.86	0.295	0.73	-0.240	0.37	-0.224	0.47
209270_at	0.247	0.86	0.422	0.98	-0.438	0.23	-0.402	0.31
223211_at	0.246	0.86	0.331	0.80	-0.235	0.37	-0.304	0.39
58780_s_at	0.245	0.85	0.330	0.80	-0.434	0.23	-0.445	0.28
204839_at	0.244	0.85	0.293	0.73	-0.414	0.24	-0.404	0.31
212780_at	0.242	0.85	0.328	0.79	-0.310	0.31	-0.369	0.34
224773_at	0.242	0.85	0.415	0.97	-0.304	0.32	-0.293	0.40
224436_s_at	0.241	0.85	0.354	0.84	-0.232	0.37	-0.195	0.50
222877_at	0.241	0.85	0.365	0.86	-0.251	0.36	-0.258	0.43
216080_s_at	0.241	0.84	0.306	0.75	-0.224	0.38	-0.307	0.39
222403_at	0.240	0.84	0.379	0.89	-0.479	0.21	-0.380	0.33

225881_at	0.239	0.84	0.422	0.98	-0.187	0.41	-0.127	0.58
203658_at	0.238	0.84	0.420	0.98	-0.311	0.31	-0.274	0.42
212880_at	0.237	0.84	0.336	0.81	-0.218	0.38	-0.192	0.50
208776_at	0.237	0.84	0.288	0.72	-0.207	0.39	-0.284	0.41
212296_at	0.237	0.84	0.371	0.88	-0.269	0.34	-0.344	0.35
211137_s_at	0.236	0.83	0.298	0.74	-0.148	0.45	-0.204	0.49
227160_s_at	0.236	0.84	0.220	0.62	-0.179	0.42	-0.245	0.45
218664_at	0.234	0.83	0.327	0.79	-0.372	0.27	-0.350	0.35
210830_s_at	0.234	0.83	0.378	0.89	-0.791	0.10	-0.745	0.14
218837_s_at	0.234	0.83	0.365	0.86	-0.367	0.27	-0.366	0.34
219064_at	0.234	0.83	0.342	0.82	-0.534	0.19	-0.563	0.21
229549_at	0.234	0.83	0.413	0.96	-0.151	0.45	-0.324	0.37
210980_s_at	0.234	0.83	0.368	0.87	-0.330	0.30	-0.412	0.30
214151_s_at	0.234	0.83	0.334	0.80	-0.607	0.16	-0.472	0.26
228368_at	0.232	0.83	0.398	0.93	-0.176	0.42	-0.313	0.38
213478_at	0.232	0.83	0.245	0.65	-0.388	0.26	-0.358	0.34
222156_x_at	0.231	0.83	0.357	0.85	-0.553	0.18	-0.503	0.25
225108_at	0.231	0.83	0.395	0.93	-0.254	0.35	-0.258	0.43
202059_s_at	0.231	0.83	0.292	0.73	-0.270	0.34	-0.276	0.41
225573_at	0.231	0.83	0.245	0.65	-0.453	0.22	-0.303	0.39
223599_at	0.231	0.83	0.353	0.84	-0.345	0.29	-0.455	0.27
228488_at	0.231	0.83	0.374	0.88	-0.337	0.29	-0.382	0.33
226805_at	0.231	0.83	0.267	0.69	-0.277	0.34	-0.214	0.48
202952_s_at	0.230	0.82	0.328	0.79	-0.298	0.32	-0.266	0.42
229948_at	0.229	0.82	0.318	0.77	-0.259	0.35	-0.245	0.45
229415_at	0.229	0.82	0.366	0.86	-0.423	0.24	-0.416	0.30
224376_s_at	0.228	0.82	0.387	0.91	-0.197	0.40	-0.247	0.44
203661_s_at	0.227	0.82	0.380	0.89	-0.227	0.38	-0.332	0.37
209160_at	0.227	0.82	0.342	0.82	-0.243	0.36	-0.302	0.39
219582_at	0.227	0.82	0.203	0.59	-0.224	0.38	-0.232	0.46
227068_at	0.226	0.82	0.350	0.84	-0.234	0.37	-0.216	0.48
229958_at	0.226	0.82	0.232	0.64	-0.286	0.33	-0.284	0.41
223204_at	0.226	0.82	0.402	0.94	-0.229	0.37	-0.325	0.37
225252_at	0.226	0.82	0.169	0.55	-0.119	0.48	-0.157	0.55
203769_s_at	0.225	0.81	0.329	0.79	-0.287	0.33	-0.308	0.39
202815_s_at	0.225	0.81	0.327	0.79	-0.192	0.41	-0.317	0.38
222622_at	0.225	0.81	0.355	0.84	-0.164	0.44	-0.273	0.42
210740_s_at	0.224	0.81	0.295	0.73	-0.293	0.32	-0.302	0.39
203662_s_at	0.224	0.81	0.422	0.98	-0.195	0.41	-0.356	0.35
220327_at	0.223	0.81	0.375	0.88	-0.245	0.36	-0.338	0.36
209208_at	0.221	0.81	0.312	0.76	-0.168	0.43	-0.141	0.57
227962_at	0.220	0.81	0.270	0.69	-0.343	0.29	-0.314	0.38
235333_at	0.220	0.81	0.316	0.77	-0.306	0.31	-0.324	0.37

232235_at	0.219	0.80	0.396	0.93	-0.374	0.27	-0.607	0.19
202670_at	0.218	0.80	0.354	0.84	-0.260	0.35	-0.256	0.44
226339_at	0.217	0.80	0.393	0.92	-0.342	0.29	-0.263	0.43
219646_at	0.217	0.80	0.421	0.98	-0.420	0.24	-0.534	0.23
212390_at	0.217	0.80	0.296	0.74	-0.331	0.30	-0.303	0.39
209822_s_at	0.215	0.80	0.360	0.85	-0.292	0.32	-0.341	0.36
209025_s_at	0.214	0.79	0.262	0.68	-0.327	0.30	-0.276	0.42
225562_at	0.214	0.79	0.206	0.60	-0.343	0.29	-0.255	0.44
223113_at	0.214	0.79	0.411	0.96	-0.435	0.23	-0.521	0.24
213902_at	0.214	0.80	0.259	0.68	-0.238	0.37	-0.242	0.45
242760_x_at	0.213	0.79	0.257	0.67	-0.162	0.44	-0.064	0.68
202055_at	0.212	0.79	0.251	0.66	-0.175	0.43	-0.157	0.55
57588_at	0.212	0.79	0.364	0.86	-0.395	0.26	-0.343	0.36
208724_s_at	0.211	0.79	0.279	0.71	-0.263	0.35	-0.153	0.55
207621_s_at	0.211	0.79	0.396	0.93	-0.326	0.30	-0.380	0.33
203303_at	0.210	0.79	0.219	0.62	-0.307	0.31	-0.279	0.41
203327_at	0.209	0.79	0.253	0.67	-0.177	0.42	-0.145	0.56
201899_s_at	0.209	0.79	0.301	0.75	-0.305	0.31	-0.245	0.45
205335_s_at	0.209	0.79	0.347	0.83	-0.187	0.41	-0.235	0.46
205110_s_at	0.208	0.78	0.230	0.63	-0.223	0.38	-0.210	0.48
209902_at	0.207	0.78	0.390	0.91	-0.648	0.14	-0.565	0.21
207196_s_at	0.207	0.78	0.245	0.65	-0.276	0.34	-0.267	0.42
225440_at	0.206	0.78	0.272	0.70	-0.252	0.36	-0.284	0.41
200015_s_at	0.206	0.78	0.418	0.97	-0.191	0.41	-0.261	0.43
227531_at	0.205	0.78	0.228	0.63	-0.092	0.51	-0.058	0.69
210312_s_at	0.205	0.78	0.197	0.59	-0.171	0.43	-0.133	0.58
228084_at	0.204	0.78	0.204	0.60	-0.194	0.41	-0.152	0.55
225200_at	0.204	0.78	0.334	0.80	-0.278	0.33	-0.223	0.47
212886_at	0.204	0.78	0.251	0.66	-0.331	0.30	-0.243	0.45
229004_at	0.203	0.77	0.255	0.67	-0.156	0.44	-0.157	0.55
218732_at	0.201	0.77	0.421	0.98	-0.244	0.36	-0.367	0.34
220065_at	0.201	0.77	0.272	0.70	-0.557	0.18	-0.686	0.16
224180_x_at	0.201	0.77	0.306	0.75	-0.517	0.19	-0.282	0.41
225176_at	0.201	0.77	0.224	0.62	-0.195	0.41	-0.178	0.52
203008_x_at	0.200	0.77	0.381	0.90	-0.159	0.44	-0.227	0.46
226059_at	0.199	0.77	0.248	0.66	-0.084	0.52	-0.143	0.56
237154_at	0.199	0.77	0.294	0.73	-0.216	0.39	-0.255	0.44
201898_s_at	0.197	0.76	0.297	0.74	-0.205	0.40	-0.176	0.52
235010_at	0.197	0.76	0.222	0.67	-0.290	0.33	-0.223	0.47
227278_at	0.196	0.76	0.341	0.82	-0.197	0.40	-0.265	0.43
203536_s_at	0.195	0.76	0.266	0.69	-0.216	0.39	-0.162	0.54
222108_at	0.195	0.76	0.204	0.60	-0.221	0.38	-0.249	0.44
219603_s_at	0.194	0.76	0.319	0.78	-0.560	0.17	-0.559	0.22

207400_at	0.194	0.76	0.282	0.71	-0.290	0.33	-0.233	0.46
200736_s_at	0.193	0.76	0.280	0.71	-0.207	0.40	-0.212	0.48
228568_at	0.193	0.76	0.234	0.64	-0.236	0.37	-0.218	0.47
209616_s_at	0.192	0.76	0.297	0.74	-0.219	0.38	-0.267	0.42
237390_at	0.191	0.75	0.234	0.64	-0.240	0.37	-0.236	0.45
215128_at	0.191	0.75	0.315	0.77	-0.182	0.42	-0.087	0.64
221797_at	0.191	0.75	0.178	0.56	-0.320	0.30	-0.166	0.54
223215_s_at	0.190	0.75	0.297	0.74	-0.146	0.45	-0.033	0.73
205618_at	0.189	0.75	0.261	0.68	-0.108	0.50	-0.132	0.58
217911_s_at	0.189	0.75	0.204	0.60	-0.228	0.38	-0.176	0.52
227550_at	0.188	0.75	0.270	0.69	-0.137	0.46	-0.270	0.42
208071_s_at	0.188	0.75	0.277	0.70	-0.202	0.40	-0.336	0.36
215947_s_at	0.188	0.75	0.132	0.50	-0.068	0.54	-0.070	0.67
207092_at	0.187	0.75	0.282	0.71	-0.207	0.39	-0.241	0.45
229573_at	0.186	0.74	0.278	0.71	-0.214	0.39	-0.139	0.57
227158_at	0.186	0.74	0.254	0.67	-0.185	0.41	-0.168	0.53
235747_at	0.186	0.74	0.259	0.68	-0.294	0.32	-0.264	0.43
209841_s_at	0.185	0.74	0.308	0.76	-0.132	0.47	-0.276	0.41
214434_at	0.185	0.74	0.342	0.82	-0.276	0.34	-0.303	0.39
205006_s_at	0.184	0.74	0.304	0.75	-0.240	0.37	-0.253	0.44
239648_at	0.184	0.74	0.263	0.68	-0.218	0.38	-0.198	0.50
226997_at	0.183	0.74	0.205	0.60	-0.186	0.41	-0.134	0.58
211758_x_at	0.183	0.74	0.376	0.89	-0.098	0.51	-0.146	0.56
218099_at	0.183	0.74	0.212	0.61	-0.269	0.34	-0.324	0.37
218756_s_at	0.183	0.74	0.187	0.57	-0.178	0.42	-0.144	0.56
215559_at	0.182	0.74	0.253	0.67	-0.177	0.42	-0.148	0.56
218219_s_at	0.182	0.74	0.235	0.64	-0.298	0.32	-0.233	0.46
200953_s_at	0.181	0.74	0.294	0.73	-0.285	0.33	-0.338	0.36
225684_at	0.181	0.74	0.241	0.65	-0.212	0.39	-0.135	0.58
218360_at	0.180	0.73	0.245	0.65	-0.181	0.42	-0.153	0.55
241359_at	0.180	0.73	0.364	0.86	-0.556	0.18	-0.671	0.17
217772_s_at	0.180	0.73	0.280	0.71	-0.192	0.41	-0.205	0.49
204079_at	0.179	0.73	0.249	0.66	-0.230	0.37	-0.154	0.55
204767_s_at	0.178	0.73	0.406	0.95	-0.191	0.41	-0.179	0.52
243813_at	0.178	0.73	0.360	0.85	-0.178	0.42	-0.304	0.39
203367_at	0.177	0.73	0.208	0.60	-0.222	0.38	-0.206	0.49
218516_s_at	0.175	0.73	0.354	0.84	-0.262	0.35	-0.215	0.48
236075_s_at	0.175	0.73	0.277	0.70	-0.214	0.39	-0.141	0.57
222894_x_at	0.175	0.73	-0.074	0.48	-0.160	0.44	-0.180	0.52
215743_at	0.175	0.73	0.212	0.61	-0.159	0.44	-0.124	0.59
205201_at	0.175	0.73	0.263	0.68	-0.111	0.58	-0.094	0.79
201468_s_at	0.174	0.72	0.291	0.73	-0.177	0.42	-0.207	0.49
224983_at	0.173	0.72	0.275	0.71	-0.076	0.53	-0.119	0.60

226777_at	0.173	0.72	0.310	0.76	-0.613	0.15	-0.566	0.21
229449_at	0.171	0.72	0.221	0.62	-0.179	0.42	-0.284	0.41
218286_s_at	0.171	0.72	0.170	0.55	-0.176	0.65	-0.098	0.79
203323_at	0.170	0.72	0.316	0.77	-0.340	0.29	-0.375	0.33
209694_at	0.169	0.72	0.164	0.54	-0.081	0.53	-0.092	0.78
213616_at	0.169	0.72	0.215	0.61	-0.201	0.40	-0.155	0.55
227476_at	0.168	0.71	0.241	0.65	-0.250	0.36	-0.256	0.43
225788_at	0.168	0.71	0.268	0.69	-0.138	0.46	-0.212	0.48
209377_s_at	0.168	0.71	-0.144	0.45	-0.070	0.54	0.039	0.86
212554_at	0.167	0.71	0.171	0.55	-0.202	0.40	-0.194	0.50
200655_s_at	0.167	0.71	0.314	0.77	-0.107	0.50	-0.151	0.55
238554_at	0.167	0.71	0.319	0.78	-0.283	0.33	-0.188	0.51
222686_s_at	0.165	0.71	0.249	0.66	-0.251	0.36	-0.226	0.47
213242_x_at	0.164	0.71	0.239	0.65	-0.235	0.37	-0.270	0.42
212334_at	0.162	0.70	0.232	0.70	-0.231	0.37	-0.205	0.49
212857_x_at	0.162	0.70	0.277	0.70	-0.283	0.33	-0.273	0.42
210896_s_at	0.161	0.70	0.252	0.67	-0.255	0.35	-0.268	0.42
223158_s_at	0.159	0.70	0.274	0.70	-0.210	0.39	-0.203	0.49
200788_s_at	0.158	0.70	0.230	0.63	-0.202	0.40	-0.177	0.52
218699_at	0.157	0.70	0.042	0.52	-0.177	0.42	-0.163	0.54
210946_at	0.157	0.70	0.260	0.68	-0.183	0.42	-0.192	0.50
223342_at	0.157	0.70	0.243	0.65	-0.200	0.40	-0.152	0.55
219090_at	0.156	0.70	0.208	0.65	-0.304	0.32	-0.251	0.44
201199_s_at	0.156	0.70	0.232	0.64	-0.232	0.37	-0.241	0.45
201032_at	0.155	0.69	0.239	0.65	-0.241	0.36	-0.188	0.51
204394_at	0.155	0.69	0.201	0.59	-0.104	0.50	-0.207	0.49
218176_at	0.154	0.69	0.238	0.64	-0.177	0.42	-0.123	0.59
229063_s_at	0.153	0.69	0.200	0.59	-0.135	0.47	-0.169	0.53
227064_at	0.152	0.69	0.206	0.60	-0.155	0.44	-0.140	0.57
231166_at	0.151	0.69	0.421	0.98	-0.293	0.32	-0.234	0.46
219664_s_at	0.151	0.69	0.182	0.57	-0.186	0.41	-0.178	0.52
204218_at	0.150	0.69	0.219	0.62	-0.098	0.51	-0.168	0.53
223100_s_at	0.150	0.69	0.233	0.64	-0.157	0.44	-0.112	0.61
226923_at	0.149	0.68	0.284	0.72	-0.122	0.48	-0.204	0.49
227336_at	0.148	0.68	0.179	0.56	-0.254	0.35	-0.220	0.47
212388_at	0.148	0.68	0.176	0.56	-0.224	0.38	-0.262	0.43
224130_s_at	0.147	0.68	0.193	0.58	-0.146	0.45	-0.215	0.48
209135_at	0.147	0.68	0.251	0.66	-0.282	0.33	-0.329	0.37
200782_at	0.146	0.68	0.274	0.70	-0.170	0.43	-0.177	0.52
205440_s_at	0.146	0.68	0.257	0.67	-0.211	0.39	-0.212	0.48
210106_at	0.144	0.68	0.221	0.62	-0.141	0.46	-0.104	0.62
204276_at	0.144	0.68	0.300	0.74	-0.229	0.37	-0.249	0.44
222150_s_at	0.143	0.67	0.425	0.99	-0.351	0.28	-0.508	0.24

225670_at	0.143	0.68	0.346	0.83	-0.278	0.33	-0.199	0.50
201266_at	0.143	0.67	0.295	0.73	-0.128	0.47	-0.144	0.56
218124_at	0.143	0.67	0.167	0.55	-0.237	0.37	-0.175	0.52
223253_at	0.143	0.67	0.195	0.64	-0.136	0.46	-0.143	0.56
204001_at	0.143	0.67	0.269	0.69	0.066	0.76	0.066	0.91
219933_at	0.142	0.67	0.007	0.51	-0.087	0.57	-0.056	0.69
223239_at	0.142	0.67	0.294	0.73	-0.196	0.40	-0.175	0.52
204341_at	0.141	0.67	0.136	0.58	-0.111	0.49	-0.153	0.55
212226_s_at	0.141	0.67	0.204	0.60	-0.215	0.39	-0.209	0.48
218078_s_at	0.141	0.67	0.246	0.66	-0.227	0.38	-0.218	0.47
224691_at	0.141	0.67	0.276	0.70	-0.164	0.44	-0.197	0.50
209934_s_at	0.140	0.67	0.200	0.59	-0.119	0.48	-0.050	0.70
224064_s_at	0.140	0.67	0.191	0.58	-0.307	0.31	-0.239	0.45
202070_s_at	0.140	0.67	0.234	0.64	-0.118	0.48	-0.097	0.63
1552501_a_at	0.139	0.67	0.237	0.64	-0.157	0.44	-0.178	0.52
202348_s_at	0.139	0.67	0.181	0.56	-0.079	0.53	-0.144	0.56
240869_at	0.138	0.67	0.151	0.53	-0.190	0.41	-0.172	0.53
210644_s_at	0.137	0.67	0.254	0.67	-0.140	0.46	-0.200	0.50
230257_s_at	0.137	0.67	0.135	0.51	-0.064	0.55	-0.103	0.62
221727_at	0.136	0.66	0.306	0.75	-0.302	0.32	-0.311	0.38
213203_at	0.135	0.66	0.201	0.59	-0.110	0.58	-0.065	0.76
225804_at	0.134	0.66	0.215	0.61	-0.230	0.37	-0.184	0.51
205633_s_at	0.134	0.66	0.181	0.56	-0.140	0.46	-0.164	0.54
202762_at	0.134	0.66	0.167	0.60	-0.209	0.39	-0.139	0.57
220966_x_at	0.132	0.66	0.140	0.51	-0.127	0.47	-0.106	0.62
202138_x_at	0.132	0.66	0.252	0.67	-0.128	0.47	-0.126	0.59
212777_at	0.132	0.66	0.231	0.63	-0.174	0.43	-0.235	0.46
210519_s_at	0.132	0.66	0.225	0.63	-0.132	0.47	-0.161	0.54
221667_s_at	0.131	0.66	0.182	0.57	-0.132	0.47	-0.118	0.60
212788_x_at	0.131	0.66	0.121	0.64	-0.128	0.47	-0.173	0.53
226915_s_at	0.130	0.65	0.108	0.48	-0.056	0.56	-0.077	0.66
218669_at	0.130	0.65	0.415	0.97	-0.677	0.13	-0.485	0.26
221047_s_at	0.129	0.65	0.178	0.56	-0.185	0.41	-0.179	0.52
218934_s_at	0.128	0.65	0.145	0.52	-0.212	0.39	-0.184	0.51
230174_at	0.127	0.65	0.144	0.52	-0.188	0.41	-0.089	0.64
201467_s_at	0.127	0.65	0.221	0.62	-0.117	0.49	-0.157	0.55
224391_s_at	0.126	0.65	0.165	0.54	-0.270	0.34	-0.174	0.53
212667_at	0.126	0.65	0.253	0.67	-0.232	0.37	-0.249	0.44
209123_at	0.126	0.65	0.152	0.53	-0.195	0.41	-0.097	0.63
211340_s_at	0.126	0.65	0.129	0.50	-0.141	0.46	-0.136	0.57
209971_x_at	0.125	0.65	0.223	0.62	-0.101	0.50	-0.082	0.65
225087_at	0.125	0.65	-0.231	0.44	-0.131	0.47	-0.039	0.72
204209_at	0.125	0.65	0.198	0.59	-0.216	0.39	-0.226	0.47

215091_s_at	0.124	0.65	0.111	0.48	-0.077	0.53	-0.121	0.59
212053_at	0.123	0.64	0.062	0.54	-0.228	0.38	-0.143	0.56
224616_at	0.123	0.64	0.148	0.52	-0.101	0.50	-0.041	0.71
218404_at	0.123	0.64	0.268	0.69	-0.093	0.51	-0.253	0.44
239039_at	0.123	0.64	0.141	0.52	-0.158	0.44	-0.187	0.51
226214_at	0.123	0.64	0.155	0.53	-0.125	0.48	-0.133	0.58
211985_s_at	0.123	0.64	0.290	0.73	-0.169	0.43	-0.100	0.62
221796_at	0.122	0.64	0.300	0.74	-0.229	0.38	-0.162	0.54
201420_s_at	0.121	0.64	0.114	0.48	-0.102	0.61	-0.032	0.73
219054_at	0.121	0.64	0.228	0.63	-0.165	0.43	-0.239	0.45
209877_at	0.121	0.64	0.237	0.64	-0.052	0.56	-0.136	0.57
205428_s_at	0.120	0.64	0.124	0.52	-0.125	0.48	-0.103	0.62
205005_s_at	0.119	0.64	0.221	0.62	-0.206	0.40	-0.188	0.51
222449_at	0.119	0.64	0.170	0.55	-0.185	0.41	-0.151	0.55
236901_at	0.118	0.64	0.189	0.58	-0.050	0.57	-0.078	0.66
229831_at	0.118	0.64	-0.019	0.52	-0.112	0.49	-0.106	0.62
212297_at	0.118	0.64	0.254	0.67	-0.107	0.50	-0.076	0.66
222561_at	0.116	0.63	0.243	0.65	-0.256	0.35	-0.165	0.54
201837_s_at	0.116	0.63	0.108	0.48	-0.101	0.50	-0.036	0.72
228817_at	0.116	0.63	0.216	0.61	-0.649	0.14	-0.572	0.21
209398_at	0.115	0.63	0.081	0.53	-0.133	0.47	-0.103	0.62
209087_x_at	0.114	0.63	0.003	0.48	-0.126	0.48	-0.112	0.61
232180_at	0.114	0.63	-0.030	0.47	-0.197	0.40	-0.160	0.54
203646_at	0.113	0.63	0.297	0.74	-0.112	0.49	-0.170	0.53
207431_s_at	0.112	0.63	0.237	0.64	-0.218	0.39	-0.145	0.56
227399_at	0.109	0.62	0.206	0.60	-0.101	0.50	-0.107	0.61
222450_at	0.107	0.62	0.197	0.59	-0.202	0.40	-0.137	0.57
200713_s_at	0.107	0.62	0.129	0.63	-0.143	0.46	-0.126	0.59
226607_at	0.107	0.69	-0.042	0.55	-0.171	0.43	-0.111	0.61
227290_at	0.106	0.62	-0.007	0.51	-0.108	0.50	-0.126	0.59
202709_at	0.105	0.62	0.280	0.71	-0.349	0.28	-0.259	0.43
222654_at	0.104	0.62	0.284	0.72	-0.223	0.38	-0.169	0.53
241936_x_at	0.103	0.62	0.115	0.50	-0.011	0.70	-0.071	0.77
215000_s_at	0.102	0.61	0.000	0.52	-0.072	0.54	-0.059	0.68
225011_at	0.101	0.61	0.013	0.47	-0.075	0.54	-0.078	0.66
221599_at	0.100	0.61	0.179	0.56	-0.180	0.42	-0.125	0.59
201579_at	0.100	0.61	0.256	0.67	-0.117	0.49	-0.163	0.54
212335_at	0.100	0.61	0.213	0.61	-0.185	0.42	-0.137	0.57
224681_at	0.099	0.61	-0.035	0.54	-0.142	0.46	-0.132	0.58
200665_s_at	0.099	0.61	0.267	0.69	-0.208	0.39	-0.168	0.53
239398_at	0.099	0.61	0.225	0.63	-0.133	0.47	-0.144	0.56
212653_s_at	0.098	0.61	0.193	0.58	-0.116	0.49	-0.142	0.57
205074_at	0.098	0.61	0.100	0.47	-0.108	0.50	-0.044	0.71

202544_at	0.097	0.61	0.156	0.53	-0.115	0.49	-0.079	0.65
238604_at	0.096	0.61	0.172	0.55	-0.056	0.56	-0.039	0.76
212055_at	0.095	0.60	0.051	0.53	-0.147	0.45	-0.145	0.56
222351_at	0.095	0.60	0.106	0.48	-0.099	0.51	-0.096	0.63
214126_at	0.095	0.63	0.194	0.58	-0.183	0.42	-0.121	0.59
202883_s_at	0.095	0.60	0.204	0.60	-0.143	0.46	-0.069	0.67
212872_s_at	0.094	0.60	0.087	0.52	-0.127	0.47	-0.049	0.70
238106_at	0.094	0.60	0.188	0.58	-0.145	0.45	-0.206	0.49
209191_at	0.093	0.60	0.131	0.50	-0.056	0.56	-0.108	0.61
212371_at	0.093	0.60	0.118	0.49	-0.137	0.46	-0.108	0.61
214733_s_at	0.092	0.60	0.098	0.47	-0.036	0.58	-0.068	0.67
209250_at	0.092	0.60	0.170	0.55	-0.088	0.52	-0.076	0.66
212971_at	0.088	0.59	-0.236	0.42	-0.126	0.48	-0.095	0.63
213217_at	0.088	0.59	0.179	0.58	-0.089	0.66	-0.018	0.75
224864_at	0.087	0.61	0.261	0.68	-0.112	0.49	-0.191	0.50
221012_s_at	0.085	0.59	0.203	0.60	-0.159	0.44	-0.145	0.56
222409_at	0.085	0.59	-0.043	0.50	-0.076	0.53	-0.116	0.60
225330_at	0.085	0.59	0.101	0.47	-0.121	0.48	-0.118	0.60
219314_s_at	0.085	0.62	-0.054	0.52	-0.137	0.46	-0.132	0.58
223144_s_at	0.084	0.59	-0.031	0.50	-0.134	0.47	-0.133	0.58
239017_at	0.083	0.59	0.154	0.53	-0.088	0.60	-0.026	0.74
227792_at	0.082	0.61	-0.055	0.52	-0.146	0.45	-0.098	0.63
226653_at	0.081	0.58	0.163	0.54	-0.111	0.49	-0.061	0.68
204239_s_at	0.079	0.58	0.157	0.53	-0.160	0.44	-0.162	0.54
201338_x_at	0.079	0.58	0.091	0.46	-0.050	0.57	-0.099	0.63
203431_s_at	0.079	0.58	0.255	0.67	-0.043	0.58	-0.091	0.64
203801_at	0.074	0.58	0.403	0.94	-0.606	0.16	-0.616	0.19
213617_s_at	0.073	0.57	-0.049	0.48	-0.115	0.49	-0.106	0.61
200978_at	0.069	0.62	0.216	0.61	-0.206	0.40	-0.185	0.51
213187_x_at	0.067	0.60	-0.074	0.53	-0.105	0.50	-0.130	0.58
203827_at	0.065	0.56	0.003	0.49	-0.140	0.46	-0.116	0.60
209935_at	0.065	0.65	-0.300	0.43	-0.192	0.41	-0.048	0.70
238992_at	0.063	0.56	0.128	0.50	-0.063	0.55	-0.043	0.71
224675_at	0.062	0.56	-0.048	0.52	-0.048	0.57	-0.031	0.73
222824_at	0.062	0.56	0.270	0.69	-0.842	0.09	-0.677	0.17
201125_s_at	0.054	0.64	-0.100	0.56	-0.203	0.40	-0.179	0.52
215543_s_at	0.053	0.60	0.171	0.55	-0.073	0.57	-0.116	0.60
213727_x_at	0.051	0.55	-0.073	0.47	-0.047	0.57	-0.054	0.69
205204_at	0.050	0.54	0.086	0.47	-0.087	0.52	-0.085	0.65
231839_at	0.049	0.56	0.091	0.46	-0.102	0.61	-0.037	0.72
226671_at	0.048	0.61	-0.088	0.48	-0.113	0.49	-0.062	0.68
209707_at	0.045	0.55	-0.087	0.48	-0.101	0.50	-0.082	0.65
226184_at	0.045	0.54	-0.080	0.44	-0.054	0.56	-0.046	0.70

221752_at	0.044	0.61	-0.100	0.52	-0.118	0.48	-0.082	0.65
218095_s_at	0.039	0.63	0.287	0.72	-0.170	0.64	-0.168	0.53
212097_at	0.037	0.57	0.150	0.53	-0.141	0.46	-0.132	0.58
203042_at	0.037	0.53	-0.072	0.43	-0.054	0.56	-0.021	0.75
209840_s_at	0.036	0.61	0.212	0.61	-0.092	0.51	-0.204	0.49
221255_s_at	0.034	0.53	-0.089	0.42	-0.174	0.64	-0.013	0.80
223312_at	0.034	0.60	0.225	0.63	-0.138	0.46	-0.129	0.58
218447_at	0.028	0.52	0.300	0.74	-0.483	0.21	-0.564	0.21
213836_s_at	0.027	0.61	0.195	0.58	-0.252	0.36	-0.143	0.56
212551_at	0.023	0.54	0.072	0.44	-0.060	0.55	-0.086	0.64
207469_s_at	0.022	0.61	-0.117	0.49	-0.083	0.53	-0.084	0.65
225593_at	0.005	0.61	0.298	0.74	-0.099	0.58	-0.039	0.74
226178_at	0.005	0.58	0.199	0.59	-0.069	0.58	-0.040	0.72
205609_at	-0.005	0.59	0.256	0.67	-0.207	0.39	-0.227	0.47
201200_at	-0.011	0.55	0.141	0.52	-0.064	0.55	-0.035	0.72
225856_at	-0.011	0.55	0.082	0.46	-0.097	0.60	-0.025	0.74
223515_s_at	-0.013	0.66	-0.039	0.57	0.017	0.78	-0.072	0.82
225651_at	-0.014	0.57	-0.166	0.50	-0.086	0.52	-0.088	0.64
224583_at	-0.021	0.63	-0.174	0.50	-0.110	0.49	-0.143	0.56
224586_x_at	-0.023	0.59	0.240	0.65	-0.190	0.41	-0.235	0.46
200653_s_at	-0.023	0.59	0.299	0.74	-0.076	0.56	-0.093	0.63
218138_at	-0.027	0.55	-0.303	0.40	-0.122	0.48	-0.054	0.80
217626_at	-0.029	0.53	-0.156	0.43	-0.045	0.57	-0.090	0.64
244444_at	-0.035	0.58	-0.198	0.50	-0.077	0.53	-0.125	0.59
208711_s_at	-0.054	0.58	0.344	0.82	-0.130	0.47	-0.215	0.48
204277_s_at	-0.055	0.56	-0.192	0.45	-0.088	0.52	-0.097	0.63
227960_s_at	-0.056	0.57	0.007	0.48	-0.113	0.60	0.059	0.90
201724_s_at	-0.070	0.58	0.223	0.62	-0.138	0.61	-0.034	0.73
219446_at	-0.071	0.56	-0.151	0.45	-0.179	0.42	-0.053	0.69
211984_at	-0.071	0.54	0.142	0.52	-0.070	0.54	-0.045	0.71
219789_at	-0.074	0.53	0.166	0.55	-0.069	0.54	-0.147	0.56
209244_s_at	-0.077	0.59	-0.225	0.47	-0.110	0.49	-0.111	0.61
222977_at	-0.077	0.53	0.120	0.49	-0.071	0.54	-0.096	0.63
203065_s_at	-0.077	0.41	-0.070	0.45	-0.051	0.56	-0.074	0.66
200028_s_at	-0.095	0.52	-0.242	0.44	-0.034	0.59	-0.033	0.73
228233_at	-0.099	0.54	-0.251	0.45	-0.064	0.55	-0.051	0.70
212457_at	-0.102	0.54	-0.240	0.43	-0.058	0.56	-0.060	0.68
228299_at	-0.105	0.57	-0.249	0.45	-0.097	0.51	-0.079	0.65
226774_at	-0.112	0.45	-0.311	0.39	-0.054	0.66	-0.014	0.76
200660_at	-0.112	0.58	-0.288	0.47	-0.090	0.52	-0.105	0.62
208970_s_at	-0.118	0.53	0.090	0.46	-0.053	0.60	-0.027	0.74
201198_s_at	-0.121	0.55	0.214	0.61	-0.101	0.50	-0.185	0.51
203324_s_at	-0.125	0.55	0.162	0.54	-0.134	0.47	-0.126	0.59

218605_at	-0.133	0.57	0.029	0.54	-0.132	0.47	-0.036	0.72
206918_s_at	-0.134	0.36	-0.191	0.32	0.085	0.77	0.069	0.92
208633_s_at	-0.134	0.51	0.002	0.48	-0.045	0.57	-0.055	0.69
208689_s_at	-0.136	0.51	-0.290	0.42	-0.026	0.61	-0.034	0.73
219108_x_at	-0.137	0.52	0.167	0.55	-0.054	0.56	-0.079	0.65
225698_at	-0.138	0.46	-0.237	0.40	-0.087	0.61	-0.025	0.74
229125_at	-0.138	0.55	-0.271	0.42	-0.156	0.62	-0.052	0.70
208795_s_at	-0.143	0.35	-0.326	0.35	0.076	0.76	0.078	0.94
221059_s_at	-0.151	0.58	-0.312	0.45	-0.064	0.55	-0.102	0.62
217875_s_at	-0.156	0.52	0.121	0.49	-0.114	0.49	-0.105	0.62
214211_at	-0.159	0.55	-0.355	0.44	-0.116	0.50	-0.110	0.61
238806_at	-0.160	0.53	-0.315	0.42	-0.082	0.56	-0.077	0.66
201319_at	-0.163	0.53	0.124	0.50	-0.111	0.67	-0.066	0.67
221788_at	-0.164	0.56	-0.250	0.28	-0.124	0.48	-0.040	0.71
205743_at	-0.174	0.50	-0.320	0.40	-0.072	0.60	-0.024	0.74
227326_at	-0.184	0.40	-0.287	0.32	0.014	0.71	0.034	0.85
201201_at	-0.193	0.52	-0.371	0.41	-0.105	0.57	-0.049	0.70
239942_at	-0.202	0.50	-0.192	0.40	-0.075	0.66	-0.012	0.76
202422_s_at	-0.203	0.51	0.122	0.49	-0.176	0.63	-0.035	0.72
201489_at	-0.204	0.52	-0.203	0.43	-0.141	0.62	-0.058	0.76
212349_at	-0.205	0.43	-0.347	0.31	-0.037	0.69	0.041	0.86
238718_at	-0.208	0.44	-0.331	0.41	-0.120	0.60	-0.072	0.67
202808_at	-0.219	0.52	0.178	0.56	-0.267	0.34	-0.149	0.56
219283_at	-0.219	0.51	0.177	0.56	-0.132	0.62	-0.028	0.76
227074_at	-0.224	0.45	0.137	0.51	-0.145	0.62	-0.104	0.62
201485_s_at	-0.225	0.45	-0.324	0.41	-0.078	0.53	-0.062	0.68
201490_s_at	-0.225	0.52	-0.101	0.45	-0.095	0.59	-0.063	0.68
213282_at	-0.227	0.44	-0.348	0.36	-0.174	0.43	-0.055	0.74
222978_at	-0.239	0.50	-0.033	0.46	-0.025	0.60	-0.032	0.73
222717_at	-0.247	0.50	-0.260	0.35	-0.047	0.57	-0.022	0.75
212506_at	-0.254	0.51	-0.389	0.39	-0.135	0.58	-0.045	0.71
209563_x_at	-0.262	0.51	0.192	0.58	-0.164	0.63	-0.042	0.71
224327_s_at	-0.304	0.49	-0.254	0.41	-0.062	0.55	-0.067	0.67
202402_s_at	-0.306	0.49	-0.406	0.37	-0.031	0.59	-0.033	0.73
228109_at	-0.311	0.49	-0.413	0.37	-0.182	0.63	-0.090	0.78
208091_s_at	-0.322	0.47	-0.485	0.32	-0.187	0.63	-0.095	0.78
