# Supplementary material for: A Systems Genetics Approach Implicates USF1, FADS3, and Other Causal Candidate Genes for Familial Combined Hyperlipidemia
Source: PLoS Genet. 2009 Sep 11;5(9):e1000642. doi: 10.1371/journal.pgen.1000642 (PMC2730565; doi:10.1371/journal.pgen.1000642)
Supplement: Table S8 — Previous association evidence with lipid or atherogenic traits for genes causally linked to FCHL from the URFA module. (0.07 MB PDF) [file pgen.1000642.s010.pdf]

Table S8. Previous association evidence with lipid or atherogenic trais for genes causally linked to FCHL from the URFA module.

Gene	Disease	PubMed ID	Year
ABCC6	CAD	12176944	2002
ABCC6	HDL	11776382	2001
ABCC6	PSE	19284998	2009
ABCC6	PSE	18440309	2008
ABCC6	PSE	17617515	2007
ABCC6	PSE	16835894	2006
ABCC6	PSE	15727254	2004
ABCC6	PSE	14631379	2004
ABCC6	TG	11776382	2001
GCLM	Average IMT	19126404	2009
GCLM	CHD	17961430	2007
GCLM	MI	12081989	2002
GCLM	Vascular Cognitive Impairment	17548779	2007
FADS3	Arachadonic acid	19148276	2009
FADS3	Arachadonic acid	18479586	2009
FADS3	Arachadonic acid	18320251	2008
FADS3	Arachadonic acid	17284757	2007
FADS3	Arachadonic acid:Linoleic acid	18842780	2008
FADS3	Di-homo-γ-linoleic acid	18479586	2009
FADS3	Eicosadienoic acid	19148276	2009
FADS3	Eicosapentanoic acid	19148276	2009
FADS3	HDL	19060906	2009
FADS3	LDL	19148276	2009
FADS3	LDL (HDL,TG)	19060911	2009
FADS3	Poly Unsaturated Fatty Acids	18320251	2008
FADS3	TG	19060906	2009
FADS3	TG	17284757	2007
IRF8	Hepatitis C, Chronic	16886895	2006
HSD11B1	Body Composition	12861241	2003
HSD11B1	Hypertension	15452033	2004
HSD11B1	Type II Diabetes	15156315	2004
AKT2	Metabolic Syndrome	17576055	2008
AKT2	TC/HDL ratio	17576055	2008
AKT2	Type II Diabetes	15166380	2004
CLN5	Ceroid Lipofuscinosis, Neuronal	19201763	2009
CLN5	Ceroid Lipofuscinosis, Neuronal	19309691	2009
CLN5	Ceroid Lipofuscinosis, Neuronal	17607606	2007
CLN5	Ceroid Lipofuscinosis, Neuronal	16814585	2006
CLN5	Ceroid Lipofuscinosis, Neuronal	15728307	2005
CLN5	Ceroid Lipofuscinosis, Neuronal	15459477	2004
CLN5	Ceroid Lipofuscinosis, Neuronal	10953198	2000
CLN5	Ceroid Lipofuscinosis, Neuronal	10191122	1999
CLN5	Ceroid Lipofuscinosis, Neuronal	9662406	1998
CLN5	Ceroid Lipofuscinosis, Neuronal	9662406	1998
CARD8	Alzheimers	19252766	2009
CARD8	Alzheimers	18841008	2008
CARD8	Rheumatoid arthritis	18263599	2008
CARD8	Rheumatoid arthritis	17878386	2007
