# Supplementary material for: A Systems Genetics Approach Implicates USF1, FADS3, and Other Causal Candidate Genes for Familial Combined Hyperlipidemia
Source: PLoS Genet. 2009 Sep 11;5(9):e1000642. doi: 10.1371/journal.pgen.1000642 (PMC2730565; doi:10.1371/journal.pgen.1000642)
Supplement: Table S9 — qRT-PCR primers for validation of genes identified as differentially expressed due to USF1 over-expression on microarrays. (0.01 MB PDF) [file pgen.1000642.s011.pdf]

Table S9. qRT-PCR primers for calidation of genes identified as differentially expressed due to *USF1* over-expression on microarrays.

Gene	Forward Primer	Reverse Primer	cDNA PCR Product Lentgth	Type
AGT	AACTGGTGCTGCAAGGATCT	CTCTCATCCGCTTCAAGCTC	163	Microarray Validation
AK5	AGGTGAAGCAAGGGGAAGAG	GATGGACGCTCGGTAGTAGG	182	Microarray Validation
CNR1	GTCCTGATCCTGGTGGTGTT	CGCAGGTCCTTACTCCTCAG	179	Microarray Validation
GALR2	TGGACATCTGCACCTTCGTCTTCA	GATGAGGATCATGCGTGTCACCTT	161	Microarray Validation
IGFBP7	GACAACCTGGCCATTCAGAC	CTGTCCTTGGGAATTGGATG	129	Microarray Validation
KISS1R	CGTTCGGTGCAGTTTCGTTGTGAA	CTGGAATGATCCAGAAAGTCCTGTG	108	Microarray Validation
PLXDC1	CATCATCCTGGCTGGAATTT	GCATAGGTGGAATGGTCAGG	129	Microarray Validation
PLXNC1	ATTTGCCTCCATTGTCATCC	CTTCCAGCCCTCGTTCTCTT	168	Microarray Validation
PROK2	TGTGACAAGGACTCCCAATG	AGACATGGGCAAGTGTGATG	167	Microarray Validation
B2M	GTCTTTCAGCAAGGACTGGTC	CAAATGCGGCATCTTCAAACC	173	Housekeeping Control
HPRT	TATGGCGACCCGCAGCCCT	CATCTCGAGCAAGACGTTCAG	158	Housekeeping Control
